# A new bacteriolytic amidase Ami of *Lysobacter capsici* XL1

**DOI:** 10.1038/s41598-025-07691-2

**Published:** 2025-07-01

**Authors:** Irina Kudryakova, Alexey Afoshin, Sergey Tarlachkov, Sofya Pavlenko, Natalia Suzina, Nina Shishkova, Elena Leontyevskaya, Natalia Leontyevskaya

**Affiliations:** 1 Laboratory of Microbial Cell Surface Biochemistry, G.K. Skryabin Institute of Biochemistry and Physiology of Microorganisms, FRC PSCBR, Russian Academy of Sciences, 5 Prosp. Nauki, Pushchino, Moscow Region Russia 142290; 2 Lomonosov Moscow State University, Leninskiye Gory, Moscow, Russia 119899; 3 FBIS State Research Center for Applied Biotechnology and Microbiology, 24 Kvartal-A Territory, Obolensk, Russia 142279

**Keywords:** *Lysobacter*, Bacterial bacteriolytic enzyme, Omics, Homologous expression system, New bacteriolytic amidase, Peptidoglycan types, Antimicrobials, Bacteria, Hydrolases

## Abstract

One of the most pressing issues in modern biomedicine is the search for new antimicrobial agents – antibiotics, peptides, bacteriolytic enzymes. This study, using transcriptomic and proteomic approaches, identified a new extracellular bacteriolytic enzyme of *Lysobacter capsici* XL1 – the amidase Ami. The enzyme was isolated and characterized. Ami was found to hydrolyze the amide bond between the carbohydrate and peptide fragments in bacterial peptidoglycans of chemotypes A1γ, A3α, and A4α. Ami lysed live target cells of opportunistic bacteria *Micrococcus luteus* Ac-2230^T^, *Bacillus cereus* 217, *Staphylococcus aureus* 209P, *Enterococcus faecium* FS86, phytopathogenic bacteria *Bacillus megaterium* MS941, *Curtobacterium flaccumfaciens* pv. *flaccumfaciens*, and pathogenic bacteria of various strains of *B. anthracis*, including plasmid strains 71/12 and ΔAmes, as well as strains of *B. cereus* with hemolytic, lecithinase, and phosphatase activities. Thus, the bacteriolytic amidase Ami is a promising candidate for the development of next-generation antimicrobial drugs.

**Supplementary Information:**

The online version contains supplementary material available at 10.1038/s41598-025-07691-2.

## Introduction

Bacteriolytic enzymes are antimicrobial agents that target various bonds in bacterial peptidoglycan. Based on the type of hydrolyzed bond, they can be divided into three groups: glucosaminidases, which cleave the β-(1–4)-glycosidic bond between N-acetylglucosamine and N-acetylmuramic acid; amidases (N-acetylmuramoyl-L-alanine amidases), which hydrolyze the amide bond between residues of N-acetylmuramic acid and L-alanine in the peptide backbone; and bacteriolytic proteases, which hydrolyze peptide bonds within the peptide fragment of peptidoglycan. The specificity of their action makes these enzymes highly relevant in addressing the problem of antibiotic resistance.

The most active producers of bacteriolytic enzymes are bacteria. All bacteria synthesize intracellular bacteriolytic enzymes (autolysins) necessary for their own growth and cell division^1^. This is the most well-studied group of bacteriolytic enzymes. Some bacteria produce extracellular bacteriolytic enzymes. These enzymes act on the cells of competing bacteria, providing the producer with nutrients, and facilitate the colonization of ecological niches. Extracellular bacteriolytic enzymes of bacteria are the focus of our interest.

The first identified extracellular bacteriolytic enzymes were lysostaphin (M23B bacteriolytic metalloprotease) of *Staphylococcus* bv. *staphylolyticus*^2^ and the α- and β-lytic proteases from *Lysobacter enzymogenes* ATCC29487^3^. To date, in addition to these, other extracellular bacteriolytic metalloproteases from the M23A family, such as LasA from *Pseudomonas aeruginosa* Paks I^4,5^ and pseudoalterin from *Pseudoalteromonas* sp. CF6-2^6^, have been isolated and studied to varying degrees. Metalloproteases from the M23B family, including millericin B from *Streptococcus milleri* NMSCC 061^7^, enterolysin A from *Enterococcus faecalis* DPC5280^8,9^, zoocin A from *Streptococcus equi* subsp. *zooepidemicus* 4881^10,11^ and Ale-1 from *Staphylococcus capitis* EPK1^12^, amidase CwhA from *L. enzymogenes* M497-1^13^, have also been identified. Most bacteriolytic enzymes have been isolated, however, from the bacterium *Lysobacter* (strains XL1 and VKM B-2533^T^) we are studying. It should be noted that lytically active species of the genus *Lysobacter* are considered an inexhaustible source of new antimicrobial agents, including not only bacteriolytic enzymes but also antibiotics, antimicrobial peptides, and anti-nematode peptides^14–18^.

The lytically active strain *L. capsici* XL1 has been studied by our team since 1975. The strain has demonstrated potent antimicrobial activity against pathogenic and opportunistic bacteria such as *S. aureus* (including clinical MRSA strains), *S. pneumoniae*, *S. pyogenes*, *Micrococcus luteus*, *Bacillus cereus*, *E. faecium*, vegetative and spore forms of *B. anthracis*, yeasts of the genera *Candida* and *Trichosporon*, and phytopathogenic fungi like *Aspergillus niger*, *Fusarium solani*, and *Sclerotinia sclerotiorum*^19–21^. Recently, we have actively studied the type strain VKM B-2533^T^, which has also shown antibacterial and antifungal activities^22^. The lytic activity of this species has been found to be due to its ability to secrete a complex of bacteriolytic enzymes with various specificities.

Currently, 12 bacteriolytic enzymes have been isolated from and partially characterized in *L. capsici* XL1 and VKM B-2533^T^: bacteriolytic proteases Serp, Serp3, Serp6, Serp7, Blp, Mep, L1, L4, L5, amidase L2, N-acetylglucosaminidase NAG, muramidase L3^17,22–24^. We have determined that the staphylolytic activity of these strains is linked to the production of β-lytic protease (Blp) which is also active against *M. luteus* Ac-2230^T^, *S. pneumoniae* 19 F, *S. pyogenes* 367, *Kocuria rosea* Ac-2200^T^, and *E. faecium* FS86^22^. L5 has shown activity against live cells of *M. luteus* Ac-2230^T^ and yeast cells of the genus *Candida*^25^. L4 has been shown to be active against live cells of *M. luteus* Ac-2230^T^.

We have long been interested in whether bacteriolytic enzyme(s) are responsible for the activity against bacteria of the genus *Bacillus*, or if this activity is due to the synergistic effect of all extracellular bacteriolytic enzymes of *L. capsici* XL1.

Thus, the aim of this study was to search for new bacteriolytic enzymes from *L. capsici* XL1. For this, we employed transcriptomic and proteomic analyses of two strains: *L. capsici* XL1, an active producer of bacteriolytic enzymes, and *L. capsici* XL2, a strain that has nearly lost this ability^26^. Our assumption was that comparative studies of these strains would help identify active genes encoding bacteriolytic enzymes in strain XL1 and enable the isolation of a new enzyme.

## Results

### Proteomic and transcriptomic approaches to the search for bacteriolytic enzymes in *L. capsici*

For transcriptomic and proteomic analyses, cell and culture-fluid samples were taken after a 21-h cultivation of strain XL1 and reference strain XL2 under identical conditions (Supplementary file Fig. S1). By this time of cultivation, both strains had reached the end of the exponential growth phase (OD_540_ = 5.0). The bacteriolytic activity of the culture fluid from strain XL1 was 163 LU/mL, while no activity was detected in that of strain XL2. The lack of a bacteriolytic activity in strain XL2 is an important prerequisite for comparative proteomic and transcriptomic studies aimed at identifying active target genes and secreted enzymes in strain XL1.

Transcriptomic analysis revealed that 1,584 genes in strain XL1 were expressed at higher levels compared to strain XL2. Among these, 350 genes had expression levels more than five times higher (*p*_adj_ < 0.05). The technical results of the RNA-seq analysis are given in Supplementary file Table S1. All sequencing and alignment statistics are presented in Supplementary file Table S4.

Proteomic analysis identified 489 proteins in the culture fluid of strain XL1 and 849 proteins in strain XL2 (Supplementary file Table S4). The total protein concentration in the culture fluid of strain XL1 was 0.685 ± 0.006 mg/mL, which was six times higher than the 0.116 ± 0.023 mg/mL concentration observed in strain XL2. Among the 462 common proteins, the production of 186 proteins differed between the strains by more than twofold (–log (*p*) > 1).

**Fig. 1:**
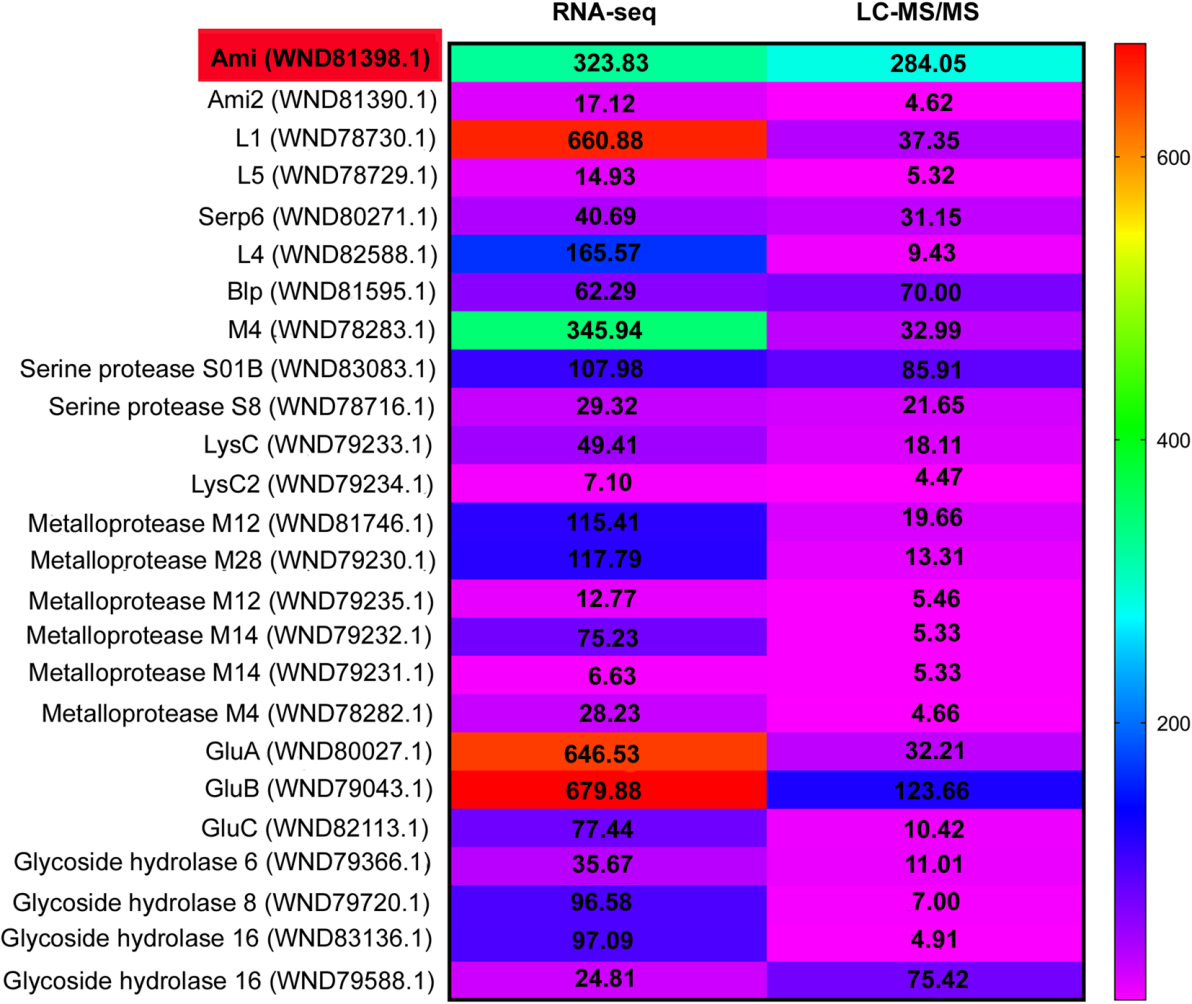
Heatmap based on proteomic (–log (*p*) > 1) and transcriptomic (*p*_adj_ < 0.05) data for *L. capsici* XL1. *L. capsici* XL2 was used as the reference strain.

The proteomic and transcriptomic results were compared (Supplementary file Table S4), which enabled the detection of 25 extracellular proteins with significantly higher concentrations in the culture fluid of strain XL1 (Fig. 1).

Among these proteins, we found previously identified bacteriolytic enzymes from *L. capsici*, such as L1, L4, L5, Blp, Serp6, and Mep (M4). Their gene expression levels were significantly elevated in strain XL1 by 660.88, 165.57, 14.93, 62.29, 40.69, and 345.94 times, respectively, compared to strain XL2. The concentrations of these proteins in the culture fluid of strain XL1 were higher by 37.35, 9.43, 5.32, 70.00, 31.15, and 32.99 times, respectively. Notably, the content of glucanases GluA, GluB, and GluC also increased by 32.21, 123.66, and 10.42 times, respectively.

Among the proteins with presumed bacteriolytic activity, we were interested in serine proteases from families S8 and S1D, metalloproteases from families M4, M12, M14, and M28, and glycosyl hydrolases from families 6, 8, and 16. Special attention was paid to the protein WND81398.1 (locus tag RJ610_03180), annotated as a Zn-dependent N-acetylmuramoyl-L-alanine amidase, which we designated as Ami. The expression level of the *ami* gene was 323.83 times higher, and the production level of the Ami amidase was 284.05 times higher in strain XL1 compared to strain XL2. We hypothesized that the Ami enzyme could be important for the antimicrobial properties of *L. capsici* XL1 to be revealed, and chose it for further study.

### Phylogenetic analysis

**Fig. 2:**
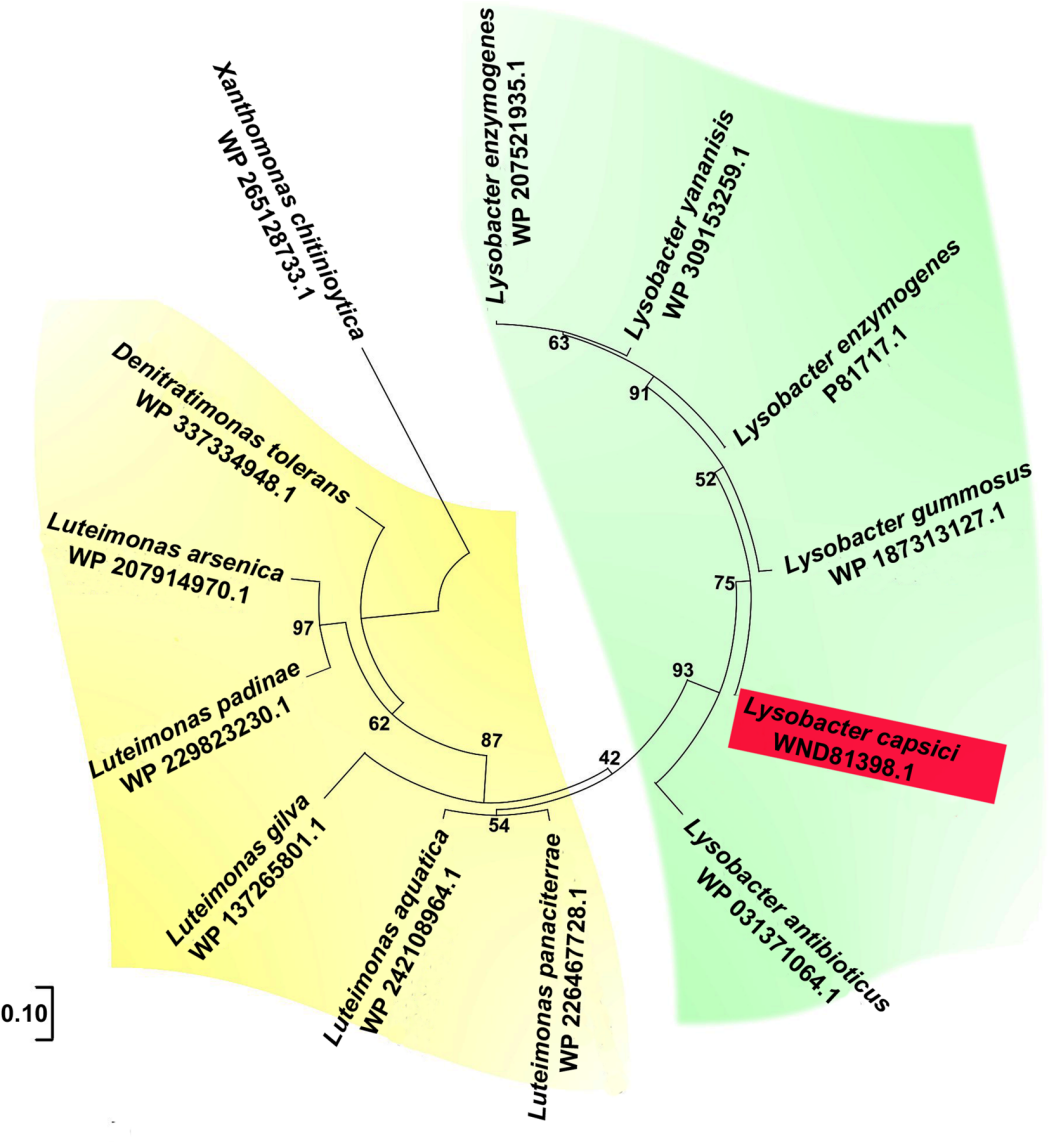
Dendrogram of extracellular amidases with > 50% identity to Ami, constructed using the maximum likelihood method. Bootstrap percentages (1,000 iterations) are shown at branching points. The scale corresponds to 10 amino acid substitutions per 100 residues. Ami from *L. capsici* XL1 is highlighted in red.

Phylogenetic analysis of Ami and secreted amidases from bacteria with over 50% identity to Ami (based on the NCBI database) identified two clusters (highlighted in yellow and green in the dendrogram) (Fig. 2). The Ami enzyme of strain XL1 belongs to a clade with extracellular amidases from other lytically active species of *Lysobacter* (*L. antibioticus*, *L. gummosus*, *L. enzymogenes*) and *L. yananisis*, for which no lytic potential has been described. The second clade comprises extracellular amidases from the genera *Luteimonas* and *Denitratimonas*.

Among the amidases represented on the dendrogram, only the amidase CwhA (P81717) of *L. enzymogenes* M497-1 (previously *Achromobacter lyticus*), with a molecular mass of 19.4 kDa, has been isolated and partially characterized^13^. The amino acid sequences of the mature parts of Ami and CwhA are 95% identical with 65% coverage (Supplementary file Fig. S2).

### Isolation and biochemical characterization of Ami

**Fig. 3:**
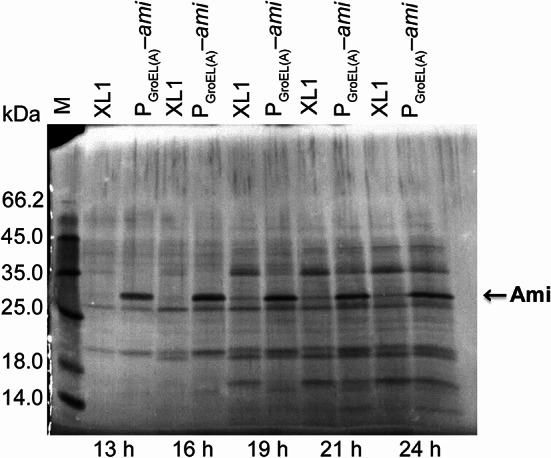
SDS-PAGE of proteins in the culture fluid of *L. capsici* XL1 and *L. capsici* P_GroEL(A)_ –*ami*.

A homologous expression system was developed to isolate the Ami enzyme. The growth rate of the expression strain *L. capsici* P_GroEL(A)_–*ami* was comparable to that of the wild-type strain XL1 (Supplementary file Fig. S3). The bacteriolytic activity of the culture fluid from the expression strain was maximal at 19 h of cultivation, reaching 366 LU/mL, which was twice as high as that of strain XL1. Electrophoretic analysis revealed that a protein with a molecular mass of 30 kDa was predominant in the culture fluid of the expression strain, corresponding to the calculated molecular mass of the mature Ami enzyme (Fig. 3).

**Fig. 4:**
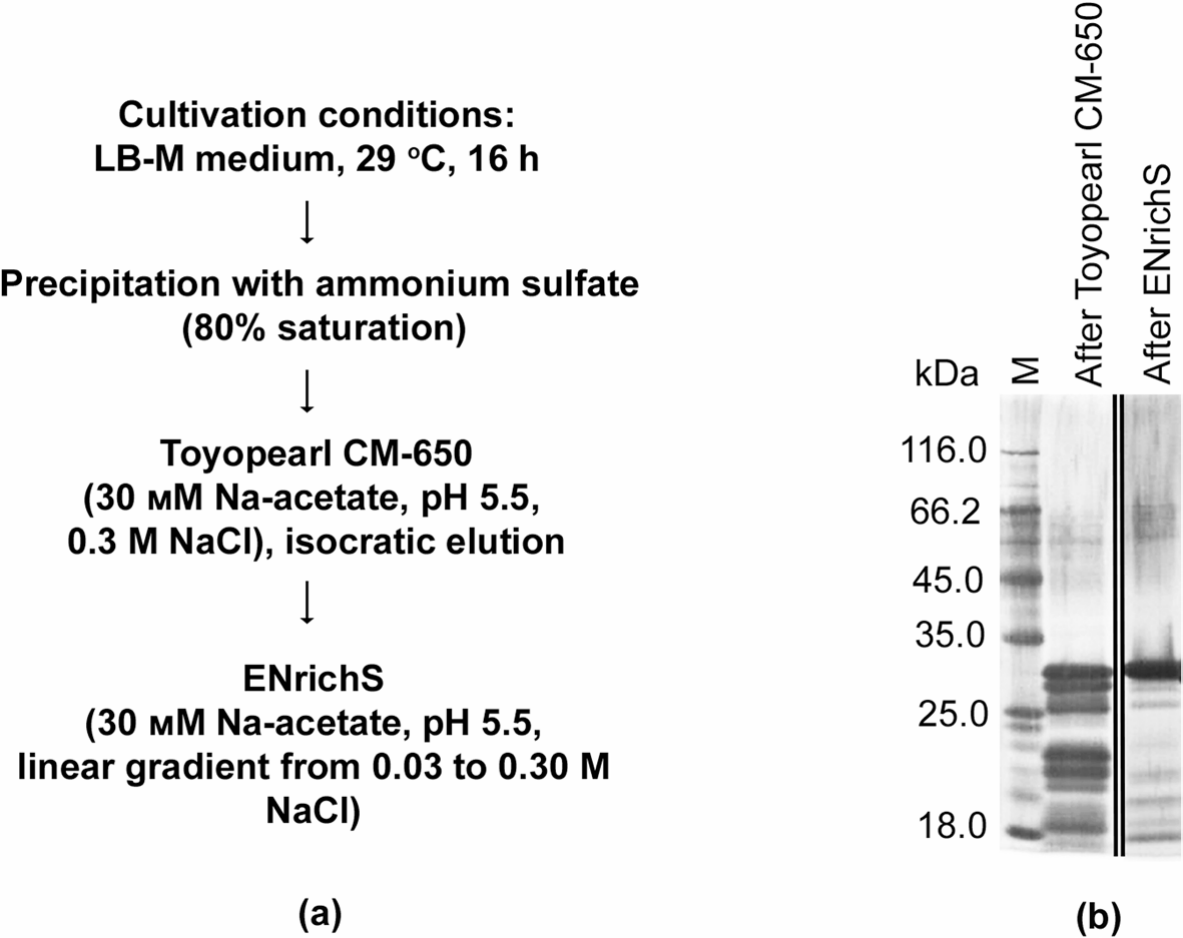
Purification of the bacteriolytic enzyme Ami from the culture fluid of *L. capsici* P_GroEL(A)_–*ami.* **(a**) Purification scheme. An Ami purification chromatogram on an EnrichS column is presented in Supplementary file Fig. S4b; **(b)** SDS-PAGE (see original figure in Supplementary file Fig. S4a).

The Ami enzyme was isolated from the culture fluid of *L. capsici* P_GroEL(A)_–*ami* according to the developed scheme (Fig. 4a). SDS-PAGE of Ami is shown in Fig. 4b. The homogeneity of the produced preparation was confirmed by gel filtration (Supplementary file Fig. S4c, d) and MALDI-TOF analysis. The concentration of the purified enzyme was 0.05 mg/mL. The Ami purification table is given in Supplementary file Table S2.

**Fig. 5:**
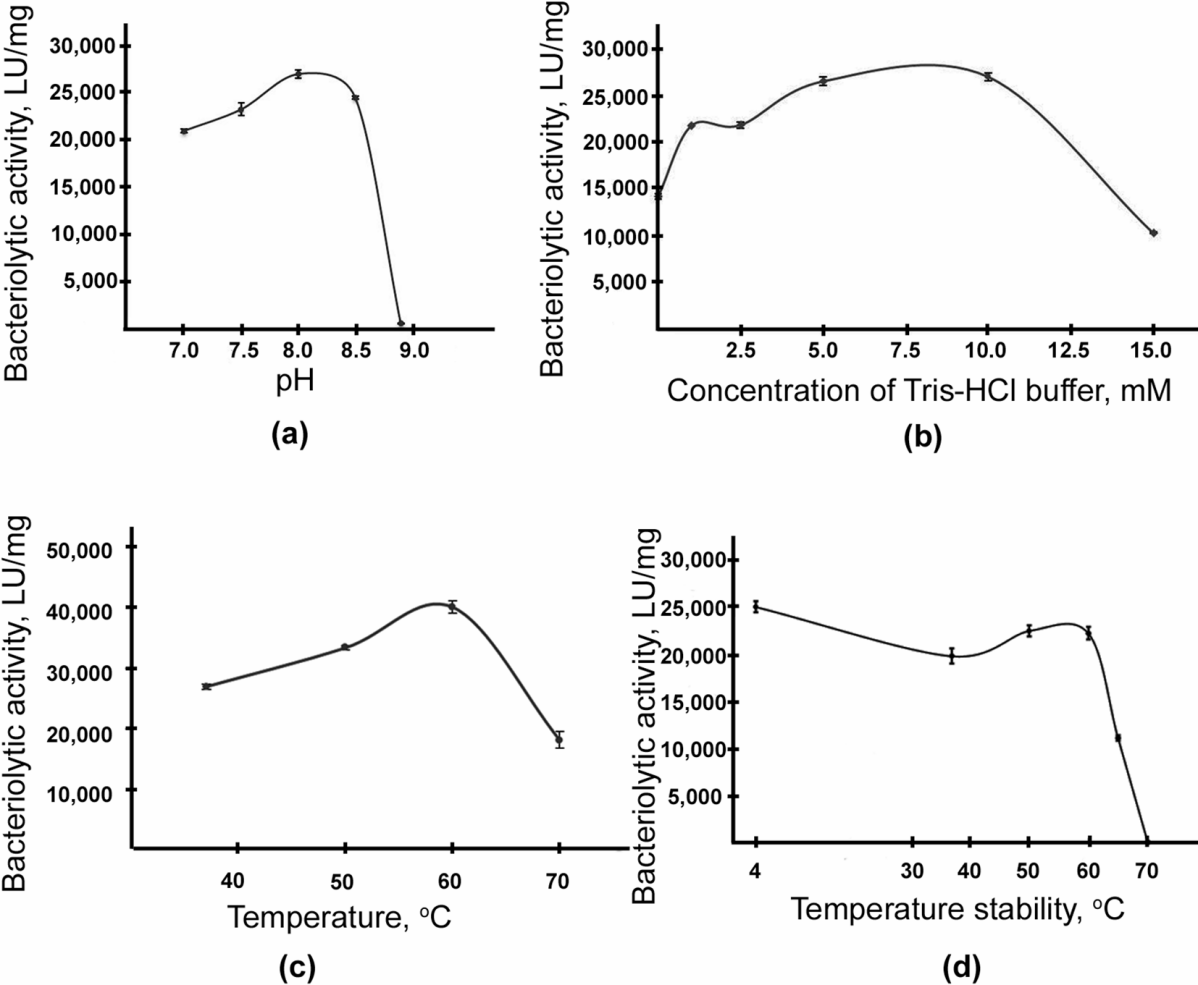
**(a–c)** Optimal conditions for hydrolysis of autoclaved *M. luteus* AC-2230^T^ cells by Ami; **(d)** Half-inactivation temperature of Ami.

The optimal conditions for the bacteriolytic activity of Ami were determined using autoclaved cells of *M. luteus* AC-2230^T^ as a substrate. The optimal conditions were found to be a Tris-HCl buffer concentration of 5–10 mM, pH 8.0, and a reaction temperature of 60 °C (Fig. 5a–c). The half-inactivation temperature of the enzyme was 65 °C (Fig. 5d).

Inhibitor analysis showed that the enzyme was completely inhibited by 1,10-phenanthroline at a concentration of 0.5 mM and *p*-chloromercuribenzoic acid (*p*-CMB) at a concentration of 2.5 mM (Table 1). The new enzyme is a metalloprotease, and sulfhydryl groups are important for its bacteriolytic activity to be revealed.

**Table 1:** Effect of inhibitors on the bacteriolytic activity of Ami.

Inhibitor	Concentration, mM	Inhibition, %
*p*-CMB	1.0	45
	2.5	100
1,10-Phenanthroline	0.1	51
	0.5	100

The Ami enzyme showed no activity against various protein substrates (casein, hemoglobin, azofibrin, collagen, elastin).

### Ami antimicrobial activity

The antimicrobial activity of Ami was studied using a turbidimetric method against various live bacterial test cultures (Table 2).

**Table 2:** Bacteriolytic activity of Ami against live test cultures. The data in the table represent the means ± standard deviations.

Test culture	Bacteriolytic activity, LU/mg
Live cells	
Opportunistic bacteria	
* Micrococcus luteus* Ac-2230^T^	815 ± 16
* Bacillus cereus* 217	2,148 ± 102
* Staphylococcus aureus* 209P	18 ± 2
* Enterococcus faecium* FS86	5.0 ± 0.5
Phytopathogenic bacteria	
* Bacillus megaterium* MS941	7,545 ± 120
* Curtobacterium flaccumfaciens* pv. *flaccumfaciens*	48 ± 4

As shown in Table 2, the Ami enzyme exhibited activity against all the selected live test organisms. The highest bacteriolytic activity was observed against the cells of *B. cereus* and *B. megaterium* MS941. This indicated a specificity of Ami towards bacteria of the genus *Bacillus*.

**Fig. 6:**
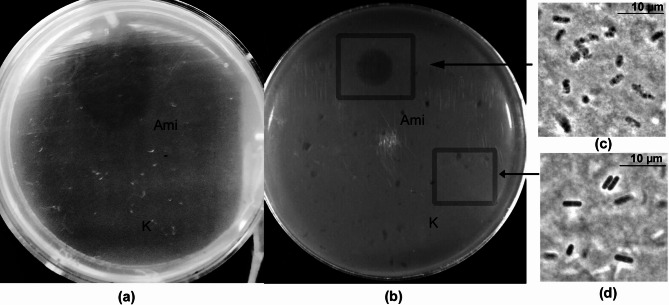
Antimicrobial activity of Ami against *B. anthracis* 71/12 **(a)** and *B. cereus* 217 **(b)** embedded in agarose gel. Phase-contrast microscopy of an agarose fragment treated with Ami **(c)** and with 10 mM Tris-HCl, pH 8.0 **(d).** Squares indicate sampling zones for phase-contrast microscopy.

The antimicrobial activity of Ami against live pathogenic bacteria of the genus *Bacillus*, including strains of *B. anthracis* harboring virulence and immunogenic plasmids pXO1 and pXO2, as well as *B. cereus* strains with hemolytic, lecithinase, and phosphatase activities, was studied by the spot-test method. The results showed that Ami lysed all the tested strains (Fig. 6a, b; Table 3). Among these, *B. anthracis* strains, including capsule-forming ones, were lysed more rapidly by Ami as compared to *B. cereus* strains.

**Table 3:** Antimicrobial activity of Ami against live pathogenic test cultures of the genus *Bacillus*.

Test culture	Antimicrobial action
*Bacillus anthracis* STI Rif 4	++
*Bacillus anthracis* 71/12	++
*Bacillus anthracis* ΔAmes	++
*Bacillus anthracis* STI	++
*Bacillus cereus* var. *anthracoides* 9	+
*Bacillus cereus* var. *anthracoides* 217	+
*Bacillus cereus* 771	+
*Bacillus cereus* АТСС9634	+
*Bacillus cereus* 504	+
*Bacillus cereus* АТСС 10,702	+

++ Lysis zone formed in 2 h after application of Ami.

+ Lysis zone formed in 6 h after application of Ami.

Microscopic analysis of an agarose fragment at the site of enzyme application showed lysed *B. cereus* 217 cells: some cells showed localized ruptures in their envelopes, others lost their characteristic rod-like shape with remnants of cytoplasmic content appearing as rare dense granules; some cells were completely destroyed, forming conglomerates of envelopes’ debris (Fig. 6c). In a control agarose gel fragment, *B. cereus* 217 cells retained their characteristic rod-like shape, were uniform in size, and displayed dense cytoplasmic content (Fig. 6d).

### Specificity of Ami action

The specificity of Ami action was studied against peptidoglycans of different chemotypes: A1γ from *B. cereus* 217; A3α, from *S. aureus* 209P; A4α, from *E. faecium* FS86. Turbidimetric analysis showed that Ami hydrolyzed the peptidoglycan *of B. cereus* 217 by 98% within 50 min (Supplementary file Fig. S5a). The peptidoglycan of *S. aureus* 209P was hydrolyzed by 81% over 6 h (Supplementary file Fig. S5b). The peptidoglycan of *E. faecium* FS86 was hydrolyzed by 95% in 1.5 h (Supplementary file Fig. S5c). The rapid and nearly complete hydrolysis of the peptidoglycans of *E. faecium* FS86 and *S. aureus* 209P was unexpected given that Ami hydrolyzed live cells of these bacteria only weakly (Table 2).

**Fig. 7:**
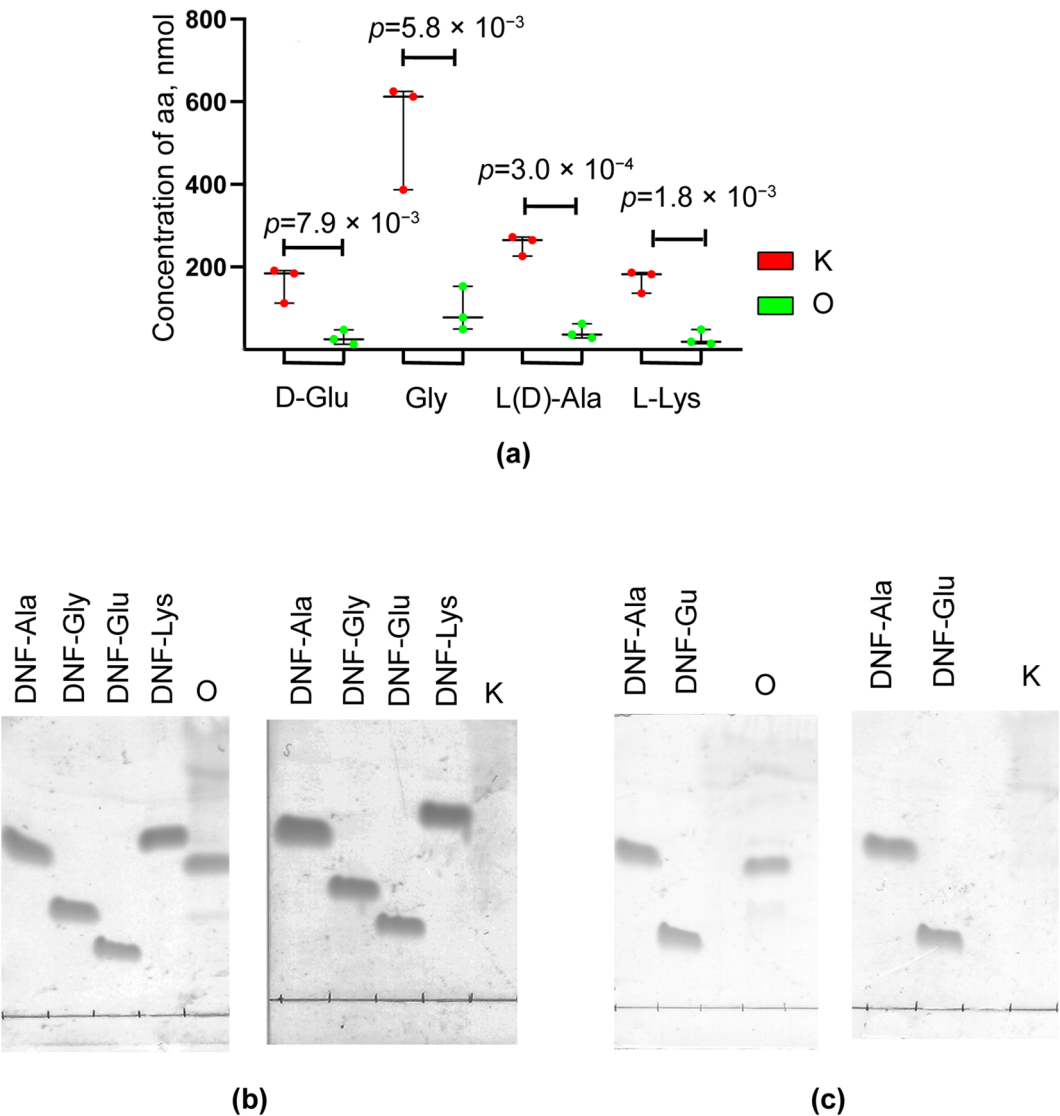
Specificity of action of bacteriolytic enzyme Ami. **(a)** Amino acid analysis of *S. aureus* 209P peptidoglycan hydrolysate; K, peptidoglycan; O, hydrolysate (debris); statistical analysis was performed using an unpaired two-tailed Student’s *t*-test: D-Glu (*t* = 4.925, *df* = 4), Gly (*t* = 5.375, *df* = 4), L-Ala (*t* = 12.500, *df* = 4), L-Lys (*t* = 7.346, *df* = 4). TLC of DNF labeled extracts of supernatant amino acids from control samples (K) and peptidoglycan hydrolysate (O) of *S. aureus* 209P **(b)** and *B. cereus* 217 **(c)**.

To determine the types of peptide bonds hydrolyzed by Ami in peptidoglycans, we performed amino acid analysis of the produced hydrolysates. First, they were centrifuged to separate debris and supernatant. The debris represented insoluble peptidoglycan fragments after enzymatic hydrolysis, while the supernatant contained unprecipitated fragments released during hydrolysis. Both fractions were treated with 2,4-dinitrofluorobenzene (DNFB) to label free NH_2_ groups of amino acids, and the resulting samples were analyzed according to the experimental scheme (Supplementary file Fig. S6). Using high-performance liquid chromatography (HPLC), amino acids not bound to DNFB were identified in the debris fraction. It was found that, after hydrolysis, no amino acids remained in the peptidoglycan of *B. cereus* 217 (Supplementary file Table S3). In the peptidoglycan of *S. aureus* 209P, the amounts of L(D)-Ala, D-Glu, Gly and L-Lys significantly decreased (down to 42 nmol, 28 nmol, 94 nmol and 27 nmol, respectively), and 212 nmol (83%), 135 nmol (84%), 447 nmol (83%) and 141 nmol (84%), respectively, was released. In the control samples, all amino acids characteristic of these peptidoglycan chemotypes were present (Supplementary file Table S3, Fig. 7a). That is, this analysis showed that practically all peptide fragment of peptidoglycan could either be extracted as DNF derivatives in the debris fraction or transferred to the supernatant. Thus, the HPLC method did not make it possible to establish which peptide bonds exactly are hydrolyzed in peptidoglycans, so we did not use it for these purposes. At the next stage, we performed TLC of DNF derivatives of amino acids extracted from the supernatant. Extracts from the supernatant of *S. aureus* 209P and *B. cereus* 217 were revealed to have DNF derivatives of alanine (Fig. 7b, c). This indicates that NH_2_ groups of L-Ala are released as a result of enzymatic hydrolysis. In control samples, no DNF derivatives of amino acids were detected (Fig. 7b, c). Similar results were obtained during the hydrolysis of peptidoglycans from *E. faecium* FS86 (Supplementary file Fig. S7).

**Fig. 8:**
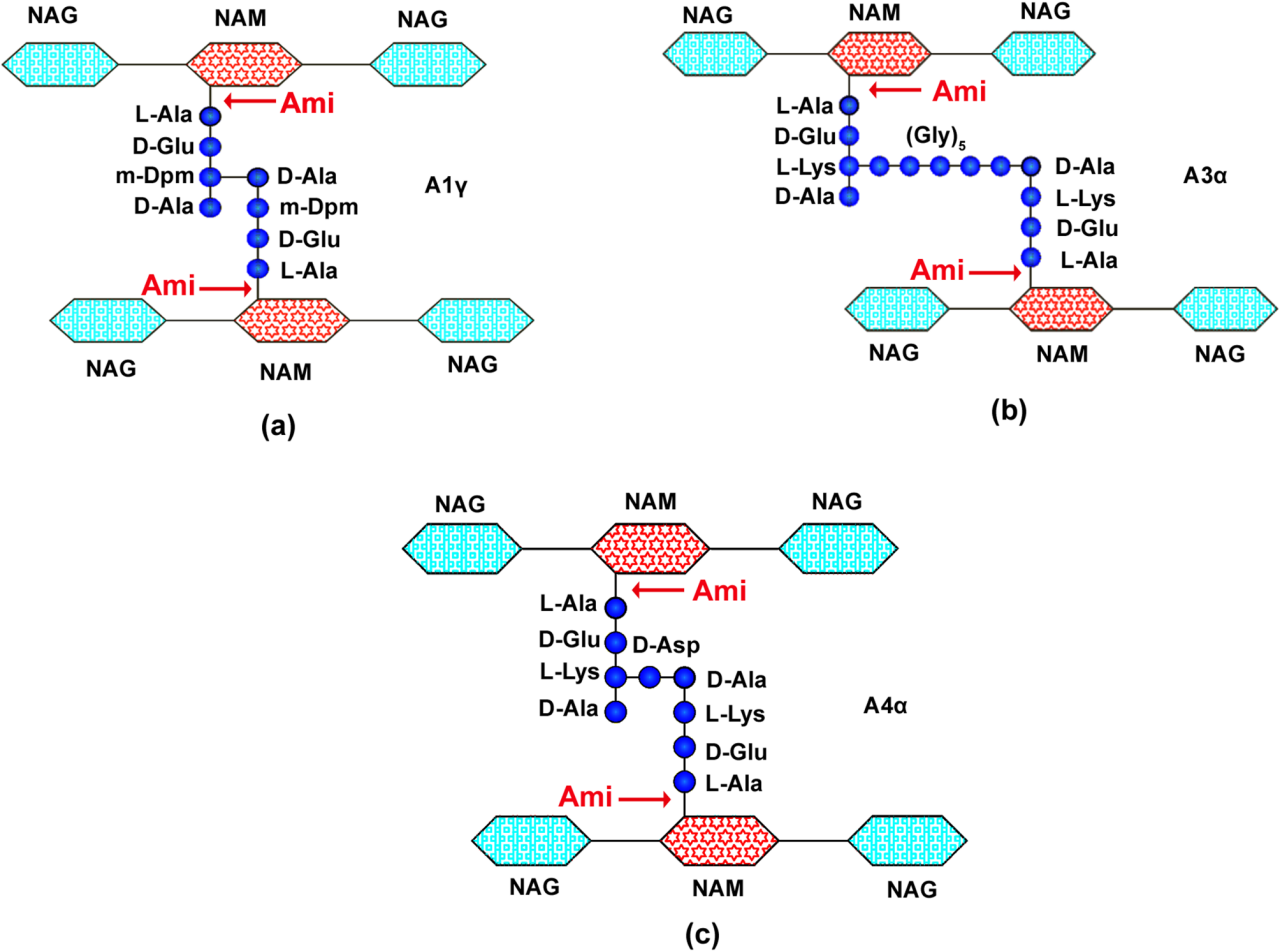
Schematic structure of peptidoglycans from **(a)** *B. cereus* 217, **(b)** *S. aureus* 209P, **(c)** *E. faecium* FS86. Arrows indicate the amide bond hydrolyzed by Ami. NAM, N-acetylmuramic acid; NAG, N-acetylglucosamine.

Thus, the bacteriolytic enzyme Ami hydrolyzed the amide bond between N-acetylmuramic acid and L-Ala in all investigated peptidoglycans (Fig. 8). This confirms the bioinformatic annotation of the enzyme as an N-acetylmuramoyl-L-alanine amidase. Notably, Ami does not hydrolyze the synthetic peptide N-acetylmuramoyl-L(Ala)-D-Glu, which indicates its specificity with respect to the amide bond of higher molecular weight substrates. Ami also does not hydrolyze the peptide bond between terminal alanine in the peptide stem, as evidenced by the absence of free alanines in the hydrolysate.

## Discussion

In this study, using proteomic and transcriptomic approaches, along with the bacteriolytic enzymes of *L. capsici* XL1 already known to us, we identified genes of the enzymes with potential bacteriolytic activity. We focused on the gene of the enzyme WND81398.1 (locus tag RJ610_03180), annotated as N-acetylmuramoyl-L-alanine amidase (Ami). In strain XL1 the expression level of this gene was 323.83 times higher, and the production level of the protein 284.05 times higher as compared to strain XL2. Phylogenetic analysis showed that genes identical to *ami* were present only in lytically active species of *Lysobacter* as well as in five species of the genera *Luteimonas*, *Denitratimonas tolerans*, and *Xanthomonas chitinolytica*, for which no lytic properties have been described. All these genera belong to the family *Xanthomonadaceae*, previously known as *Lysobacteraceae*. It is known from the literature that only the amidase CwhA (P81717), from *L. enzymogenes* M497-1, has been previously isolated and partially characterized^13^. Comparison of the amino acid sequences of the mature CwhA and Ami proteins showed their 95% identity with merely 65% coverage. The mature part of Ami consists of 270 amino acid residues, which corresponds to a molecular weight of 30 kDa of the enzyme. According to the data by Li et al., the mature part of CwhA consists of 177 amino acids with a molecular weight of 19.4 kDa. The full genomic sequence of *L. enzymogenes* M497-1 (NCBI database accession number AP014940)^27^ contains a complete gene encoding an amidase (WP_096376559.1). The amino acid sequence of CwhA differs from that of WP_096376559.1 in the segment corresponding to the C-terminal fragment of 93 amino acids. This fragment is absent in CwhA. At the same time, the amino acid sequence of amidase WP_096376559.1 is identical (by 85.56%, with 100% coverage) to Ami of *L. capsici* XL1 (WND81398.1). It is probable that an error may have occurred in Li et al. during the isolation of CwhA. This enables us to consider the identified bacteriolytic enzyme Ami as a previously uncharacterized bacteriolytic enzyme. We should note here that information about extracellular bacteriolytic amidases is extremely scarce. Previously, our colleagues^28^ have isolated and partially characterized the amidase L2 from *L. capsici* XL1. Apart from an amidase activity, the enzyme L2 also possesses a protease activity on casein and digests synthetic peptide Abz-Ala-Ala-Phe-pNa but is not active with respect to live bacterial cell targets. For this enzyme, the N-terminal amino acid has been determined (UniProt P85143). However, genome analysis we have recently conducted reveals that the N-terminal sequence of L2 matches that of the N-acetylglucosaminidase (Nag) from *L. capsici*. Additionally, no data exist on the antimicrobial activity of the extracellular amidase CwhA.

To isolate the new enzyme Ami, we developed a homologous expression system and a purification scheme. As a result, the enzyme was isolated in a homogeneous form and partially characterized. Biochemical characterization showed Ami to exhibit a maximum bacteriolytic activity at pH 8.0, in a 5–10 mM Tris-HCl buffer, at a reaction temperature of 60 °C. Ami does not hydrolyze substrates for proteases or synthetic peptide Abz-Ala-Ala-Phe-pNa. Inhibitor analysis showed that Ami was completely inhibited by 1,10-phenanthroline and *p*-CMB, which indicates the importance of metal ions and thiol groups for its activity. To date, the structure of known extracellular bacteriolytic amidases has not been solved and there is no information about the mechanism of enzymatic hydrolysis. For autolysins, endolysins, and a number of extracellular bacteriolytic proteases of Gram-positive bacteria (lysostaphin, zoocin, enterolysin), a modular structure with a catalytic domain and a substrate-binding domain has been shown^8,29–31^. The known extracellular bacteriolytic enzymes of Gram-negative bacteria have a single-domain structure, i.e., there is only a catalytic domain. However, in pseudoalterin of the Gram-negative bacterium *Pseudoalteromonas* sp. CF6-2 a structural region has been found, which, as it is suggested, may be responsible for the interaction of the enzyme with the carbohydrate part of peptidoglycan^6^.

Our close attention was focused on the antimicrobial properties of the new enzyme. We found out that Ami lysed live cells of opportunistic bacteria *S. aureus* 209P, *M. luteus* Ac-2230^T^, *E. faecium* FS86, pathogenic bacteria of various *B. cereus*, *B. anthracis* strains and phytopathogenic bacteria *B. megaterium* MS941 and *C. flaccumfaciens* pv. *flaccumfaciens*. Antimicrobial activity has been shown for the amidases of bacteriophage endolysins and bacterial autolysins; herewith, it features a narrow specificity^32^. Thus, the LytA amidase of *S. pneumonia*^33^, Pal amidase of bacteriophage Dp-1^34^, and chimeric protein PL3 (Pal catalytic domain and LytA substrate-binding domain)^35^ have lytic activities against various serotypes of *S. pneumoniae* and some *Streptococcus* species (*S. pseudopneumoniae*, *S. mitis*, *S. oralis*). The autolytic amidase AmiBA2446 of *Bacillus anthracis* Ames has a lytic activity against strains of *B. cereus* and *B. anthracis*^36^.

Bacteriolytic enzymes with amidase activities in bacterial peptidoglycan hydrolyze the bond between N-acetylmuramic acid (NAM) and L-Ala, the first amino acid of the peptide backbone. To determine the specificity of action of the new amidase, we used peptidoglycans of *B. cereus* 217, *S. aureus* 209P and *E. faecium* FS86. According to the classification of Schleifer and Kandler, peptidoglycans of *B. cereus* 217, *S. aureus* 209P and *E. faecium* FS86 belong to chemotypes A1γ, A3α and A4α, respectively^37^. The main difference is that the peptide stems in peptidoglycan of *B. cereus* 217 are directly linked; in *S. aureus* 209, via an interpeptide bridge of five glycines; in *E. faecium* FS86, via aspartate (Fig. 8). We found that in all peptidoglycans Ami hydrolyzed the amide bond between NAM and L-Ala, which is characteristic of amidases. Herewith, the synthetic fragment NAM-L-Ala-D-Glu of peptidoglycan was not hydrolyzed. This can indicate the importance of a specific structural fragment of peptidoglycan, required for enzyme binding and further hydrolysis. The issues of studying the mechanism of interaction/binding of extracellular bacteriolytic enzymes with target cells are significant at the moment. For more investigated autolysins, in particular for AmiA (from AtlA) of *S. aureus* SA113, it has been shown that this enzyme is able to hydrolyze peptidoglycan of only one chemotype, *S. aureus* SA113, but not *B. subtilis* ATCC 6051^38^. The authors suggested this to be due to a more rigid conformation of *B. subtilis* peptidoglycan, so it is difficult for the enzyme to “adjust” to it. In contrast, the bacteriolytic amidase Ami of *L. capsici* XL1 we isolated hydrolyzes the amide bond in peptidoglycans of different chemotypes A1γ, A3α and A4α. This indicates the ability of the Ami enzyme to “adjust” to the substrate. The interaction of bacteriolytic enzymes with peptidoglycan and the mechanism of hydrolysis of this substrate could be studied using structural studies and molecular biological approaches.

Thus, the bacteriolytic amidase Ami of *L. capsici* XL1 is a new promising antimicrobial agent with a broader range of antimicrobial action as compared to known bacterial and bacteriophage amidases and requires further careful study. Based on Ami, it is possible to develop novel treatments, especially against antibiotic-resistant pathogens.

## Methods

### Cultivation conditions

Cells of *Lysobacter* strains were cultivated in a modified LB medium (LB-M) containing (g/L): peptone, 5.0; yeast extract, 5.0; NaCl, 5.0, pH 7.5; and RM medium containing (g/L): glucose, 5.0; peptone, 2.0; yeast extract, 2.0; Na_2_HPO_4_ × 12H_2_O, 4.2; KH_2_PO_4_, 1.0; KCl, 0.6; MgSO_4_ × 7H_2_O, 5.0, pH 7.0^20^. Cultivation was performed at 29 °C for 21 h with shaking at 205 rpm.

Target cells of *B. cereus* 217, *B. megaterium* MS941, *M. luteus* AC-2230^T^, *K. rosea* Ac-2200^T^, *S. aureus* 209P, *E. faecium* FS86 were cultivated on a 5/5 medium developed at IBPM RAS, containing (g/L): yeast extract, 1.0; soybean extract, 30.0; tryptone, 5.0; aminopeptide, 60.0; agar, 15.0, pH 7.2, at 29 °C for 18 h. Cells of *C. flaccumfaciens* pv. *flaccumfaciens* and all pathogenic strains of *B. anthracis* and *B. cereus* were cultivated on an LB medium at 29 °C for 14–21 h.

To obtain peptidoglycans, cells of *B. cereus* 217 and *S. aureus* 209P were cultivated on a BHI medium (Difco, Detroit, MI, USA) at 29 °C and 37 °C, respectively, for 24 h with shaking at 205 rpm. Cells of *E. faecium* FS86 were cultivated on an MRS medium (g/L): nutrient broth (Himedia, Maharashtra, India), 16.0; yeast extract, 4.0; glucose, 20.0; sorbitan mono oleate, 1.0; K_2_HPO_4_, 2.0; Na-acetate, 5.0; diammonium hydrogen citrate, 2.0; MgSO_4_ × 7H_2_O, 0.2; MnSO_4_×H_2_O, 0.04, pH 5.7^39^ at 29 °C for 18 h with shaking at 205 rpm.

The respective dense nutrient media contained 1.5% agar.

### RNA-seq RNA extraction

Cells of *L. capsici* XL1 and XL2 were cultivated in 150 mL of an RM medium in three biological replicates. Total RNA was extracted from equal amounts of cells using the RiboPure RNA Purification Kit (Thermo Scientific, Waltham, MA, USA), following the manufacturer’s instructions. The kits used to obtain libraries are described in Afoshin et al.^17^. A library was sequenced on the Illumina HiSeq 4,000 system (Illumina, San Diego, CA, USA) to obtain 151 bp reads.

The quality of reads was controlled using FastQC v0.12.1 (http://www.bioinformatics.babraham.ac.uk/projects/fastqc). Adapter sequences and low quality regions in raw reads were removed using Trimomatic v0.39^40^. The clean reads of both *L. capsici* XL1 and XL2 were mapped to the genome of strain XL1 (GenBank access No. CP134270.1) using the Bowtie2 v2.5.1^41^; the mapped reads were counted using the featureCounts v2.0.4^42^. The DESeq2 v1.34.0 package was used to assess differential gene expression^43^. The read count for *L. capsici* XL2 was used as a control. A gene was assumed to change the expression level if the adjusted *p*-value (*p*_adj_) < 0.05.

### LC−MS/MS analysis

Cells of *L. capsici* XL1 and XL2 were cultivated as described above. Equal volumes of culture were centrifuged at 7,000× *g* for 20 min to discard cells. Proteins in the culture fluid were precipitated with 10% trichloroacetic acid at 4 °C for 3 h and centrifuged at 25,000× *g* for 10 min. The residue was washed thrice with cold acetone at the same speed. At the last stage, the residue was suspended in a 50 mM Tris-HCl buffer, pH 8.0, containing 5% SDS. Protein concentration was determined using the BSA method. Proteins (50 µg per sample) were digested using the S-Trap^44^ method with trypsin (Promega, Madison, WI, USA) at a 50:1 ratio at 37 °C for 18 h. For LC–MS/MS analysis, we used an Ultimate 3,000 RSLCnano system (Thermo Scientific, Waltham, MA, USA) coupled to a Q-Exactive HF-X mass spectrometer (Thermo Scientific, Waltham, MA, USA). Peptides were separated on an Acclaim PepMap C18 column (Thermo Scientific, Waltham, MA, USA) in a gradient of 0.1% formic acid in water to 0.1% formic acid in 80% acetonitrile at a column temperature of 55 °C for 90 min at 0.3 µL/min.

Raw data were converted to MGF format using MSConvert (ProteoWizard 3.0) and analyzed with MaxQuant v2.0.1.0 (the Andromeda search engine) against the *L. capsici* XL1 genome (GenBank access No. CP134270.1). The parameters of protein identification by the LC–MS/MS data are similar to Borvinskaya et al.^45^. Proteins were considered to be identified with at least two peptides using HUPO guidelines. Statistical analysis was performed using Perseus v1.6.15.0.

### Generation of *L. capsici* P_GroEL(A)_-*ami* expression strain

All molecular genetic procedures were performed in accordance with manufacturers’ recommendations and Sambrook and Russell’s manual^46^. Use was made of restriction endonucleases, alkaline phosphatase, T4 DNA ligase, T4 polynucleotide kinase (Thermo Fisher Scientific, Waltham, MA, USA). PCR was performed with Q5 DNA polymerase (New England Biolabs, Ipswich, MA, USA). DNA was visualized in gel at 354 nm. A QIAquick Gel Extraction kit (Qiagen, Germantown, MD, USA) and a diaGene kit (Diaem, Moscow, Russia) were used to isolate DNA from agarose gel and from bacterial cells, respectively.

The expression vector pBBR1-MCS5 P_GroEL(A)_–*ami* was constructed by amplifying by the PCR method a 1,623-bp DNA fragment from *L. capsici* XL1 containing the *ami* gene with primers AMI_*HindIII* (forward) (AAGCTTATGCATCTTCACTCTCTCGCCG) and AMI_*BamHI* (reverse) (GGATCCTCAGCAGTCCGGGATCACC). The amplified fragment was cloned into the plasmid vector pBBR1-MCS5 P_GroEL(A)_–*gfp* (the plasmid was constructed in^47^) at the *BamHI* and *HindIII* sites. The resulting plasmid in the amount of 1 µg was electroporated into *L. capsici* XL1 cells under previously optimized conditions^48^. The expression strain *L. capsici* P_GroEL(A)_–*ami* was validated using oligonucleotide primers Gro_*KpnI* (forward) (GGTACCCGGACCGACGCCTGTCA) and Term_*XbaI* (reverse) (TCTAGAAGAGTTTGTAGAAACGCAAAAAGGC) and by sequencing at Evrogen (Moscow, Russia).

### Purification of bacteriolytic enzyme Ami

Cells of the expression strain *L. capsici* P_GroEL(A)_–*ami* were cultivated in an LB-M medium. A 2-L culture was centrifuged at 7,000× *g* for 30 min. Proteins from the culture fluid were precipitated with 80% saturated (NH_4_)_2_SO_4_ at 4 °C for 16 h and centrifuged at 22,470× *g* for 1 h. The residue was suspended in a 30 mM sodium acetate buffer, pH 5.5, and dialyzed against 100 V of the same buffer for 16 h. Then the purification scheme was developed. The first step of purification involved cation exchange chromatography on a Toyopearl CM-650 column (Merck, Darmstadt, Germany) equilibrated with a 30 mM sodium acetate buffer, pH 5.5. Proteins were eluted with the same 30 mM buffer containing 0.3 M NaCl. Eluted fractions were pooled, dialyzed again against a 30 mM sodium acetate buffer, pH 5.5, and subjected to further purification on an ENrichS column (Bio-Rad, Hercules, CA, USA) coupled with an NGC chromatographic system (Bio-Rad, Hercules, CA, USA) equilibrated with the same buffer. Proteins were eluted with a linear gradient of NaCl, 0.03–0.30 M. Electrophoretically homogeneous fractions were combined for the further research. The purified amidase was validated by MALDI-TOF analysis.

### Determination of protein concentration

Protein concentration was measured using the Bradford method^49^ with Coomassie reagent (Thermo Fisher Scientific, Waltham, MA, USA) or a BSA kit (FineTest, Wuhan, China). The reaction was carried out according to the manufacturers’ protocols. Calibration curves were generated using bovine serum albumin solutions (Sigma, Ronkonkoma, NY, USA) in the range of 1–25 µg/mL or 0.25–2.00 mg/mL.

### Phylogenetic analysis

Comparative phylogenetic analysis was performed using MEGA 11.0.13 software^50^, employing the maximum likelihood method with the Jones–Taylor–Thornton amino acid substitution model^51^. The bootstrap test was performed with 1,000 iterations^52^.

### Determination of bacteriolytic activity using a turbidimetric method

For determining bacteriolytic activity, autoclaved lyophilized cells of *S. aureus* 209P or *M. luteus* AC-2230^T^ were used as substrates. The reaction mixture contained 1 mL of cell suspension and Ami enzyme (0.05 µg)/culture fluid. The mixture was incubated at 37 °C for 5 min.

The bacteriolytic activity of Ami was also tested against live cells of opportunistic bacteria (*B. cereus* 217, *M. luteus* AC-2230^T^, *S. aureus* 209P, *E. faecium* FS86) and phytopathogenic bacteria (*B. megaterium* MS941, *C. flaccumfaciens* pv. *flaccumfaciens*). Target cells were suspended in 10 mM Tris-HCl, pH 8.0 (OD_540_ = 0.5), and 0.18–1.4 µg of Ami was added. As a control, a suspension of target cells not treated with the enzyme was used. The reaction was conducted at 37 °C for 20 min, 60 min, 2.5 h, 8 h, and 15 h, depending on the target cells.

The reaction was stopped by placing tubes in an ice bath. The decrease in optical density of the suspension was measured at 540 nm. Bacteriolytic activity (LU/mL) was calculated using the formula:

[0.5 (OD_540_ of the control suspension) – OD_540 _of the experimental suspension] × 1000 × L (total reaction volume) × dilution/[min (time of reaction) × L (volume of sample) × 0.01 (correction coefficient for the OD reduction per min)].

Here and further, all biochemical reactions were carried out in three repeats.

### Determination of optimal conditions for Ami bacteriolytic activity

The optimal conditions for bacteriolytic activity were determined using autoclaved lyophilized cells of *M. luteus* AC-2230^T^ as the substrate. Bacteriolytic activity was measured as described above. The optimal values were determined within a range of buffer concentrations (1–15 mM Tris-HCl), pH levels (7.0–9.0), and reaction temperatures (37–70 °C). Thermal stability was assessed by incubating 5–10 µL (0.05–0.21 µg) of Ami in 10 mM Tris-HCl, pH 8.0, for 15 min at temperatures ranging from 37 to 70 °C. Then a suspension of micrococcal cells was added, the mixture was incubated for 5 min, and the residual bacteriolytic activity was measured.

### Effect of inhibitors on Ami bacteriolytic activity

The effect of inhibitors on Ami bacteriolytic activity was studied using *p*-CMB at concentrations of 1.0 and 2.5 mM, and 1,10-phenanthroline at concentrations of 0.1 and 0.5 mM. The reaction was conducted in 10 mM Tris-HCl, pH 8.0. An inhibitor was added to 10.0 µL of Ami (0.2 µg), and the volume was adjusted to 0.3 mL with Tris-HCl buffer. The mixture was incubated at room temperature for 20 min, followed by the addition of 0.3 mL of *M. luteus* AC-2230^T^ cell suspension. After a 10-min incubation at 37 °C, the residual bacteriolytic activity was measured.

### Determination of Ami protease activity on protein substrates

The protease activity of Ami was tested against substrates gelatin, azofibrin, collagen, elastin, and hemoglobin using a spot-test method. Petri dishes containing 0.5–1% substrate in 10 mM Tris-HCl, pH 8.0, and 1.5% agar were prepared. Wells were created, and 45 µL (0.95 µg) of the enzyme preparation or buffer each as a control was added. Dishes were incubated at 29 °C, until the appearance of zones of clearance.

### Determination of antimicrobial activity of Ami using the spot-test method

Pathogenic bacteria from the second pathogenicity group were used as test organisms: *B. anthracis* STI Rif 4, *B. anthracis* 71/12 (containing plasmids pXO_1_ and pXO_2_), *B. anthracis* ΔAmes (containing plasmid pXO_2_), *B. anthracis* STI (containing plasmid pXO_1_), as well as pathogenic strains *B. cereus* var. *anthracoides* 9, *B. cereus* var. *anthracoides* 217, *B. cereus* 771, *B. cereus* ATCC9634, *B. cereus* 504, *B. cereus* ATCC10702 with hemolytic, lecithinase, and phosphatase activities. Cell suspensions of target bacteria were prepared in 10 mM Tris-HCl, pH 8.0 (OD_540_ = 1.5), with 1% agarose and were spread into Petri dishes. Onto an experimental dish, 12 µL of Ami enzyme (0.6 µg) each was applied. Control samples contained 12 µL of 10 mM Tris-HCl buffer, pH 8.0. Dishes were incubated at 37 °C for 2–18 h until zones of clearance appeared.

### Phase-contrast microscopy

Fragments of 2 × 2 mm were excised from agarose gel containing *B. cereus* 217 cells, both from the lysis zone (treated with Ami) and the control zone. Microscopy was performed in phase-contrast mode using a Nikon Eclipse Ci microscope equipped with a phase objective and a ProgRes SpeedXT camera (Jenoptic, Jena, Germany).

### Isolation of peptidoglycans

Peptidoglycans were extracted using a modified Shaw method^53^. After cultivation, cells of *B. cereus* 217, *S. aureus* 209P and *E. faecium* FS86 were autoclaved at 1 atm for 1 h, centrifuged at 5,000× *g* for 20 min, and washed with 0.1 M Tris-HCl, pH 7.2. The suspension of cells in the same buffer was then frozen at − 20 °C and disrupted using a french press. The homogenate was treated with DNase I (Roche, Mannheim, Germany) and RNase A (Sigma, Ronkonkoma, NY, USA) at a final concentration of 0.02 mg/mL, followed by centrifugation under the same conditions to discard intact cells. Cell walls were precipitated by centrifugation at 22,000× *g* for 15 min at 4 °C, boiled in Milli-Q water, treated with trypsin (Sigma, Ronkonkoma, NY, USA) at a final concentration of 0.1 mg/mL in 0.1 M Tris-HCl, pH 8.0, and phospholipids were extracted with a chloroform–methanol mixture (2:1). The aqueous fraction, representing purified peptidoglycan, was lyophilized. Purity and quality were assessed using amino acid analysis by HPLC. As standards, use was made of mixtures of L-Ala, D-Glu, m-Dpm (Sigma, Ronkonkoma, NY, USA) for *B. cereus* 217; L-Ala, D-Glu, L-Lys, Gly (Sigma, Ronkonkoma, NY, USA) for *S. aureus* 209P; L-Ala, D-Glu, L-Lys, D-Asp (Sigma, Ronkonkoma, NY, USA) for *E. faecium* FS86, at concentrations of 2.5 µmol/mL for each amino acid.

### Determination of specificity of Ami action on peptidoglycans of various chemotypes

The dynamics of the hydrolysis of *B. cereus* 217, *S. aureus* 209P and *E. faecium* FS86 peptidoglycans by the bacteriolytic enzyme Ami was studied turbidimetrically. Suspensions of peptidoglycans (OD_540_ = 1.0) were prepared in 10 mM Tris-HCl, pH 8.0. Ami (2.6 µg) was added, and the reaction was incubated at 37 °C for 1–12 h, with OD_540_ measured every 5 min.

To determine the types of peptide bonds hydrolyzed by Ami in investigated peptidoglycans, the Ghuysen and Strominger dinitrophenylation method was used^54^. The scheme of the experiment is shown in Fig. S6 (Supplementary file). The hydrolysate was centrifuged at 17,000× *g* for 15 min to obtain preparations of debris and supernatant. The preparations were treated with DNFB (Sigma, Ronkonkoma, NY, USA). Control samples (suspension of peptidoglycan without Ami) were also treated with DNFB. Then acid hydrolysis with 6 N HCl at 110 °C for 15 h followed. DNF derivatives of amino acids were extracted with chloroform and applied on a 60 F254 plate (HPTLC, Merck, Darmstadt, Germany). The amino acids were separated by the TLC in a chromatographic system of chloroform: methanol: acetic acid (25:5:1). As standards, use was made of DNF derivatives of amino acids (DNF-Ala, DNF-Glu, DNF-Lys, DNF-Gly, DNF-Asp) (Serva, Heidelberg, Germany) at concentrations of 2.5 µmol/mL.

Free amino acids in the supernatant were analyzed using TLC in a butanol: acetic acid: water (4:3:1) system after a preliminary evaporation. As standards, use was made of of separate amino acids (L-Ala, D-Glu, L-Lys, Gly, m-Dpm and D-Asp (Sigma, Ronkonkoma, NY, USA) at concentrations of 2.5 µmol/mL. The chromatogram was treated with ninhydrin (0.2%) in acetone.

After extraction of DNF-derived amino acids, the precipitate was suspended in a buffer containing 0.04 M trisodium citrate dihydrate, 0.03 M citric acid, 1.4% thiodiglycol, 0.02 M phenol, pH 2.2, and applied to a column with a cation exchange resin (4.6 × 150 mm, 7 μm particle size) coupled to an amino acid analyzer. Elution was performed with a buffer containing 0.07 M trisodium citrate dihydrate, 0.08 M boric acid, pH 10.85. A mixture of amino acids L-Ala, D-Glu, L-Lys, and Gly was used as standards for the analysis of peptidoglycan samples from *S. aureus* 209P and of L-Ala, D-Glu, m-Dpm (Sigma, Ronkonkoma, NY, USA) for the analysis of *B. cereus* 217 peptidoglycan samples at a concentration of 2.5 µmol/mL. Peak areas were calculated using Clarity Chromatography Software (3.0.6.589).

A synthetic fragment of the peptidoglycan NAM-L-Ala-D-Glu (Sigma, Ronkonkoma, NY, USA) was also used to study the specificity of Ami action. The reaction mixture contained 150 µg of substrate and 0.8 µg of Ami. The mixture was incubated at 37 °C for 13 h. The hydrolysis products were analyzed by TLC in a butanol: acetic acid: water (4:3:1) chromatographic system. L-Ala-D-Glu dipeptide was used as the standard at a concentration of 2.5 µmol/mL. The chromatogram was treated with ninhydrin solution (0.2%) in acetone.

### Statistical analysis

Statistical analysis was performed using GraphPad Prism v8.0.1 (GraphPad Software, San Diego, CA, USA). All experiments were conducted in 3–6 repeats.

The data are presented as means ± standard deviations, as well as in the form of boxplots (medians ± interquartile spans). The data were considered to be significant at *p* < 0.05.

Normal distribution of data was assessed using the Shapiro–Wilk test. The *F*-test was employed to determine the equality of variances of two independent groups.

## Electronic supplementary material

Below is the link to the electronic supplementary material.

Supplementary Material 1

## Data Availability

Data is provided within the manuscript or supplementary information files.
