# Supplementary material for: A new bacteriolytic amidase Ami of Lysobacter capsici XL1
Source: Sci Rep. 2025 Jul 1;15:22257. doi: 10.1038/s41598-025-07691-2 (PMC12219114; doi:10.1038/s41598-025-07691-2)
Supplement: Supplementary file 1 — Supplementary Material 1 [file 41598_2025_7691_MOESM1_ESM.pdf]

# A new bacteriolytic amidase Ami of *Lysobacter capsici* XL1


**Irina Kudryakova[1], Alexey Afoshin[1], Sergey Tarlachkov[1], Sofya Pavlenko[1,2], Natalya Suzina[1], Nina Shishkova[3], Elena Leontyevskaya[1], Natalya Leontyevskaya[1,*]**

[1] *Laboratory of Microbial Cell Surface Biochemistry, G.K. Skryabin Institute of Biochemistry and Physiology of Microorganisms, FRC PSCBR, Russian Academy of Sciences, 5 Prosp. Nauki, 142290 Pushchino, Russia*

[2] *Lomonosov Moscow State University, Leninskiye Gory, 119899 Moscow, Russia*

[3] *FBIS State Research Center for Applied Biotechnology and Microbiology, 24 Kvartal-A Territory, 142279 Obolensk, Russia*

***Corresponding author**:

N.V. Leontyevskaya, 5 Prosp. Nauki, Pushchino, Moscow Region, 142290, Russia

E-mail address: vasilyevanv@rambler.ru


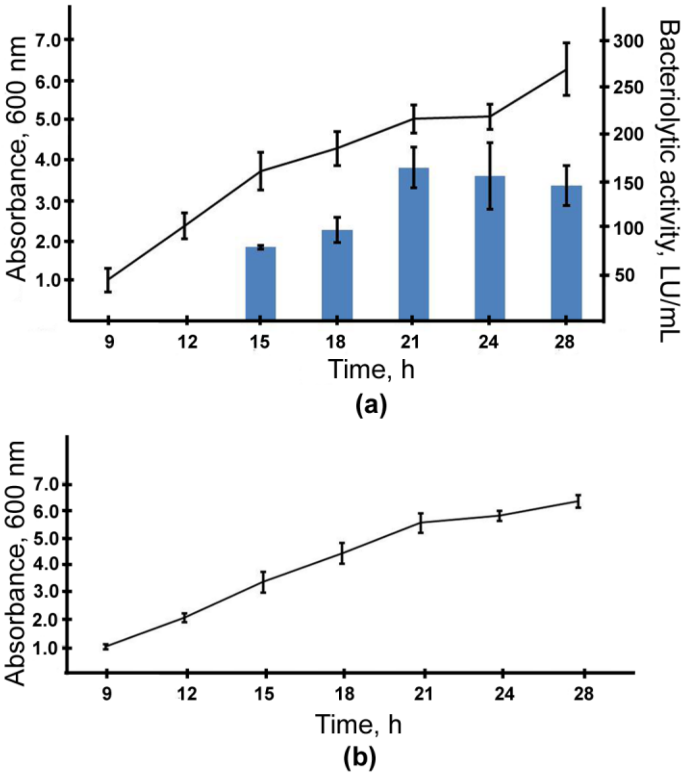


**Supplementary file Figure S1**. Dynamics of growth and bacteriolytic activity with respect to autoclaved cells of *S. aureus* 209P for *L. capsici* XL1 **(a)** and *L. capsici* XL2 **(b)**.

| Strain | Repeats | Number of raw reads | Number of clean reads | Number of mapped reads as referred to genes |
|---|---|---|---|---|
| *L. capsici* XL1 | 1 | 36,313,206 | 36,221,042 | 35,361,805 |
| | 2 | 32,728,251 | 32,651,099 | 31,551,406 |
| | 3 | 29,577,042 | 29,507,760 | 28,586,408 |
| *L. capsici* XL2 | 1 | 31,412,335 | 31,343,791 | 30,303,068 |
| | 2 | 24,442,334 | 24,379,791 | 23,714,068 |
| | 3 | 29,596,561 | 29,539,558 | 28,735,220 |

**Supplementary file Table S1.** Number of reads used to assess differential expression.

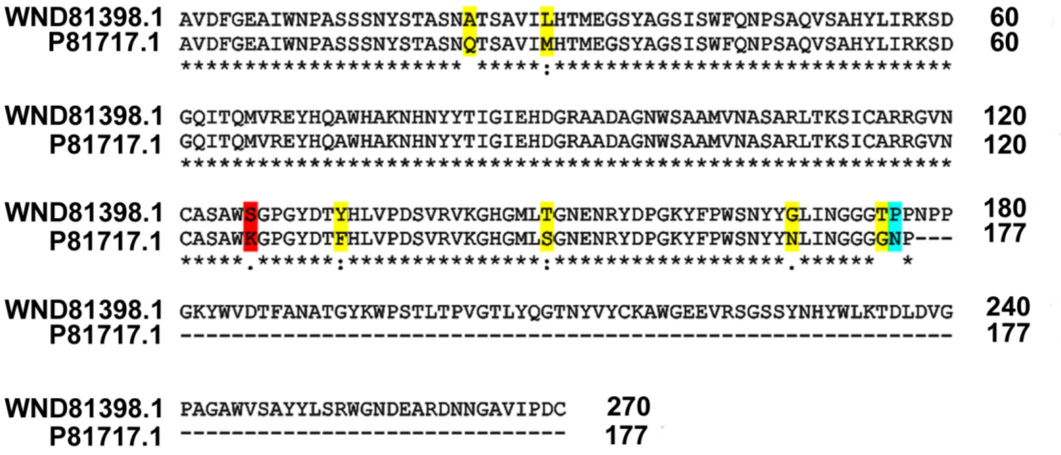


**Supplementary file Figure S2**. Comparison of amino acid sequences of Ami of *L. capsici* XL1 (WND81398.1) and amidase CwhA of *L. enzymogenes* (P81717.1). Yellow highlights, aa substitutions; red highlights, aa substitutions with change of charge; turquoise highlight, substitution with proline.

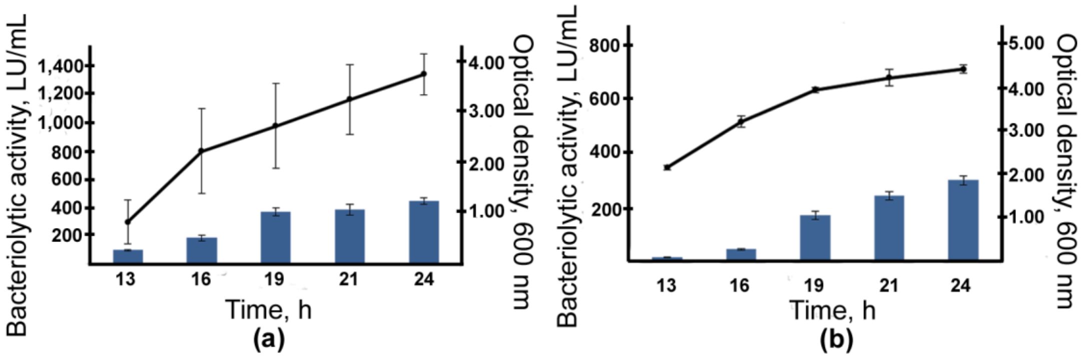


**Supplementary file Figure S3**. Dynamics of growth and bacteriolytic activity with respect to autoclaved *M. luteus* AC-2230$^{T}$ cells for *L. capsici* $P_{GroEL(A)}$–*ami* **(a)** and wild-type *L. capsici* XL1 **(b).**

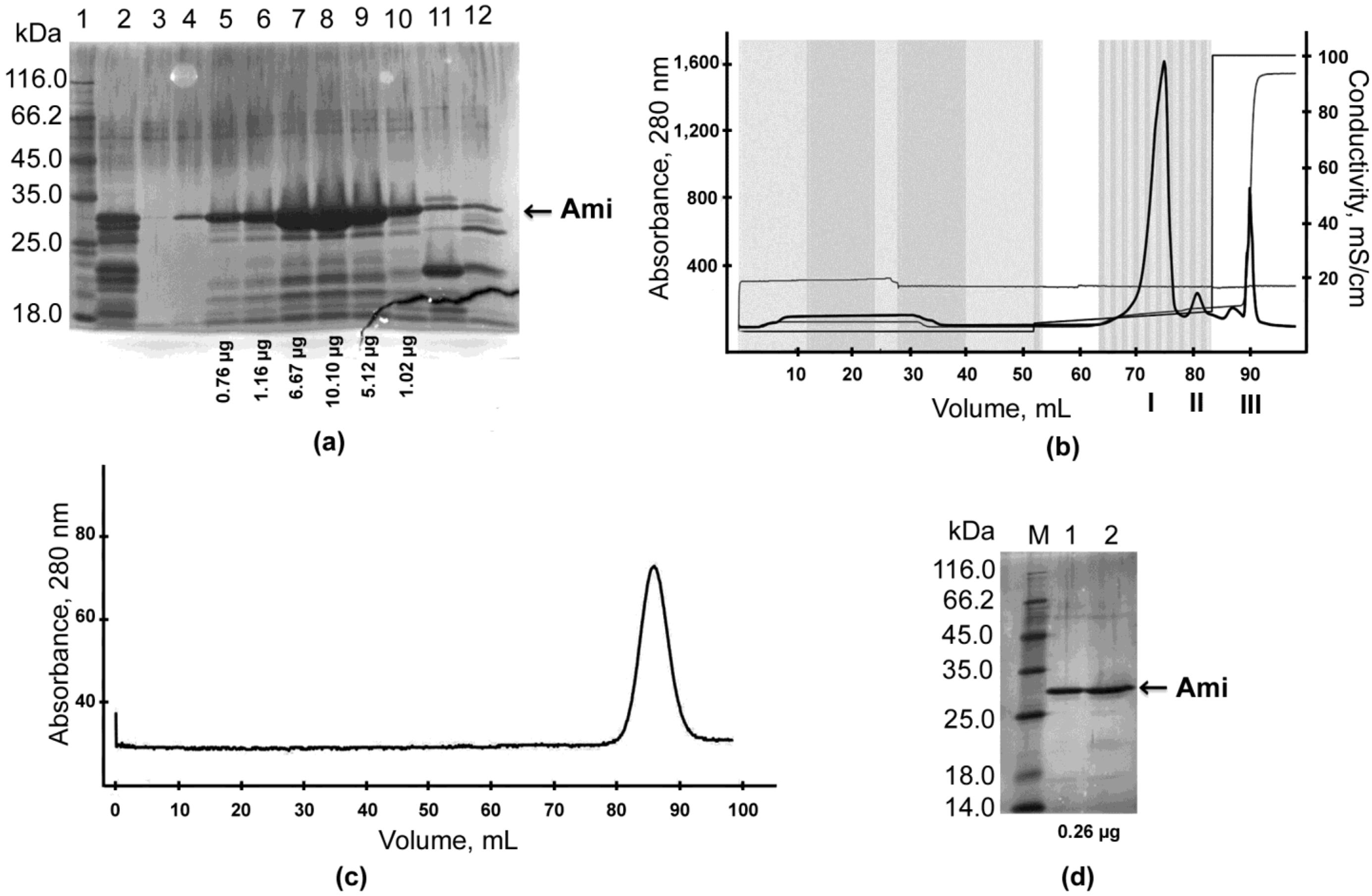


**Supplementary file Figure S4**. **(a)** Original gel images for Figure 4b. Lane 1 corresponds to M of Figure 4b; lane 2, to protein fraction after Toyopearl CM-650 of Figure 4b; lane 6, to protein fraction after ENrichS of Figure 4b. Lane 3, flow-through fraction; lane 4, washing fraction; lanes 5–10, peak I (purified bacteriolytic amidase Ami, autolysis products are observed); lane 11, peak II; lane 12, peak III. **(b)** Chromatogram of the purification of bacteriolytic amidase Ami on the ENrichS column. Peak I (lanes 5–10), purified amidase Ami. **(c)** Chromatogram of Ami purification on the HiLoad 16/60 column (Superdex 75). **(d)** SDS-PAGE. Lane 1 corresponds to Ami after purification on the ENrichS column; lane 2, to Ami after purification on the HiLoad 16/60 column.

| Step | Total protein (mg) | Total activity (U)* | Specific activity (U/mg) | Purification (fold) | Yield (%) |
|---|---|---|---|---|---|
| Culture fluid | 15.66 | 13,505 | 862 | 1.00 | 100.00 |
| CM 650 elution fractions | 1.12 | 2,664 | 2,378 | 2.76 | 19.72 |
| ENrich S | 0.21 | 472 | 2,248 | 2.60 | 3.50 |

**Supplementary file Table S2.** Ami purification table.

* Living cells of *Bacillus cereus* 217 was used as substrate for determining bacteriolytic activity.

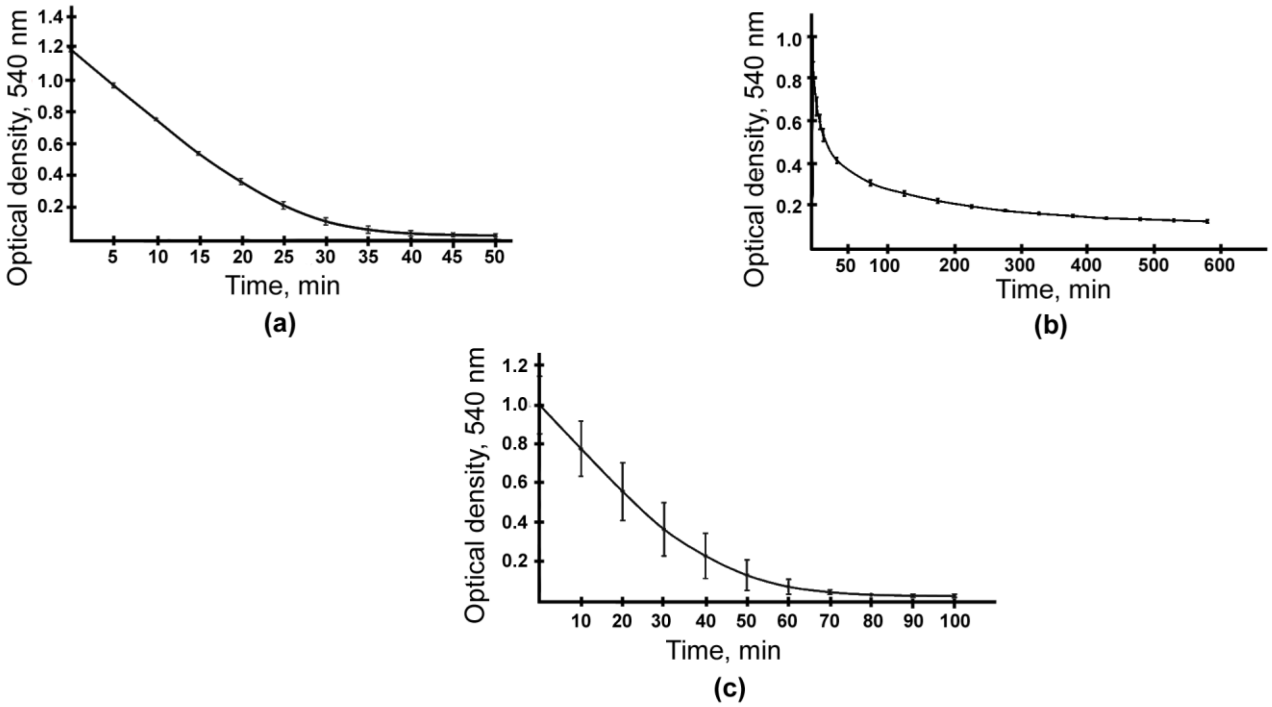


**Supplementary file Figure S5**. Dynamics of enzymatic hydrolysis of peptidoglycans from *B. cereus* 217 **(a),** *S. aureus* 209P **(b)** and *E. faecium* FS86 **(c)**.

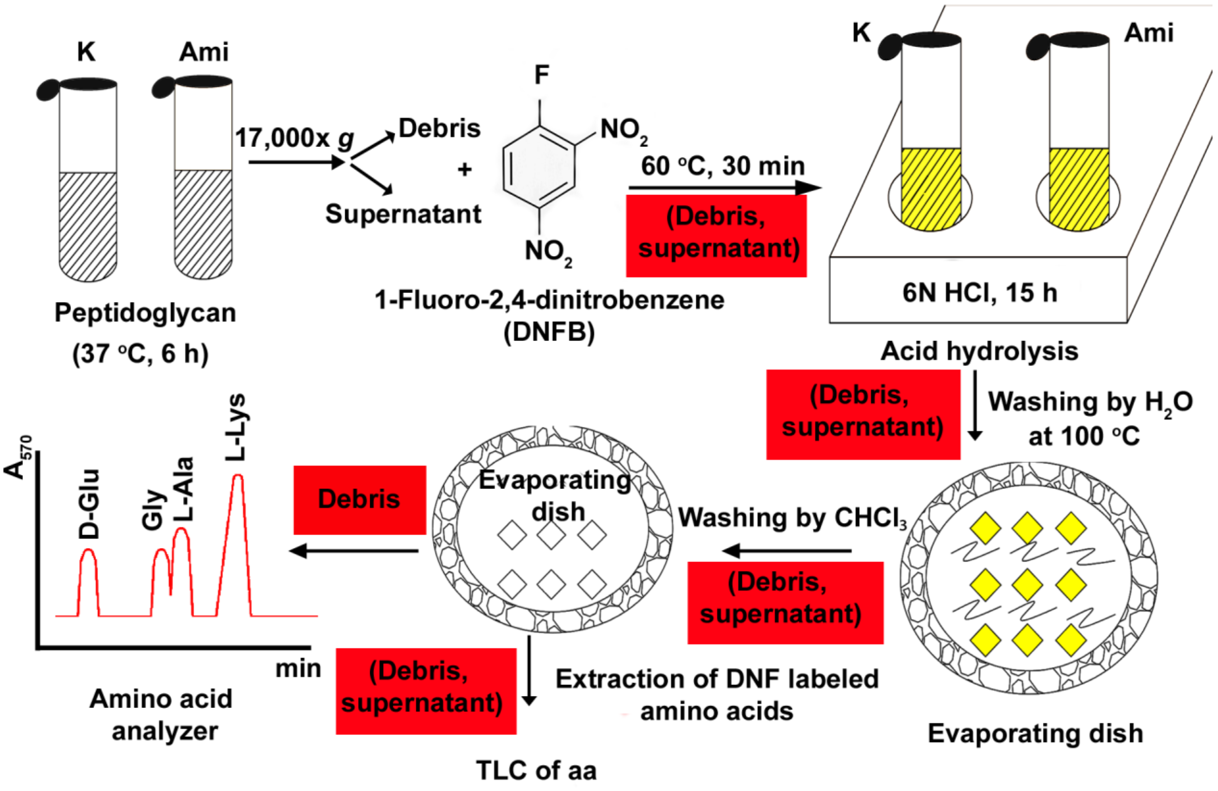


**Supplementary file Figure S6**. Scheme of an experiment on the specificity of Ami action with respect to peptidoglycans of *B. cereus* 217, *S. aureus* 209P and *E. faecium* FS86.

| **Peptidoglycan debris** | **Amino acid** | **Control sample, nmol** | **Experimental sample, nmol** |
|---|---|---|---|
| *S. aureus* 209P | D-Glu | 163±44 | 28±18 |
| | Gly | 541±134 | 94±54 |
| | L(D)-Ala | 254±24 | 42±18 |
| | L-Lys | 168±28 | 27±18 |
| *B. cereus* 217 | D-Glu | 224±32 | 0 |
| | L(D)-Ala | 431±51 | 0 |
| | m-Dpm | 168±24 | 0 |

**Supplementary file Table S3.** Amino acid analysis of the debris fraction of *B. cereus* 217 and *S. aureus* 209P peptidoglycans after extraction of DNF derivatives of amino acids.

The data in the table represent the means±s.d.

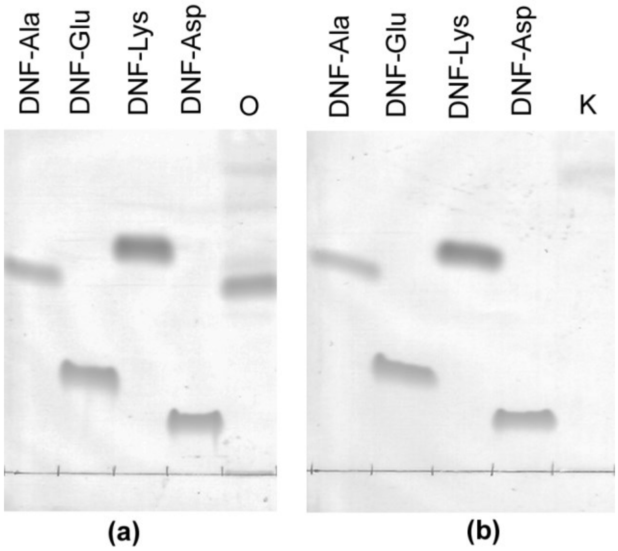


**Supplementary file Figure S7**. TLC of DNF labeled extracts of supernatant amino acids from *E. faecium* FS86 peptidoglycan: **(a)** experimental sample (O); **(b)** control sample (K).

| locus_tag | product_accession | rna_fc | rna_padj | prot_fc | prot_pvalue | product |
|---|---|---|---|---|---|---|
| RJ610_19470 | WND79457.1 | 277,26 | 1,39E-136 | 10309,37 | 8,54E-07 | LamG domain-containing protein |
| RJ610_15510 | WND78712.1 | 24,92 | 4,32E-61 | 417,74 | 5,05E-05 | pectin acetylesterase-family hydrolase |
| RJ610_03180 | WND81398.1 | 323,83 | 2,79E-273 | 284,05 | 2,77E-06 | peptidoglycan recognition family protein |
| RJ610_06330 | WND81974.1 | 132,55 | 3,59E-111 | 239,74 | 3,21E-05 | hypothetical protein |
| RJ610_14880 | WND78595.1 | 5,99 | 6,08E-51 | 229,96 | 1,04E-05 | phospholipase C, phosphocholine-specific |
| RJ610_02225 | WND81213.1 | 42,74 | 6,37E-72 | 133,93 | 1,00E+00 | TonB-dependent receptor |
| RJ610_17270 | WND79043.1 | 679,88 | 0,00E+00 | 123,66 | 4,37E-05 | glycoside hydrolase family 64 protein |
| RJ610_12335 | WND83083.1 | 107,98 | 1,33E-85 | 85,91 | 1,38E-04 | trypsin-like serine protease |
| RJ610_01060 | WND80997.1 | 82,57 | 3,36E-73 | 78,43 | 1,00E+00 | TonB-dependent receptor |
| RJ610_20200 | WND79588.1 | 24,81 | 1,34E-63 | 75,42 | 6,59E-05 | glycoside hydrolase family 16 protein |
| RJ610_14265 | WND78472.1 | 335,63 | 2,41E-174 | 73,85 | 1,00E+00 | chitinase |
| RJ610_04240 | WND81595.1 | 62,29 | 1,21E-145 | 70,00 | 1,59E-04 | M23 family metallopeptidase |
| RJ610_07205 | WND82144.1 | 6,62 | 4,03E-18 | 61,88 | 2,16E-03 | DUF1800 domain-containing protein |
| RJ610_15610 | WND78730.1 | 660,88 | 8,07E-56 | 37,35 | 1,18E-04 | S1 family peptidase |
| RJ610_13660 | WND78360.1 | 555,94 | 4,06E-231 | 34,33 | 1,00E+00 | DUF1501 domain-containing protein |
| RJ610_13255 | WND78283.1 | 345,94 | 2,04E-50 | 32,99 | 3,37E-05 | M4 family metallopeptidase |
| RJ610_22540 | WND80027.1 | 646,53 | 0,00E+00 | 32,21 | 8,44E-05 | glycoside hydrolase family 16 protein |
| RJ610_23840 | WND80271.1 | 40,69 | 2,31E-95 | 31,15 | 4,78E-04 | serine protease |
| RJ610_01140 | WND81013.1 | 10,44 | 2,82E-48 | 26,53 | 1,41E-04 | DNRLRE domain-containing protein |
| RJ610_15530 | WND78716.1 | 29,32 | 1,23E-246 | 21,65 | 1,26E-04 | S8 family serine peptidase |
| RJ610_04900 | WND81710.1 | 36,06 | 3,49E-229 | 21,41 | 1,00E+00 | DUF6289 family protein |
| RJ610_05100 | WND81746.1 | 115,41 | 2,71E-241 | 19,66 | 2,80E-03 | M12 family metallo-peptidase |
| RJ610_18285 | WND79233.1 | 49,41 | 6,67E-120 | 18,11 | 8,10E-05 | PKD domain-containing protein |
| RJ610_22340 | WND79988.1 | 140,07 | 2,77E-95 | 15,55 | 1,72E-04 | alginate lyase family protein |
| RJ610_04550 | WND81645.1 | 38,97 | 3,74E-120 | 13,41 | 2,16E-04 | VOC family protein |
| RJ610_18270 | WND79230.1 | 117,79 | 2,17E-26 | 13,31 | 2,62E-04 | M20/M25/M40 family metallo-hydrolase |
| RJ610_25430 | WND80580.1 | 5,16 | 1,32E-35 | 13,30 | 3,44E-06 | hypothetical protein |
| RJ610_02270 | WND81222.1 | 4,58 | 6,16E-34 | 12,92 | 2,59E-03 | TonB-dependent receptor |
| RJ610_18995 | WND79366.1 | 35,67 | 1,90E-178 | 11,01 | 1,51E-04 | glycoside hydrolase family 6 protein |
| RJ610_13405 | WND78312.1 | 2,51 | 1,12E-25 | 10,70 | 4,05E-03 | beta-phosphoglucomutase |
| RJ610_07045 | WND82113.1 | 77,44 | 2,30E-103 | 10,42 | 1,29E-04 | carbohydrate-binding protein |
| RJ610_22880 | WND80089.1 | 4,17 | 1,22E-15 | 9,67 | 1,64E-03 | lytic polysaccharide monooxygenase |
| RJ610_09635 | WND82588.1 | 165,57 | 1,10E-120 | 9,43 | 1,43E-03 | M35 family metallo-endopeptidase |
| RJ610_03230 | WND81408.1 | 2,20 | 3,86E-13 | 7,13 | 4,54E-02 | efflux RND transporter permease subunit |
| RJ610_20895 | WND79720.1 | 96,58 | 1,38E-81 | 7,00 | 5,53E-05 | glycosyl hydrolase family 8 |
| RJ610_20015 | WND79555.1 | 1,38 | 3,16E-03 | 6,89 | 3,59E-03 | type II secretion system secretin GspD |
| RJ610_02080 | WND81185.1 | 1,33 | 4,09E-03 | 6,09 | 1,00E+00 | cytochrome c oxidase subunit II |
| RJ610_06010 | WND81918.1 | 10,30 | 1,96E-24 | 5,58 | 1,11E-05 | hemopexin repeat-containing protein |
| RJ610_07230 | WND82149.1 | 0,95 | 7,89E-01 | 5,57 | 1,37E-02 | ubiquinol-cytochrome c reductase iron-sulfur subunit |
| RJ610_18295 | WND79235.1 | 12,77 | 7,48E-23 | 5,46 | 6,26E-03 | M12 family metallo-peptidase |
| RJ610_23970 | WND80297.1 | 0,62 | 1,39E-03 | 5,37 | 2,60E-03 | TonB-dependent receptor |
| RJ610_18280 | WND79232.1 | 75,23 | 3,92E-126 | 5,33 | 2,36E-02 | M14 family zinc carboxypeptidase |

| | | | | | | |
|---|---|---|---|---|---|---|
| RJ610_18275 | WND79231.1 | 6,63 | 5,64E-26 | 5,33 | 2,36E-02 | M14 family zinc carboxypeptidase |
| RJ610_15605 | WND78729.1 | 14,93 | 4,05E-32 | 5,32 | 2,33E-03 | S1 family peptidase |
| RJ610_12605 | WND83136.1 | 97,09 | 0,00E+00 | 4,91 | 8,95E-04 | glycoside hydrolase family 16 protein |
| RJ610_17675 | WND79123.1 | 0,88 | 3,97E-01 | 4,70 | 5,20E-06 | OmpA family protein |
| RJ610_13250 | WND78282.1 | 28,23 | 9,90E-38 | 4,66 | 3,85E-04 | M4 family metallopeptidase |
| RJ610_03140 | WND81390.1 | 17,12 | 2,90E-60 | 4,62 | 7,02E-04 | peptidoglycan recognition family protein |
| RJ610_18290 | WND79234.1 | 7,10 | 7,53E-20 | 4,47 | 1,71E-04 | PKD domain-containing protein |
| RJ610_08245 | WND82331.1 | 0,78 | 9,62E-03 | 4,28 | 7,20E-04 | outer membrane protein assembly factor BamA |
| RJ610_02800 | WND81324.1 | 9,64 | 2,07E-20 | 4,22 | 1,48E-03 | hypothetical protein |
| RJ610_09115 | WND82488.1 | 0,95 | 6,76E-01 | 3,58 | 9,16E-03 | 4-hydroxy-tetrahydrodipicolinate synthase |
| RJ610_00445 | WND80879.1 | 2,53 | 9,11E-16 | 3,56 | 5,54E-03 | efflux transporter outer membrane subunit |
| RJ610_17260 | WND79041.1 | 3,12 | 1,31E-21 | 3,40 | 4,66E-03 | TonB-dependent receptor |
| RJ610_21360 | WND79807.1 | 1,07 | 5,07E-01 | 3,38 | 2,42E-04 | leucyl aminopeptidase family protein |
| RJ610_19510 | WND79465.1 | 1,43 | 5,98E-01 | 3,32 | 4,75E-04 | TonB-dependent receptor |
| RJ610_19605 | WND79479.1 | 1,99 | 3,04E-05 | 3,32 | 4,75E-04 | TonB-dependent receptor |
| RJ610_16400 | WND78876.1 | 0,87 | 1,73E-01 | 3,23 | 8,37E-02 | SRPBCC family protein |
| RJ610_22630 | WND83335.1 | 1,29 | 2,31E-02 | 3,15 | 6,98E-03 | sugar phosphate isomerase/epimerase family protein |
| RJ610_13570 | WND78342.1 | 1,11 | 3,85E-01 | 3,12 | 4,30E-04 | amidohydrolase |
| RJ610_08595 | WND82396.1 | 16,38 | 9,90E-96 | 3,07 | 1,69E-02 | S8 family peptidase |
| RJ610_22500 | WND80019.1 | 6,18 | 2,20E-77 | 2,89 | 1,49E-03 | cellulase family glycosylhydrolase |
| RJ610_08570 | WND82391.1 | 1,18 | 1,88E-01 | 2,87 | 1,00E+00 | DUF4139 domain-containing protein |
| RJ610_00835 | WND80954.1 | 1,23 | 1,33E-01 | 2,84 | 2,30E-04 | META and DUF4377 domain-containing protein |
| RJ610_11470 | WND82917.1 | 0,33 | 2,82E-24 | 2,75 | 1,00E+00 | putative Ig domain-containing protein |
| RJ610_23750 | WND80253.1 | 0,98 | 8,59E-01 | 2,75 | 1,44E-03 | gamma carbonic anhydrase family protein |
| RJ610_22930 | WND80098.1 | 0,46 | 3,38E-18 | 2,69 | 1,00E+00 | signal peptide peptidase SppA |
| RJ610_07260 | WND82154.1 | 0,74 | 3,04E-03 | 2,67 | 1,93E-03 | carboxylating nicotinate-nucleotide diphosphorylase |
| RJ610_03235 | WND81409.1 | 2,34 | 5,91E-12 | 2,67 | 2,22E-02 | efflux transporter outer membrane subunit |
| RJ610_15225 | WND78659.1 | 1,15 | 3,63E-01 | 2,49 | 5,85E-04 | GTP cyclohydrolase FolE2 |
| RJ610_09540 | WND82570.1 | 0,90 | 2,97E-01 | 2,47 | 4,84E-02 | TonB-dependent receptor |
| RJ610_03225 | WND81407.1 | 1,69 | 4,56E-10 | 2,46 | 1,55E-01 | efflux RND transporter periplasmic adaptor subunit |
| RJ610_00065 | WND80805.1 | 1,38 | 3,78E-03 | 2,42 | 9,09E-03 | pyridoxine 5'-phosphate synthase |
| RJ610_01320 | WND81048.1 | 3,46 | 8,03E-12 | 2,34 | 7,18E-02 | CocE/NonD family hydrolase |
| RJ610_16975 | WND78988.1 | 1,63 | 9,09E-07 | 2,34 | 1,08E-03 | glutathione-disulfide reductase |
| RJ610_00010 | WND80795.1 | 0,69 | 9,13E-05 | 2,32 | 1,17E-01 | DNA polymerase III subunit beta |
| RJ610_06775 | WND82061.1 | 4,97 | 1,40E-17 | 2,32 | 1,00E+00 | cytochrome ubiquinol oxidase subunit I |
| RJ610_05600 | WND83184.1 | 39,17 | 5,47E-237 | 2,29 | 1,32E-02 | glycosyl hydrolase family 18 protein |
| RJ610_18525 | WND79278.1 | 0,86 | 1,93E-01 | 2,26 | 4,60E-02 | peptide-N4-asparagine amidase |
| RJ610_01805 | WND81137.1 | 6,17 | 4,40E-13 | 2,26 | 2,97E-02 | M12 family metallo-peptidase |
| RJ610_00605 | WND80910.1 | 0,70 | 4,65E-04 | 2,25 | 1,26E-05 | N-acetylglucosamine-6-phosphate deacetylase |
| RJ610_13410 | WND78313.1 | 3,04 | 3,04E-31 | 2,24 | 2,20E-02 | TonB-dependent receptor |
| RJ610_13500 | WND78329.1 | 1,20 | 2,48E-01 | 2,21 | 5,00E-02 | TonB-dependent receptor |
| RJ610_01865 | WND81148.1 | 0,54 | 2,89E-06 | 2,19 | 4,62E-03 | dUTP diphosphatase |
| RJ610_15245 | WND78663.1 | 1,11 | 4,66E-01 | 2,18 | 1,18E-02 | TRZ/ATZ family hydrolase |

| | | | | | | |
|---|---|---|---|---|---|---|
| RJ610_09715 | WND82600.1 | 0,92 | 7,03E-01 | 2,17 | 1,00E+00 | succinate dehydrogenase iron-sulfur subunit |
| RJ610_05080 | WND81742.1 | 0,94 | 5,78E-01 | 2,09 | 1,26E-01 | response regulator transcription factor |
| RJ610_19920 | WND79537.1 | 1,08 | 6,00E-01 | 2,09 | 1,11E-02 | riboflavin synthase |
| RJ610_20450 | WND79637.1 | 0,80 | 1,65E-01 | 2,01 | 4,42E-04 | beta-aspartyl-peptidase |
| RJ610_07350 | WND82171.1 | 1,70 | 5,20E-05 | 2,00 | 2,12E-02 | PilC/PilY family type IV pilus protein |
| RJ610_10270 | WND82697.1 | 8,30 | 9,30E-47 | 1,99 | 8,41E-03 | isocitrate lyase/phosphoenolpyruvate mutase family protein |
| RJ610_22680 | WND80051.1 | 3,11 | 3,92E-17 | 1,93 | 3,90E-02 | TonB-dependent receptor |
| RJ610_22950 | WND80102.1 | 1,23 | 1,56E-01 | 1,91 | 1,49E-02 | serine protease |
| RJ610_10900 | WND82811.1 | 0,44 | 2,78E-07 | 1,84 | 2,42E-01 | TrbG/VirB9 family P-type conjugative transfer protein |
| RJ610_15870 | WND78777.1 | 1,16 | 2,20E-01 | 1,83 | 1,67E-01 | Slp family lipoprotein |
| RJ610_19385 | WND79442.1 | 0,51 | 2,21E-09 | 1,81 | 1,44E-01 | peptidoglycan-associated lipoprotein Pal |
| RJ610_13245 | WND78281.1 | 6,50 | 6,82E-22 | 1,79 | 1,04E-02 | M4 family metallopeptidase |
| RJ610_08030 | WND82295.1 | 1,25 | 3,51E-02 | 1,73 | 6,42E-02 | SRPBCC family protein |
| RJ610_03715 | WND81498.1 | 1,02 | 8,56E-01 | 1,72 | 1,44E-01 | 6-carboxytetrahydropterin synthase QueD |
| RJ610_21720 | WND79873.1 | 1,10 | 7,22E-01 | 1,70 | 6,06E-02 | ATP-dependent protease subunit HslV |
| RJ610_18010 | WND79186.1 | 1,45 | 5,07E-03 | 1,68 | 1,89E-01 | 50S ribosomal protein L22 |
| RJ610_05050 | WND83179.1 | 0,97 | 8,14E-01 | 1,65 | 5,32E-02 | autotransporter domain-containing protein |
| RJ610_07525 | WND82201.1 | 0,10 | 1,90E-26 | 1,64 | 2,59E-01 | DUF6689 family protein |
| RJ610_01815 | WND81139.1 | 0,66 | 7,11E-04 | 1,63 | 1,16E-02 | arginine decarboxylase |
| RJ610_15150 | WND78645.1 | 4,56 | 3,93E-13 | 1,62 | 2,70E-01 | acetylornithine deacetylase |
| RJ610_06435 | WND81995.1 | 0,68 | 5,37E-03 | 1,62 | 4,50E-02 | PhzF family phenazine biosynthesis protein |
| RJ610_04915 | WND81713.1 | 1,48 | 2,67E-04 | 1,61 | 6,85E-02 | pyruvate kinase |
| RJ610_18035 | WND79191.1 | 0,82 | 1,48E-01 | 1,60 | 2,66E-01 | 50S ribosomal protein L3 |
| RJ610_13465 | WND78324.1 | 1,40 | 5,94E-03 | 1,59 | 4,66E-02 | glucokinase |
| RJ610_04250 | WND81597.1 | 0,83 | 5,91E-02 | 1,58 | 1,00E+00 | phosphomethylpyrimidine synthase ThiC |
| RJ610_04385 | WND81621.1 | 1,06 | 5,90E-01 | 1,57 | 1,16E-02 | acetylornithine/succinyldiaminopimelate transaminase |
| RJ610_04095 | WND81568.1 | 0,94 | 6,11E-01 | 1,57 | 1,00E+00 | pirin family protein |
| RJ610_13590 | WND78346.1 | 4,46 | 4,96E-23 | 1,53 | 1,10E-02 | peptidoglycan-binding protein |
| RJ610_16875 | WND78968.1 | 0,81 | 1,09E-01 | 1,53 | 7,42E-02 | 2-C-methyl-D-erythritol 2,4-cyclodiphosphate synthase |
| RJ610_16095 | WND78817.1 | 0,25 | 1,16E-14 | 1,53 | 5,17E-01 | hypothetical protein |
| RJ610_02020 | WND81173.1 | 2,08 | 1,02E-17 | 1,53 | 2,55E-01 | outer membrane protein transport protein |
| RJ610_18620 | WND79295.1 | 0,96 | 6,74E-01 | 1,52 | 1,69E-02 | LPS assembly protein LptD |
| RJ610_26235 | WND80735.1 | 1,14 | 3,22E-01 | 1,51 | 2,51E-01 | M20 family metallopeptidase |
| RJ610_18030 | WND79190.1 | 1,04 | 7,78E-01 | 1,49 | 3,38E-01 | 50S ribosomal protein L4 |
| RJ610_03505 | WND81457.1 | 1,57 | 2,25E-05 | 1,46 | 1,33E-01 | F0F1 ATP synthase subunit alpha |
| RJ610_05165 | WND81758.1 | 1,79 | 1,73E-07 | 1,43 | 2,44E-02 | adenosylhomocysteinase |
| RJ610_04270 | WND81601.1 | 2,83 | 1,84E-01 | 1,43 | 4,69E-02 | TonB-dependent receptor |
| RJ610_17415 | WND79071.1 | 0,82 | 3,95E-01 | 1,37 | 1,00E+00 | VOC family protein |
| RJ610_20110 | WND79572.1 | 1,20 | 2,84E-01 | 1,36 | 2,51E-02 | leucyl aminopeptidase |
| RJ610_06075 | WND81931.1 | 0,88 | 2,19E-01 | 1,36 | 2,64E-02 | phosphoribosylformylglycinamidine cyclo-ligase |
| RJ610_17510 | WND79090.1 | 1,17 | 4,86E-01 | 1,36 | 2,29E-01 | TonB-dependent receptor |
| RJ610_13275 | WND83250.1 | 0,87 | 4,59E-01 | 1,36 | 3,84E-01 | S8 family serine peptidase |
| RJ610_08360 | WND82353.1 | 1,15 | 3,46E-01 | 1,34 | 1,11E-01 | succinyl-diaminopimelate desuccinylase |

| | | | | | | |
|---|---|---|---|---|---|---|
| RJ610_20135 | WND83307.1 | 10,07 | 4,24E-47 | 1,34 | 5,70E-01 | catalase |
| RJ610_04520 | WND81639.1 | 0,77 | 1,13E-02 | 1,34 | 2,06E-01 | Xaa-Pro dipeptidase |
| RJ610_20395 | WND79626.1 | 0,73 | 9,52E-03 | 1,34 | 3,78E-01 | 50S ribosomal protein L13 |
| RJ610_07635 | WND82222.1 | 1,19 | 3,09E-01 | 1,30 | 4,30E-02 | aminotransferase class III-fold pyridoxal phosphate-dependent enzyme |
| RJ610_13455 | WND78322.1 | 1,20 | 2,05E-01 | 1,29 | 4,83E-02 | GH1 family beta-glucosidase |
| RJ610_01810 | WND81138.1 | 0,71 | 3,63E-04 | 1,27 | 3,39E-01 | polyamine aminopropyltransferase |
| RJ610_18190 | WND79215.1 | 0,65 | 4,36E-04 | 1,26 | 1,14E-01 | molybdenum cofactor biosynthesis protein B |
| RJ610_06870 | WND82079.1 | 6,21 | 1,22E-10 | 1,25 | 2,87E-01 | pectate lyase |
| RJ610_03435 | WND81444.1 | 0,85 | 3,03E-01 | 1,25 | 4,00E-01 | OmpW family outer membrane protein |
| RJ610_13730 | WND78374.1 | 1,25 | 3,43E-02 | 1,23 | 2,12E-01 | polyribonucleotide nucleotidyltransferase |
| RJ610_19985 | WND79550.1 | 2,47 | 1,85E-13 | 1,23 | 4,16E-01 | orotidine-5'-phosphate decarboxylase |
| RJ610_01870 | WND81149.1 | 0,51 | 1,05E-08 | 1,23 | 6,06E-01 | phosphomannomutase/phosphoglucomutase |
| RJ610_07270 | WND82156.1 | 0,72 | 1,07E-03 | 1,21 | 4,73E-01 | 5-(carboxyamino)imidazole ribonucleotide mutase |
| RJ610_03515 | WND81459.1 | 1,65 | 1,44E-06 | 1,20 | 4,95E-01 | F0F1 ATP synthase subunit beta |
| RJ610_09595 | WND82581.1 | 0,94 | 6,84E-01 | 1,20 | 2,15E-01 | NIPSNAP family protein |
| RJ610_22845 | WND80082.1 | 1,54 | 9,63E-07 | 1,19 | 5,65E-01 | peptide deformylase |
| RJ610_17985 | WND79181.1 | 1,58 | 1,08E-03 | 1,19 | 7,67E-01 | 50S ribosomal protein L14 |
| RJ610_05245 | WND81773.1 | 0,63 | 3,78E-04 | 1,18 | 1,00E+00 | ribokinase |
| RJ610_21695 | WND79869.1 | 0,76 | 8,45E-02 | 1,17 | 4,42E-01 | nucleoside deaminase |
| RJ610_06260 | WND81962.1 | 0,57 | 5,48E-07 | 1,16 | 1,53E-01 | Fe2+-dependent dioxygenase |
| RJ610_18020 | WND79188.1 | 1,11 | 5,09E-01 | 1,15 | 6,15E-01 | 50S ribosomal protein L2 |
| RJ610_04825 | WND81696.1 | 1,09 | 3,84E-01 | 1,13 | 2,46E-01 | type I glyceraldehyde-3-phosphate dehydrogenase |
| RJ610_14505 | WND78520.1 | 0,70 | 4,97E-02 | 1,13 | 5,57E-01 | S1 family peptidase |
| RJ610_14380 | WND78495.1 | 1,88 | 3,69E-02 | 1,12 | 6,78E-01 | hypothetical protein |
| RJ610_17980 | WND79180.1 | 1,49 | 1,47E-03 | 1,12 | 7,81E-01 | 50S ribosomal protein L24 |
| RJ610_16535 | WND78902.1 | 0,94 | 6,66E-01 | 1,12 | 1,71E-02 | aminomethyl-transferring glycine dehydrogenase |
| RJ610_22765 | WND80067.1 | 1,03 | 8,37E-01 | 1,11 | 4,68E-01 | AGE family epimerase/isomerase |
| RJ610_16965 | WND78986.1 | 1,38 | 9,07E-04 | 1,10 | 5,11E-01 | N-formylglutamate deformylase |
| RJ610_01370 | WND83365.1 | 0,84 | 2,29E-01 | 1,10 | 6,96E-01 | diffusible signal factor-reguated Ax21 family protein |
| RJ610_08425 | WND82365.1 | 2,26 | 4,86E-13 | 1,08 | 5,25E-01 | serine protease |
| RJ610_05505 | WND81825.1 | 0,50 | 3,46E-09 | 1,07 | 7,18E-01 | TonB-dependent receptor |
| RJ610_05500 | WND81824.1 | 1,30 | 1,01E-01 | 1,07 | 7,18E-01 | TonB-dependent receptor |
| RJ610_07290 | WND82160.1 | 1,35 | 1,37E-03 | 1,06 | 5,29E-01 | Fe-Mn family superoxide dismutase |
| RJ610_07275 | WND82157.1 | 0,68 | 1,05E-02 | 1,05 | 8,55E-01 | 5-(carboxyamino)imidazole ribonucleotide synthase |
| RJ610_18800 | WND83293.1 | 1,07 | 6,95E-01 | 1,03 | 8,97E-01 | adenosylmethionine--8-amino-7-oxononanoate transaminase |
| RJ610_07315 | WND82165.1 | 0,66 | 9,60E-06 | 1,03 | 4,29E-01 | nucleotide 5'-monophosphate nucleosidase PpnN |
| RJ610_20575 | WND79661.1 | 1,87 | 2,18E-08 | 1,02 | 9,34E-01 | MarR family winged helix-turn-helix transcriptional regulator |
| RJ610_14255 | WND78470.1 | 2,70 | 1,18E-17 | 1,01 | 8,16E-01 | RidA family protein |
| RJ610_13180 | WND78270.1 | 1,16 | 2,00E-01 | 1,01 | 9,24E-01 | dihydrolipoyl dehydrogenase |
| RJ610_20505 | WND79648.1 | 0,98 | 8,39E-01 | 1,00 | 9,87E-01 | ribulose-phosphate 3-epimerase |
| RJ610_11440 | WND82912.1 | 1,03 | 8,86E-01 | 1,00 | 9,91E-01 | thioredoxin-disulfide reductase |
| RJ610_02950 | WND81354.1 | 0,46 | 2,89E-11 | 0,99 | 9,50E-01 | hypothetical protein |

| | | | | | | |
|---|---|---|---|---|---|---|
| RJ610_12840 | WND78203.1 | 1,14 | 2,63E-01 | 0,96 | 8,58E-01 | carbonate dehydratase |
| RJ610_22415 | WND80003.1 | 1,34 | 4,31E-02 | 0,96 | 7,65E-01 | MBL fold metallo-hydrolase |
| RJ610_04125 | WND81574.1 | 0,73 | 3,31E-03 | 0,95 | 6,28E-01 | TolC family outer membrane protein |
| RJ610_13745 | WND78377.1 | 1,10 | 4,87E-01 | 0,95 | 1,00E+00 | heme-binding protein |
| RJ610_22320 | WND79984.1 | 2,28 | 1,54E-14 | 0,95 | 8,85E-01 | superoxide dismutase |
| RJ610_03960 | WND81543.1 | 1,38 | 8,92E-03 | 0,93 | 7,76E-01 | protein-disulfide reductase DsbD |
| RJ610_16675 | WND78930.1 | 0,72 | 1,96E-02 | 0,93 | 8,59E-01 | MipA/OmpV family protein |
| RJ610_07310 | WND82164.1 | 0,54 | 6,72E-09 | 0,92 | 5,81E-01 | carboxypeptidase regulatory-like domain-containing protein |
| RJ610_02290 | WND81226.1 | 1,26 | 1,51E-02 | 0,91 | 6,20E-01 | thioredoxin domain-containing protein |
| RJ610_05090 | WND81744.1 | 1,02 | 8,53E-01 | 0,91 | 5,21E-01 | branched-chain amino acid transaminase |
| RJ610_03050 | WND81373.1 | 1,11 | 5,24E-01 | 0,90 | 4,94E-01 | hypothetical protein |
| RJ610_13545 | WND78337.1 | 1,31 | 2,69E-01 | 0,88 | 1,53E-01 | serine hydrolase domain-containing protein |
| RJ610_00765 | WND80941.1 | 2,08 | 5,19E-09 | 0,88 | 6,08E-01 | TonB-dependent receptor |
| RJ610_17680 | WND79124.1 | 1,08 | 4,53E-01 | 0,88 | 3,78E-01 | bifunctional 2-methylcitrate dehydratase/aconitate hydratase |
| RJ610_20675 | WND79680.1 | 1,38 | 1,95E-03 | 0,87 | 3,22E-01 | arginase |
| RJ610_26130 | WND80716.1 | 0,61 | 2,03E-03 | 0,87 | 6,07E-01 | DUF2170 family protein |
| RJ610_03015 | WND81366.1 | 0,84 | 4,38E-01 | 0,86 | 3,40E-01 | OsmC family protein |
| RJ610_17460 | WND79080.1 | 5,12 | 3,46E-13 | 0,86 | 4,33E-02 | DUF4082 domain-containing protein |
| RJ610_07980 | WND83202.1 | 0,66 | 3,60E-06 | 0,85 | 3,45E-01 | LPS assembly lipoprotein LptE |
| RJ610_14595 | WND78538.1 | 0,97 | 8,38E-01 | 0,84 | 1,37E-01 | S8 family serine peptidase |
| RJ610_16215 | WND78840.1 | 1,68 | 1,04E-05 | 0,84 | 1,00E+00 | hypothetical protein |
| RJ610_08190 | WND82321.1 | 0,98 | 9,15E-01 | 0,83 | 2,94E-01 | NADH:flavin oxidoreductase/NADH oxidase |
| RJ610_11575 | WND82937.1 | 5,28 | 1,18E-05 | 0,82 | 1,81E-01 | substrate-binding domain-containing protein |
| RJ610_14435 | WND78506.1 | 1,04 | 7,84E-01 | 0,81 | 5,36E-01 | M14 family metallocarboxypeptidase |
| RJ610_11260 | WND82878.1 | 2,03 | 5,53E-11 | 0,81 | 4,25E-01 | TonB-dependent receptor |
| RJ610_06215 | WND81954.1 | 0,63 | 4,48E-05 | 0,81 | 9,58E-02 | M20 family metallopeptidase |
| RJ610_02640 | WND81294.1 | 0,59 | 2,66E-07 | 0,81 | 3,67E-01 | NADP-dependent malic enzyme |
| RJ610_03690 | WND81493.1 | 0,92 | 4,61E-01 | 0,81 | 1,63E-01 | TonB-dependent vitamin B12 receptor |
| RJ610_13495 | WND78328.1 | 1,48 | 5,38E-03 | 0,81 | 1,00E+00 | glucoamylase family protein |
| RJ610_15010 | WND78619.1 | 0,96 | 7,88E-01 | 0,81 | 3,23E-03 | aromatic amino acid lyase |
| RJ610_23615 | WND80226.1 | 0,45 | 8,29E-17 | 0,80 | 5,21E-01 | PA domain-containing protein |
| RJ610_14330 | WND78485.1 | 0,82 | 2,21E-01 | 0,80 | 1,00E+00 | VOC family protein |
| RJ610_25660 | WND80626.1 | 0,64 | 2,07E-06 | 0,80 | 3,62E-01 | TonB-dependent siderophore receptor |
| RJ610_03550 | WND81466.1 | 4,66 | 1,58E-27 | 0,79 | 1,54E-01 | hypothetical protein |
| RJ610_04815 | WND81694.1 | 1,06 | 6,68E-01 | 0,78 | 1,00E+00 | S1/P1 nuclease |
| RJ610_26370 | WND80759.1 | 2,56 | 2,68E-14 | 0,77 | 9,26E-02 | hypothetical protein |
| RJ610_13855 | WND78395.1 | 0,81 | 3,55E-02 | 0,77 | 8,21E-02 | triose-phosphate isomerase |
| RJ610_05710 | WND81861.1 | 1,40 | 9,02E-02 | 0,74 | 1,00E+00 | uroporphyrinogen decarboxylase |
| RJ610_17670 | WND79122.1 | 1,16 | 1,19E-01 | 0,74 | 3,88E-01 | OmpA family protein |
| RJ610_14580 | WND78535.1 | 0,90 | 3,22E-01 | 0,74 | 4,34E-02 | Glu/Leu/Phe/Val dehydrogenase dimerization domain-containing protein |
| RJ610_13645 | WND78357.1 | 1,05 | 7,51E-01 | 0,73 | 4,34E-01 | hypothetical protein |
| RJ610_22480 | WND80015.1 | 12,28 | 7,81E-164 | 0,73 | 1,02E-01 | TonB-dependent receptor |

| | | | | | | |
|---|---|---|---|---|---|---|
| RJ610_13595 | WND78347.1 | 5,30 | 2,21E-26 | 0,70 | 2,02E-01 | hypothetical protein |
| RJ610_22850 | WND80083.1 | 2,43 | 2,65E-18 | 0,70 | 1,00E+00 | LysM peptidoglycan-binding domain-containing protein |
| RJ610_20930 | WND79726.1 | 1,01 | 9,28E-01 | 0,70 | 3,87E-02 | DUF1428 domain-containing protein |
| RJ610_03470 | WND83164.1 | 2,38 | 6,77E-06 | 0,69 | 1,37E-01 | hypothetical protein |
| RJ610_19905 | WND79534.1 | 1,01 | 9,42E-01 | 0,68 | 1,44E-02 | 6,7-dimethyl-8-ribityllumazine synthase |
| RJ610_13395 | WND78310.1 | 1,00 | 9,94E-01 | 0,68 | 1,22E-02 | serine hydrolase |
| RJ610_12875 | WND78210.1 | 1,17 | 1,65E-01 | 0,68 | 2,64E-01 | NAD(P)/FAD-dependent oxidoreductase |
| RJ610_06705 | WND82047.1 | 1,00 | 9,76E-01 | 0,67 | 2,20E-02 | single-stranded DNA-binding protein |
| RJ610_01275 | WND81039.1 | 1,01 | 9,58E-01 | 0,67 | 1,39E-01 | superoxide dismutase family protein |
| RJ610_03120 | WND81386.1 | 0,79 | 7,78E-02 | 0,67 | 4,69E-02 | lactonase family protein |
| RJ610_17085 | WND79009.1 | 1,84 | 1,45E-04 | 0,67 | 1,23E-01 | alkaline phosphatase |
| RJ610_23365 | WND80182.1 | 0,92 | 5,73E-01 | 0,66 | 2,00E-02 | oxygen-dependent coproporphyrinogen oxidase |
| RJ610_05495 | WND81823.1 | 0,43 | 1,12E-04 | 0,66 | 6,99E-02 | TonB-dependent receptor |
| RJ610_18045 | WND79193.1 | 0,98 | 9,20E-01 | 0,66 | 4,15E-01 | elongation factor Tu |
| RJ610_18110 | WND79204.1 | 0,82 | 5,60E-02 | 0,66 | 4,15E-01 | elongation factor Tu |
| RJ610_15790 | WND78764.1 | 0,92 | 5,70E-01 | 0,65 | 1,00E+00 | beta-ketoacyl-ACP synthase II |
| RJ610_11250 | WND83235.1 | 3,76 | 4,52E-39 | 0,63 | 1,00E+00 | GH92 family glycosyl hydrolase |
| RJ610_11035 | WND83233.1 | 1,11 | 5,83E-01 | 0,63 | 2,46E-01 | CoA-acylating methylmalonate-semialdehyde dehydrogenase |
| RJ610_23560 | WND80217.1 | 0,43 | 2,03E-10 | 0,62 | 9,44E-03 | autotransporter domain-containing protein |
| RJ610_17430 | WND79074.1 | 1,28 | 8,54E-02 | 0,62 | 4,71E-03 | urocanate hydratase |
| RJ610_00375 | WND80866.1 | 0,94 | 5,92E-01 | 0,61 | 3,04E-01 | MlaA family lipoprotein |
| RJ610_20220 | WND79592.1 | 1,13 | 3,67E-01 | 0,59 | 1,00E+00 | CoA transferase subunit B |
| RJ610_21590 | WND79848.1 | 5,45 | 6,50E-64 | 0,59 | 1,49E-01 | glycosyl hydrolase family 18 protein |
| RJ610_11395 | WND82904.1 | 0,14 | 4,49E-45 | 0,59 | 1,00E+00 | hypothetical protein |
| RJ610_07530 | WND82202.1 | 0,06 | 4,04E-73 | 0,58 | 1,00E-01 | S8 family serine peptidase |
| RJ610_23375 | WND80184.1 | 1,85 | 5,93E-09 | 0,58 | 5,76E-02 | TonB-dependent receptor |
| RJ610_15160 | WND78647.1 | 2,85 | 1,10E-08 | 0,58 | 1,14E-01 | N-acetylornithine carbamoyltransferase |
| RJ610_23305 | WND80170.1 | 1,09 | 6,30E-01 | 0,57 | 2,68E-01 | thioredoxin |
| RJ610_18140 | WND79207.1 | 0,80 | 5,61E-02 | 0,57 | 2,17E-01 | 50S ribosomal protein L25/general stress protein Ctc |
| RJ610_20325 | WND79613.1 | 0,83 | 8,02E-02 | 0,57 | 3,09E-02 | cystathionine gamma-synthase |
| RJ610_04395 | WND81623.1 | 0,60 | 6,20E-06 | 0,56 | 1,25E-02 | TonB-dependent siderophore receptor |
| RJ610_21325 | WND79802.1 | 1,17 | 2,69E-01 | 0,56 | 1,00E+00 | Do family serine endopeptidase |
| RJ610_12565 | WND83128.1 | 7,18 | 1,03E-40 | 0,55 | 2,82E-02 | hypothetical protein |
| RJ610_05440 | WND81812.1 | 0,25 | 6,00E-42 | 0,55 | 1,00E+00 | putative Ig domain-containing protein |
| RJ610_14210 | WND78461.1 | 2,56 | 4,80E-07 | 0,54 | 2,21E-02 | PHB depolymerase family esterase |
| RJ610_00640 | WND80916.1 | 0,75 | 6,39E-03 | 0,52 | 2,55E-02 | bifunctional 4-hydroxy-2-oxoglutarate aldolase/2-dehydro-3-deoxy-phosphogluconate aldolase |
| RJ610_04235 | WND81594.1 | 0,96 | 7,61E-01 | 0,52 | 1,00E+00 | YbhB/YbcL family Raf kinase inhibitor-like protein |
| RJ610_20655 | WND79676.1 | 0,83 | 1,17E-01 | 0,52 | 1,00E+00 | tryptophan--tRNA ligase |
| RJ610_17645 | WND79117.1 | 0,72 | 5,99E-04 | 0,50 | 1,00E+00 | L-threonine 3-dehydrogenase |
| RJ610_12665 | WND83148.1 | 1,09 | 6,00E-01 | 0,49 | 2,94E-02 | S8 family serine peptidase |
| RJ610_14480 | WND78515.1 | 2,78 | 7,98E-24 | 0,47 | 4,01E-03 | polysaccharide lyase family 8 super-sandwich domain-containing protein |

| | | | | | | |
|---|---|---|---|---|---|---|
| RJ610_22000 | WND79926.1 | 1,22 | 6,44E-02 | 0,46 | 7,29E-02 | organic hydroperoxide resistance protein |
| RJ610_18265 | WND79229.1 | 1,45 | 5,46E-02 | 0,46 | 2,57E-02 | M20/M25/M40 family metallo-hydrolase |
| RJ610_06865 | WND82078.1 | 2,29 | 1,63E-08 | 0,46 | 9,06E-03 | hypothetical protein |
| RJ610_02570 | WND81280.1 | 1,44 | 2,70E-03 | 0,46 | 1,50E-02 | DUF924 family protein |
| RJ610_16750 | WND78944.1 | 2,93 | 1,31E-10 | 0,46 | 1,94E-04 | hypothetical protein |
| RJ610_26375 | WND80760.1 | 1,37 | 1,65E-02 | 0,46 | 7,86E-04 | hypothetical protein |
| RJ610_04845 | WND81700.1 | 4,27 | 1,78E-02 | 0,46 | 1,18E-01 | 5-methyltetrahydropteroyltriglutamate--homocysteine S-methyltransferase |
| RJ610_04860 | WND81703.1 | 0,62 | 5,66E-04 | 0,45 | 1,12E-02 | acyl-CoA thioesterase |
| RJ610_14375 | WND78494.1 | 3,46 | 8,26E-16 | 0,44 | 4,20E-03 | right-handed parallel beta-helix repeat-containing protein |
| RJ610_20625 | WND79670.1 | 4,16 | 6,24E-20 | 0,43 | 1,24E-01 | peptidoglycan DD-metalloendopeptidase family protein |
| RJ610_04990 | WND81727.1 | 0,85 | 2,24E-01 | 0,43 | 6,05E-03 | M20/M25/M40 family metallo-hydrolase |
| RJ610_01620 | WND81104.1 | 0,09 | 5,41E-37 | 0,43 | 4,00E-03 | isocitrate lyase |
| RJ610_20570 | WND79660.1 | 1,68 | 2,37E-01 | 0,43 | 1,00E+00 | 4-hydroxyphenylpyruvate dioxygenase |
| RJ610_20215 | WND79591.1 | 1,14 | 1,37E-01 | 0,42 | 1,17E-02 | FG-GAP-like repeat-containing protein |
| RJ610_17965 | WND79177.1 | 0,72 | 4,97E-03 | 0,42 | 1,36E-01 | 30S ribosomal protein S8 |
| RJ610_15940 | WND78789.1 | 1,10 | 5,76E-01 | 0,42 | 1,00E+00 | YncE family protein |
| RJ610_20905 | WND79722.1 | 1,29 | 6,62E-02 | 0,41 | 1,25E-01 | decarboxylating 6-phosphogluconate dehydrogenase |
| RJ610_10125 | WND82671.1 | 0,72 | 1,00E-01 | 0,41 | 6,25E-03 | 3-phosphoserine/phosphohydroxythreonine transaminase |
| RJ610_03150 | WND81392.1 | 3,22 | 1,36E-16 | 0,40 | 1,34E-02 | peptidoglycan DD-metalloendopeptidase family protein |
| RJ610_04030 | WND81556.1 | 1,73 | 3,04E-04 | 0,39 | 8,85E-02 | chaperonin GroEL |
| RJ610_04855 | WND81702.1 | 0,60 | 1,37E-07 | 0,39 | 1,29E-01 | TonB-dependent siderophore receptor |
| RJ610_22980 | WND80107.1 | 0,85 | 2,04E-01 | 0,39 | 7,29E-03 | transaldolase |
| RJ610_23025 | WND80116.1 | 0,30 | 1,27E-15 | 0,36 | 3,91E-02 | DUF5916 domain-containing protein |
| RJ610_08835 | WND82438.1 | 1,02 | 8,65E-01 | 0,36 | 2,28E-01 | 50S ribosomal protein L19 |
| RJ610_21935 | WND79913.1 | 0,16 | 2,69E-35 | 0,36 | 5,72E-04 | autotransporter domain-containing protein |
| RJ610_18000 | WND79184.1 | 1,45 | 4,36E-03 | 0,35 | 2,82E-01 | 50S ribosomal protein L16 |
| RJ610_21760 | WND79880.1 | 1,92 | 5,97E-05 | 0,35 | 7,46E-06 | S9 family peptidase |
| RJ610_08125 | WND83203.1 | 0,89 | 4,45E-01 | 0,35 | 5,93E-03 | SIMPL domain-containing protein |
| RJ610_22195 | WND79959.1 | 2,82 | 1,73E-09 | 0,34 | 5,47E-03 | hypothetical protein |
| RJ610_15440 | WND78699.1 | 1,12 | 2,63E-01 | 0,34 | 4,63E-04 | aldehyde dehydrogenase family protein |
| RJ610_09160 | WND82496.1 | 0,76 | 3,71E-02 | 0,33 | 1,79E-02 | aspartate 1-decarboxylase |
| RJ610_15205 | WND78655.1 | 1,01 | 9,25E-01 | 0,33 | 1,07E-02 | dihydroorotase |
| RJ610_09120 | WND82489.1 | 0,81 | 4,22E-02 | 0,33 | 5,72E-03 | outer membrane protein assembly factor BamC |
| RJ610_06265 | WND81963.1 | 0,47 | 7,14E-09 | 0,32 | 1,29E-03 | TonB-dependent receptor |
| RJ610_00525 | WND80894.1 | 0,44 | 2,08E-10 | 0,31 | 1,31E-02 | hypothetical protein |
| RJ610_05280 | WND81780.1 | 1,44 | 3,64E-02 | 0,30 | 4,29E-02 | TonB-dependent hemoglobin/transferrin/lactoferrin family receptor |
| RJ610_10140 | WND82674.1 | 0,61 | 2,50E-02 | 0,29 | 1,00E+00 | 3-phosphoshikimate 1-carboxyvinyltransferase |
| RJ610_13620 | WND78352.1 | 2,41 | 8,54E-22 | 0,29 | 9,97E-02 | peptidoglycan-binding domain-containing protein |
| RJ610_06245 | WND81960.1 | 0,72 | 3,50E-03 | 0,29 | 9,21E-04 | DUF4198 domain-containing protein |
| RJ610_17650 | WND79118.1 | 0,84 | 1,31E-01 | 0,28 | 1,00E+00 | glycine C-acetyltransferase |
| RJ610_02810 | WND81326.1 | 1,67 | 1,24E-02 | 0,28 | 9,37E-05 | agmatine deiminase family protein |
| RJ610_12465 | WND83108.1 | 1,27 | 3,72E-02 | 0,28 | 1,43E-03 | glycoside hydrolase family 9 protein |

| | | | | | | |
|---|---|---|---|---|---|---|
| RJ610_08515 | WND82383.1 | 0,43 | 3,15E-04 | 0,27 | 4,54E-02 | phosphate ABC transporter substrate-binding protein PstS |
| RJ610_14160 | WND78452.1 | 0,78 | 2,63E-02 | 0,27 | 1,89E-02 | DUF1993 domain-containing protein |
| RJ610_10760 | WND82787.1 | 0,72 | 3,39E-03 | 0,26 | 5,92E-03 | SGNH/GDSL hydrolase family protein |
| RJ610_02955 | WND81355.1 | 0,26 | 1,30E-34 | 0,25 | 2,39E-02 | hypothetical protein |
| RJ610_17975 | WND79179.1 | 1,40 | 1,20E-02 | 0,24 | 1,19E-02 | 50S ribosomal protein L5 |
| RJ610_23650 | WND80233.1 | 2,01 | 2,62E-03 | 0,24 | 3,29E-02 | sulfate ABC transporter substrate-binding protein |
| RJ610_11085 | WND82846.1 | 0,95 | 7,18E-01 | 0,23 | 2,20E-03 | M35 family metallo-endopeptidase |
| RJ610_17005 | WND78994.1 | 0,07 | 1,95E-87 | 0,23 | 4,04E-04 | hypothetical protein |
| RJ610_20595 | WND79665.1 | 0,13 | 2,51E-56 | 0,23 | 3,19E-02 | lamin tail domain-containing protein |
| RJ610_10710 | WND82777.1 | 0,56 | 3,13E-05 | 0,22 | 6,07E-04 | STM4015 family protein |
| RJ610_06405 | WND81989.1 | 1,40 | 7,86E-02 | 0,22 | 3,41E-02 | OsmC family protein |
| RJ610_19590 | WND83301.1 | 0,31 | 6,37E-24 | 0,22 | 4,63E-04 | TonB-dependent vitamin B12 receptor |
| RJ610_18495 | WND79273.1 | 1,92 | 6,38E-08 | 0,22 | 2,44E-03 | PKD domain-containing protein |
| RJ610_19960 | WND79545.1 | 0,88 | 2,92E-01 | 0,22 | 1,09E-02 | serine hydroxymethyltransferase |
| RJ610_04820 | WND81695.1 | 2,99 | 2,70E-28 | 0,22 | 1,19E-02 | OmpW family outer membrane protein |
| RJ610_04025 | WND81555.1 | 1,35 | 7,89E-02 | 0,22 | 1,00E+00 | co-chaperone GroES |
| RJ610_05810 | WND81881.1 | 2,29 | 2,31E-12 | 0,21 | 1,09E-04 | RHS repeat-associated core domain-containing protein |
| RJ610_16945 | WND78982.1 | 0,48 | 6,71E-06 | 0,21 | 3,94E-03 | cellulase family glycosylhydrolase |
| RJ610_03995 | WND81549.1 | 3,15 | 4,10E-13 | 0,20 | 2,37E-03 | endonuclease |
| RJ610_09425 | WND82547.1 | 0,71 | 1,94E-03 | 0,20 | 8,24E-03 | M14 family metallopeptidase |
| RJ610_17170 | WND79024.1 | 0,49 | 7,61E-07 | 0,20 | 1,50E-03 | DUF885 domain-containing protein |
| RJ610_08540 | WND82386.1 | 1,21 | 1,26E-01 | 0,18 | 7,15E-02 | enoyl-CoA hydratase-related protein |
| RJ610_05740 | WND81867.1 | 0,70 | 3,78E-02 | 0,17 | 1,00E+00 | dodecin family protein |
| RJ610_13605 | WND78349.1 | 1,39 | 1,03E-02 | 0,17 | 1,00E+00 | peptidoglycan-binding protein |
| RJ610_04090 | WND81567.1 | 0,94 | 6,52E-01 | 0,17 | 5,41E-05 | YceI family protein |
| RJ610_21975 | WND79921.1 | 2,27 | 3,12E-14 | 0,17 | 1,00E+00 | lipid A deacylase LpxR family protein |
| RJ610_06385 | WND81985.1 | 0,44 | 2,47E-17 | 0,17 | 2,60E-04 | hypothetical protein |
| RJ610_23875 | WND80278.1 | 1,89 | 2,33E-06 | 0,17 | 1,85E-02 | acetate--CoA ligase |
| RJ610_22670 | WND83337.1 | 1,62 | 6,58E-04 | 0,16 | 3,78E-03 | TIM barrel protein |
| RJ610_04580 | WND81651.1 | 0,75 | 1,63E-02 | 0,16 | 4,99E-03 | RidA family protein |
| RJ610_12970 | WND78228.1 | 0,29 | 2,61E-12 | 0,16 | 3,09E-05 | right-handed parallel beta-helix repeat-containing protein |
| RJ610_06120 | WND81940.1 | 6,28 | | 0,14 | 3,55E-02 | cysteine synthase A |
| RJ610_04945 | WND81719.1 | 0,77 | 9,91E-02 | 0,14 | 3,55E-02 | cysteine synthase A |
| RJ610_09565 | WND82575.1 | 0,59 | 3,58E-05 | 0,14 | 1,00E+00 | ferredoxin--NADP reductase |
| RJ610_19390 | WND79443.1 | 0,61 | 1,15E-06 | 0,14 | 1,23E-04 | Tol-Pal system beta propeller repeat protein TolB |
| RJ610_01945 | WND81158.1 | 0,40 | 4,68E-20 | 0,14 | 3,94E-05 | glycosyl hydrolase family 28-related protein |
| RJ610_00905 | WND80966.1 | 12,04 | 6,80E-09 | 0,13 | 3,23E-04 | copper chaperone PCu(A)C |
| RJ610_18485 | WND79271.1 | 0,97 | 8,47E-01 | 0,13 | 1,33E-04 | prolyl aminopeptidase |
| RJ610_01170 | WND81019.1 | 1,17 | 1,66E-01 | 0,13 | 5,52E-05 | hypothetical protein |
| RJ610_08050 | WND82299.1 | 0,97 | 8,57E-01 | 0,13 | 7,94E-04 | prolyl aminopeptidase |
| RJ610_13280 | WND78287.1 | 1,31 | 8,05E-02 | 0,13 | 7,46E-04 | YceI family protein |
| RJ610_13170 | WND78268.1 | 1,06 | 5,88E-01 | 0,13 | 8,32E-02 | 2-oxoglutarate dehydrogenase E1 component |
| RJ610_20070 | WND79564.1 | 0,25 | 1,26E-38 | 0,13 | 1,28E-04 | hypothetical protein |

| | | | | | | |
|---|---|---|---|---|---|---|
| RJ610_07095 | WND82123.1 | 1,37 | 1,66E-02 | 0,12 | 2,96E-04 | S9 family peptidase |
| RJ610_17495 | WND79087.1 | 0,20 | 1,14E-30 | 0,12 | 1,69E-03 | outer membrane beta-barrel protein |
| RJ610_07905 | WND82275.1 | 0,68 | 2,22E-03 | 0,12 | 3,09E-02 | PQQ-dependent sugar dehydrogenase |
| RJ610_00385 | WND80868.1 | 0,64 | 1,68E-03 | 0,11 | 2,32E-04 | ABC transporter substrate-binding protein |
| RJ610_04925 | WND81715.1 | 1,19 | 7,31E-02 | 0,11 | 1,00E+00 | class I fructose-bisphosphate aldolase |
| RJ610_23675 | WND80238.1 | 6,95 | 2,96E-26 | 0,11 | 4,50E-02 | porphobilinogen synthase |
| RJ610_16100 | WND78818.1 | 0,32 | 1,07E-13 | 0,10 | 5,01E-03 | hypothetical protein |
| RJ610_05980 | WND81912.1 | 1,08 | 6,21E-01 | 0,10 | 2,91E-03 | DUF3108 domain-containing protein |
| RJ610_16890 | WND78971.1 | 1,01 | 9,24E-01 | 0,10 | 1,35E-03 | phosphopyruvate hydratase |
| RJ610_14495 | WND78518.1 | 0,75 | 1,86E-02 | 0,09 | 5,51E-03 | TonB-dependent siderophore receptor |
| RJ610_11160 | WND82860.1 | 1,26 | 3,41E-01 | 0,09 | 2,37E-04 | molecular chaperone DnaK |
| RJ610_21265 | WND79790.1 | 0,23 | 9,81E-38 | 0,07 | 9,08E-05 | delta-60 repeat domain-containing protein |
| RJ610_20560 | WND83309.1 | 1,05 | 9,48E-01 | 0,06 | 4,29E-03 | homogentisate 1,2-dioxygenase |
| RJ610_15740 | WND78754.1 | 7,99 | 8,25E-48 | 0,06 | 8,17E-04 | Do family serine endopeptidase |
| RJ610_23445 | WND80196.1 | 0,23 | 4,98E-21 | 0,06 | 6,37E-03 | DUF885 domain-containing protein |
| RJ610_26300 | WND80748.1 | 0,59 | 1,10E-06 | 0,06 | 4,99E-04 | NADP-dependent isocitrate dehydrogenase |
| RJ610_12820 | WND78199.1 | 0,52 | 1,16E-06 | 0,06 | 5,27E-04 | TldD/PmbA family protein |
| RJ610_12825 | WND78200.1 | 0,60 | 9,12E-05 | 0,06 | 8,02E-05 | TldD/PmbA family protein |
| RJ610_05290 | WND81782.1 | 1,16 | 3,56E-01 | 0,06 | 1,11E-04 | hypothetical protein |
| RJ610_21765 | WND79881.1 | 0,58 | 1,24E-05 | 0,06 | 1,00E+00 | prolyl oligopeptidase family serine peptidase |
| RJ610_20365 | WND79621.1 | 0,99 | 9,71E-01 | 0,05 | 1,41E-03 | peptidoglycan DD-metalloendopeptidase family protein |
| RJ610_04445 | WND81628.1 | 0,60 | 2,80E-05 | 0,05 | 9,30E-04 | hypothetical protein |
| RJ610_03460 | WND81449.1 | 0,90 | 4,16E-01 | 0,04 | 2,25E-02 | dihydrolipoyl dehydrogenase |
| RJ610_04630 | WND81661.1 | 0,80 | 3,53E-02 | 0,04 | 1,49E-02 | citrate synthase |
| RJ610_12980 | WND78230.1 | 0,48 | 3,01E-08 | 0,04 | 2,77E-04 | hypothetical protein |
| RJ610_17635 | WND79115.1 | 1,54 | 3,42E-04 | 0,03 | 1,00E+00 | M66 family metalloprotease |
| RJ610_01980 | WND81165.1 | 1,14 | 4,56E-01 | 0,03 | 2,84E-04 | M28 family metallopeptidase |
| RJ610_06250 | WND81961.1 | 0,67 | 6,37E-04 | 0,03 | 3,24E-05 | DUF2271 domain-containing protein |
| RJ610_21680 | WND79866.1 | 1,44 | 1,08E-01 | 0,02 | 1,00E+00 | SDR family oxidoreductase |
| RJ610_14455 | WND78510.1 | 0,67 | 1,82E-02 | 0,02 | 6,17E-03 | M13 family metallopeptidase |
| RJ610_22135 | WND79948.1 | 0,98 | 9,07E-01 | 0,02 | 3,68E-03 | Gfo/Idh/MocA family oxidoreductase |
| RJ610_07800 | WND82254.1 | 0,47 | 2,37E-09 | 0,02 | 3,16E-04 | M13-type metalloendopeptidase |
| RJ610_21165 | WND79771.1 | 0,81 | 1,24E-01 | 0,02 | 5,53E-04 | M28 family metallopeptidase |
| RJ610_18505 | WND79275.1 | 1,32 | 1,45E-01 | 0,02 | 3,52E-03 | peroxiredoxin |
| RJ610_05240 | WND81772.1 | 0,09 | 1,43E-136 | 0,02 | 5,35E-05 | TonB-dependent receptor |
| RJ610_00895 | WND80964.1 | 2,30 | 4,14E-12 | 0,01 | 3,27E-04 | glycoside hydrolase family 3 N-terminal domain-containing protein |
| RJ610_18500 | WND79274.1 | 1,58 | 1,21E-02 | 0,01 | 1,00E+00 | carboxymuconolactone decarboxylase family protein |
| RJ610_20330 | WND79614.1 | 0,84 | 7,69E-02 | 0,01 | 3,33E-05 | pyridoxal-phosphate dependent enzyme |
| RJ610_04275 | WND81602.1 | 0,54 | 1,25E-09 | 0,01 | 8,60E-05 | inorganic diphosphatase |
| RJ610_05660 | WND81852.1 | 0,89 | 4,75E-01 | 0,01 | 2,38E-05 | YceI family protein |
| RJ610_17410 | WND79070.1 | 0,20 | 3,28E-18 | 0,01 | 4,00E-05 | DUF885 domain-containing protein |
| RJ610_17720 | WND79132.1 | 0,05 | 1,98E-55 | 0,01 | 1,17E-03 | TonB-dependent receptor |
| RJ610_09525 | WND82567.1 | 0,69 | 2,82E-02 | 0,01 | 1,22E-04 | M3 family metallopeptidase |

| | | | | | | |
|---|---|---|---|---|---|---|
| RJ610_17815 | WND79149.1 | 1,15 | 2,78E-01 | 0,01 | 2,49E-05 | malate dehydrogenase |
| RJ610_15540 | WND78718.1 | 0,20 | 1,44E-67 | 0,01 | 1,44E-05 | S8 family serine peptidase |
| RJ610_17630 | WND79114.1 | 1,26 | 8,13E-02 | 0,01 | 8,21E-04 | S46 family peptidase |
| RJ610_19380 | WND79441.1 | 0,62 | 1,12E-04 | 0,00 | 7,39E-05 | tol-pal system protein YbgF |
| RJ610_00005 | WND80794.1 | 0,75 | 1,02E-02 | | | chromosomal replication initiator protein DnaA |
| RJ610_00015 | WND80796.1 | 0,76 | 2,01E-02 | | | DNA replication/repair protein RecF |
| RJ610_00020 | WND80797.1 | 1,02 | 8,60E-01 | | | DNA topoisomerase (ATP-hydrolyzing) subunit B |
| RJ610_00025 | WND80798.1 | 0,95 | 6,40E-01 | | | type II CAAX endopeptidase family protein |
| RJ610_00030 | WND80799.1 | 0,47 | 5,77E-09 | | | M48 family metallopeptidase |
| RJ610_00035 | WND80800.1 | 0,85 | 8,34E-02 | | | tetratricopeptide repeat protein |
| RJ610_00040 | WND83358.1 | 0,73 | 6,20E-03 | | | energy transducer TonB |
| RJ610_00045 | WND80801.1 | 0,71 | 8,73E-05 | | | MotA/TolQ/ExbB proton channel family protein |
| RJ610_00050 | WND80802.1 | 0,74 | 1,08E-03 | | | biopolymer transporter ExbD |
| RJ610_00055 | WND80803.1 | 0,73 | 3,59E-04 | | | biopolymer transporter ExbD |
| RJ610_00060 | WND80804.1 | 0,09 | 1,24E-91 | | | biopolymer transporter ExbD |
| RJ610_00070 | WND80806.1 | 1,78 | 3,66E-08 | | | hypothetical protein |
| RJ610_00075 | WND80807.1 | 0,51 | 2,50E-08 | | | cardiolipin synthase |
| RJ610_00080 | WND80808.1 | 0,40 | 4,41E-11 | | | SIMPL domain-containing protein |
| RJ610_00085 | WND80809.1 | 0,36 | 2,58E-13 | | | PA0069 family radical SAM protein |
| RJ610_00090 | WND80810.1 | 0,59 | 1,22E-05 | | | hypothetical protein |
| RJ610_00095 | WND80811.1 | 0,64 | 4,23E-03 | | | MFS transporter |
| RJ610_00100 | WND80812.1 | 1,14 | 3,21E-01 | | | LysR family transcriptional regulator |
| RJ610_00105 | WND80813.1 | 3,36 | 9,70E-25 | | | helix-turn-helix domain-containing protein |
| RJ610_00110 | WND80814.1 | 0,31 | 2,89E-11 | | | lysozyme inhibitor LprI family protein |
| RJ610_00115 | WND80815.1 | 0,35 | 9,23E-12 | | | hypothetical protein |
| RJ610_00120 | WND80816.1 | 0,18 | 1,05E-14 | | | lysozyme inhibitor LprI family protein |
| RJ610_00125 | WND80817.1 | 0,23 | 1,24E-02 | | | hypothetical protein |
| RJ610_00130 | WND80818.1 | 1,61 | 8,63E-03 | | | M4 family metallopeptidase |
| RJ610_00135 | WND80819.1 | 0,88 | 6,36E-01 | | | hypothetical protein |
| RJ610_00140 | WND80820.1 | 0,34 | 2,15E-07 | | | hypothetical protein |
| RJ610_00145 | WND80821.1 | 0,27 | 2,62E-26 | | | SLC13 family permease |
| RJ610_00150 | WND80822.1 | 0,48 | 7,14E-09 | | | PAS domain S-box protein |
| RJ610_00155 | WND80823.1 | 0,60 | 3,07E-05 | | | response regulator |
| RJ610_00160 | WND80824.1 | 0,50 | 2,77E-12 | | | 2OG-Fe(II) oxygenase |
| RJ610_00165 | WND80825.1 | 0,11 | 5,63E-82 | | | hypothetical protein |
| RJ610_00170 | WND80826.1 | 1,56 | 5,38E-03 | | | sigma-54 dependent transcriptional regulator |
| RJ610_00175 | WND80827.1 | 0,79 | 8,20E-02 | | | class 1 fructose-bisphosphatase |
| RJ610_00180 | WND80828.1 | 1,21 | 1,65E-01 | | | hypothetical protein |
| RJ610_00185 | WND80829.1 | 0,87 | 6,80E-01 | | | hypothetical protein |
| RJ610_00190 | WND80830.1 | 1,67 | 2,94E-02 | | | hypothetical protein |
| RJ610_00195 | WND80831.1 | 1,88 | 4,25E-05 | | | DNA polymerase IV |
| RJ610_00200 | WND80832.1 | 7,38 | 3,99E-14 | | | GNAT family N-acetyltransferase |
| RJ610_00205 | WND80833.1 | 0,91 | 5,76E-01 | | | response regulator transcription factor |

| | | | | | | |
|---|---|---|---|---|---|---|
| RJ610_00210 | WND80834.1 | 0,70 | 1,37E-02 | | | ATP-binding protein |
| RJ610_00215 | WND80835.1 | 0,50 | 1,85E-03 | | | hypothetical protein |
| RJ610_00220 | WND80836.1 | 0,40 | 4,02E-08 | | | NIPSNAP family protein |
| RJ610_00225 | WND80837.1 | 0,73 | 4,27E-02 | | | YafY family protein |
| RJ610_00230 | WND80838.1 | 0,97 | 8,82E-01 | | | RNA polymerase sigma factor SigJ |
| RJ610_00235 | WND80839.1 | 0,56 | 5,30E-04 | | | carboxymuconolactone decarboxylase family protein |
| RJ610_00240 | WND80840.1 | 0,26 | 1,42E-16 | | | response regulator transcription factor |
| RJ610_00245 | WND80841.1 | 0,22 | 7,35E-29 | | | histidine kinase |
| RJ610_00250 | WND80842.1 | 0,13 | 1,52E-27 | | | hypothetical protein |
| RJ610_00255 | WND80843.1 | 0,50 | 6,18E-08 | | | alpha/beta hydrolase |
| RJ610_00260 | WND80844.1 | 1,28 | 3,39E-01 | | | FAD/NAD(P)-binding protein |
| RJ610_00265 | WND80845.1 | 0,26 | 1,16E-10 | | | hypothetical protein |
| RJ610_00270 | WND83359.1 | 0,54 | 1,03E-02 | | | DUF4291 domain-containing protein |
| RJ610_00275 | WND80846.1 | 0,73 | 5,74E-01 | | | phenylalanine 4-monooxygenase |
| RJ610_00280 | WND80847.1 | 0,72 | 2,94E-02 | | | Lrp/AsnC family transcriptional regulator |
| RJ610_00285 | WND80848.1 | 0,67 | 4,45E-03 | | | cytochrome c oxidase assembly factor Coa1 family protein |
| RJ610_00290 | WND80849.1 | 0,58 | 4,69E-05 | | | aspartate/glutamate racemase family protein |
| RJ610_00295 | WND80850.1 | 0,90 | 5,73E-01 | | | M28 family peptidase |
| RJ610_00300 | WND80851.1 | 0,41 | 2,90E-06 | | | phosphoethanolamine transferase |
| RJ610_00305 | WND80852.1 | 0,15 | 1,20E-43 | | | protein kinase |
| RJ610_00310 | WND80853.1 | 0,58 | 1,16E-05 | | | ECF-type sigma factor |
| RJ610_00315 | WND80854.1 | 0,75 | 2,46E-01 | | | hypothetical protein |
| RJ610_00320 | WND80855.1 | 0,61 | 4,16E-03 | | | hypothetical protein |
| RJ610_00325 | WND80856.1 | 0,74 | 1,24E-03 | | | transporter |
| RJ610_00330 | WND80857.1 | 0,83 | 9,12E-02 | | | hypothetical protein |
| RJ610_00335 | WND80858.1 | 2,03 | 6,64E-07 | | | rhomboid family intramembrane serine protease |
| RJ610_00340 | WND80859.1 | 0,98 | 8,13E-01 | | | class I SAM-dependent rRNA methyltransferase |
| RJ610_00345 | WND80860.1 | 0,64 | 3,72E-05 | | | hypothetical protein |
| RJ610_00350 | WND80861.1 | 0,63 | 7,68E-06 | | | hypothetical protein |
| RJ610_00355 | WND80862.1 | 0,53 | 1,81E-08 | | | hypothetical protein |
| RJ610_00360 | WND80863.1 | 0,43 | 1,03E-09 | | | DNA recombination protein RmuC |
| RJ610_00365 | WND80864.1 | 0,52 | 6,00E-04 | | | hypothetical protein |
| RJ610_00370 | WND80865.1 | 0,73 | 1,05E-01 | | | YrhB domain-containing protein |
| RJ610_00380 | WND80867.1 | 0,78 | 1,24E-01 | | | STAS domain-containing protein |
| RJ610_00390 | WND80869.1 | 0,68 | 3,25E-03 | | | outer membrane lipid asymmetry maintenance protein MlaD |
| RJ610_00395 | WND80870.1 | 0,79 | 2,25E-02 | | | MlaE family lipid ABC transporter permease subunit |
| RJ610_00400 | WND80871.1 | 0,68 | 2,15E-04 | | | ABC transporter ATP-binding protein |
| RJ610_00405 | WND80872.1 | 0,68 | 1,83E-03 | | | exodeoxyribonuclease V subunit gamma |
| RJ610_00410 | WND80873.1 | 0,73 | 1,32E-02 | | | exodeoxyribonuclease V subunit beta |
| RJ610_00415 | WND80874.1 | 0,85 | 2,06E-01 | | | exodeoxyribonuclease V subunit alpha |
| RJ610_00420 | | 1,25 | 3,54E-02 | | | LysR family transcriptional regulator |
| RJ610_00425 | WND80875.1 | 2,33 | 1,43E-09 | | | DUF2798 domain-containing protein |
| RJ610_00430 | WND80876.1 | 2,58 | 3,74E-20 | | | SDR family oxidoreductase |

| | | | | | | |
|---|---|---|---|---|---|---|
| RJ610_00435 | WND80877.1 | 2,47 | 1,09E-13 | | | efflux RND transporter periplasmic adaptor subunit |
| RJ610_00440 | WND80878.1 | 2,82 | 1,05E-22 | | | multidrug efflux RND transporter permease subunit |
| RJ610_00450 | WND80880.1 | 1,42 | 2,61E-03 | | | ketosteroid isomerase-related protein |
| RJ610_00455 | WND80881.1 | 68,44 | 2,24E-232 | | | MHYT domain-containing protein |
| RJ610_00460 | WND80882.1 | 61,58 | 3,93E-246 | | | 4-vinyl reductase |
| RJ610_00465 | WND80883.1 | 1,82 | 5,47E-05 | | | pyridoxal-phosphate dependent enzyme |
| RJ610_00470 | WND80884.1 | 2,06 | 1,48E-10 | | | ABC transporter permease |
| RJ610_00475 | WND80885.1 | 1,70 | 1,01E-04 | | | hypothetical protein |
| RJ610_00480 | WND80886.1 | 1,74 | 2,55E-07 | | | HlyD family efflux transporter periplasmic adaptor subunit |
| RJ610_00485 | WND80887.1 | 1,52 | 2,88E-03 | | | HlyD family efflux transporter periplasmic adaptor subunit |
| RJ610_00490 | WND80888.1 | 2,20 | 4,23E-09 | | | ABC transporter ATP-binding protein |
| RJ610_00495 | WND80889.1 | 10,30 | 5,65E-84 | | | hypothetical protein |
| RJ610_00500 | WND80890.1 | 0,95 | 7,56E-01 | | | hypothetical protein |
| RJ610_00505 | WND80891.1 | 1,31 | 6,59E-03 | | | tRNA uridine-5-carboxymethylaminomethyl(34) synthesis enzyme MnmG |
| RJ610_00510 | WND83360.1 | 1,03 | 8,48E-01 | | | glucan biosynthesis protein D |
| RJ610_00515 | WND80892.1 | 0,55 | 1,72E-08 | | | NAD(P)/FAD-dependent oxidoreductase |
| RJ610_00520 | WND80893.1 | 0,29 | 7,23E-19 | | | serine hydrolase domain-containing protein |
| RJ610_00530 | WND80895.1 | 0,43 | 5,39E-09 | | | 3'-5' exonuclease |
| RJ610_00535 | WND80896.1 | 0,58 | 6,59E-04 | | | putative quinol monooxygenase |
| RJ610_00540 | WND80897.1 | 0,61 | 1,84E-04 | | | S41 family peptidase |
| RJ610_00545 | WND80898.1 | 0,54 | 4,36E-02 | | | cytochrome b/b6 domain-containing protein |
| RJ610_00550 | WND80899.1 | 0,68 | 9,88E-02 | | | catalase family peroxidase |
| RJ610_00555 | WND80900.1 | 0,48 | 5,27E-06 | | | sigma-70 family RNA polymerase sigma factor |
| RJ610_00560 | WND80901.1 | 0,75 | 6,23E-02 | | | anti-sigma factor |
| RJ610_00565 | WND80902.1 | 0,80 | 1,07E-01 | | | hypothetical protein |
| RJ610_00570 | WND80903.1 | 1,07 | 6,11E-01 | | | indolepyruvate ferredoxin oxidoreductase family protein |
| RJ610_00575 | WND80904.1 | 0,52 | 1,36E-05 | | | hypothetical protein |
| RJ610_00580 | WND80905.1 | 0,32 | 1,53E-16 | | | hypothetical protein |
| RJ610_00585 | WND80906.1 | 0,79 | 3,81E-02 | | | macro domain-containing protein |
| RJ610_00590 | WND80907.1 | 0,51 | 5,74E-07 | | | hypothetical protein |
| RJ610_00595 | WND80908.1 | 0,48 | 3,36E-08 | | | hypothetical protein |
| RJ610_00600 | WND80909.1 | 0,81 | 8,24E-02 | | | amidohydrolase family protein |
| RJ610_00610 | WND83361.1 | 0,55 | 9,60E-10 | | | SIS domain-containing protein |
| RJ610_00615 | WND80911.1 | 0,68 | 5,95E-04 | | | GntR family transcriptional regulator |
| RJ610_00620 | WND80912.1 | 0,54 | 1,54E-08 | | | glucose-6-phosphate dehydrogenase |
| RJ610_00625 | WND80913.1 | 0,71 | 4,33E-04 | | | 6-phosphogluconolactonase |
| RJ610_00630 | WND80914.1 | 0,61 | 1,43E-08 | | | phosphogluconate dehydratase |
| RJ610_00635 | WND80915.1 | 0,67 | 2,24E-06 | | | HAD family hydrolase |
| RJ610_00645 | WND80917.1 | 0,69 | 5,96E-05 | | | LacI family DNA-binding transcriptional regulator |
| RJ610_00650 | WND80918.1 | 0,32 | 1,02E-22 | | | hotdog fold thioesterase |
| RJ610_00655 | WND80919.1 | 0,02 | 7,17E-121 | | | hypothetical protein |
| RJ610_00660 | WND80920.1 | 0,43 | 2,30E-06 | | | hypothetical protein |

| | | | | | | |
|---|---|---|---|---|---|---|
| RJ610_00665 | WND80921.1 | 0,65 | 8,77E-05 | | | phospholipase D family protein |
| RJ610_00670 | WND80922.1 | 0,74 | 4,33E-02 | | | YceI family protein |
| RJ610_00675 | WND80923.1 | 0,62 | 1,05E-04 | | | histidine phosphatase family protein |
| RJ610_00680 | WND80924.1 | 0,67 | 3,59E-03 | | | ParA family protein |
| RJ610_00685 | WND80925.1 | 1,98 | 2,85E-01 | | | hypothetical protein |
| RJ610_00690 | WND80926.1 | 0,68 | 1,09E-03 | | | hypothetical protein |
| RJ610_00695 | WND80927.1 | 0,45 | 4,96E-05 | | | AEC family transporter |
| RJ610_00700 | WND80928.1 | 0,49 | 8,67E-04 | | | AAA family ATPase |
| RJ610_00705 | WND80929.1 | 0,54 | 1,44E-02 | | | gamma-glutamylcyclotransferase family protein |
| RJ610_00710 | WND80930.1 | 0,56 | 2,07E-03 | | | hypothetical protein |
| RJ610_00715 | WND80931.1 | 0,54 | 3,67E-05 | | | gamma-glutamyltransferase |
| RJ610_00720 | WND80932.1 | 1,05 | 8,07E-01 | | | DUF2974 domain-containing protein |
| RJ610_00725 | WND80933.1 | 0,66 | 3,58E-02 | | | ankyrin repeat domain-containing protein |
| RJ610_00730 | WND80934.1 | 1,02 | 9,82E-01 | | | hypothetical protein |
| RJ610_00735 | WND80935.1 | 0,69 | 2,44E-03 | | | hypothetical protein |
| RJ610_00740 | WND80936.1 | 1,73 | 2,15E-06 | | | ankyrin repeat domain-containing protein |
| RJ610_00745 | WND80937.1 | 1,51 | 4,40E-04 | | | ATP-binding protein |
| RJ610_00750 | WND80938.1 | 1,35 | 4,76E-02 | | | EAL domain-containing response regulator |
| RJ610_00755 | WND80939.1 | 0,21 | 5,94E-18 | | | tetratricopeptide repeat protein |
| RJ610_00760 | WND80940.1 | 1,97 | 1,32E-06 | | | family 20 glycosylhydrolase |
| RJ610_00770 | WND80942.1 | 1,92 | 1,31E-10 | | | glucokinase |
| RJ610_00775 | WND80943.1 | 1,34 | 3,04E-03 | | | sugar MFS transporter |
| RJ610_00780 | WND80944.1 | 0,57 | 1,34E-06 | | | lysophospholipid acyltransferase family protein |
| RJ610_00785 | WND83362.1 | 0,71 | 5,35E-03 | | | alpha/beta fold hydrolase |
| RJ610_00790 | WND80945.1 | 0,61 | 5,10E-05 | | | hypothetical protein |
| RJ610_00795 | WND80946.1 | 0,44 | 1,18E-14 | | | tetratricopeptide repeat protein |
| RJ610_00800 | WND80947.1 | 0,53 | 3,77E-07 | | | YbhB/YbcL family Raf kinase inhibitor-like protein |
| RJ610_00805 | WND80948.1 | 0,55 | 4,59E-06 | | | SDR family oxidoreductase |
| RJ610_00810 | WND80949.1 | 0,52 | 6,68E-09 | | | RDD family protein |
| RJ610_00815 | WND80950.1 | 0,67 | 1,08E-02 | | | RDD family protein |
| RJ610_00820 | WND80951.1 | 0,69 | 4,03E-02 | | | RDD family protein |
| RJ610_00825 | WND80952.1 | 1,03 | 8,74E-01 | | | hypothetical protein |
| RJ610_00830 | WND80953.1 | 1,25 | 9,05E-02 | | | hypothetical protein |
| RJ610_00840 | WND80955.1 | 1,07 | 6,66E-01 | | | DUF2750 domain-containing protein |
| RJ610_00845 | WND80956.1 | 1,05 | 6,79E-01 | | | undecaprenyl-diphosphate phosphatase |
| RJ610_00850 | WND80957.1 | 1,82 | 2,58E-03 | | | type I glutamate--ammonia ligase |
| RJ610_00855 | WND80958.1 | 5,60 | 8,98E-17 | | | TorF family putative porin |
| RJ610_00860 | WND80959.1 | 5,38 | 1,95E-22 | | | P-II family nitrogen regulator |
| RJ610_00865 | WND83363.1 | 5,08 | 4,38E-14 | | | ammonium transporter |
| RJ610_00870 | | 4,28 | 8,27E-10 | | | ammonia channel protein |
| RJ610_00875 | WND80960.1 | 2,23 | 5,86E-08 | | | 2OG-Fe(II) oxygenase |
| RJ610_00880 | WND80961.1 | 461,25 | 1,98E-218 | | | S8 family peptidase |
| RJ610_00885 | WND80962.1 | 2,21 | 1,18E-13 | | | sodium/sugar symporter |

| | | | | | | |
|---|---|---|---|---|---|---|
| RJ610_00890 | WND80963.1 | 1,41 | 1,61E-04 | | | LacI family DNA-binding transcriptional regulator |
| RJ610_00900 | WND80965.1 | 1,87 | 6,57E-06 | | | response regulator |
| RJ610_00910 | WND80967.1 | 11,76 | 6,35E-09 | | | SCO family protein |
| RJ610_00915 | WND80968.1 | 1,09 | 6,20E-01 | | | hypothetical protein |
| RJ610_00920 | WND80969.1 | 1,45 | 3,15E-03 | | | hypothetical protein |
| RJ610_00925 | WND80970.1 | 2,44 | 8,95E-17 | | | type III secretion system export apparatus subunit SctU |
| RJ610_00930 | WND80971.1 | 1,59 | 1,46E-03 | | | type III secretion system export apparatus subunit SctT |
| RJ610_00935 | WND80972.1 | 1,12 | 5,21E-01 | | | type III secretion system export apparatus subunit SctS |
| RJ610_00940 | WND80973.1 | 1,23 | 1,51E-01 | | | EscR/YscR/HrcR family type III secretion system export apparatus protein |
| RJ610_00945 | WND80974.1 | 1,15 | 3,19E-01 | | | type III secretion system cytoplasmic ring protein SctQ |
| RJ610_00950 | WND80975.1 | 1,30 | 8,29E-02 | | | response regulator |
| RJ610_00955 | WND80976.1 | 1,37 | 3,55E-02 | | | ATP-binding protein |
| RJ610_00960 | WND80977.1 | 1,94 | 2,04E-11 | | | flagellar biosynthetic protein FliO |
| RJ610_00965 | WND80978.1 | 1,91 | 6,11E-07 | | | FHIPEP family type III secretion protein |
| RJ610_00970 | WND80979.1 | 1,36 | 1,45E-02 | | | FHA domain-containing protein |
| RJ610_00975 | WND80980.1 | 1,29 | 3,75E-02 | | | hypothetical protein |
| RJ610_00980 | WND80981.1 | 1,43 | 4,16E-03 | | | hypothetical protein |
| RJ610_00985 | WND80982.1 | 1,29 | 8,16E-02 | | | type III secretion inner membrane ring lipoprotein SctJ |
| RJ610_00990 | WND80983.1 | 1,28 | 6,31E-02 | | | hypothetical protein |
| RJ610_00995 | WND80984.1 | 1,04 | 7,78E-01 | | | FliH/SctL family protein |
| RJ610_01000 | WND80985.1 | 1,46 | 2,19E-03 | | | FliI/YscN family ATPase |
| RJ610_01005 | WND80986.1 | 1,48 | 1,78E-03 | | | hypothetical protein |
| RJ610_01010 | WND80987.1 | 1,72 | 2,81E-05 | | | hypothetical protein |
| RJ610_01015 | WND80988.1 | 1,25 | 2,15E-01 | | | hypothetical protein |
| RJ610_01020 | WND80989.1 | 1,18 | 1,66E-01 | | | EscF/YscF/HrpA family type III secretion system needle major subunit |
| RJ610_01025 | WND80990.1 | 1,56 | 1,43E-04 | | | hypothetical protein |
| RJ610_01030 | WND80991.1 | 2,17 | 2,32E-05 | | | hypothetical protein |
| RJ610_01035 | WND80992.1 | 0,49 | 4,95E-07 | | | MFS transporter |
| RJ610_01040 | WND80993.1 | 0,82 | 2,43E-01 | | | hypothetical protein |
| RJ610_01045 | WND80994.1 | 0,04 | 2,01E-153 | | | glycosyltransferase family 1 protein |
| RJ610_01050 | WND80995.1 | 0,25 | 1,97E-35 | | | metalloregulator ArsR/SmtB family transcription factor |
| RJ610_01055 | WND80996.1 | 0,55 | 4,73E-07 | | | hypothetical protein |
| RJ610_01065 | WND80998.1 | 4,37 | 2,23E-05 | | | YciI family protein |
| RJ610_01070 | WND80999.1 | 3,06 | 2,46E-04 | | | VOC family protein |
| RJ610_01075 | WND81000.1 | 0,77 | 3,67E-02 | | | RNA polymerase sigma factor |
| RJ610_01080 | WND81001.1 | 0,84 | 6,70E-01 | | | MgtC/SapB family protein |
| RJ610_01085 | WND81002.1 | 1,05 | 9,10E-01 | | | nitrate/sulfonate/bicarbonate ABC transporter ATP-binding protein |
| RJ610_01090 | WND81003.1 | 1,24 | 5,35E-01 | | | ABC transporter permease subunit |
| RJ610_01095 | WND81004.1 | 0,21 | 4,32E-04 | | | hypothetical protein |
| RJ610_01100 | WND81005.1 | 0,90 | 5,36E-01 | | | hypothetical protein |
| RJ610_01105 | WND81006.1 | 0,75 | 2,32E-02 | | | TetR/AcrR family transcriptional regulator |

| | | | | | | |
|---|---|---|---|---|---|---|
| RJ610_01110 | WND81007.1 | 0,75 | 3,10E-02 | | | hypothetical protein |
| RJ610_01115 | WND81008.1 | 1,02 | 9,12E-01 | | | multidrug efflux RND transporter permease subunit |
| RJ610_01120 | WND81009.1 | 0,82 | 3,10E-01 | | | hypothetical protein |
| RJ610_01125 | WND81010.1 | 4,81 | 1,17E-21 | | | multicopper oxidase domain-containing protein |
| RJ610_01130 | WND81011.1 | 8,16 | 4,11E-41 | | | FKBP-type peptidyl-prolyl cis-trans isomerase |
| RJ610_01135 | WND81012.1 | 5,89 | 4,56E-14 | | | hypothetical protein |
| RJ610_01145 | WND81014.1 | 3,17 | 7,08E-09 | | | hypothetical protein |
| RJ610_01150 | WND81015.1 | 2,07 | 4,29E-09 | | | ankyrin repeat domain-containing protein |
| RJ610_01155 | WND81016.1 | 1,79 | 2,82E-07 | | | TerC family protein |
| RJ610_01160 | WND81017.1 | 1,63 | 5,72E-04 | | | hypothetical protein |
| RJ610_01165 | WND81018.1 | 1,60 | 4,99E-04 | | | DNA alkylation repair protein |
| RJ610_01175 | WND81020.1 | 1,05 | 7,75E-01 | | | NAD(P)H-dependent oxidoreductase |
| RJ610_01180 | WND83364.1 | 0,88 | 4,51E-01 | | | isoaspartyl peptidase/L-asparaginase |
| RJ610_01185 | WND81021.1 | 0,51 | 2,25E-02 | | | cyanophycinase |
| RJ610_01190 | WND81022.1 | 0,34 | 4,84E-02 | | | hypothetical protein |
| RJ610_01195 | WND81023.1 | 0,40 | 9,37E-09 | | | sigma-70 family RNA polymerase sigma factor |
| RJ610_01200 | WND81024.1 | 0,53 | 6,11E-05 | | | FecR domain-containing protein |
| RJ610_01205 | WND81025.1 | 0,88 | 4,37E-01 | | | TonB-dependent receptor |
| RJ610_01210 | WND81026.1 | 0,87 | 4,67E-01 | | | trans-aconitate 2-methyltransferase |
| RJ610_01215 | WND81027.1 | 0,12 | 3,57E-39 | | | VCBS repeat-containing protein |
| RJ610_01220 | WND81028.1 | 0,33 | 7,13E-10 | | | NAD(P)-dependent oxidoreductase |
| RJ610_01225 | WND81029.1 | 0,78 | 1,94E-01 | | | hypothetical protein |
| RJ610_01230 | WND81030.1 | 1,21 | 1,55E-01 | | | hypothetical protein |
| RJ610_01235 | WND81031.1 | 0,81 | 6,47E-02 | | | hypothetical protein |
| RJ610_01240 | WND81032.1 | 0,62 | 4,93E-05 | | | hypothetical protein |
| RJ610_01245 | WND81033.1 | 0,74 | 1,57E-02 | | | hypothetical protein |
| RJ610_01250 | WND81034.1 | 0,77 | 1,38E-01 | | | hypothetical protein |
| RJ610_01255 | WND81035.1 | 1,45 | 5,76E-03 | | | hypothetical protein |
| RJ610_01260 | WND81036.1 | 0,79 | 1,05E-01 | | | hypothetical protein |
| RJ610_01265 | WND81037.1 | 1,55 | 6,11E-04 | | | ATP-binding protein |
| RJ610_01270 | WND81038.1 | 1,45 | 2,07E-04 | | | nitrogen regulation protein NR(I) |
| RJ610_01280 | WND81040.1 | 1,26 | 1,21E-01 | | | GNAT family N-acetyltransferase |
| RJ610_01285 | WND81041.1 | 0,83 | 1,29E-01 | | | hypothetical protein |
| RJ610_01290 | WND81042.1 | 0,69 | 2,48E-03 | | | hypothetical protein |
| RJ610_01295 | WND81043.1 | 0,77 | 1,02E-02 | | | acetyl-CoA C-acetyltransferase |
| RJ610_01300 | WND81044.1 | 2,19 | 4,24E-07 | | | sulfatase-like hydrolase/transferase |
| RJ610_01305 | WND81045.1 | 0,27 | 2,21E-28 | | | hypothetical protein |
| RJ610_01310 | WND81046.1 | 0,73 | 3,21E-03 | | | hypothetical protein |
| RJ610_01315 | WND81047.1 | 0,90 | 3,81E-01 | | | hypothetical protein |
| RJ610_01325 | WND81049.1 | 0,97 | 9,18E-01 | | | hypothetical protein |
| RJ610_01330 | WND81050.1 | 0,45 | 2,55E-11 | | | heme biosynthesis HemY N-terminal domain-containing protein |
| RJ610_01335 | WND81051.1 | 0,53 | 4,02E-07 | | | uroporphyrinogen-III C-methyltransferase |
| RJ610_01340 | WND81052.1 | 2,39 | 1,61E-07 | | | uroporphyrinogen-III synthase |

| | | | | | | |
|---|---|---|---|---|---|---|
| RJ610_01345 | WND81053.1 | 0,74 | 3,88E-02 | | | YiiD C-terminal domain-containing protein |
| RJ610_01350 | WND81054.1 | 0,63 | 5,52E-06 | | | hypothetical protein |
| RJ610_01355 | WND81055.1 | 0,69 | 1,16E-03 | | | rhodanese-like domain-containing protein |
| RJ610_01360 | WND81056.1 | 0,98 | 9,08E-01 | | | protein-export chaperone SecB |
| RJ610_01365 | WND81057.1 | 0,98 | 9,12E-01 | | | NAD(P)H-dependent glycerol-3-phosphate dehydrogenase |
| RJ610_01375 | WND81058.1 | 1,04 | 8,44E-01 | | | 2OG-Fe(II) oxygenase |
| RJ610_01380 | WND81059.1 | 2,20 | 7,78E-03 | | | peptidoglycan-binding domain-containing protein |
| RJ610_01385 | WND81060.1 | 2,35 | 1,63E-06 | | | TonB-dependent siderophore receptor |
| RJ610_01390 | WND81061.1 | 2,67 | 2,05E-03 | | | ABC transporter ATP-binding protein |
| RJ610_01395 | WND81062.1 | 2,54 | 1,20E-02 | | | DUF3526 domain-containing protein |
| RJ610_01400 | WND81063.1 | 1,97 | 8,47E-02 | | | DUF3526 domain-containing protein |
| RJ610_01405 | WND81064.1 | 1,98 | 1,58E-02 | | | TonB-dependent receptor |
| RJ610_01410 | WND81065.1 | 1,62 | 1,22E-01 | | | hypothetical protein |
| RJ610_01415 | WND81066.1 | 1,35 | 1,35E-01 | | | pyridoxal-phosphate dependent enzyme |
| RJ610_01420 | WND81067.1 | 2,05 | 8,06E-05 | | | ATP-grasp domain-containing protein |
| RJ610_01425 | WND81068.1 | 1,30 | 2,97E-01 | | | DUF364 domain-containing protein |
| RJ610_01430 | WND81069.1 | 0,69 | 5,12E-03 | | | MFS transporter |
| RJ610_01435 | WND81070.1 | 0,71 | 1,44E-03 | | | tRNA (uridine(34)/cytosine(34)/5-carboxymethylaminomethyluridine(34)-2'-O)-methyltransferase TrmL |
| RJ610_01440 | WND81071.1 | 1,18 | 1,67E-01 | | | pitrilysin family protein |
| RJ610_01445 | | 0,87 | 2,01E-01 | | | DUF4156 domain-containing protein |
| RJ610_01450 | WND81072.1 | 0,51 | 5,70E-14 | | | 3-oxoacyl-ACP synthase III |
| RJ610_01455 | WND81073.1 | 0,60 | 2,19E-05 | | | hypothetical protein |
| RJ610_01460 | WND81074.1 | 0,57 | 1,92E-07 | | | DUF2170 family protein |
| RJ610_01465 | WND81075.1 | 0,54 | 2,28E-07 | | | PspA/IM30 family protein |
| RJ610_01470 | WND81076.1 | 0,55 | 3,80E-04 | | | hypothetical protein |
| RJ610_01475 | WND81077.1 | 0,42 | 5,84E-14 | | | flotillin family protein |
| RJ610_01480 | WND81078.1 | 0,45 | 4,21E-10 | | | DNA repair ATPase |
| RJ610_01485 | WND81079.1 | 0,45 | 9,35E-09 | | | phospholipase D-like domain-containing protein |
| RJ610_01490 | WND81080.1 | 0,53 | 2,92E-05 | | | CoA transferase |
| RJ610_01495 | WND81081.1 | 1,08 | 6,17E-01 | | | glycoside hydrolase family 127 protein |
| RJ610_01500 | WND81082.1 | 1,04 | 8,10E-01 | | | SUMF1/EgtB/PvdO family nonheme iron enzyme |
| RJ610_01505 | WND81083.1 | 0,61 | 7,31E-02 | | | ribokinase |
| RJ610_01510 | WND83366.1 | 0,70 | 1,25E-01 | | | serine hydrolase domain-containing protein |
| RJ610_01515 | WND81084.1 | 0,93 | 5,91E-01 | | | TonB-dependent receptor |
| RJ610_01520 | WND81085.1 | 0,72 | 4,21E-03 | | | LysR family transcriptional regulator |
| RJ610_01525 | WND81086.1 | 0,63 | 5,57E-03 | | | sodium:solute symporter |
| RJ610_01530 | WND81087.1 | 0,46 | 3,63E-05 | | | CoA ester lyase |
| RJ610_01535 | WND81088.1 | 0,59 | 2,97E-07 | | | alpha/beta fold hydrolase |
| RJ610_01540 | WND81089.1 | 0,59 | 1,65E-05 | | | fatty acid CoA ligase family protein |
| RJ610_01545 | WND81090.1 | 0,76 | 3,56E-02 | | | 2-alkyl-3-oxoalkanoate reductase |
| RJ610_01550 | WND81091.1 | 0,33 | 9,73E-11 | | | DUF1328 domain-containing protein |

| | | | | | | |
|---|---|---|---|---|---|---|
| RJ610_01555 | WND81092.1 | 0,70 | 5,78E-04 | | | SCP2 sterol-binding domain-containing protein |
| RJ610_01560 | WND81093.1 | 0,69 | 2,59E-03 | | | ubiquinone biosynthesis regulatory protein kinase UbiB |
| RJ610_01565 | WND81094.1 | 0,98 | 9,06E-01 | | | pitrilysin family protein |
| RJ610_01570 | WND81095.1 | 0,56 | 5,78E-08 | | | pseudouridine synthase |
| RJ610_01575 | WND81096.1 | 0,37 | 4,19E-26 | | | DUF2059 domain-containing protein |
| RJ610_01580 | WND81097.1 | 1,40 | 3,85E-03 | | | hypothetical protein |
| RJ610_01585 | WND81098.1 | 0,52 | 1,65E-07 | | | alternative ribosome rescue aminoacyl-tRNA hydrolase ArfB |
| RJ610_01590 | WND81099.1 | 0,60 | 2,18E-06 | | | 1-acyl-sn-glycerol-3-phosphate acyltransferase |
| RJ610_01595 | WND81100.1 | 1,00 | 9,99E-01 | | | hypothetical protein |
| RJ610_01600 | WND81101.1 | 1,11 | 4,32E-01 | | | GntR family transcriptional regulator |
| RJ610_01605 | WND81102.1 | 1,31 | 8,70E-02 | | | collagenase |
| RJ610_01610 | WND83367.1 | 0,73 | 1,45E-02 | | | LysR family transcriptional regulator |
| RJ610_01615 | WND81103.1 | 0,13 | 4,85E-33 | | | malate synthase A |
| RJ610_01625 | WND81105.1 | 0,49 | 1,82E-07 | | | S9 family peptidase |
| RJ610_01630 | WND83368.1 | 0,57 | 2,19E-07 | | | GGDEF domain-containing protein |
| RJ610_01635 | WND81106.1 | 0,35 | 2,15E-29 | | | putative peptide modification system cyclase |
| RJ610_01640 | WND81107.1 | 0,31 | 1,96E-32 | | | NHLP-related RiPP peptide |
| RJ610_01645 | WND81108.1 | 0,55 | 6,82E-04 | | | putative peptide maturation dehydrogenase |
| RJ610_01650 | WND81109.1 | 1,17 | 3,58E-01 | | | nitronate monooxygenase |
| RJ610_01655 | WND81110.1 | 0,94 | 8,47E-01 | | | alpha/beta hydrolase |
| RJ610_01660 | WND81111.1 | 0,62 | 4,59E-05 | | | alpha/beta hydrolase |
| RJ610_01665 | WND81112.1 | 0,47 | 2,47E-08 | | | hypothetical protein |
| RJ610_01670 | WND81113.1 | 1,21 | 2,19E-01 | | | AraC family transcriptional regulator |
| RJ610_01675 | WND81114.1 | 17,42 | 8,09E-32 | | | GNAT family N-acetyltransferase |
| RJ610_01680 | WND81115.1 | 7,72 | 8,62E-15 | | | FAD-binding oxidoreductase |
| RJ610_01685 | | 0,79 | 1,10E-01 | | | ATP-binding protein |
| RJ610_01690 | WND81116.1 | 1,04 | 8,47E-01 | | | hypothetical protein |
| RJ610_01695 | WND81117.1 | 0,79 | 6,78E-02 | | | hypothetical protein |
| RJ610_01700 | WND81118.1 | 0,80 | 7,91E-02 | | | DUF4238 domain-containing protein |
| RJ610_01705 | WND81119.1 | 1,16 | 1,65E-01 | | | hypothetical protein |
| RJ610_01710 | WND81120.1 | 1,15 | 1,46E-01 | | | dsDNA nuclease domain-containing protein |
| RJ610_01715 | WND81121.1 | 0,88 | 3,36E-01 | | | hypothetical protein |
| RJ610_01720 | WND81122.1 | 0,67 | 2,35E-03 | | | hypothetical protein |
| RJ610_01725 | WND81123.1 | 1,55 | 4,40E-03 | | | hypothetical protein |
| RJ610_01730 | WND81124.1 | 0,91 | 4,58E-01 | | | hypothetical protein |
| RJ610_01735 | WND81125.1 | 1,06 | 6,77E-01 | | | nucleotidyl transferase AbiEii/AbiGii toxin family protein |
| RJ610_01740 | WND81126.1 | 1,14 | 2,55E-01 | | | helix-turn-helix transcriptional regulator |
| RJ610_01745 | WND81127.1 | 1,45 | 2,95E-03 | | | hypothetical protein |
| RJ610_01750 | WND81128.1 | 1,03 | 8,65E-01 | | | hypothetical protein |
| RJ610_01755 | WND81129.1 | 0,99 | 9,63E-01 | | | SseB family protein |
| RJ610_01760 | WND81130.1 | 1,01 | 9,48E-01 | | | GIY-YIG nuclease family protein |
| RJ610_01765 | WND81131.1 | 1,21 | 1,05E-01 | | | hypothetical protein |
| RJ610_01770 | WND81132.1 | 0,87 | 4,72E-01 | | | helix-turn-helix transcriptional regulator |

| | | | | | | |
|---|---|---|---|---|---|---|
| RJ610_01775 | WND81133.1 | 0,80 | 8,66E-02 | | | tyrosine-type recombinase/integrase |
| RJ610_01780 | | 0,37 | 2,95E-14 | | | YifB family Mg chelatase-like AAA ATPase |
| RJ610_01785 | WND81134.1 | 0,40 | 1,05E-11 | | | accessory factor UbiK family protein |
| RJ610_01790 | WND81135.1 | 0,69 | 1,08E-01 | | | P-II family nitrogen regulator |
| RJ610_01795 | WND81136.1 | 0,75 | 6,66E-02 | | | LysR family transcriptional regulator |
| RJ610_01800 | WND83369.1 | 1,42 | 8,48E-02 | | | YeiH family protein |
| RJ610_01820 | WND81140.1 | 0,63 | 5,49E-03 | | | YciI family protein |
| RJ610_01825 | WND81141.1 | 1,12 | 4,00E-01 | | | NAD(P)-dependent oxidoreductase |
| RJ610_01830 | WND81142.1 | 1,56 | 2,19E-01 | | | hypothetical protein |
| RJ610_01835 | WND81143.1 | 4,48 | 1,25E-20 | | | hypothetical protein |
| RJ610_01840 | WND81144.1 | 0,53 | 3,10E-10 | | | SPOR domain-containing protein |
| RJ610_01845 | WND81145.1 | 0,44 | 2,60E-21 | | | arginine--tRNA ligase |
| RJ610_01850 | WND81146.1 | 0,71 | 2,40E-01 | | | DNA repair protein RadC |
| RJ610_01855 | | 0,55 | 4,17E-06 | | | bifunctional phosphopantothenoylcysteine decarboxylase/phosphopantothenate--cysteine ligase CoaBC |
| RJ610_01860 | WND81147.1 | 0,55 | 1,04E-03 | | | hypothetical protein |
| RJ610_01875 | WND81150.1 | 2,02 | 1,38E-09 | | | hypothetical protein |
| RJ610_01880 | WND81151.1 | 0,80 | 5,21E-02 | | | orotate phosphoribosyltransferase |
| RJ610_01885 | WND81152.1 | 0,68 | 2,08E-03 | | | GNAT family N-acetyltransferase |
| RJ610_01890 | WND81153.1 | 0,61 | 1,84E-06 | | | exodeoxyribonuclease III |
| RJ610_01895 | WND83370.1 | 0,75 | 9,63E-03 | | | MFS transporter |
| RJ610_01900 | WND81154.1 | 0,91 | 4,49E-01 | | | hypothetical protein |
| RJ610_01905 | WND81155.1 | 0,80 | 9,31E-02 | | | anhydro-N-acetylmuramic acid kinase |
| RJ610_01910 | WND81156.1 | 1,42 | 1,98E-04 | | | M23 family metallopeptidase |
| RJ610_01915 | WND81157.1 | 1,29 | 7,00E-03 | | | tyrosine--tRNA ligase |
| RJ610_01940 | | 1,44 | 6,75E-01 | | | 5S ribosomal RNA |
| RJ610_01950 | WND81159.1 | 0,77 | 9,04E-02 | | | hypothetical protein |
| RJ610_01955 | WND81160.1 | 0,82 | 1,92E-01 | | | HAMP domain-containing sensor histidine kinase |
| RJ610_01960 | WND81161.1 | 0,74 | 4,65E-02 | | | response regulator transcription factor |
| RJ610_01965 | WND81162.1 | 0,61 | 9,51E-03 | | | DJ-1/PfpI family protein |
| RJ610_01970 | WND81163.1 | 0,65 | 1,81E-03 | | | 2OG-Fe dioxygenase family protein |
| RJ610_01975 | WND81164.1 | 0,53 | 2,35E-06 | | | MAPEG family protein |
| RJ610_01985 | WND81166.1 | 1,51 | 4,36E-02 | | | hypothetical protein |
| RJ610_01990 | WND81167.1 | 0,84 | 8,56E-02 | | | 2,3-bisphosphoglycerate-independent phosphoglycerate mutase |
| RJ610_01995 | WND81168.1 | 1,02 | 9,07E-01 | | | peptidoglycan DD-metalloendopeptidase family protein |
| RJ610_02000 | WND81169.1 | 1,12 | 3,15E-01 | | | S41 family peptidase |
| RJ610_02005 | WND81170.1 | 0,77 | 1,85E-01 | | | hypothetical protein |
| RJ610_02010 | WND81171.1 | 0,85 | 4,81E-01 | | | DUF1820 family protein |
| RJ610_02015 | WND81172.1 | 0,78 | 2,48E-02 | | | rhomboid family intramembrane serine protease |
| RJ610_02025 | WND81174.1 | 0,97 | 8,96E-01 | | | class I SAM-dependent methyltransferase |
| RJ610_02030 | WND81175.1 | 1,09 | 7,26E-01 | | | adenylosuccinate lyase family protein |
| RJ610_02035 | WND81176.1 | 1,36 | 1,98E-01 | | | adenylosuccinate lyase |
| RJ610_02040 | WND81177.1 | 1,79 | 6,28E-02 | | | HOASN domain-containing protein |

| | | | | | | |
|---|---|---|---|---|---|---|
| RJ610_02045 | WND81178.1 | 1,54 | 4,34E-02 | | | HOASN domain-containing protein |
| RJ610_02050 | WND81179.1 | 4,64 | 4,43E-06 | | | YHS domain-containing protein |
| RJ610_02055 | WND81180.1 | 7,50 | 1,37E-11 | | | heavy metal-responsive transcriptional regulator |
| RJ610_02060 | WND81181.1 | 2,93 | 2,22E-10 | | | MOSC domain-containing protein |
| RJ610_02065 | WND81182.1 | 3,01 | 3,13E-19 | | | DUF2164 domain-containing protein |
| RJ610_02070 | WND81183.1 | 7,54 | 1,29E-55 | | | DUF3574 domain-containing protein |
| RJ610_02075 | WND81184.1 | 0,88 | 2,29E-01 | | | bifunctional proline dehydrogenase/L-glutamate gamma-semialdehyde dehydrogenase PutA |
| RJ610_02085 | WND81186.1 | 1,19 | 8,88E-02 | | | cytochrome c oxidase subunit I |
| RJ610_02090 | WND81187.1 | 1,16 | 3,45E-01 | | | hypothetical protein |
| RJ610_02095 | WND81188.1 | 1,40 | 4,17E-03 | | | cytochrome c oxidase assembly protein |
| RJ610_02100 | WND81189.1 | 1,33 | 1,61E-02 | | | cytochrome c oxidase subunit 3 |
| RJ610_02105 | WND81190.1 | 5,24 | 1,89E-20 | | | twin transmembrane helix small protein |
| RJ610_02110 | WND81191.1 | 5,63 | 1,94E-15 | | | SURF1 family protein |
| RJ610_02115 | WND81192.1 | 4,70 | 3,21E-12 | | | hypothetical protein |
| RJ610_02120 | WND83155.1 | 3,04 | 3,36E-09 | | | COX15/CtaA family protein |
| RJ610_02125 | WND81193.1 | 3,34 | 1,43E-09 | | | heme o synthase |
| RJ610_02130 | WND81194.1 | 4,24 | 1,31E-14 | | | amidohydrolase family protein |
| RJ610_02135 | WND81195.1 | 2,21 | 2,76E-11 | | | bile acid:sodium symporter family protein |
| RJ610_02140 | WND81196.1 | 4,08 | 3,36E-53 | | | hypothetical protein |
| RJ610_02145 | WND81197.1 | 1,38 | 7,32E-03 | | | DNA/RNA non-specific endonuclease |
| RJ610_02150 | WND81198.1 | 0,79 | 6,50E-02 | | | hypothetical protein |
| RJ610_02155 | WND81199.1 | 0,52 | 2,09E-05 | | | S9 family peptidase |
| RJ610_02160 | WND81200.1 | 0,50 | 5,29E-07 | | | membrane dipeptidase |
| RJ610_02165 | WND81201.1 | 0,55 | 9,46E-10 | | | DNA primase |
| RJ610_02170 | WND81202.1 | 1,94 | 4,34E-08 | | | glutathione S-transferase family protein |
| RJ610_02175 | WND81203.1 | 2,35 | 5,49E-15 | | | YihY/virulence factor BrkB family protein |
| RJ610_02180 | WND81204.1 | 0,96 | 8,09E-01 | | | SEC-C metal-binding domain-containing protein |
| RJ610_02185 | WND81205.1 | 0,48 | 1,74E-09 | | | GatB/YqeY domain-containing protein |
| RJ610_02190 | WND81206.1 | 0,73 | 1,97E-02 | | | 30S ribosomal protein S21 |
| RJ610_02195 | WND81207.1 | 1,03 | 8,38E-01 | | | tRNA (adenosine(37)-N6)-threonylcarbamoyltransferase complex transferase subunit TsaD |
| RJ610_02200 | WND81208.1 | 0,94 | 6,36E-01 | | | dihydroneopterin aldolase |
| RJ610_02205 | WND81209.1 | 0,46 | 9,17E-11 | | | DUF6159 family protein |
| RJ610_02210 | WND81210.1 | 0,97 | 9,06E-01 | | | hypothetical protein |
| RJ610_02215 | WND81211.1 | 0,65 | 1,69E-04 | | | SAM-dependent methyltransferase |
| RJ610_02220 | WND81212.1 | 0,84 | 3,37E-01 | | | VanZ family protein |
| RJ610_02230 | WND81214.1 | 1,35 | 2,41E-02 | | | PAAR domain-containing protein |
| RJ610_02235 | WND81215.1 | 1,25 | 2,29E-01 | | | transglycosylase SLT domain-containing protein |
| RJ610_02240 | WND81216.1 | 1,17 | 3,04E-01 | | | hypothetical protein |
| RJ610_02245 | WND81217.1 | 0,78 | 2,20E-02 | | | multifunctional CCA addition/repair protein |
| RJ610_02250 | WND81218.1 | 0,87 | 3,13E-01 | | | PH domain-containing protein |
| RJ610_02255 | WND81219.1 | 1,80 | 2,99E-07 | | | complex I NDUFA9 subunit family protein |

| | | | | | | |
|---|---|---|---|---|---|---|
| RJ610_02260 | WND81220.1 | 1,56 | 1,87E-04 | | | transglycosylase SLT domain-containing protein |
| RJ610_02265 | WND81221.1 | 1,81 | 8,95E-05 | | | sulfite exporter TauE/SafE family protein |
| RJ610_02275 | WND81223.1 | 9,75 | 1,43E-54 | | | TonB-dependent receptor |
| RJ610_02280 | WND81224.1 | 1,08 | 6,45E-01 | | | ATP-grasp domain-containing protein |
| RJ610_02285 | WND81225.1 | 1,63 | 1,59E-07 | | | endonuclease/exonuclease/phosphatase family protein |
| RJ610_02295 | WND81227.1 | 2,86 | 9,64E-19 | | | thiol:disulfide interchange protein DsbA/DsbL |
| RJ610_02300 | WND81228.1 | 0,99 | 9,18E-01 | | | c-type cytochrome |
| RJ610_02305 | WND81229.1 | 0,92 | 4,76E-01 | | | ribosome biogenesis GTP-binding protein YihA/YsxC |
| RJ610_02310 | WND81230.1 | 0,85 | 3,26E-01 | | | tetratricopeptide repeat protein |
| RJ610_02315 | WND81231.1 | 0,49 | 3,24E-06 | | | DUF2007 domain-containing protein |
| RJ610_02320 | WND81232.1 | 0,55 | 3,26E-05 | | | alpha-amylase family glycosyl hydrolase |
| RJ610_02325 | WND81233.1 | 0,87 | 5,10E-01 | | | TonB-dependent receptor |
| RJ610_02330 | WND81234.1 | 1,26 | 2,01E-01 | | | MFS transporter |
| RJ610_02335 | WND81235.1 | 1,26 | 2,10E-01 | | | alpha-amylase family glycosyl hydrolase |
| RJ610_02340 | WND81236.1 | 0,74 | 1,59E-02 | | | LacI family DNA-binding transcriptional regulator |
| RJ610_02345 | WND81237.1 | 0,78 | 1,57E-01 | | | ABC transporter permease subunit |
| RJ610_02350 | WND81238.1 | 0,74 | 1,17E-01 | | | VIT family protein |
| RJ610_02355 | WND81239.1 | 0,73 | 3,75E-02 | | | peptide ABC transporter substrate-binding protein |
| RJ610_02360 | WND83156.1 | 1,07 | 6,77E-01 | | | glutamate--cysteine ligase |
| RJ610_02365 | WND81240.1 | 1,63 | 1,64E-03 | | | isochorismatase family cysteine hydrolase |
| RJ610_02370 | WND81241.1 | 2,53 | 3,34E-07 | | | DMT family transporter |
| RJ610_02375 | WND81242.1 | 0,73 | 2,31E-01 | | | LysR substrate-binding domain-containing protein |
| RJ610_02380 | WND81243.1 | 0,38 | 2,11E-07 | | | tetratricopeptide repeat protein |
| RJ610_02385 | WND81244.1 | 0,61 | 1,17E-02 | | | phosphoenolpyruvate carboxylase |
| RJ610_02390 | WND81245.1 | 1,23 | 5,52E-01 | | | formaldehyde-responsive transcriptional repressor FrmR |
| RJ610_02395 | WND83157.1 | 0,86 | 3,67E-01 | | | S-(hydroxymethyl)glutathione dehydrogenase/class III alcohol dehydrogenase |
| RJ610_02400 | WND81246.1 | 1,60 | 1,39E-02 | | | hypothetical protein |
| RJ610_02405 | WND81247.1 | 1,38 | 3,85E-03 | | | S-formylglutathione hydrolase |
| RJ610_02410 | WND81248.1 | 0,46 | 5,30E-04 | | | efflux RND transporter periplasmic adaptor subunit |
| RJ610_02415 | WND81249.1 | 0,43 | 7,88E-04 | | | efflux RND transporter permease subunit |
| RJ610_02420 | WND81250.1 | 0,47 | 1,13E-06 | | | hypothetical protein |
| RJ610_02425 | WND81251.1 | 0,38 | 3,04E-16 | | | hypothetical protein |
| RJ610_02430 | WND81252.1 | 1,74 | 7,33E-04 | | | AraC family transcriptional regulator |
| RJ610_02435 | WND81253.1 | 1,75 | 1,83E-02 | | | S41 family peptidase |
| RJ610_02440 | WND81254.1 | 0,94 | 7,75E-01 | | | helix-turn-helix domain-containing protein |
| RJ610_02445 | WND81255.1 | 0,13 | 2,81E-34 | | | S8 family serine peptidase |
| RJ610_02450 | WND81256.1 | 3,00 | 3,72E-04 | | | hypothetical protein |
| RJ610_02455 | WND81257.1 | 2,74 | 1,25E-07 | | | hypothetical protein |
| RJ610_02460 | WND81258.1 | 1,45 | 1,37E-01 | | | hypothetical protein |
| RJ610_02465 | WND81259.1 | 1,28 | 1,39E-01 | | | GMC family oxidoreductase |
| RJ610_02470 | WND81260.1 | 0,75 | 5,50E-02 | | | YafY family protein |
| RJ610_02475 | WND81261.1 | 0,98 | 8,57E-01 | | | GFA family protein |

| | | | | | | |
|---|---|---|---|---|---|---|
| RJ610_02480 | WND81262.1 | 0,73 | 1,52E-02 | | | MmcQ/YjbR family DNA-binding protein |
| RJ610_02485 | WND81263.1 | 0,77 | 4,82E-02 | | | PLP-dependent aminotransferase family protein |
| RJ610_02490 | WND81264.1 | 0,85 | 1,50E-01 | | | hypothetical protein |
| RJ610_02495 | WND81265.1 | 0,94 | 5,79E-01 | | | twin transmembrane helix small protein |
| RJ610_02500 | WND81266.1 | 1,03 | 8,15E-01 | | | hypothetical protein |
| RJ610_02505 | WND81267.1 | 1,17 | 1,84E-01 | | | cupin domain-containing protein |
| RJ610_02510 | WND81268.1 | 1,40 | 5,11E-03 | | | ADP-ribosylglycohydrolase family protein |
| RJ610_02515 | WND81269.1 | 0,92 | 6,31E-01 | | | hypothetical protein |
| RJ610_02520 | WND81270.1 | 0,40 | 3,84E-10 | | | amidohydrolase family protein |
| RJ610_02525 | WND81271.1 | 0,72 | 2,16E-02 | | | DegV family protein |
| RJ610_02530 | WND81272.1 | 1,02 | 8,62E-01 | | | 2-hydroxychromene-2-carboxylate isomerase |
| RJ610_02535 | WND81273.1 | 0,76 | 3,54E-02 | | | DUF1761 domain-containing protein |
| RJ610_02540 | WND81274.1 | 0,76 | 3,72E-02 | | | hypothetical protein |
| RJ610_02545 | WND81275.1 | 0,68 | 4,40E-02 | | | hypothetical protein |
| RJ610_02550 | WND81276.1 | 0,52 | 8,18E-08 | | | amidohydrolase family protein |
| RJ610_02555 | WND81277.1 | 0,46 | 2,33E-09 | | | amidohydrolase |
| RJ610_02560 | WND81278.1 | 1,36 | 4,61E-03 | | | AMP nucleosidase |
| RJ610_02565 | WND81279.1 | 4,27 | 5,68E-38 | | | lysozyme inhibitor LprI family protein |
| RJ610_02575 | WND81281.1 | 0,88 | 6,06E-01 | | | glutaredoxin domain-containing protein |
| RJ610_02580 | WND81282.1 | 0,72 | 1,72E-02 | | | polysaccharide deacetylase family protein |
| RJ610_02585 | WND81283.1 | 0,75 | 1,40E-02 | | | methyltransferase domain-containing protein |
| RJ610_02590 | WND81284.1 | 0,63 | 1,07E-04 | | | SGNH/GDSL hydrolase family protein |
| RJ610_02595 | WND81285.1 | 0,57 | 1,37E-09 | | | pyridoxal phosphate-dependent aminotransferase |
| RJ610_02600 | WND81286.1 | 0,61 | 9,13E-05 | | | ribosome small subunit-dependent GTPase A |
| RJ610_02605 | WND81287.1 | 0,86 | 2,90E-01 | | | flavohemoglobin expression-modulating QEGLA motif protein |
| RJ610_02610 | WND81288.1 | 0,86 | 5,34E-01 | | | acetoacetyl-CoA reductase |
| RJ610_02615 | WND81289.1 | 0,69 | 5,02E-02 | | | CitMHS family transporter |
| RJ610_02620 | WND81290.1 | 0,62 | 4,34E-02 | | | porin |
| RJ610_02625 | WND81291.1 | 1,01 | 9,37E-01 | | | response regulator transcription factor |
| RJ610_02630 | WND81292.1 | 0,90 | 5,44E-01 | | | sensor histidine kinase |
| RJ610_02635 | WND81293.1 | 0,80 | 2,49E-01 | | | ABC transporter substrate-binding protein |
| RJ610_02645 | WND81295.1 | 0,58 | 3,74E-07 | | | glycosyltransferase |
| RJ610_02650 | WND81296.1 | 0,62 | 1,41E-01 | | | flavodoxin family protein |
| RJ610_02655 | WND81297.1 | 0,59 | 2,63E-02 | | | LysR substrate-binding domain-containing protein |
| RJ610_02660 | WND81298.1 | 0,63 | 1,10E-02 | | | LysR family transcriptional regulator |
| RJ610_02665 | WND81299.1 | 0,68 | 1,26E-01 | | | glutathione transferase GstA |
| RJ610_02670 | WND81300.1 | 0,95 | 8,86E-01 | | | multidrug efflux SMR transporter |
| RJ610_02675 | WND81301.1 | 0,97 | 8,59E-01 | | | transposase |
| RJ610_02680 | WND81302.1 | 0,89 | 3,53E-01 | | | pyruvate dehydrogenase (acetyl-transferring), homodimeric type |
| RJ610_02685 | WND81303.1 | 0,89 | 5,52E-01 | | | hypothetical protein |
| RJ610_02690 | WND81304.1 | 0,45 | 3,74E-07 | | | hypothetical protein |
| RJ610_02695 | | 0,89 | 7,72E-01 | | | pyruvate dehydrogenase (acetyl-transferring), homodimeric type |
| RJ610_02700 | WND81305.1 | 0,97 | 8,75E-01 | | | hypothetical protein |

| | | | | | | |
|---|---|---|---|---|---|---|
| RJ610_02705 | WND81306.1 | 0,93 | 7,24E-01 | | | hypothetical protein |
| RJ610_02710 | WND81307.1 | 0,83 | 3,19E-01 | | | ATP-binding protein |
| RJ610_02715 | WND81308.1 | 0,93 | 6,30E-01 | | | hypothetical protein |
| RJ610_02720 | WND81309.1 | 1,17 | 3,00E-01 | | | helix-turn-helix transcriptional regulator |
| RJ610_02725 | WND81310.1 | 0,64 | 3,43E-03 | | | hypothetical protein |
| RJ610_02730 | WND81311.1 | 0,59 | 1,73E-04 | | | hypothetical protein |
| RJ610_02735 | WND81312.1 | 0,83 | 5,29E-01 | | | hypothetical protein |
| RJ610_02740 | WND81313.1 | 1,09 | 5,41E-01 | | | hypothetical protein |
| RJ610_02745 | WND81314.1 | 1,19 | 1,84E-01 | | | hypothetical protein |
| RJ610_02750 | WND81315.1 | 0,93 | 5,94E-01 | | | hypothetical protein |
| RJ610_02755 | WND81316.1 | 1,24 | 1,63E-01 | | | hypothetical protein |
| RJ610_02760 | WND81317.1 | 1,46 | 3,25E-03 | | | hypothetical protein |
| RJ610_02765 | WND81318.1 | 3,68 | 1,22E-13 | | | hypothetical protein |
| RJ610_02770 | WND81319.1 | 3,28 | 3,87E-13 | | | hypothetical protein |
| RJ610_02775 | WND81320.1 | 2,30 | 1,23E-02 | | | hypothetical protein |
| RJ610_02780 | WND81321.1 | 2,21 | 3,21E-08 | | | phytanoyl-CoA dioxygenase family protein |
| RJ610_02785 | WND83158.1 | 1,37 | 2,78E-02 | | | VOC family protein |
| RJ610_02790 | WND81322.1 | 1,83 | 4,10E-03 | | | hypothetical protein |
| RJ610_02795 | WND81323.1 | 2,36 | 1,36E-03 | | | FAD-dependent monooxygenase |
| RJ610_02805 | WND81325.1 | 1,93 | 4,84E-02 | | | VOC family protein |
| RJ610_02815 | WND81327.1 | 2,01 | 2,01E-04 | | | HAMP domain-containing sensor histidine kinase |
| RJ610_02820 | WND81328.1 | 1,31 | 1,25E-01 | | | response regulator transcription factor |
| RJ610_02825 | WND81329.1 | 12,96 | 3,99E-29 | | | hypothetical protein |
| RJ610_02830 | WND81330.1 | 1,12 | 5,70E-01 | | | DUF3348 domain-containing protein |
| RJ610_02835 | WND81331.1 | 0,99 | 9,71E-01 | | | DUF802 domain-containing protein |
| RJ610_02840 | WND81332.1 | 1,82 | 2,12E-02 | | | OmpA family protein |
| RJ610_02845 | WND81333.1 | 1,47 | 2,98E-02 | | | DUF2894 domain-containing protein |
| RJ610_02850 | WND81334.1 | 0,32 | 2,58E-14 | | | hypothetical protein |
| RJ610_02855 | WND81335.1 | 0,84 | 4,87E-01 | | | LytTR family DNA-binding domain-containing protein |
| RJ610_02860 | WND81336.1 | 0,85 | 3,56E-01 | | | histidine kinase |
| RJ610_02865 | WND81337.1 | 0,92 | 7,30E-01 | | | chromate efflux transporter |
| RJ610_02870 | WND81338.1 | 0,70 | 9,89E-03 | | | DUF4357 domain-containing protein |
| RJ610_02875 | WND81339.1 | 1,57 | 9,93E-05 | | | DUF6229 family protein |
| RJ610_02880 | WND81340.1 | 1,73 | 4,11E-03 | | | type 2 lanthipeptide synthetase LanM family protein |
| RJ610_02885 | WND81341.1 | 4,62 | 9,01E-10 | | | PHB depolymerase family esterase |
| RJ610_02890 | WND81342.1 | 5,59 | 9,12E-37 | | | MaoC family dehydratase |
| RJ610_02895 | WND81343.1 | 2,21 | 3,76E-15 | | | AraC family transcriptional regulator |
| RJ610_02900 | WND81344.1 | 2,32 | 1,39E-06 | | | TonB-dependent receptor |
| RJ610_02905 | WND81345.1 | 1,69 | 1,15E-01 | | | alpha/beta hydrolase |
| RJ610_02910 | WND81346.1 | 0,96 | 8,32E-01 | | | AMP-binding protein |
| RJ610_02915 | WND81347.1 | 0,60 | 8,06E-05 | | | hypothetical protein |
| RJ610_02920 | WND81348.1 | 1,43 | 3,07E-02 | | | M13 family metallopeptidase |
| RJ610_02925 | WND81349.1 | 5,33 | 1,85E-35 | | | type 2 lanthipeptide synthetase LanM family protein |

| | | | | | | |
|---|---|---|---|---|---|---|
| RJ610_02930 | WND81350.1 | 1,89 | 8,98E-07 | | | DUF6229 family protein |
| RJ610_02935 | WND81351.1 | 18,08 | 3,50E-44 | | | hypothetical protein |
| RJ610_02940 | WND81352.1 | 1,03 | 8,83E-01 | | | hypothetical protein |
| RJ610_02945 | WND81353.1 | 0,66 | 5,76E-03 | | | hypothetical protein |
| RJ610_02960 | WND81356.1 | 0,51 | 4,12E-08 | | | TonB-dependent receptor |
| RJ610_02965 | WND81357.1 | 1,43 | 1,11E-03 | | | FecR domain-containing protein |
| RJ610_02970 | WND81358.1 | 1,00 | 9,97E-01 | | | sigma-70 family RNA polymerase sigma factor |
| RJ610_02975 | WND81359.1 | 1,95 | 2,50E-10 | | | TetR/AcrR family transcriptional regulator |
| RJ610_02980 | WND81360.1 | 0,92 | 4,96E-01 | | | helix-turn-helix transcriptional regulator |
| RJ610_02985 | WND81361.1 | 0,76 | 1,11E-02 | | | hypothetical protein |
| RJ610_02990 | WND81362.1 | 1,08 | 5,65E-01 | | | hypothetical protein |
| RJ610_02995 | WND81363.1 | 1,36 | 8,49E-02 | | | hypothetical protein |
| RJ610_03000 | WND81364.1 | 1,83 | 2,10E-05 | | | fasciclin domain-containing protein |
| RJ610_03005 | WND83159.1 | 1,96 | 1,65E-06 | | | glucoamylase family protein |
| RJ610_03010 | WND81365.1 | 3,05 | 1,94E-06 | | | hypothetical protein |
| RJ610_03020 | WND81367.1 | 0,80 | 2,10E-01 | | | pirin family protein |
| RJ610_03025 | WND81368.1 | 0,77 | 8,36E-02 | | | OsmC family protein |
| RJ610_03030 | WND81369.1 | 0,44 | 1,42E-07 | | | hypothetical protein |
| RJ610_03035 | WND81370.1 | 0,35 | 6,21E-11 | | | hypothetical protein |
| RJ610_03040 | WND81371.1 | 0,61 | 9,66E-04 | | | PepSY domain-containing protein |
| RJ610_03045 | WND81372.1 | 0,75 | 1,38E-02 | | | ATP-grasp domain-containing protein |
| RJ610_03055 | WND81374.1 | 0,70 | 1,27E-04 | | | DUF3016 domain-containing protein |
| RJ610_03060 | WND81375.1 | 0,53 | 3,41E-02 | | | excalibur calcium-binding domain-containing protein |
| RJ610_03065 | WND81376.1 | 0,34 | 4,15E-12 | | | beta-propeller domain-containing protein |
| RJ610_03070 | WND81377.1 | 0,45 | 3,17E-08 | | | YciI family protein |
| RJ610_03075 | WND81378.1 | 0,59 | 2,32E-05 | | | tRNA (N6-threonylcarbamoyladenosine(37)-N6)-methyltransferase TrmO |
| RJ610_03080 | WND81379.1 | 1,09 | 6,46E-01 | | | SRPBCC domain-containing protein |
| RJ610_03085 | WND81380.1 | 1,52 | 6,57E-04 | | | ABC transporter ATP-binding protein |
| RJ610_03090 | WND81381.1 | 1,42 | 1,05E-02 | | | ABC transporter permease |
| RJ610_03095 | WND81382.1 | 0,83 | 1,45E-01 | | | amino acid permease |
| RJ610_03100 | WND83160.1 | 0,73 | 3,72E-02 | | | VWA domain-containing protein |
| RJ610_03105 | WND81383.1 | 0,83 | 1,48E-01 | | | tetratricopeptide repeat protein |
| RJ610_03110 | WND81384.1 | 0,65 | 2,97E-04 | | | DUF3297 family protein |
| RJ610_03115 | WND81385.1 | 0,91 | 6,06E-01 | | | SIMPL domain-containing protein |
| RJ610_03125 | WND81387.1 | 1,90 | 3,82E-07 | | | aldo/keto reductase |
| RJ610_03130 | WND81388.1 | 1,33 | 3,72E-02 | | | hypothetical protein |
| RJ610_03135 | WND81389.1 | 2,34 | 5,54E-15 | | | hypothetical protein |
| RJ610_03145 | WND81391.1 | 7,16 | 2,75E-30 | | | SpoIID/LytB domain-containing protein |
| RJ610_03155 | WND81393.1 | 1,08 | 6,10E-01 | | | HEAT repeat domain-containing protein |
| RJ610_03160 | WND81394.1 | 1,10 | 5,60E-01 | | | cold-shock protein |
| RJ610_03165 | WND81395.1 | 1,50 | 4,41E-03 | | | hypothetical protein |
| RJ610_03170 | WND81396.1 | 42,20 | 4,64E-114 | | | glycosyltransferase |

| | | | | | | |
|---|---|---|---|---|---|---|
| RJ610_03175 | WND81397.1 | 165,71 | 1,17E-174 | | | condensation domain-containing protein |
| RJ610_03185 | WND81399.1 | 1,48 | 1,73E-02 | | | MFS transporter |
| RJ610_03190 | WND81400.1 | 0,14 | 6,67E-51 | | | M48 family metallopeptidase |
| RJ610_03195 | WND81401.1 | 0,74 | 5,37E-02 | | | hypothetical protein |
| RJ610_03200 | WND81402.1 | 0,59 | 8,51E-05 | | | AAA domain-containing protein |
| RJ610_03205 | WND81403.1 | 1,16 | 5,04E-01 | | | DsbA family oxidoreductase |
| RJ610_03210 | WND81404.1 | 59,96 | 6,24E-121 | | | hypothetical protein |
| RJ610_03215 | WND81405.1 | 1,52 | 1,40E-03 | | | RHS repeat-associated core domain-containing protein |
| RJ610_03220 | WND81406.1 | 1,59 | 1,38E-03 | | | hypothetical protein |
| RJ610_03240 | WND81410.1 | 2,20 | 2,93E-09 | | | TetR/AcrR family transcriptional regulator |
| RJ610_03245 | WND81411.1 | 1,55 | 3,11E-03 | | | PLP-dependent aminotransferase family protein |
| RJ610_03250 | WND81412.1 | 5,75 | 1,08E-25 | | | DMT family transporter |
| RJ610_03255 | WND81413.1 | 107,85 | 1,67E-139 | | | hypothetical protein |
| RJ610_03260 | WND81414.1 | 0,69 | 2,68E-04 | | | RtcB family protein |
| RJ610_03265 | WND81415.1 | 0,66 | 1,30E-04 | | | hypothetical protein |
| RJ610_03270 | WND81416.1 | 0,42 | 5,22E-14 | | | slipin family protein |
| RJ610_03275 | | 0,77 | 2,94E-02 | | | tRNA-Leu |
| RJ610_03280 | | 0,74 | 9,45E-03 | | | tRNA-OTHER |
| RJ610_03285 | WND81417.1 | 0,75 | 4,70E-01 | | | cysteine hydrolase family protein |
| RJ610_03290 | WND81418.1 | 1,86 | 4,51E-02 | | | antibiotic biosynthesis monooxygenase |
| RJ610_03295 | WND81419.1 | 3,77 | 1,23E-19 | | | Lrp/AsnC family transcriptional regulator |
| RJ610_03300 | WND81420.1 | 3,23 | 3,72E-21 | | | polysaccharide deacetylase family protein |
| RJ610_03305 | WND81421.1 | 0,80 | 6,43E-02 | | | DUF6249 domain-containing protein |
| RJ610_03310 | WND81422.1 | 0,76 | 2,13E-02 | | | sigma-70 family RNA polymerase sigma factor |
| RJ610_03315 | WND81423.1 | 0,69 | 1,16E-02 | | | hypothetical protein |
| RJ610_03320 | WND81424.1 | 1,97 | 3,05E-01 | | | RNA polymerase subunit sigma-70 |
| RJ610_03325 | WND81425.1 | 1,61 | 1,46E-01 | | | RebB family R body protein |
| RJ610_03330 | WND83161.1 | 1,62 | 1,33E-01 | | | hypothetical protein |
| RJ610_03335 | WND81426.1 | 2,00 | 3,62E-05 | | | hypothetical protein |
| RJ610_03340 | WND81427.1 | 2,23 | 5,89E-02 | | | hypothetical protein |
| RJ610_03345 | WND81428.1 | 1,58 | 1,46E-02 | | | hypothetical protein |
| RJ610_03350 | WND81429.1 | 3,88 | 8,34E-16 | | | RebB family R body protein |
| RJ610_03355 | WND81430.1 | 4,99 | 2,92E-15 | | | RebB family R body protein |
| RJ610_03360 | WND81431.1 | 1,36 | 1,67E-01 | | | Crp/Fnr family transcriptional regulator |
| RJ610_03365 | WND81432.1 | 0,77 | 3,96E-02 | | | arylesterase |
| RJ610_03370 | WND83162.1 | 0,94 | 7,99E-01 | | | ABC transporter ATP-binding protein |
| RJ610_03375 | WND81433.1 | 1,03 | 8,87E-01 | | | FtsX-like permease family protein |
| RJ610_03380 | WND81434.1 | 0,51 | 3,28E-08 | | | hypothetical protein |
| RJ610_03385 | WND81435.1 | 0,47 | 1,55E-07 | | | hypothetical protein |
| RJ610_03390 | WND81436.1 | 0,78 | 2,74E-02 | | | class I SAM-dependent methyltransferase |
| RJ610_03395 | WND81437.1 | 0,85 | 1,82E-01 | | | pseudouridine synthase |
| RJ610_03400 | WND83163.1 | 0,91 | 4,66E-01 | | | HAD family phosphatase |
| RJ610_03405 | WND81438.1 | 0,70 | 1,85E-02 | | | DUF1415 domain-containing protein |

| | | | | | | |
|---|---|---|---|---|---|---|
| RJ610_03410 | WND81439.1 | 0,58 | 1,28E-05 | | | YajQ family cyclic di-GMP-binding protein |
| RJ610_03415 | WND81440.1 | 0,54 | 4,90E-06 | | | NAD(P)-dependent alcohol dehydrogenase |
| RJ610_03420 | WND81441.1 | 0,77 | 3,75E-02 | | | DUF1453 domain-containing protein |
| RJ610_03425 | WND81442.1 | 0,62 | 4,79E-04 | | | DMT family transporter |
| RJ610_03430 | WND81443.1 | 0,58 | 1,19E-02 | | | VOC family protein |
| RJ610_03440 | WND81445.1 | 1,16 | 5,97E-01 | | | hypothetical protein |
| RJ610_03445 | WND81446.1 | 0,09 | 1,86E-34 | | | DUF2884 family protein |
| RJ610_03450 | WND81447.1 | 1,08 | 4,81E-01 | | | dihydrolipoyllysine-residue acetyltransferase |
| RJ610_03455 | WND81448.1 | 0,97 | 8,60E-01 | | | hypothetical protein |
| RJ610_03465 | WND81450.1 | 1,52 | 6,33E-03 | | | hypothetical protein |
| RJ610_03475 | WND81451.1 | 0,58 | 7,49E-03 | | | DUF2061 domain-containing protein |
| RJ610_03480 | WND81452.1 | 1,62 | 5,34E-05 | | | hypothetical protein |
| RJ610_03485 | WND81453.1 | 1,16 | 3,22E-01 | | | F0F1 ATP synthase subunit A |
| RJ610_03490 | WND81454.1 | 1,26 | 9,32E-02 | | | F0F1 ATP synthase subunit C |
| RJ610_03495 | WND81455.1 | 1,34 | 2,25E-02 | | | F0F1 ATP synthase subunit B |
| RJ610_03500 | WND81456.1 | 1,54 | 3,36E-04 | | | F0F1 ATP synthase subunit delta |
| RJ610_03510 | WND81458.1 | 1,67 | 8,66E-06 | | | F0F1 ATP synthase subunit gamma |
| RJ610_03520 | WND81460.1 | 1,39 | 5,28E-03 | | | F0F1 ATP synthase subunit epsilon |
| RJ610_03525 | WND81461.1 | 0,90 | 5,83E-01 | | | GtrA family protein |
| RJ610_03530 | WND81462.1 | 0,69 | 1,82E-02 | | | glycosyltransferase |
| RJ610_03535 | WND81463.1 | 0,65 | 1,37E-03 | | | ADP-ribosylglycohydrolase family protein |
| RJ610_03540 | WND81464.1 | 0,65 | 3,60E-04 | | | glycosyltransferase 87 family protein |
| RJ610_03545 | WND81465.1 | 0,83 | 1,50E-01 | | | bifunctional UDP-N-acetylglucosamine diphosphorylase/glucosamine-1-phosphate N-acetyltransferase GlmU |
| RJ610_03555 | WND81467.1 | 0,35 | 6,11E-24 | | | glucose 1-dehydrogenase |
| RJ610_03560 | WND81468.1 | 0,68 | 1,10E-03 | | | helix-turn-helix domain-containing protein |
| RJ610_03565 | WND83165.1 | 0,53 | 2,90E-09 | | | ATP-binding protein |
| RJ610_03570 | WND81469.1 | 0,76 | 3,50E-02 | | | sigma-54 dependent transcriptional regulator |
| RJ610_03575 | WND81470.1 | 1,20 | 1,96E-01 | | | DUF885 family protein |
| RJ610_03580 | WND81471.1 | 0,70 | 3,22E-02 | | | FtsX-like permease family protein |
| RJ610_03585 | WND81472.1 | 1,25 | 1,19E-01 | | | ABC transporter permease |
| RJ610_03590 | WND81473.1 | 1,40 | 4,39E-02 | | | ABC transporter ATP-binding protein |
| RJ610_03595 | WND81474.1 | 0,73 | 1,82E-01 | | | hypothetical protein |
| RJ610_03600 | WND81475.1 | 0,93 | 7,87E-01 | | | efflux RND transporter periplasmic adaptor subunit |
| RJ610_03605 | WND81476.1 | 1,91 | 1,46E-10 | | | glutamine--fructose-6-phosphate transaminase (isomerizing) |
| RJ610_03610 | WND81477.1 | 0,46 | 1,09E-04 | | | SDR family oxidoreductase |
| RJ610_03615 | WND81478.1 | 0,74 | 1,17E-01 | | | class I SAM-dependent methyltransferase |
| RJ610_03620 | WND81479.1 | 0,70 | 5,01E-02 | | | TetR family transcriptional regulator |
| RJ610_03625 | WND81480.1 | 1,93 | 8,33E-05 | | | C1 family peptidase |
| RJ610_03630 | WND81481.1 | 0,97 | 7,84E-01 | | | hypothetical protein |
| RJ610_03635 | WND81482.1 | 0,84 | 3,42E-01 | | | NAD(P)H-binding protein |
| RJ610_03640 | WND81483.1 | 1,08 | 7,72E-01 | | | hypothetical protein |

| | | | | | | |
|---|---|---|---|---|---|---|
| RJ610_03645 | WND81484.1 | 1,31 | 3,59E-01 | | | hypothetical protein |
| RJ610_03650 | WND81485.1 | 0,71 | 2,39E-02 | | | S41 family peptidase |
| RJ610_03655 | WND81486.1 | 1,04 | 8,91E-01 | | | hypothetical protein |
| RJ610_03660 | WND81487.1 | 1,23 | 6,65E-01 | | | hypothetical protein |
| RJ610_03665 | WND81488.1 | 2,21 | 1,14E-01 | | | hypothetical protein |
| RJ610_03670 | WND81489.1 | 1,83 | 9,58E-05 | | | hypothetical protein |
| RJ610_03675 | WND81490.1 | 1,50 | 1,92E-02 | | | hypothetical protein |
| RJ610_03680 | WND81491.1 | 1,85 | 9,13E-04 | | | hypothetical protein |
| RJ610_03685 | WND81492.1 | 1,80 | 1,21E-01 | | | hypothetical protein |
| RJ610_03695 | WND81494.1 | 2,05 | 1,44E-12 | | | TfoX/Sxy family protein |
| RJ610_03700 | WND81495.1 | 1,91 | 8,91E-07 | | | GAF domain-containing protein |
| RJ610_03705 | WND81496.1 | 1,36 | 8,25E-03 | | | dethiobiotin synthase |
| RJ610_03710 | WND81497.1 | 0,22 | 1,03E-20 | | | phasin family protein |
| RJ610_03720 | | 1,21 | 2,25E-01 | | | tRNA-Met |
| RJ610_03725 | | 1,08 | 4,83E-01 | | | RNA pyrophosphohydrolase |
| RJ610_03730 | WND81499.1 | 0,47 | 1,66E-05 | | | (2Fe-2S)-binding protein |
| RJ610_03735 | WND81500.1 | 1,19 | 4,25E-01 | | | bacterioferritin |
| RJ610_03740 | WND81501.1 | 0,89 | 5,42E-01 | | | hypothetical protein |
| RJ610_03745 | WND81502.1 | 0,35 | 7,81E-14 | | | DUF4126 domain-containing protein |
| RJ610_03750 | WND83166.1 | 2,93 | 1,72E-19 | | | hypothetical protein |
| RJ610_03755 | WND83167.1 | 2,15 | 1,98E-12 | | | polymer-forming cytoskeletal protein |
| RJ610_03760 | WND81503.1 | 1,46 | 5,17E-02 | | | hypothetical protein |
| RJ610_03765 | WND81504.1 | 1,51 | 1,17E-02 | | | iron-sulfur cluster insertion protein ErpA |
| RJ610_03770 | WND81505.1 | 1,43 | 4,58E-02 | | | NAD(+) diphosphatase |
| RJ610_03775 | WND81506.1 | 0,73 | 1,00E-02 | | | regulatory signaling modulator protein AmpE |
| RJ610_03780 | WND81507.1 | 0,56 | 1,23E-06 | | | RNA polymerase sigma factor |
| RJ610_03785 | WND81508.1 | 0,58 | 3,25E-08 | | | FecR family protein |
| RJ610_03790 | WND81509.1 | 0,53 | 1,25E-12 | | | TonB-dependent receptor |
| RJ610_03795 | WND81510.1 | 0,41 | 2,99E-19 | | | DUF2752 domain-containing protein |
| RJ610_03800 | WND81511.1 | 0,29 | 1,80E-26 | | | CD225/dispanin family protein |
| RJ610_03805 | WND81512.1 | 1,36 | 9,98E-02 | | | MFS transporter |
| RJ610_03810 | WND81513.1 | 0,88 | 5,17E-01 | | | mechanosensitive ion channel |
| RJ610_03815 | WND81514.1 | 0,82 | 1,09E-01 | | | hypothetical protein |
| RJ610_03820 | WND81515.1 | 1,07 | 6,31E-01 | | | hypothetical protein |
| RJ610_03825 | WND81516.1 | 0,98 | 8,98E-01 | | | hypothetical protein |
| RJ610_03830 | WND81517.1 | 0,75 | 5,98E-02 | | | FAD-dependent monooxygenase |
| RJ610_03835 | WND81518.1 | 0,75 | 1,07E-02 | | | phosphoribosylamine--glycine ligase |
| RJ610_03840 | WND81519.1 | 0,56 | 9,08E-06 | | | alanine/glycine:cation symporter family protein |
| RJ610_03845 | WND81520.1 | 0,66 | 3,28E-03 | | | hypothetical protein |
| RJ610_03850 | WND81521.1 | 0,71 | 2,64E-03 | | | bifunctional phosphoribosylaminoimidazolecarboxamide formyltransferase/IMP cyclohydrolase |
| RJ610_03855 | WND81522.1 | 0,93 | 7,28E-01 | | | CDP-alcohol phosphatidyltransferase family protein |

| | | | | | | |
|---|---|---|---|---|---|---|
| RJ610_03860 | WND81523.1 | 0,93 | 7,21E-01 | | | bifunctional alpha/beta hydrolase/class I SAM-dependent methyltransferase |
| RJ610_03865 | WND81524.1 | 0,80 | 2,55E-01 | | | phosphatase PAP2/dual specificity phosphatase family protein |
| RJ610_03870 | WND81525.1 | 0,68 | 2,76E-01 | | | hypothetical protein |
| RJ610_03875 | WND81526.1 | 0,57 | 1,90E-04 | | | TIGR04222 domain-containing membrane protein |
| RJ610_03880 | WND81527.1 | 0,55 | 1,52E-03 | | | CDP-alcohol phosphatidyltransferase family protein |
| RJ610_03885 | WND81528.1 | 0,61 | 7,11E-04 | | | lysophospholipid acyltransferase family protein |
| RJ610_03890 | WND81529.1 | 0,51 | 2,61E-06 | | | phosphatidate cytidylyltransferase |
| RJ610_03895 | WND81530.1 | 0,94 | 7,10E-01 | | | tetratricopeptide repeat protein |
| RJ610_03900 | WND81531.1 | 1,02 | 9,10E-01 | | | hypothetical protein |
| RJ610_03905 | WND81532.1 | 1,02 | 8,77E-01 | | | DNA-binding transcriptional regulator Fis |
| RJ610_03910 | WND81533.1 | 2,24 | 9,83E-15 | | | DUF3426 domain-containing protein |
| RJ610_03915 | WND81534.1 | 0,87 | 3,46E-01 | | | 50S ribosomal protein L11 methyltransferase |
| RJ610_03920 | WND81535.1 | 0,89 | 4,80E-01 | | | hypothetical protein |
| RJ610_03925 | WND81536.1 | 1,01 | 9,71E-01 | | | hypothetical protein |
| RJ610_03930 | WND81537.1 | 1,15 | 3,22E-01 | | | hypothetical protein |
| RJ610_03935 | WND81538.1 | 2,86 | 1,27E-18 | | | acetyl-CoA carboxylase biotin carboxylase subunit |
| RJ610_03940 | WND81539.1 | 3,12 | 3,70E-19 | | | four helix bundle protein |
| RJ610_03945 | WND81540.1 | 2,96 | 2,72E-18 | | | acetyl-CoA carboxylase biotin carboxyl carrier protein |
| RJ610_03950 | WND81541.1 | 5,80 | 1,18E-25 | | | type II 3-dehydroquinate dehydratase |
| RJ610_03955 | WND81542.1 | 1,84 | 3,47E-08 | | | TlpA disulfide reductase family protein |
| RJ610_03965 | WND81544.1 | 1,12 | 3,74E-01 | | | divalent-cation tolerance protein CutA |
| RJ610_03970 | WND81545.1 | 0,89 | 8,58E-01 | | | LysR substrate-binding domain-containing protein |
| RJ610_03975 | WND81546.1 | 0,67 | 4,93E-03 | | | AcvB/VirJ family lysyl-phosphatidylglycerol hydrolase |
| RJ610_03980 | | 0,70 | 1,02E-03 | | | bifunctional lysylphosphatidylglycerol flippase/synthetase MprF |
| RJ610_03985 | WND81547.1 | 0,84 | 2,82E-01 | | | LysR family transcriptional regulator |
| RJ610_03990 | WND81548.1 | 0,95 | 8,42E-01 | | | NAD(P)-dependent alcohol dehydrogenase |
| RJ610_04000 | WND81550.1 | 1,19 | 2,02E-01 | | | helix-turn-helix domain-containing protein |
| RJ610_04005 | WND81551.1 | 1,27 | 3,96E-02 | | | cupin domain-containing protein |
| RJ610_04010 | WND81552.1 | 0,88 | 5,70E-01 | | | hypothetical protein |
| RJ610_04015 | WND81553.1 | 1,28 | 1,23E-01 | | | AraC family ligand binding domain-containing protein |
| RJ610_04020 | WND81554.1 | 1,35 | 2,78E-01 | | | helix-turn-helix domain-containing protein |
| RJ610_04035 | WND81557.1 | 2,23 | 1,07E-02 | | | hypothetical protein |
| RJ610_04040 | WND81558.1 | 2,01 | 6,55E-02 | | | hypothetical protein |
| RJ610_04045 | WND81559.1 | 3,49 | 2,77E-05 | | | hypothetical protein |
| RJ610_04050 | WND81560.1 | 0,86 | 3,20E-01 | | | alpha/beta fold hydrolase |
| RJ610_04055 | WND81561.1 | 1,76 | 2,47E-03 | | | multicopper oxidase family protein |
| RJ610_04060 | WND81562.1 | 0,62 | 9,80E-04 | | | hypothetical protein |
| RJ610_04065 | WND81563.1 | 0,78 | 8,73E-02 | | | hypothetical protein |
| RJ610_04070 | WND81564.1 | 0,60 | 3,31E-05 | | | bifunctional [glutamate--ammonia ligase]-adenylyl-L-tyrosine phosphorylase/[glutamate--ammonia-ligase] adenylyltransferase |
| RJ610_04075 | WND81565.1 | 0,75 | 7,92E-02 | | | immunity 22 family protein |
| RJ610_04080 | WND83168.1 | 0,54 | 2,99E-07 | | | mitochondrial fission ELM1 family protein |

| | | | | | | |
|---|---|---|---|---|---|---|
| RJ610_04085 | WND81566.1 | 0,58 | 1,11E-07 | | | malonic semialdehyde reductase |
| RJ610_04100 | WND81569.1 | 0,52 | 1,97E-11 | | | zinc-finger domain-containing protein |
| RJ610_04105 | WND81570.1 | 0,52 | 2,85E-11 | | | glycosyltransferase |
| RJ610_04110 | WND81571.1 | 0,64 | 2,83E-04 | | | O-antigen ligase |
| RJ610_04115 | WND81572.1 | 0,61 | 8,50E-04 | | | LpxL/LpxP family Kdo(2)-lipid IV(A) lauroyl/palmitoleoyl acyltransferase |
| RJ610_04120 | WND81573.1 | 0,66 | 1,11E-03 | | | lipid IV(A) 3-deoxy-D-manno-octulosonic acid transferase |
| RJ610_04130 | WND83169.1 | 0,69 | 4,60E-04 | | | protein-L-isoaspartate O-methyltransferase |
| RJ610_04135 | WND81575.1 | 1,44 | 1,39E-04 | | | TetR/AcrR family transcriptional regulator |
| RJ610_04140 | WND81576.1 | 2,03 | 6,07E-11 | | | efflux RND transporter periplasmic adaptor subunit |
| RJ610_04145 | WND81577.1 | 2,19 | 2,26E-11 | | | efflux RND transporter permease subunit |
| RJ610_04150 | WND81578.1 | 2,86 | 2,25E-12 | | | glycerol kinase GlpK |
| RJ610_04155 | WND81579.1 | 3,42 | 2,43E-11 | | | MIP/aquaporin family protein |
| RJ610_04160 | WND83170.1 | 8,93 | 2,00E-51 | | | glycerol-3-phosphate dehydrogenase |
| RJ610_04165 | WND81580.1 | 0,87 | 6,30E-01 | | | 3-isopropylmalate dehydrogenase |
| RJ610_04170 | WND81581.1 | 0,96 | 8,95E-01 | | | 3-isopropylmalate dehydratase small subunit |
| RJ610_04175 | WND81582.1 | 0,90 | 8,26E-01 | | | 3-isopropylmalate dehydratase large subunit |
| RJ610_04180 | WND81583.1 | 0,90 | 7,43E-01 | | | 2-isopropylmalate synthase |
| RJ610_04185 | WND81584.1 | 0,98 | 9,43E-01 | | | threonine dehydratase |
| RJ610_04190 | WND81585.1 | 1,02 | 9,38E-01 | | | aminotransferase class IV |
| RJ610_04195 | WND81586.1 | 0,99 | 9,85E-01 | | | ACT domain-containing protein |
| RJ610_04200 | WND81587.1 | 0,98 | 9,53E-01 | | | acetolactate synthase 2 catalytic subunit |
| RJ610_04205 | WND81588.1 | 0,77 | 4,09E-01 | | | ketol-acid reductoisomerase |
| RJ610_04210 | WND81589.1 | 0,69 | 1,41E-01 | | | dihydroxy-acid dehydratase |
| RJ610_04215 | WND81590.1 | 0,67 | 1,63E-01 | | | hypothetical protein |
| RJ610_04220 | WND81591.1 | 0,69 | 4,81E-01 | | | threonine synthase |
| RJ610_04225 | WND81592.1 | 0,61 | 3,42E-01 | | | homoserine kinase |
| RJ610_04230 | WND81593.1 | 0,65 | 1,35E-01 | | | bifunctional aspartate kinase/homoserine dehydrogenase I |
| RJ610_04245 | WND81596.1 | 5,26 | 9,50E-31 | | | hypothetical protein |
| RJ610_04255 | WND81598.1 | 0,82 | 1,87E-01 | | | ion channel |
| RJ610_04260 | WND81599.1 | 0,95 | 6,21E-01 | | | winged helix-turn-helix domain-containing protein |
| RJ610_04265 | WND81600.1 | 0,88 | 4,03E-01 | | | helix-turn-helix domain-containing protein |
| RJ610_04280 | WND81603.1 | 2,81 | 5,97E-26 | | | sterol desaturase family protein |
| RJ610_04285 | WND81604.1 | 2,50 | 1,96E-17 | | | discoidin domain-containing protein |
| RJ610_04290 | WND81605.1 | 2,12 | 2,43E-07 | | | M91 family zinc metallopeptidase |
| RJ610_04295 | WND81606.1 | 0,51 | 2,93E-08 | | | M91 family zinc metallopeptidase |
| RJ610_04300 | WND81607.1 | 0,37 | 2,24E-18 | | | hypothetical protein |
| RJ610_04305 | WND81608.1 | 0,40 | 1,36E-11 | | | hypothetical protein |
| RJ610_04310 | WND81609.1 | 0,68 | 1,12E-03 | | | prolyl oligopeptidase family serine peptidase |
| RJ610_04315 | WND83171.1 | 5,40 | 9,52E-59 | | | OmpA family protein |
| RJ610_04320 | | 4,23 | 3,06E-34 | | | sodium-translocating pyrophosphatase |
| RJ610_04325 | WND81610.1 | 1,70 | 1,45E-03 | | | hypothetical protein |
| RJ610_04330 | WND81611.1 | 2,87 | 1,37E-25 | | | hypothetical protein |

| | | | | | | |
|---|---|---|---|---|---|---|
| RJ610_04335 | WND81612.1 | 1,37 | 2,93E-02 | | | VOC family protein |
| RJ610_04340 | WND81613.1 | 1,13 | 3,51E-01 | | | AraC family transcriptional regulator |
| RJ610_04345 | WND81614.1 | 1,05 | 7,51E-01 | | | 6-phosphofructokinase |
| RJ610_04350 | WND81615.1 | 1,02 | 8,55E-01 | | | adenylate kinase |
| RJ610_04355 | WND83172.1 | 0,97 | 7,96E-01 | | | UDP-N-acetylmuramate:L-alanyl-gamma-D-glutamyl-meso-diaminopimelate ligase |
| RJ610_04360 | WND81616.1 | 1,01 | 9,28E-01 | | | LON peptidase substrate-binding domain-containing protein |
| RJ610_04365 | WND81617.1 | 0,58 | 4,99E-07 | | | bifunctional DedA family/phosphatase PAP2 family protein |
| RJ610_04370 | WND81618.1 | 0,53 | 1,78E-06 | | | serine/threonine protein kinase |
| RJ610_04375 | WND81619.1 | 0,72 | 1,52E-03 | | | NAD(P)-dependent oxidoreductase |
| RJ610_04380 | WND81620.1 | 1,78 | 2,69E-04 | | | M20/M25/M40 family metallo-hydrolase |
| RJ610_04390 | WND81622.1 | 1,22 | 1,75E-01 | | | HIT family protein |
| RJ610_04400 | WND81624.1 | 0,89 | 3,04E-01 | | | glutamate-1-semialdehyde 2,1-aminomutase |
| RJ610_04405 | WND81625.1 | 1,16 | 3,59E-01 | | | thiamine phosphate synthase |
| RJ610_04410 | WND81626.1 | 0,72 | 3,12E-03 | | | rubredoxin |
| RJ610_04415 | WND81627.1 | 0,69 | 1,40E-03 | | | DUF192 domain-containing protein |
| RJ610_04450 | WND83173.1 | 0,65 | 2,13E-04 | | | ABC transporter ATP-binding protein |
| RJ610_04455 | WND81629.1 | 0,53 | 1,26E-07 | | | hypothetical protein |
| RJ610_04460 | WND81630.1 | 0,75 | 7,28E-03 | | | ribose-5-phosphate isomerase RpiA |
| RJ610_04465 | WND81631.1 | 0,83 | 1,23E-01 | | | EVE domain-containing protein |
| RJ610_04470 | WND81632.1 | 0,82 | 1,01E-01 | | | 5-formyltetrahydrofolate cyclo-ligase |
| RJ610_04475 | | 0,81 | 3,79E-01 | | | 6S RNA |
| RJ610_04480 | WND81633.1 | 0,78 | 8,62E-03 | | | cell division protein ZapA |
| RJ610_04485 | WND83174.1 | 0,80 | 3,48E-02 | | | TIGR02449 family protein |
| RJ610_04490 | WND81634.1 | 0,61 | 1,29E-06 | | | EAL domain-containing protein |
| RJ610_04495 | WND81635.1 | 0,50 | 4,50E-10 | | | UPF0149 family protein |
| RJ610_04500 | WND81636.1 | 0,60 | 3,87E-05 | | | aminopeptidase P N-terminal domain-containing protein |
| RJ610_04505 | WND81637.1 | 0,61 | 7,60E-04 | | | LysR family transcriptional regulator |
| RJ610_04510 | WND81638.1 | 0,52 | 2,42E-05 | | | short chain dehydrogenase |
| RJ610_04515 | | 0,90 | 5,52E-01 | | | phosphotyrosine protein phosphatase |
| RJ610_04525 | WND81640.1 | 0,92 | 6,27E-01 | | | PilZ domain-containing protein |
| RJ610_04530 | WND81641.1 | 0,72 | 1,11E-03 | | | DUF1631 family protein |
| RJ610_04535 | WND81642.1 | 1,74 | 2,28E-07 | | | radical SAM family heme chaperone HemW |
| RJ610_04540 | WND81643.1 | 95,22 | 8,63E-235 | | | hypothetical protein |
| RJ610_04545 | WND81644.1 | 106,85 | 2,38E-256 | | | RdgB/HAM1 family non-canonical purine NTP pyrophosphatase |
| RJ610_04555 | WND81646.1 | 0,85 | 1,89E-01 | | | ribonuclease PH |
| RJ610_04560 | WND81647.1 | 0,74 | 1,10E-02 | | | YicC/YloC family endoribonuclease |
| RJ610_04565 | WND81648.1 | 0,74 | 8,98E-03 | | | guanylate kinase |
| RJ610_04570 | WND81649.1 | 0,92 | 4,72E-01 | | | DNA-directed RNA polymerase subunit omega |
| RJ610_04575 | WND81650.1 | 0,94 | 5,45E-01 | | | bifunctional (p)ppGpp synthetase/guanosine-3',5'-bis(diphosphate) 3'-pyrophosphohydrolase |
| RJ610_04585 | WND81652.1 | 0,94 | 6,32E-01 | | | AraC family transcriptional regulator |
| RJ610_04590 | WND81653.1 | 0,44 | 3,02E-12 | | | parallel beta-helix domain-containing protein |

| | | | | | | |
|---|---|---|---|---|---|---|
| RJ610_04595 | WND81654.1 | 0,63 | 4,61E-03 | | | SO2930 family diheme c-type cytochrome |
| RJ610_04600 | WND81655.1 | 1,29 | 5,30E-02 | | | TonB-dependent receptor |
| RJ610_04605 | WND81656.1 | 0,75 | 4,10E-03 | | | ATP-dependent DNA helicase RecG |
| RJ610_04610 | WND81657.1 | 0,16 | 6,94E-59 | | | calcium-binding protein |
| RJ610_04615 | WND81658.1 | 0,59 | 6,82E-05 | | | DUF4166 domain-containing protein |
| RJ610_04620 | WND81659.1 | 0,70 | 8,23E-04 | | | nucleoside hydrolase |
| RJ610_04625 | WND81660.1 | 0,88 | 3,56E-01 | | | type B 50S ribosomal protein L31 |
| RJ610_04635 | WND81662.1 | 0,56 | 1,12E-03 | | | hypothetical protein |
| RJ610_04640 | WND81663.1 | 0,46 | 4,47E-09 | | | hypothetical protein |
| RJ610_04645 | WND81664.1 | 0,50 | 7,59E-05 | | | hypothetical protein |
| RJ610_04650 | WND81665.1 | 0,67 | 2,62E-02 | | | hypothetical protein |
| RJ610_04655 | WND81666.1 | 0,71 | 3,02E-03 | | | penicillin-binding protein 1A |
| RJ610_04660 | | 3,95 | 3,97E-28 | | | pilus assembly protein PilM |
| RJ610_04665 | WND81667.1 | 3,11 | 2,06E-10 | | | PilN domain-containing protein |
| RJ610_04670 | WND81668.1 | 3,12 | 1,69E-13 | | | type 4a pilus biogenesis protein PilO |
| RJ610_04675 | WND81669.1 | 2,12 | 5,44E-07 | | | pilus assembly protein PilP |
| RJ610_04680 | WND81670.1 | 1,57 | 6,65E-04 | | | type IV pilus secretin PilQ |
| RJ610_04685 | WND81671.1 | 1,05 | 7,53E-01 | | | MoxR family ATPase |
| RJ610_04690 | WND81672.1 | 1,12 | 3,88E-01 | | | DUF58 domain-containing protein |
| RJ610_04695 | WND81673.1 | 1,01 | 9,69E-01 | | | DUF4381 domain-containing protein |
| RJ610_04700 | WND81674.1 | 1,51 | 3,07E-02 | | | VWA domain-containing protein |
| RJ610_04705 | WND81675.1 | 1,06 | 7,26E-01 | | | VWA domain-containing protein |
| RJ610_04710 | WND81676.1 | 1,25 | 1,21E-01 | | | BatD family protein |
| RJ610_04715 | WND81677.1 | 0,85 | 1,38E-01 | | | dicarboxylate/amino acid:cation symporter |
| RJ610_04720 | WND81678.1 | 1,66 | 2,48E-02 | | | transporter |
| RJ610_04725 | WND81679.1 | 0,71 | 6,44E-02 | | | IclR family transcriptional regulator C-terminal domain-containing protein |
| RJ610_04730 | WND81680.1 | 1,78 | 9,98E-02 | | | CoA transferase subunit A |
| RJ610_04735 | WND81681.1 | 1,06 | 9,18E-01 | | | CoA-transferase subunit beta |
| RJ610_04740 | WND81682.1 | 0,68 | 2,56E-01 | | | 3-oxoadipyl-CoA thiolase |
| RJ610_04745 | WND81683.1 | 0,91 | 8,30E-01 | | | protocatechuate 3,4-dioxygenase subunit beta |
| RJ610_04750 | WND81684.1 | 1,52 | 3,65E-01 | | | protocatechuate 3,4-dioxygenase subunit alpha |
| RJ610_04755 | WND81685.1 | 1,46 | 5,73E-01 | | | 3-carboxy-cis,cis-muconate cycloisomerase |
| RJ610_04760 | | 1,21 | 8,20E-01 | | | 3-oxoadipate enol-lactonase |
| RJ610_04765 | WND83175.1 | 1,45 | 3,52E-01 | | | 4-carboxymuconolactone decarboxylase |
| RJ610_04770 | WND81686.1 | 0,82 | 5,21E-01 | | | 4-hydroxybenzoate 3-monooxygenase |
| RJ610_04775 | WND81687.1 | 1,28 | 3,67E-01 | | | MFS transporter |
| RJ610_04780 | WND81688.1 | 1,13 | 2,73E-01 | | | transketolase |
| RJ610_04785 | WND81689.1 | 1,15 | 5,97E-01 | | | transposase |
| RJ610_04790 | WND81690.1 | 0,91 | 4,14E-01 | | | acetyl-CoA hydrolase/transferase C-terminal domain-containing protein |
| RJ610_04795 | WND81691.1 | 0,79 | 1,47E-01 | | | ATP-binding cassette domain-containing protein |
| RJ610_04800 | WND81692.1 | 0,95 | 7,60E-01 | | | molybdate ABC transporter permease subunit |

| | | | | | | |
|---|---|---|---|---|---|---|
| RJ610_04805 | WND83176.1 | 0,89 | 3,85E-01 | | | molybdate ABC transporter substrate-binding protein |
| RJ610_04810 | WND81693.1 | 1,25 | 1,57E-01 | | | MBL fold metallo-hydrolase |
| RJ610_04830 | WND81697.1 | 0,86 | 3,45E-01 | | | hypothetical protein |
| RJ610_04835 | WND81698.1 | 1,03 | 8,59E-01 | | | ATP-binding cassette domain-containing protein |
| RJ610_04840 | WND81699.1 | 4,94 | 1,33E-08 | | | hypothetical protein |
| RJ610_04850 | WND81701.1 | 0,47 | 1,42E-11 | | | hypothetical protein |
| RJ610_04865 | WND81704.1 | 0,62 | 1,52E-04 | | | ribonucleotide-diphosphate reductase subunit beta |
| RJ610_04870 | WND83177.1 | 0,82 | 2,26E-01 | | | ribonucleoside-diphosphate reductase subunit alpha |
| RJ610_04875 | WND81705.1 | 1,09 | 4,54E-01 | | | DUF2339 domain-containing protein |
| RJ610_04880 | WND81706.1 | 0,17 | 5,81E-42 | | | DUF3999 domain-containing protein |
| RJ610_04885 | WND81707.1 | 0,94 | 6,74E-01 | | | phosphoglycerate kinase |
| RJ610_04890 | WND81708.1 | 1,04 | 8,58E-01 | | | HAD hydrolase-like protein |
| RJ610_04895 | WND81709.1 | 30,53 | 1,39E-187 | | | DUF6289 family protein |
| RJ610_04905 | WND81711.1 | 65,15 | 6,91E-164 | | | DUF6289 family protein |
| RJ610_04910 | WND81712.1 | 11,43 | 1,74E-52 | | | GNAT family N-acetyltransferase |
| RJ610_04920 | WND81714.1 | 0,66 | 2,15E-04 | | | hypothetical protein |
| RJ610_04930 | WND81716.1 | 0,54 | 6,25E-03 | | | LysE family translocator |
| RJ610_04935 | WND81717.1 | 0,47 | 8,36E-04 | | | hypothetical protein |
| RJ610_04940 | WND81718.1 | 0,56 | 6,93E-04 | | | hypothetical protein |
| RJ610_04950 | WND81720.1 | 0,21 | 4,22E-32 | | | efflux RND transporter periplasmic adaptor subunit |
| RJ610_04955 | WND81721.1 | 0,25 | 2,57E-19 | | | hypothetical protein |
| RJ610_04960 | WND81722.1 | 0,31 | 8,13E-25 | | | efflux RND transporter permease subunit |
| RJ610_04965 | WND81723.1 | 0,44 | 1,43E-05 | | | hypothetical protein |
| RJ610_04970 | WND81724.1 | 0,71 | 1,03E-01 | | | hypothetical protein |
| RJ610_04975 | WND81725.1 | 0,69 | 1,39E-02 | | | GNAT family protein |
| RJ610_04980 | | 0,78 | 5,56E-02 | | | RNA methyltransferase |
| RJ610_04985 | WND81726.1 | 7,84 | 3,22E-14 | | | RebB family R body protein |
| RJ610_04995 | WND81728.1 | 0,97 | 8,62E-01 | | | large-conductance mechanosensitive channel protein MscL |
| RJ610_05000 | WND81729.1 | 1,00 | 9,99E-01 | | | fumarylacetoacetate hydrolase family protein |
| RJ610_05005 | WND81730.1 | 0,75 | 8,04E-02 | | | hypothetical protein |
| RJ610_05010 | WND83178.1 | 1,06 | 6,89E-01 | | | Rieske 2Fe-2S domain-containing protein |
| RJ610_05015 | WND81731.1 | 1,12 | 2,76E-01 | | | SLC13 family permease |
| RJ610_05020 | WND81732.1 | 1,04 | 7,56E-01 | | | tRNA (guanosine(46)-N7)-methyltransferase TrmB |
| RJ610_05025 | WND81733.1 | 0,96 | 7,44E-01 | | | thiazole synthase |
| RJ610_05030 | WND81734.1 | 0,99 | 9,73E-01 | | | phosphatidylinositol-specific phospholipase C1-like protein |
| RJ610_05035 | WND81735.1 | 1,67 | 7,07E-03 | | | hypothetical protein |
| RJ610_05040 | WND81736.1 | 2,11 | 1,61E-03 | | | hypothetical protein |
| RJ610_05045 | WND81737.1 | 0,83 | 1,22E-01 | | | sulfur carrier protein ThiS |
| RJ610_05055 | | 0,43 | 3,50E-01 | | | tRNA-Gly |
| RJ610_05060 | WND81738.1 | 0,83 | 3,26E-01 | | | LTA synthase family protein |
| RJ610_05065 | WND81739.1 | 0,53 | 5,13E-02 | | | hypothetical protein |
| RJ610_05070 | WND81740.1 | 0,96 | 8,39E-01 | | | RimK/LysX family protein |
| RJ610_05075 | WND81741.1 | 0,96 | 8,42E-01 | | | 30S ribosomal protein S6--L-glutamate ligase |

| | | | | | | |
|---|---|---|---|---|---|---|
| RJ610_05085 | WND81743.1 | 0,94 | 5,37E-01 | | | HAMP domain-containing sensor histidine kinase |
| RJ610_05095 | WND81745.1 | 3,89 | 1,20E-33 | | | hypothetical protein |
| RJ610_05105 | WND81747.1 | 3,04 | 1,24E-06 | | | methylenetetrahydrofolate reductase [NAD(P)H] |
| RJ610_05110 | WND81748.1 | 4,07 | 8,67E-26 | | | GNAT family N-acetyltransferase |
| RJ610_05115 | WND81749.1 | 2,46 | 2,64E-09 | | | RHS repeat domain-containing protein |
| RJ610_05120 | WND81750.1 | 1,38 | 9,33E-02 | | | RHS repeat-associated core domain-containing protein |
| RJ610_05125 | WND81751.1 | 0,90 | 6,16E-01 | | | hypothetical protein |
| RJ610_05130 | WND83180.1 | 1,11 | 5,16E-01 | | | RHS repeat-associated core domain-containing protein |
| RJ610_05135 | WND81752.1 | 0,80 | 1,17E-01 | | | hypothetical protein |
| RJ610_05140 | WND81753.1 | 1,09 | 6,37E-01 | | | alpha/beta hydrolase |
| RJ610_05145 | WND81754.1 | 1,14 | 3,46E-01 | | | histidine kinase |
| RJ610_05150 | WND81755.1 | 1,24 | 3,05E-01 | | | LytTR family DNA-binding domain-containing protein |
| RJ610_05155 | WND81756.1 | 1,31 | 6,14E-02 | | | hypothetical protein |
| RJ610_05160 | WND81757.1 | 1,34 | 1,13E-02 | | | hypothetical protein |
| RJ610_05170 | WND83181.1 | 0,67 | 9,40E-04 | | | prolyl oligopeptidase family serine peptidase |
| RJ610_05175 | WND81759.1 | 0,96 | 8,46E-01 | | | hypothetical protein |
| RJ610_05180 | WND81760.1 | 1,24 | 3,87E-01 | | | DUF3955 domain-containing protein |
| RJ610_05185 | WND81761.1 | 1,24 | 2,73E-01 | | | OmpA family protein |
| RJ610_05190 | WND81762.1 | 0,78 | 8,54E-02 | | | methionine adenosyltransferase |
| RJ610_05195 | WND81763.1 | 0,28 | 8,59E-23 | | | lysophospholipid acyltransferase family protein |
| RJ610_05200 | WND81764.1 | 0,35 | 5,59E-11 | | | UDP-2,3-diacylglucosamine diphosphatase |
| RJ610_05205 | WND81765.1 | 0,44 | 2,15E-08 | | | amino acid racemase |
| RJ610_05210 | WND81766.1 | 0,49 | 2,73E-07 | | | metal-dependent hydrolase |
| RJ610_05215 | WND81767.1 | 0,88 | 5,09E-01 | | | PEGA domain-containing protein |
| RJ610_05220 | WND81768.1 | 0,65 | 1,22E-04 | | | tRNA dihydrouridine synthase DusB |
| RJ610_05225 | WND81769.1 | 1,58 | 2,58E-01 | | | YegP family protein |
| RJ610_05230 | WND81770.1 | 0,74 | 9,10E-02 | | | alpha/beta hydrolase |
| RJ610_05235 | WND81771.1 | 0,13 | 1,07E-64 | | | PepSY-associated TM helix domain-containing protein |
| RJ610_05250 | WND81774.1 | 0,70 | 9,08E-03 | | | nucleoside transporter C-terminal domain-containing protein |
| RJ610_05255 | WND81775.1 | 0,86 | 4,05E-01 | | | GGDEF domain-containing protein |
| RJ610_05260 | WND81776.1 | 2,00 | 4,08E-06 | | | TMEM165/GDT1 family protein |
| RJ610_05265 | WND81777.1 | 5,17 | 4,36E-33 | | | hypothetical protein |
| RJ610_05270 | WND81778.1 | 3,09 | 2,50E-15 | | | aldo/keto reductase |
| RJ610_05275 | WND81779.1 | 1,07 | 8,04E-01 | | | hemin uptake protein HemP |
| RJ610_05285 | WND81781.1 | 1,30 | 8,93E-02 | | | Hemin transport protein |
| RJ610_05295 | WND81783.1 | 0,93 | 6,91E-01 | | | hypothetical protein |
| RJ610_05300 | WND81784.1 | 0,88 | 3,41E-01 | | | PDZ domain-containing protein |
| RJ610_05305 | WND81785.1 | 0,82 | 2,45E-01 | | | hypothetical protein |
| RJ610_05310 | WND81786.1 | 0,88 | 4,45E-01 | | | sigma-70 family RNA polymerase sigma factor |
| RJ610_05315 | WND81787.1 | 0,56 | 1,44E-09 | | | SPFH domain-containing protein |
| RJ610_05320 | WND81788.1 | 0,57 | 4,53E-05 | | | NfeD family protein |
| RJ610_05325 | WND81789.1 | 0,67 | 4,17E-03 | | | hypothetical protein |
| RJ610_05330 | WND81790.1 | 0,65 | 4,25E-05 | | | glycine cleavage system aminomethyltransferase GcvT |

| | | | | | | |
|---|---|---|---|---|---|---|
| RJ610_05335 | WND81791.1 | 0,66 | 2,35E-04 | | | 2-hydroxychromene-2-carboxylate isomerase |
| RJ610_05340 | WND81792.1 | 1,03 | 8,02E-01 | | | glycine cleavage system protein GcvH |
| RJ610_05345 | WND81793.1 | 1,31 | 4,37E-01 | | | hypothetical protein |
| RJ610_05350 | WND81794.1 | 0,37 | 2,73E-16 | | | hypothetical protein |
| RJ610_05355 | WND81795.1 | 0,67 | 9,88E-04 | | | hypothetical protein |
| RJ610_05360 | WND81796.1 | 0,79 | 1,14E-01 | | | SRPBCC family protein |
| RJ610_05365 | WND81797.1 | 1,35 | 3,85E-03 | | | serine hydrolase domain-containing protein |
| RJ610_05370 | WND81798.1 | 0,66 | 2,38E-05 | | | choline dehydrogenase |
| RJ610_05375 | WND81799.1 | 0,81 | 1,93E-01 | | | MFS transporter |
| RJ610_05380 | WND81800.1 | 0,92 | 6,57E-01 | | | metallophosphoesterase |
| RJ610_05385 | WND81801.1 | 0,38 | 1,04E-06 | | | MFS transporter |
| RJ610_05390 | WND81802.1 | 0,44 | 1,64E-06 | | | NAD-dependent epimerase/dehydratase family protein |
| RJ610_05395 | WND81803.1 | 1,48 | 5,36E-03 | | | DUF1304 domain-containing protein |
| RJ610_05400 | WND81804.1 | 0,20 | 3,07E-63 | | | DUF4132 domain-containing protein |
| RJ610_05405 | WND81805.1 | 0,21 | 2,83E-14 | | | AAA family ATPase |
| RJ610_05410 | WND81806.1 | 0,20 | 7,77E-21 | | | DUF5682 family protein |
| RJ610_05415 | WND81807.1 | 0,20 | 5,74E-22 | | | VWA domain-containing protein |
| RJ610_05420 | WND81808.1 | 1,10 | 4,90E-01 | | | SWIM zinc finger family protein |
| RJ610_05425 | WND81809.1 | 3,48 | 1,62E-24 | | | NAD(P)H:quinone oxidoreductase |
| RJ610_05430 | WND81810.1 | 4,29 | 3,23E-28 | | | hypothetical protein |
| RJ610_05435 | WND81811.1 | 2,00 | 1,26E-10 | | | MFS transporter |
| RJ610_05445 | WND81813.1 | 0,80 | 6,02E-02 | | | hypothetical protein |
| RJ610_05450 | WND81814.1 | 0,88 | 3,16E-01 | | | hypothetical protein |
| RJ610_05455 | WND81815.1 | 1,03 | 8,86E-01 | | | hotdog fold domain-containing protein |
| RJ610_05460 | WND81816.1 | 1,34 | 2,05E-02 | | | acyl-CoA dehydrogenase |
| RJ610_05465 | WND81817.1 | 1,07 | 5,90E-01 | | | TetR/AcrR family transcriptional regulator |
| RJ610_05470 | WND81818.1 | 1,27 | 1,76E-02 | | | phosphoenolpyruvate carboxykinase (GTP) |
| RJ610_05475 | WND81819.1 | 1,39 | 7,51E-02 | | | sigma-70 family RNA polymerase sigma factor |
| RJ610_05480 | WND81820.1 | 1,19 | 3,26E-01 | | | hypothetical protein |
| RJ610_05485 | WND81821.1 | 1,07 | 6,79E-01 | | | DUF4097 family beta strand repeat-containing protein |
| RJ610_05490 | WND81822.1 | 0,48 | 2,15E-09 | | | aspartyl/asparaginyl beta-hydroxylase domain-containing protein |
| RJ610_05510 | WND83182.1 | 1,75 | 1,80E-04 | | | hypothetical protein |
| RJ610_05515 | WND81826.1 | 0,84 | 3,22E-01 | | | Hsp33 family molecular chaperone HslO |
| RJ610_05520 | WND81827.1 | 0,64 | 1,91E-03 | | | monofunctional biosynthetic peptidoglycan transglycosylase |
| RJ610_05525 | WND81828.1 | 0,76 | 2,24E-02 | | | glycosyltransferase family 2 protein |
| RJ610_05530 | WND81829.1 | 1,13 | 3,29E-01 | | | CBS domain-containing protein |
| RJ610_05535 | WND81830.1 | 0,96 | 8,07E-01 | | | hypothetical protein |
| RJ610_05540 | WND81831.1 | 0,88 | 3,60E-01 | | | hypothetical protein |
| RJ610_05545 | WND81832.1 | 0,82 | 1,00E-01 | | | AsmA family protein |
| RJ610_05550 | WND81833.1 | 1,82 | 4,49E-08 | | | hemolysin III family protein |
| RJ610_05555 | WND81834.1 | 1,14 | 5,10E-01 | | | hypothetical protein |
| RJ610_05560 | WND81835.1 | 0,74 | 1,28E-02 | | | peptide chain release factor 3 |
| RJ610_05565 | WND81836.1 | 0,97 | 8,59E-01 | | | M23 family metallopeptidase |

| | | | | | | |
|---|---|---|---|---|---|---|
| RJ610_05570 | WND81837.1 | 0,96 | 8,18E-01 | | | phytase |
| RJ610_05575 | WND81838.1 | 2,58 | 6,41E-02 | | | homoserine O-succinyltransferase |
| RJ610_05580 | WND83183.1 | 4,07 | 2,45E-02 | | | O-succinylhomoserine (thiol)-lyase |
| RJ610_05585 | WND81839.1 | 4,97 | 1,14E-02 | | | homoserine dehydrogenase |
| RJ610_05590 | WND81840.1 | 1,54 | 1,98E-02 | | | DUF3089 domain-containing protein |
| RJ610_05595 | WND81841.1 | 1,80 | 2,36E-03 | | | hypothetical protein |
| RJ610_05605 | WND81842.1 | 1,09 | 5,84E-01 | | | hypothetical protein |
| RJ610_05610 | WND83185.1 | 0,93 | 6,11E-01 | | | alpha/beta hydrolase |
| RJ610_05615 | WND81843.1 | 0,54 | 4,22E-06 | | | hypothetical protein |
| RJ610_05620 | WND81844.1 | 1,00 | 9,89E-01 | | | cytochrome c |
| RJ610_05625 | WND81845.1 | 1,18 | 7,91E-01 | | | (2Fe-2S)-binding protein |
| RJ610_05630 | WND81846.1 | 1,24 | 3,14E-01 | | | molybdopterin cofactor-binding domain-containing protein |
| RJ610_05635 | WND81847.1 | 0,35 | 4,68E-03 | | | alpha/beta hydrolase |
| RJ610_05640 | WND81848.1 | 1,51 | 2,57E-03 | | | hypothetical protein |
| RJ610_05645 | WND81849.1 | 1,25 | 8,80E-02 | | | L-serine ammonia-lyase |
| RJ610_05650 | WND81850.1 | 0,86 | 5,86E-01 | | | glutaredoxin family protein |
| RJ610_05655 | WND81851.1 | 1,17 | 2,92E-01 | | | hypothetical protein |
| RJ610_05665 | | 0,76 | 2,95E-02 | | | cytochrome b |
| RJ610_05670 | WND81853.1 | 0,81 | 1,48E-01 | | | YceI family protein |
| RJ610_05675 | WND81854.1 | 0,38 | 2,86E-14 | | | ATP-binding protein |
| RJ610_05680 | WND81855.1 | 1,58 | 7,55E-07 | | | ATP-binding protein |
| RJ610_05685 | WND81856.1 | 1,13 | 2,39E-01 | | | ATP-binding protein |
| RJ610_05690 | WND81857.1 | 0,92 | 5,94E-01 | | | GntR family transcriptional regulator |
| RJ610_05695 | WND81858.1 | 0,77 | 7,19E-02 | | | ABC transporter ATP-binding protein |
| RJ610_05700 | WND81859.1 | 0,53 | 4,91E-04 | | | hypothetical protein |
| RJ610_05705 | WND81860.1 | 0,88 | 5,83E-01 | | | prolyl oligopeptidase family serine peptidase |
| RJ610_05715 | WND81862.1 | 2,40 | 1,21E-11 | | | WGR domain-containing protein |
| RJ610_05720 | WND81863.1 | 0,57 | 8,15E-05 | | | helix-turn-helix domain-containing protein |
| RJ610_05725 | WND81864.1 | 0,79 | 1,81E-01 | | | cysteine hydrolase family protein |
| RJ610_05730 | WND81865.1 | 0,65 | 5,05E-04 | | | 3-dehydroquinate synthase |
| RJ610_05735 | WND81866.1 | 0,66 | 6,56E-03 | | | shikimate kinase |
| RJ610_05745 | WND81868.1 | 2,04 | 6,71E-06 | | | kinase |
| RJ610_05750 | WND81869.1 | 3,58 | 5,87E-17 | | | pyridoxamine 5'-phosphate oxidase |
| RJ610_05755 | WND81870.1 | 3,72 | 3,48E-14 | | | GNAT family N-acetyltransferase |
| RJ610_05760 | WND81871.1 | 1,97 | 1,62E-05 | | | GNAT family N-acetyltransferase |
| RJ610_05765 | WND81872.1 | 1,04 | 8,10E-01 | | | ABC transporter substrate-binding protein |
| RJ610_05770 | WND81873.1 | 2,27 | 2,48E-11 | | | triacylglycerol lipase |
| RJ610_05775 | WND81874.1 | 2,23 | 1,64E-07 | | | lipase secretion chaperone |
| RJ610_05780 | WND81875.1 | 0,88 | 5,41E-01 | | | DUF4426 domain-containing protein |
| RJ610_05785 | WND81876.1 | 1,15 | 3,09E-01 | | | pyrroline-5-carboxylate reductase |
| RJ610_05790 | WND81877.1 | 1,84 | 5,74E-07 | | | hypothetical protein |
| RJ610_05795 | WND81878.1 | 2,21 | 5,72E-09 | | | YggS family pyridoxal phosphate-dependent enzyme |
| RJ610_05800 | WND81879.1 | 2,79 | 1,26E-16 | | | type IV pilus twitching motility protein PilT |

| | | | | | | |
|---|---|---|---|---|---|---|
| RJ610_05805 | WND81880.1 | 2,30 | 6,36E-14 | | | PilT/PilU family type 4a pilus ATPase |
| RJ610_05815 | WND81882.1 | 1,70 | 1,43E-05 | | | hypothetical protein |
| RJ610_05820 | WND81883.1 | 1,72 | 7,99E-04 | | | hypothetical protein |
| RJ610_05825 | WND81884.1 | 0,88 | 3,93E-01 | | | ATP-grasp domain-containing protein |
| RJ610_05830 | WND81885.1 | 1,00 | 9,96E-01 | | | GNAT family N-acetyltransferase |
| RJ610_05835 | WND81886.1 | 1,17 | 2,55E-01 | | | aromatic ring-hydroxylating dioxygenase subunit alpha |
| RJ610_05840 | WND81887.1 | 0,87 | 3,39E-01 | | | DUF4153 domain-containing protein |
| RJ610_05845 | | 0,61 | 9,75E-07 | | | DNA-3-methyladenine glycosylase I |
| RJ610_05850 | WND81888.1 | 1,84 | 6,91E-07 | | | YqgE/AlgH family protein |
| RJ610_05855 | WND81889.1 | 1,41 | 2,84E-03 | | | Holliday junction resolvase RuvX |
| RJ610_05860 | WND81890.1 | 1,15 | 2,69E-01 | | | aspartate carbamoyltransferase catalytic subunit |
| RJ610_05865 | | 1,20 | 1,36E-01 | | | alkylphosphonate utilization protein |
| RJ610_05870 | WND81891.1 | 0,91 | 4,42E-01 | | | hypothetical protein |
| RJ610_05875 | WND81892.1 | 0,84 | 1,25E-01 | | | prolyl oligopeptidase family serine peptidase |
| RJ610_05880 | WND81893.1 | 0,44 | 2,61E-02 | | | hypothetical protein |
| RJ610_05885 | WND81894.1 | 1,03 | 8,33E-01 | | | magnesium transporter |
| RJ610_05890 | WND81895.1 | 1,11 | 3,85E-01 | | | hypothetical protein |
| RJ610_05895 | WND81896.1 | 0,74 | 1,85E-03 | | | phosphoenolpyruvate--protein phosphotransferase |
| RJ610_05900 | WND81897.1 | 0,82 | 1,52E-01 | | | HPr family phosphocarrier protein |
| RJ610_05905 | WND81898.1 | 1,07 | 5,97E-01 | | | PTS sugar transporter subunit IIA |
| RJ610_05910 | WND81899.1 | 0,65 | 1,67E-01 | | | hypothetical protein |
| RJ610_05915 | WND81900.1 | 0,50 | 3,73E-03 | | | hypothetical protein |
| RJ610_05920 | WND83186.1 | 0,64 | 8,80E-06 | | | RNase adapter RapZ |
| RJ610_05925 | WND81901.1 | 0,70 | 2,32E-04 | | | HPr(Ser) kinase/phosphatase |
| RJ610_05930 | WND81902.1 | 0,87 | 1,35E-01 | | | PTS sugar transporter subunit IIA |
| RJ610_05935 | WND81903.1 | 0,82 | 1,03E-01 | | | ribosome-associated translation inhibitor RaiA |
| RJ610_05940 | WND81904.1 | 1,18 | 1,16E-01 | | | RNA polymerase factor sigma-54 |
| RJ610_05945 | WND81905.1 | 0,85 | 8,86E-02 | | | LPS export ABC transporter ATP-binding protein |
| RJ610_05950 | WND81906.1 | 0,84 | 1,07E-01 | | | lipopolysaccharide transport periplasmic protein LptA |
| RJ610_05955 | WND81907.1 | 0,87 | 2,33E-01 | | | LPS export ABC transporter periplasmic protein LptC |
| RJ610_05960 | WND81908.1 | 0,96 | 7,81E-01 | | | HAD hydrolase family protein |
| RJ610_05965 | WND81909.1 | 1,40 | 3,77E-04 | | | KpsF/GutQ family sugar-phosphate isomerase |
| RJ610_05970 | WND81910.1 | 0,71 | 4,47E-03 | | | BolA/IbaG family iron-sulfur metabolism protein |
| RJ610_05975 | WND81911.1 | 0,62 | 6,42E-08 | | | UDP-N-acetylglucosamine 1-carboxyvinyltransferase |
| RJ610_05985 | WND81913.1 | 0,92 | 6,30E-01 | | | DUF3108 domain-containing protein |
| RJ610_05990 | WND81914.1 | 0,67 | 3,98E-03 | | | phosphoribosylglycinamide formyltransferase |
| RJ610_05995 | WND81915.1 | 1,19 | 2,73E-01 | | | TonB-dependent receptor |
| RJ610_06000 | WND81916.1 | 0,69 | 1,87E-01 | | | alpha-D-ribose 1-methylphosphonate 5-triphosphate diphosphatase |
| RJ610_06005 | WND81917.1 | 0,63 | 2,43E-01 | | | phosphonate metabolism transcriptional regulator PhnF |
| RJ610_06015 | WND81919.1 | 2,60 | 2,47E-09 | | | tetratricopeptide repeat protein |
| RJ610_06020 | WND81920.1 | 5,05 | 2,37E-27 | | | hypothetical protein |
| RJ610_06025 | WND81921.1 | 3,32 | 3,51E-04 | | | TIGR03364 family FAD-dependent oxidoreductase |

| | | | | | | |
|---|---|---|---|---|---|---|
| RJ610_06030 | WND81922.1 | 1,45 | 2,73E-01 | | | DUF5690 family protein |
| RJ610_06035 | WND81923.1 | 0,67 | 3,52E-01 | | | HAD hydrolase-like protein |
| RJ610_06040 | WND81924.1 | 0,69 | 8,88E-03 | | | hypothetical protein |
| RJ610_06045 | WND81925.1 | 0,63 | 2,47E-03 | | | DUF4259 domain-containing protein |
| RJ610_06050 | WND81926.1 | 0,46 | 1,68E-03 | | | hypothetical protein |
| RJ610_06055 | WND81927.1 | 0,35 | 3,87E-07 | | | hypothetical protein |
| RJ610_06060 | WND81928.1 | 0,32 | 1,16E-15 | | | hypothetical protein |
| RJ610_06065 | WND81929.1 | 0,58 | 7,18E-03 | | | hypothetical protein |
| RJ610_06070 | WND81930.1 | 0,75 | 5,26E-02 | | | DUF2238 domain-containing protein |
| RJ610_06080 | WND81932.1 | 0,48 | 2,00E-09 | | | DUF2066 domain-containing protein |
| RJ610_06085 | WND81933.1 | 0,47 | 1,37E-11 | | | AI-2E family transporter |
| RJ610_06090 | WND81934.1 | 0,48 | 9,30E-10 | | | DnaA regulatory inactivator Hda |
| RJ610_06095 | WND81935.1 | 0,63 | 5,48E-02 | | | hypothetical protein |
| RJ610_06100 | WND81936.1 | 0,75 | 2,43E-01 | | | O-acetyl-ADP-ribose deacetylase |
| RJ610_06105 | WND81937.1 | 0,35 | 8,07E-09 | | | RNA 2'-phosphotransferase |
| RJ610_06110 | WND81938.1 | 0,53 | 4,57E-05 | | | nucleotidyltransferase family protein |
| RJ610_06115 | WND81939.1 | 0,54 | 2,65E-05 | | | phosphotransferase |
| RJ610_06125 | WND81941.1 | 3,31 | 1,07E-01 | | | siroheme synthase CysG |
| RJ610_06130 | WND81942.1 | 1,96 | 3,07E-03 | | | LysR family transcriptional regulator |
| RJ610_06135 | WND81943.1 | 4,12 | | | | phosphoadenylyl-sulfate reductase |
| RJ610_06140 | WND81944.1 | 12,98 | 2,68E-08 | | | assimilatory sulfite reductase (NADPH) hemoprotein subunit |
| RJ610_06145 | WND81945.1 | 4,10 | | | | assimilatory sulfite reductase (NADPH) flavoprotein subunit |
| RJ610_06150 | WND83187.1 | 1,16 | 8,15E-01 | | | sulfate adenylyltransferase subunit CysD |
| RJ610_06155 | WND83188.1 | 1,04 | 9,57E-01 | | | sulfate adenylyltransferase subunit CysN |
| RJ610_06160 | WND81946.1 | 0,30 | 5,27E-10 | | | GlsB/YeaQ/YmgE family stress response membrane protein |
| RJ610_06165 | WND81947.1 | 2,15 | 3,06E-04 | | | lipocalin family protein |
| RJ610_06170 | WND81948.1 | 1,10 | 7,21E-01 | | | cyclopropane-fatty-acyl-phospholipid synthase family protein |
| RJ610_06175 | WND83189.1 | 1,08 | 8,64E-01 | | | DUF1295 domain-containing protein |
| RJ610_06180 | WND81949.1 | 1,21 | 7,29E-01 | | | DUF2878 domain-containing protein |
| RJ610_06185 | WND83190.1 | 1,19 | 5,49E-01 | | | cyclopropane-fatty-acyl-phospholipid synthase family protein |
| RJ610_06190 | WND81950.1 | 3,31 | 1,83E-03 | | | DUF1365 domain-containing protein |
| RJ610_06195 | WND81951.1 | 1,74 | 1,20E-02 | | | FAD-dependent oxidoreductase |
| RJ610_06200 | WND83191.1 | 1,64 | 9,33E-03 | | | acyl-CoA desaturase |
| RJ610_06205 | WND81952.1 | 1,67 | 1,99E-01 | | | hypothetical protein |
| RJ610_06210 | WND81953.1 | 2,04 | 8,72E-03 | | | outer membrane beta-barrel protein |
| RJ610_06220 | WND81955.1 | 2,46 | 9,58E-12 | | | ComEA family DNA-binding protein |
| RJ610_06225 | WND81956.1 | 0,64 | 4,23E-04 | | | HutD family protein |
| RJ610_06230 | WND81957.1 | 0,64 | 3,33E-04 | | | Imm1 family immunity protein |
| RJ610_06235 | WND81958.1 | 0,52 | 1,27E-07 | | | sulfite reductase subunit alpha |
| RJ610_06240 | WND81959.1 | 0,61 | 2,59E-06 | | | FAD:protein FMN transferase |
| RJ610_06255 | WND83192.1 | 0,52 | 5,85E-09 | | | PepSY-associated TM helix domain-containing protein |
| RJ610_06270 | WND81964.1 | 0,65 | 1,98E-03 | | | non-heme iron oxygenase ferredoxin subunit |
| RJ610_06275 | WND81965.1 | 0,56 | 5,60E-08 | | | GNAT family N-acetyltransferase |

| | | | | | | |
|---|---|---|---|---|---|---|
| RJ610_06280 | WND81966.1 | 0,46 | 1,46E-14 | | | cysteine desulfurase |
| RJ610_06285 | WND81967.1 | 0,50 | 1,92E-09 | | | Fe-S cluster assembly protein SufD |
| RJ610_06290 | WND83193.1 | 0,50 | 8,15E-08 | | | Fe-S cluster assembly ATPase SufC |
| RJ610_06295 | WND81968.1 | 0,47 | 8,98E-09 | | | hypothetical protein |
| RJ610_06300 | WND81969.1 | 0,53 | 6,19E-08 | | | Fe-S cluster assembly protein SufB |
| RJ610_06305 | WND81970.1 | 0,78 | 7,19E-02 | | | SUF system Fe-S cluster assembly regulator |
| RJ610_06310 | | 0,58 | 1,32E-05 | | | SET domain-containing protein-lysine N-methyltransferase |
| RJ610_06315 | WND81971.1 | 0,84 | 3,40E-01 | | | 3-hydroxybutyrate dehydrogenase |
| RJ610_06320 | WND81972.1 | 1,24 | 6,49E-02 | | | DUF3734 domain-containing protein |
| RJ610_06325 | WND81973.1 | 0,67 | 1,58E-02 | | | DUF5916 domain-containing protein |
| RJ610_06335 | WND81975.1 | 28,92 | 6,78E-129 | | | TonB-dependent receptor |
| RJ610_06340 | WND81976.1 | 2,76 | 2,03E-07 | | | DMT family transporter |
| RJ610_06345 | WND81977.1 | 0,89 | 3,83E-01 | | | AraC family transcriptional regulator |
| RJ610_06350 | WND81978.1 | 0,70 | 9,98E-02 | | | PIN domain-containing protein |
| RJ610_06355 | WND81979.1 | 0,57 | 5,31E-07 | | | hypothetical protein |
| RJ610_06360 | WND81980.1 | 0,58 | 2,93E-05 | | | hypothetical protein |
| RJ610_06365 | WND81981.1 | 1,16 | 4,75E-01 | | | hypothetical protein |
| RJ610_06370 | WND81982.1 | 1,20 | 4,01E-01 | | | tellurite resistance TerB family protein |
| RJ610_06375 | WND81983.1 | 0,59 | 5,92E-04 | | | LysE family translocator |
| RJ610_06380 | WND81984.1 | 0,78 | 2,41E-02 | | | thioredoxin family protein |
| RJ610_06390 | WND81986.1 | 1,15 | 1,67E-01 | | | mechanosensitive ion channel |
| RJ610_06395 | WND81987.1 | 1,26 | 4,39E-02 | | | DUF4087 domain-containing protein |
| RJ610_06400 | WND81988.1 | 2,30 | 4,41E-11 | | | elongation factor G |
| RJ610_06410 | WND81990.1 | 1,91 | 3,94E-05 | | | alpha/beta fold hydrolase |
| RJ610_06415 | WND81991.1 | 1,25 | 1,88E-01 | | | hypothetical protein |
| RJ610_06420 | WND81992.1 | 0,79 | 1,29E-01 | | | Crp/Fnr family transcriptional regulator |
| RJ610_06425 | WND81993.1 | 0,66 | 8,49E-03 | | | class II aldolase/adducin family protein |
| RJ610_06430 | WND81994.1 | 1,24 | 1,48E-01 | | | bile acid:sodium symporter family protein |
| RJ610_06440 | WND81996.1 | 0,67 | 1,14E-03 | | | bifunctional aspartate kinase/diaminopimelate decarboxylase |
| RJ610_06445 | | 0,66 | 1,67E-02 | | | endonuclease domain-containing protein |
| RJ610_06450 | | 0,66 | 2,31E-02 | | | endonuclease domain-containing protein |
| RJ610_06455 | WND81997.1 | 0,78 | 2,27E-01 | | | UDP-N-acetylmuramoyl-L-alanine--D-glutamate ligase |
| RJ610_06460 | WND81998.1 | 0,85 | 3,51E-01 | | | dienelactone hydrolase family protein |
| RJ610_06465 | WND81999.1 | 0,94 | 5,75E-01 | | | polyprenyl synthetase family protein |
| RJ610_06470 | WND82000.1 | 0,17 | 1,58E-28 | | | Ig-like domain-containing protein |
| RJ610_06475 | WND82001.1 | 0,19 | 1,09E-21 | | | hypothetical protein |
| RJ610_06480 | WND82002.1 | 0,90 | 4,60E-01 | | | hypothetical protein |
| RJ610_06485 | WND82003.1 | 1,11 | 5,11E-01 | | | hypothetical protein |
| RJ610_06490 | WND82004.1 | 1,27 | 5,09E-01 | | | hypothetical protein |
| RJ610_06495 | WND82005.1 | 1,08 | 5,67E-01 | | | hypothetical protein |
| RJ610_06500 | WND82006.1 | 1,08 | 7,47E-01 | | | hypothetical protein |
| RJ610_06505 | WND82007.1 | 1,25 | 2,06E-01 | | | hypothetical protein |
| RJ610_06510 | WND82008.1 | 1,61 | 1,63E-03 | | | hypothetical protein |

| | | | | | | |
|---|---|---|---|---|---|---|
| RJ610_06515 | WND82009.1 | 1,56 | 6,38E-03 | | | DUF4926 domain-containing protein |
| RJ610_06520 | WND82010.1 | 1,81 | 1,31E-03 | | | hypothetical protein |
| RJ610_06525 | WND82011.1 | 1,49 | 2,53E-02 | | | hypothetical protein |
| RJ610_06530 | WND82012.1 | 1,18 | 4,57E-01 | | | hypothetical protein |
| RJ610_06535 | WND82013.1 | 1,59 | 1,63E-02 | | | transposase |
| RJ610_06540 | WND82014.1 | 1,02 | 9,43E-01 | | | hypothetical protein |
| RJ610_06545 | WND82015.1 | 0,85 | 6,08E-01 | | | hypothetical protein |
| RJ610_06550 | WND82016.1 | 0,85 | 2,57E-01 | | | transposase |
| RJ610_06555 | WND82017.1 | 0,69 | 1,48E-01 | | | hypothetical protein |
| RJ610_06560 | WND82018.1 | 0,21 | 1,03E-35 | | | putative Ig domain-containing protein |
| RJ610_06565 | WND82019.1 | 0,62 | 3,03E-04 | | | hypothetical protein |
| RJ610_06570 | WND82020.1 | 1,28 | 1,30E-01 | | | hypothetical protein |
| RJ610_06575 | WND82021.1 | 0,10 | 3,41E-53 | | | tetratricopeptide repeat protein |
| RJ610_06580 | WND82022.1 | 0,16 | 1,20E-17 | | | flagellar biosynthetic protein FliQ |
| RJ610_06585 | WND82023.1 | 0,14 | 2,35E-16 | | | flagellar biosynthetic protein FliR |
| RJ610_06590 | WND82024.1 | 0,25 | 4,41E-08 | | | EscU/YscU/HrcU family type III secretion system export apparatus switch protein |
| RJ610_06595 | WND82025.1 | 0,26 | 2,86E-13 | | | flagellar biosynthesis protein FlhA |
| RJ610_06600 | WND82026.1 | 0,22 | 4,06E-11 | | | flagellar hook basal-body protein |
| RJ610_06605 | WND82027.1 | 0,06 | 4,24E-17 | | | flagellar basal-body rod protein FlgG |
| RJ610_06610 | WND82028.1 | 0,09 | 3,13E-14 | | | flagellar basal body P-ring formation chaperone FlgA |
| RJ610_06615 | WND82029.1 | 0,08 | 8,64E-10 | | | flagellar basal body L-ring protein FlgH |
| RJ610_06620 | WND82030.1 | 0,10 | 1,80E-20 | | | flagellar basal body P-ring protein FlgI |
| RJ610_06625 | WND82031.1 | 0,15 | 6,11E-12 | | | hypothetical protein |
| RJ610_06630 | WND82032.1 | 0,12 | 3,48E-16 | | | flagellar basal body rod C-terminal domain-containing protein |
| RJ610_06635 | WND82033.1 | 0,12 | 3,49E-06 | | | flagellar hook-basal body complex protein FliE |
| RJ610_06640 | WND82034.1 | 0,10 | 4,19E-29 | | | flagellar basal-body MS-ring/collar protein FliF |
| RJ610_06645 | WND82035.1 | 0,10 | 5,43E-04 | | | hypothetical protein |
| RJ610_06650 | WND82036.1 | 0,20 | 2,02E-08 | | | FliH/SctL family protein |
| RJ610_06655 | WND82037.1 | 0,22 | 5,98E-12 | | | FliI/YscN family ATPase |
| RJ610_06660 | WND82038.1 | 0,19 | 1,72E-04 | | | hypothetical protein |
| RJ610_06665 | WND82039.1 | 0,18 | 6,45E-12 | | | hypothetical protein |
| RJ610_06670 | WND82040.1 | 0,38 | 4,59E-07 | | | flagellar hook capping FlgD N-terminal domain-containing protein |
| RJ610_06675 | WND82041.1 | 0,24 | 4,79E-18 | | | flagellar basal-body rod protein FlgF |
| RJ610_06680 | WND82042.1 | 0,18 | 8,18E-13 | | | FliM/FliN family flagellar motor C-terminal domain-containing protein |
| RJ610_06685 | WND82043.1 | 0,19 | 1,65E-10 | | | FliM/FliN family flagellar motor C-terminal domain-containing protein |
| RJ610_06690 | WND82044.1 | 0,16 | 1,27E-06 | | | hypothetical protein |
| RJ610_06695 | WND82045.1 | 0,37 | 1,17E-08 | | | flagellar type III secretion system pore protein FliP |
| RJ610_06700 | WND82046.1 | 0,83 | 3,43E-01 | | | sigma-70 family RNA polymerase sigma factor |
| RJ610_06710 | WND82048.1 | 3,35 | 6,63E-09 | | | universal stress protein |
| RJ610_06715 | WND82049.1 | 3,75 | 1,19E-11 | | | GNAT family N-acetyltransferase |

| | | | | | | |
|---|---|---|---|---|---|---|
| RJ610_06720 | WND82050.1 | 5,61 | 1,54E-22 | | | low affinity iron permease family protein |
| RJ610_06725 | WND82051.1 | 4,98 | 2,43E-16 | | | DUF4010 domain-containing protein |
| RJ610_06730 | WND82052.1 | 3,61 | 6,86E-11 | | | zinc-dependent alcohol dehydrogenase |
| RJ610_06735 | WND82053.1 | 1,78 | 7,49E-03 | | | response regulator |
| RJ610_06740 | WND82054.1 | 2,78 | 5,78E-08 | | | PAS domain S-box protein |
| RJ610_06745 | WND82055.1 | 1,79 | 2,31E-04 | | | helix-turn-helix domain-containing protein |
| RJ610_06750 | WND82056.1 | 3,85 | 1,92E-13 | | | hypothetical protein |
| RJ610_06755 | WND82057.1 | 5,12 | 2,57E-19 | | | hypothetical protein |
| RJ610_06760 | WND82058.1 | 4,46 | 7,71E-16 | | | universal stress protein |
| RJ610_06765 | WND82059.1 | 4,56 | 1,02E-13 | | | BON domain-containing protein |
| RJ610_06770 | WND82060.1 | 5,19 | 2,54E-17 | | | hypothetical protein |
| RJ610_06780 | WND82062.1 | 5,13 | 2,13E-17 | | | cytochrome d ubiquinol oxidase subunit II |
| RJ610_06785 | WND82063.1 | 5,20 | 1,41E-15 | | | cytochrome bd-I oxidase subunit CydX |
| RJ610_06790 | WND82064.1 | 5,78 | 1,54E-19 | | | cyd operon YbgE family protein |
| RJ610_06795 | WND82065.1 | 3,76 | 1,36E-11 | | | universal stress protein |
| RJ610_06800 | WND82066.1 | 4,86 | 3,25E-16 | | | DUF1857 family protein |
| RJ610_06805 | WND82067.1 | 5,48 | 7,47E-23 | | | NmrA/HSCARG family protein |
| RJ610_06810 | WND82068.1 | 4,67 | 1,02E-17 | | | LysR substrate-binding domain-containing protein |
| RJ610_06815 | WND82069.1 | 1,47 | 5,01E-03 | | | TetR/AcrR family transcriptional regulator |
| RJ610_06820 | WND82070.1 | 1,24 | 1,98E-01 | | | oxidoreductase |
| RJ610_06825 | WND82071.1 | 1,68 | 1,24E-05 | | | NADP-dependent oxidoreductase |
| RJ610_06830 | WND83194.1 | 2,11 | 2,18E-08 | | | MFS transporter |
| RJ610_06835 | WND82072.1 | 0,86 | 5,07E-01 | | | alpha/beta hydrolase |
| RJ610_06840 | WND82073.1 | 0,94 | 7,84E-01 | | | NAD(P)-dependent alcohol dehydrogenase |
| RJ610_06845 | WND82074.1 | 1,33 | 6,02E-02 | | | LysR family transcriptional regulator |
| RJ610_06850 | WND82075.1 | 1,12 | 5,27E-01 | | | NAD(P)H-binding protein |
| RJ610_06855 | WND82076.1 | 2,18 | 6,09E-07 | | | DoxX family protein |
| RJ610_06860 | WND82077.1 | 1,73 | 4,67E-06 | | | helix-turn-helix domain-containing protein |
| RJ610_06875 | WND82080.1 | 2,55 | 1,49E-05 | | | hypothetical protein |
| RJ610_06880 | WND82081.1 | 2,15 | 2,76E-08 | | | substrate-binding domain-containing protein |
| RJ610_06885 | WND82082.1 | 1,36 | 1,33E-01 | | | cupin domain-containing protein |
| RJ610_06890 | WND82083.1 | 0,99 | 9,70E-01 | | | 2-dehydro-3-deoxy-D-gluconate 5-dehydrogenase KduD |
| RJ610_06895 | WND82084.1 | 0,72 | 3,87E-02 | | | 5-dehydro-4-deoxy-D-glucuronate isomerase |
| RJ610_06900 | WND82085.1 | 0,86 | 1,96E-01 | | | LacI family DNA-binding transcriptional regulator |
| RJ610_06905 | WND82086.1 | 0,79 | 2,87E-01 | | | TRAP transporter large permease |
| RJ610_06910 | WND82087.1 | 1,01 | 9,79E-01 | | | TRAP transporter small permease |
| RJ610_06915 | WND82088.1 | 0,78 | 3,94E-01 | | | TRAP transporter substrate-binding protein |
| RJ610_06920 | WND82089.1 | 0,56 | 8,62E-03 | | | 2-keto-4-pentenoate hydratase |
| RJ610_06925 | WND82090.1 | 0,82 | 2,43E-01 | | | hypothetical protein |
| RJ610_06930 | WND82091.1 | 0,79 | 2,88E-01 | | | carboxylesterase/lipase family protein |
| RJ610_06935 | WND82092.1 | 1,48 | 8,46E-02 | | | rhamnogalacturonan acetylesterase |
| RJ610_06940 | WND82093.1 | 1,35 | 6,91E-02 | | | TonB-dependent receptor |
| RJ610_06945 | WND82094.1 | 1,08 | 7,52E-01 | | | sugar kinase |

| | | | | | | |
|---|---|---|---|---|---|---|
| RJ610_06950 | WND82095.1 | 1,32 | 1,88E-01 | | | hypothetical protein |
| RJ610_06955 | WND82096.1 | 1,98 | 6,99E-04 | | | hypothetical protein |
| RJ610_06960 | WND82097.1 | 1,42 | 3,13E-01 | | | hypothetical protein |
| RJ610_06965 | WND82098.1 | 1,66 | 6,39E-03 | | | phospholipase D-like domain-containing protein |
| RJ610_06970 | WND82099.1 | 1,56 | 4,41E-03 | | | exodeoxyribonuclease III |
| RJ610_06975 | WND82100.1 | 1,23 | 3,06E-01 | | | ferritin-like domain-containing protein |
| RJ610_06980 | WND82101.1 | 1,34 | 2,68E-01 | | | SDR family oxidoreductase |
| RJ610_06985 | WND82102.1 | 1,30 | 3,39E-01 | | | hypothetical protein |
| RJ610_06990 | WND82103.1 | 1,48 | 3,60E-01 | | | hypothetical protein |
| RJ610_06995 | WND82104.1 | 1,73 | 5,58E-03 | | | hypothetical protein |
| RJ610_07000 | WND82105.1 | 1,07 | 6,99E-01 | | | N-acetylglucosamine-6-phosphate deacetylase |
| RJ610_07005 | WND83195.1 | 1,19 | 1,84E-01 | | | DeoR family transcriptional regulator |
| RJ610_07010 | WND82106.1 | 1,21 | 1,56E-01 | | | D-tagatose-bisphosphate aldolase, class II, non-catalytic subunit |
| RJ610_07015 | WND82107.1 | 1,21 | 2,95E-01 | | | ROK family protein |
| RJ610_07020 | WND82108.1 | 0,96 | 8,47E-01 | | | SIS domain-containing protein |
| RJ610_07025 | WND82109.1 | 1,13 | 5,10E-01 | | | glycoside hydrolase family 31 protein |
| RJ610_07030 | WND82110.1 | 2,66 | 5,16E-10 | | | TonB-dependent receptor |
| RJ610_07035 | WND82111.1 | 7,76 | 6,69E-06 | | | DUF2867 domain-containing protein |
| RJ610_07040 | WND82112.1 | 12,60 | 1,95E-09 | | | thioesterase domain-containing protein |
| RJ610_07050 | WND82114.1 | 3,05 | 3,10E-10 | | | SDR family NAD(P)-dependent oxidoreductase |
| RJ610_07055 | WND82115.1 | 1,79 | 8,55E-05 | | | hypothetical protein |
| RJ610_07060 | WND82116.1 | 3,72 | 3,93E-26 | | | dTDP-glucose 4,6-dehydratase |
| RJ610_07065 | WND82117.1 | 4,06 | 7,34E-28 | | | glucose-1-phosphate thymidylyltransferase RfbA |
| RJ610_07070 | WND82118.1 | 4,24 | 6,39E-34 | | | dTDP-4-dehydrorhamnose 3,5-epimerase |
| RJ610_07075 | WND82119.1 | 5,04 | 1,04E-37 | | | dTDP-4-dehydrorhamnose reductase |
| RJ610_07080 | WND82120.1 | 0,68 | 3,85E-03 | | | S9 family peptidase |
| RJ610_07085 | WND82121.1 | 0,88 | 3,18E-01 | | | oligopeptide:H+ symporter |
| RJ610_07090 | WND82122.1 | 0,94 | 6,71E-01 | | | oligopeptide transporter, OPT family |
| RJ610_07100 | WND82124.1 | 0,64 | 5,26E-03 | | | DUF819 family protein |
| RJ610_07105 | WND82125.1 | 1,24 | 1,42E-01 | | | YbdD/YjiX family protein |
| RJ610_07110 | WND82126.1 | 1,18 | 1,39E-01 | | | carbon starvation CstA family protein |
| RJ610_07115 | WND82127.1 | 0,87 | 2,03E-01 | | | magnesium/cobalt transporter CorA |
| RJ610_07120 | WND82128.1 | 1,05 | 7,63E-01 | | | DUF4105 domain-containing protein |
| RJ610_07125 | WND82129.1 | 0,91 | 4,07E-01 | | | transporter associated domain-containing protein |
| RJ610_07130 | WND82130.1 | 3,34 | 4,42E-24 | | | GspH/FimT family pseudopilin |
| RJ610_07135 | WND82131.1 | 5,29 | 3,90E-55 | | | hypothetical protein |
| RJ610_07140 | WND82132.1 | 5,27 | 9,73E-53 | | | rRNA maturation RNase YbeY |
| RJ610_07145 | WND82133.1 | 6,16 | 1,99E-73 | | | PhoH family protein |
| RJ610_07150 | WND82134.1 | 14,45 | 2,60E-36 | | | hypothetical protein |
| RJ610_07155 | WND82135.1 | 1,15 | 4,32E-01 | | | phosphatase PAP2 family protein |
| RJ610_07160 | WND82136.1 | 0,63 | 8,44E-03 | | | 3-oxoacyl-[acyl-carrier-protein] synthase III C-terminal domain-containing protein |
| RJ610_07165 | WND82137.1 | 0,71 | 2,00E-02 | | | NAD-dependent epimerase/dehydratase family protein |

| | | | | | | |
|---|---|---|---|---|---|---|
| RJ610_07170 | WND82138.1 | 0,52 | 6,41E-04 | | | MBL fold metallo-hydrolase |
| RJ610_07175 | WND82139.1 | 0,54 | 3,03E-05 | | | TetR/AcrR family transcriptional regulator |
| RJ610_07180 | WND82140.1 | 0,65 | 8,38E-03 | | | HlyD family efflux transporter periplasmic adaptor subunit |
| RJ610_07185 | WND83196.1 | 0,99 | 9,80E-01 | | | ABC transporter ATP-binding protein |
| RJ610_07190 | WND82141.1 | 0,84 | 3,06E-01 | | | ABC transporter permease |
| RJ610_07195 | WND82142.1 | 0,84 | 4,79E-01 | | | hypothetical protein |
| RJ610_07200 | WND82143.1 | 1,13 | 4,66E-01 | | | hypothetical protein |
| RJ610_07210 | WND82145.1 | 3,28 | 8,85E-09 | | | DUF1501 domain-containing protein |
| RJ610_07215 | WND82146.1 | 1,11 | 5,34E-01 | | | GNAT family N-acetyltransferase |
| RJ610_07220 | WND82147.1 | 1,01 | 9,33E-01 | | | tRNA (N6-isopentenyl adenosine(37)-C2)-methylthiotransferase MiaB |
| RJ610_07225 | WND82148.1 | 2,76 | 5,76E-22 | | | lytic transglycosylase domain-containing protein |
| RJ610_07235 | WND82150.1 | 0,75 | 1,09E-02 | | | cytochrome bc complex cytochrome b subunit |
| RJ610_07240 | WND83197.1 | 0,84 | 1,30E-01 | | | cytochrome c1 |
| RJ610_07245 | WND82151.1 | 1,45 | 1,37E-02 | | | glutathione S-transferase N-terminal domain-containing protein |
| RJ610_07250 | WND82152.1 | 1,19 | 1,85E-01 | | | ClpXP protease specificity-enhancing factor |
| RJ610_07255 | WND82153.1 | 1,76 | 5,91E-06 | | | DUF3301 domain-containing protein |
| RJ610_07265 | WND82155.1 | 0,64 | 3,96E-05 | | | Trm112 family protein |
| RJ610_07280 | WND82158.1 | 0,69 | 1,97E-02 | | | hypothetical protein |
| RJ610_07285 | WND82159.1 | 0,57 | 1,93E-04 | | | hypothetical protein |
| RJ610_07295 | WND82161.1 | 1,02 | 8,68E-01 | | | Grx4 family monothiol glutaredoxin |
| RJ610_07300 | WND82162.1 | 1,05 | 6,26E-01 | | | SDR family NAD(P)-dependent oxidoreductase |
| RJ610_07305 | WND82163.1 | 1,07 | 6,24E-01 | | | MarC family protein |
| RJ610_07320 | | 0,69 | 1,43E-01 | | | tRNA-Asn |
| RJ610_07325 | WND82166.1 | 1,25 | 1,81E-01 | | | GspH/FimT family pseudopilin |
| RJ610_07330 | WND82167.1 | 1,70 | 1,50E-05 | | | GspH/FimT family pseudopilin |
| RJ610_07335 | WND82168.1 | 1,72 | 2,23E-04 | | | type IV pilus modification protein PilV |
| RJ610_07340 | WND82169.1 | 1,59 | 6,67E-04 | | | PilW family protein |
| RJ610_07345 | WND82170.1 | 1,52 | 1,87E-03 | | | PilX N-terminal domain-containing pilus assembly protein |
| RJ610_07355 | WND82172.1 | 1,61 | 7,00E-04 | | | type IV pilin protein |
| RJ610_07360 | WND83198.1 | 0,59 | 3,55E-05 | | | excinuclease ABC subunit UvrB |
| RJ610_07365 | | 0,87 | 6,21E-01 | | | tRNA-Val |
| RJ610_07370 | WND82173.1 | 0,39 | 8,43E-07 | | | hypothetical protein |
| RJ610_07375 | WND82174.1 | 0,53 | 8,66E-08 | | | hypothetical protein |
| RJ610_07380 | WND82175.1 | 0,92 | 5,57E-01 | | | ABC transporter ATP-binding protein/permease |
| RJ610_07385 | WND82176.1 | 1,02 | 8,85E-01 | | | threonine--tRNA ligase |
| RJ610_07390 | WND83199.1 | 1,13 | 2,63E-01 | | | translation initiation factor IF-3 |
| RJ610_07395 | WND82177.1 | 0,79 | 8,62E-03 | | | 50S ribosomal protein L35 |
| RJ610_07400 | WND82178.1 | 0,73 | 1,87E-03 | | | 50S ribosomal protein L20 |
| RJ610_07405 | WND82179.1 | 0,90 | 4,07E-01 | | | hypothetical protein |
| RJ610_07410 | WND82180.1 | 1,12 | 2,97E-01 | | | phenylalanine--tRNA ligase subunit alpha |
| RJ610_07415 | WND82181.1 | 1,07 | 5,21E-01 | | | phenylalanine--tRNA ligase subunit beta |
| RJ610_07420 | WND82182.1 | 1,26 | 2,36E-02 | | | integration host factor subunit alpha |

| | | | | | | |
|---|---|---|---|---|---|---|
| RJ610_07425 | WND82183.1 | 1,37 | 1,40E-03 | | | MerR family transcriptional regulator |
| RJ610_07430 | | 1,34 | 2,41E-02 | | | tRNA-Pro |
| RJ610_07435 | WND82184.1 | 0,83 | 2,26E-01 | | | DUF2845 domain-containing protein |
| RJ610_07440 | WND82185.1 | 0,49 | 9,40E-03 | | | hypothetical protein |
| RJ610_07445 | WND82186.1 | 1,03 | 9,12E-01 | | | hypothetical protein |
| RJ610_07450 | WND82187.1 | 2,32 | 7,00E-12 | | | acyltransferase |
| RJ610_07455 | WND82188.1 | 2,10 | 3,01E-10 | | | TraB/GumN family protein |
| RJ610_07460 | WND82189.1 | 0,32 | 1,82E-28 | | | hypothetical protein |
| RJ610_07465 | WND82190.1 | 0,33 | 6,39E-28 | | | PepSY-associated TM helix domain-containing protein |
| RJ610_07470 | WND82191.1 | 0,41 | 3,18E-09 | | | DUF3325 domain-containing protein |
| RJ610_07475 | WND82192.1 | 0,50 | 3,31E-04 | | | hypothetical protein |
| RJ610_07480 | WND82193.1 | 1,00 | 9,92E-01 | | | 1-deoxy-D-xylulose-5-phosphate synthase |
| RJ610_07485 | WND82194.1 | 1,64 | 1,80E-08 | | | hypothetical protein |
| RJ610_07490 | WND83200.1 | 0,95 | 6,59E-01 | | | HNH endonuclease |
| RJ610_07495 | WND82195.1 | 0,93 | 6,21E-01 | | | acyl-CoA dehydrogenase C-terminal domain-containing protein |
| RJ610_07500 | WND82196.1 | 0,81 | 8,46E-02 | | | LEA type 2 family protein |
| RJ610_07505 | WND82197.1 | 0,59 | 6,77E-05 | | | response regulator transcription factor |
| RJ610_07510 | WND82198.1 | 0,53 | 1,73E-06 | | | sensor histidine kinase |
| RJ610_07515 | WND82199.1 | 0,55 | 5,97E-09 | | | protein kinase |
| RJ610_07520 | WND82200.1 | 0,34 | 4,57E-23 | | | FHA domain-containing protein |
| RJ610_07535 | WND82203.1 | 0,33 | 6,58E-26 | | | hypothetical protein |
| RJ610_07540 | WND82204.1 | 0,45 | 3,50E-12 | | | DUF6491 family protein |
| RJ610_07545 | | 0,59 | 1,72E-02 | | | tRNA-Phe |
| RJ610_07550 | WND82205.1 | 0,42 | 1,66E-09 | | | N-acetyltransferase |
| RJ610_07555 | WND82206.1 | 10,16 | 7,98E-103 | | | hypothetical protein |
| RJ610_07560 | WND82207.1 | 1,13 | 3,49E-01 | | | oxidative damage protection protein |
| RJ610_07565 | WND82208.1 | 0,58 | 3,87E-05 | | | A/G-specific adenine glycosylase |
| RJ610_07570 | WND82209.1 | 0,75 | 6,79E-02 | | | hypothetical protein |
| RJ610_07575 | WND82210.1 | 0,63 | 7,04E-04 | | | signal recognition particle-docking protein FtsY |
| RJ610_07580 | WND82211.1 | 0,76 | 4,58E-02 | | | 16S rRNA (guanine(966)-N(2))-methyltransferase RsmD |
| RJ610_07585 | WND82212.1 | 0,60 | 1,22E-07 | | | pantetheine-phosphate adenylyltransferase |
| RJ610_07590 | WND82213.1 | 0,72 | 7,20E-03 | | | hypothetical protein |
| RJ610_07595 | WND82214.1 | 0,74 | 2,58E-02 | | | YfhL family 4Fe-4S dicluster ferredoxin |
| RJ610_07600 | WND82215.1 | 0,68 | 1,23E-03 | | | gamma-glutamyltransferase |
| RJ610_07605 | WND82216.1 | 0,37 | 1,24E-16 | | | hypothetical protein |
| RJ610_07610 | WND82217.1 | 0,66 | 1,01E-05 | | | MBL fold metallo-hydrolase |
| RJ610_07615 | WND82218.1 | 0,78 | 8,66E-02 | | | TMEM43 family protein |
| RJ610_07620 | WND82219.1 | 0,52 | 7,82E-08 | | | uracil phosphoribosyltransferase |
| RJ610_07625 | WND82220.1 | 0,57 | 6,78E-06 | | | hypothetical protein |
| RJ610_07630 | WND82221.1 | 0,73 | 3,07E-02 | | | hypothetical protein |
| RJ610_07640 | WND82223.1 | 0,63 | 3,34E-01 | | | hypothetical protein |
| RJ610_07645 | WND82224.1 | 0,57 | 5,05E-06 | | | sensor histidine kinase |
| RJ610_07650 | WND82225.1 | 0,62 | 5,14E-04 | | | response regulator transcription factor |

| | | | | | | |
|---|---|---|---|---|---|---|
| RJ610_07655 | WND82226.1 | 0,76 | 6,16E-02 | | | tRNA preQ1(34) S-adenosylmethionine ribosyltransferase-isomerase QueA |
| RJ610_07660 | WND82227.1 | 0,86 | 2,23E-01 | | | tRNA guanosine(34) transglycosylase Tgt |
| RJ610_07665 | WND82228.1 | 1,17 | 2,08E-01 | | | preprotein translocase subunit YajC |
| RJ610_07670 | WND82229.1 | 1,38 | 2,39E-02 | | | protein translocase subunit SecD |
| RJ610_07675 | WND82230.1 | 1,61 | 1,45E-04 | | | protein translocase subunit SecF |
| RJ610_07680 | WND82231.1 | 1,13 | 4,24E-01 | | | hypothetical protein |
| RJ610_07685 | WND82232.1 | 0,95 | 7,55E-01 | | | class I SAM-dependent methyltransferase |
| RJ610_07690 | WND82233.1 | 1,07 | 7,30E-01 | | | hypothetical protein |
| RJ610_07695 | WND82234.1 | 0,93 | 5,06E-01 | | | inositol monophosphatase family protein |
| RJ610_07700 | WND82235.1 | 0,72 | 8,78E-03 | | | RNA methyltransferase |
| RJ610_07705 | WND83201.1 | 0,67 | 2,75E-03 | | | phosphate/phosphite/phosphonate ABC transporter substrate-binding protein |
| RJ610_07710 | WND82236.1 | 0,67 | 1,68E-03 | | | EAL domain-containing protein |
| RJ610_07715 | WND82237.1 | 0,56 | 1,18E-04 | | | exopolysaccharide biosynthesis protein |
| RJ610_07720 | WND82238.1 | 0,41 | 2,18E-14 | | | hemolysin family protein |
| RJ610_07725 | WND82239.1 | 0,56 | 2,15E-10 | | | DUF47 family protein |
| RJ610_07730 | WND82240.1 | 0,58 | 1,59E-07 | | | inorganic phosphate transporter |
| RJ610_07735 | WND82241.1 | 0,71 | 1,32E-03 | | | hypothetical protein |
| RJ610_07740 | WND82242.1 | 1,03 | 8,67E-01 | | | MGMT family protein |
| RJ610_07745 | WND82243.1 | 0,71 | 5,59E-03 | | | rhomboid family intramembrane serine protease |
| RJ610_07750 | WND82244.1 | 1,09 | 6,10E-01 | | | hypothetical protein |
| RJ610_07755 | WND82245.1 | 0,97 | 8,33E-01 | | | tetratricopeptide repeat protein |
| RJ610_07760 | WND82246.1 | 0,85 | 2,58E-01 | | | hypothetical protein |
| RJ610_07765 | WND82247.1 | 0,67 | 9,94E-04 | | | ATP-binding protein |
| RJ610_07770 | WND82248.1 | 0,14 | 2,01E-50 | | | AAA family ATPase |
| RJ610_07775 | WND82249.1 | 0,23 | 8,80E-08 | | | hypothetical protein |
| RJ610_07780 | WND82250.1 | 0,17 | 1,35E-36 | | | hypothetical protein |
| RJ610_07785 | WND82251.1 | 0,17 | 1,65E-36 | | | bpX6 domain-containing protein |
| RJ610_07790 | WND82252.1 | 0,85 | 1,06E-01 | | | M13 family metallopeptidase |
| RJ610_07795 | WND82253.1 | 0,57 | 1,02E-04 | | | hypothetical protein |
| RJ610_07805 | WND82255.1 | 0,86 | 4,45E-01 | | | M13-type metalloendopeptidase |
| RJ610_07810 | WND82256.1 | 0,67 | 1,46E-02 | | | MBL fold metallo-hydrolase |
| RJ610_07815 | WND82257.1 | 0,91 | 6,28E-01 | | | type II secretion system protein GspG |
| RJ610_07820 | WND82258.1 | 0,97 | 9,07E-01 | | | hypothetical protein |
| RJ610_07825 | WND82259.1 | 0,79 | 5,94E-02 | | | BCCT family transporter |
| RJ610_07830 | WND82260.1 | 0,62 | 4,19E-05 | | | DUF3304 domain-containing protein |
| RJ610_07835 | WND82261.1 | 0,48 | 7,55E-10 | | | low temperature requirement protein A |
| RJ610_07840 | WND82262.1 | 0,55 | 1,09E-04 | | | hypothetical protein |
| RJ610_07845 | WND82263.1 | 0,39 | 1,03E-06 | | | SDR family oxidoreductase |
| RJ610_07850 | WND82264.1 | 0,15 | 2,32E-26 | | | AraC family transcriptional regulator |
| RJ610_07855 | WND82265.1 | 0,19 | 1,13E-28 | | | ABC transporter ATP-binding protein |
| RJ610_07860 | WND82266.1 | 0,21 | 4,61E-20 | | | M1 family aminopeptidase |

| | | | | | | |
|---|---|---|---|---|---|---|
| RJ610_07865 | WND82267.1 | 0,18 | 6,20E-61 | | | DEAD/DEAH box helicase |
| RJ610_07870 | WND82268.1 | 0,47 | 9,67E-10 | | | CHASE2 domain-containing protein |
| RJ610_07875 | WND82269.1 | 0,41 | 1,66E-23 | | | FecR domain-containing protein |
| RJ610_07880 | WND82270.1 | 0,71 | 1,23E-01 | | | DUF885 family protein |
| RJ610_07885 | WND82271.1 | 1,32 | 8,72E-02 | | | hypothetical protein |
| RJ610_07890 | WND82272.1 | 0,78 | 1,96E-01 | | | histidine kinase |
| RJ610_07895 | WND82273.1 | 0,62 | 1,83E-02 | | | LytTR family DNA-binding domain-containing protein |
| RJ610_07900 | WND82274.1 | 0,71 | 1,42E-02 | | | queuosine precursor transporter |
| RJ610_07910 | WND82276.1 | 0,61 | 1,34E-05 | | | DUF502 domain-containing protein |
| RJ610_07915 | WND82277.1 | 0,75 | 8,52E-03 | | | bifunctional serine/threonine-protein kinase/formylglycine-generating enzyme family protein |
| RJ610_07920 | WND82278.1 | 1,22 | 1,72E-01 | | | DUF4442 domain-containing protein |
| RJ610_07925 | WND82279.1 | 1,38 | 1,02E-01 | | | thioredoxin |
| RJ610_07930 | WND82280.1 | 2,95 | 9,88E-10 | | | DUF998 domain-containing protein |
| RJ610_07935 | WND82281.1 | 5,11 | 1,25E-29 | | | hypothetical protein |
| RJ610_07940 | WND82282.1 | 110,73 | 7,91E-109 | | | hypothetical protein |
| RJ610_07945 | WND82283.1 | 0,90 | 5,36E-01 | | | amidohydrolase family protein |
| RJ610_07950 | WND82284.1 | 1,25 | 7,62E-02 | | | hypothetical protein |
| RJ610_07955 | WND82285.1 | 1,84 | 1,32E-07 | | | hypothetical protein |
| RJ610_07960 | WND82286.1 | 0,42 | 5,40E-06 | | | hypothetical protein |
| RJ610_07965 | WND82287.1 | 0,75 | 5,31E-02 | | | diguanylate cyclase |
| RJ610_07970 | WND82288.1 | 0,77 | 8,67E-03 | | | leucine--tRNA ligase |
| RJ610_07975 | WND82289.1 | 1,06 | 7,10E-01 | | | glutathione S-transferase family protein |
| RJ610_07985 | WND82290.1 | 0,61 | 5,53E-06 | | | DNA polymerase III subunit delta |
| RJ610_07990 | WND82291.1 | 0,68 | 7,42E-03 | | | nicotinate-nucleotide adenylyltransferase |
| RJ610_07995 | WND82292.1 | 0,65 | 3,22E-06 | | | ribosome silencing factor |
| RJ610_08000 | WND82293.1 | 0,79 | 1,47E-01 | | | Bax inhibitor-1/YccA family protein |
| RJ610_08005 | | 1,09 | 5,17E-01 | | | tRNA-Pro |
| RJ610_08010 | | 1,12 | 3,73E-01 | | | tRNA-Arg |
| RJ610_08015 | | 1,23 | 8,97E-02 | | | tRNA-His |
| RJ610_08020 | | 1,94 | 1,53E-06 | | | tRNA-Lys |
| RJ610_08025 | WND82294.1 | 1,95 | 2,91E-12 | | | hypothetical protein |
| RJ610_08035 | WND82296.1 | 15,12 | 4,58E-39 | | | hypothetical protein |
| RJ610_08040 | WND82297.1 | 14,07 | 1,23E-60 | | | hypothetical protein |
| RJ610_08045 | WND82298.1 | 39,10 | 7,12E-86 | | | hypothetical protein |
| RJ610_08055 | | 1,27 | 7,77E-02 | | | tRNA-Leu |
| RJ610_08060 | WND82300.1 | 1,09 | 4,31E-01 | | | trigger factor |
| RJ610_08065 | WND82301.1 | 1,15 | 5,41E-01 | | | ATP-dependent Clp endopeptidase proteolytic subunit ClpP |
| RJ610_08070 | WND82302.1 | 1,00 | 9,82E-01 | | | ATP-dependent Clp protease ATP-binding subunit ClpX |
| RJ610_08075 | WND82303.1 | 1,15 | 5,11E-01 | | | endopeptidase La |
| RJ610_08080 | WND82304.1 | 0,79 | 4,24E-02 | | | HU family DNA-binding protein |
| RJ610_08085 | | 0,84 | 9,23E-02 | | | tRNA-Val |
| RJ610_08090 | | 0,84 | 1,68E-01 | | | tRNA-Asp |

| | | | | | | |
|---|---|---|---|---|---|---|
| RJ610_08095 | | 0,51 | 9,74E-08 | | | tRNA-Asp |
| RJ610_08100 | WND82305.1 | 1,15 | 2,05E-01 | | | peptidyl-prolyl cis-trans isomerase |
| RJ610_08105 | WND82306.1 | 1,42 | 1,47E-02 | | | hypothetical protein |
| RJ610_08110 | WND82307.1 | 0,41 | 4,78E-06 | | | 23S rRNA (pseudouridine(1915)-N(3))-methyltransferase RlmH |
| RJ610_08115 | WND82308.1 | 0,01 | 5,68E-143 | | | energy transducer TonB |
| RJ610_08120 | WND82309.1 | 0,98 | 9,17E-01 | | | hypothetical protein |
| RJ610_08130 | WND82310.1 | 1,18 | 1,53E-01 | | | Maf family protein |
| RJ610_08135 | WND82311.1 | 1,40 | 1,68E-03 | | | ribonuclease G |
| RJ610_08140 | WND82312.1 | 1,31 | 2,22E-02 | | | YhdP family protein |
| RJ610_08145 | WND82313.1 | 1,30 | 7,94E-02 | | | metalloprotease TldD |
| RJ610_08150 | WND82314.1 | 0,76 | 5,00E-03 | | | ribosome biogenesis factor YjgA |
| RJ610_08155 | WND83204.1 | 0,96 | 7,78E-01 | | | metalloprotease PmbA |
| RJ610_08160 | WND82315.1 | 1,44 | 9,55E-02 | | | DUF1800 domain-containing protein |
| RJ610_08165 | WND82316.1 | 2,92 | 3,90E-10 | | | DUF1501 domain-containing protein |
| RJ610_08170 | WND82317.1 | 0,63 | 2,88E-05 | | | DUF4870 domain-containing protein |
| RJ610_08175 | WND82318.1 | 7,07 | 2,56E-37 | | | (2E,6E)-farnesyl diphosphate synthase |
| RJ610_08180 | WND82319.1 | 5,77 | 1,34E-37 | | | exodeoxyribonuclease VII small subunit |
| RJ610_08185 | WND82320.1 | 0,76 | 7,48E-02 | | | tRNA lysidine(34) synthetase TilS |
| RJ610_08195 | WND82322.1 | 2,05 | 6,29E-08 | | | transglycosylase domain-containing protein |
| RJ610_08200 | WND82323.1 | 1,96 | 2,12E-08 | | | acetyl-CoA carboxylase carboxyltransferase subunit alpha |
| RJ610_08205 | WND82324.1 | 0,97 | 7,81E-01 | | | DNA polymerase III subunit alpha |
| RJ610_08210 | WND82325.1 | 7,49 | 2,05E-91 | | | hypothetical protein |
| RJ610_08215 | WND83205.1 | 1,14 | 3,71E-01 | | | ribonuclease HII |
| RJ610_08220 | WND82326.1 | 1,91 | 2,27E-06 | | | lipid-A-disaccharide synthase |
| RJ610_08225 | WND82327.1 | 0,92 | 6,01E-01 | | | hypothetical protein |
| RJ610_08230 | WND82328.1 | 0,76 | 9,25E-03 | | | acyl-ACP--UDP-N-acetylglucosamine O-acyltransferase |
| RJ610_08235 | WND82329.1 | 0,74 | 6,95E-03 | | | 3-hydroxyacyl-ACP dehydratase FabZ |
| RJ610_08240 | WND82330.1 | 0,79 | 3,08E-02 | | | UDP-3-O-(3-hydroxymyristoyl)glucosamine N-acyltransferase |
| RJ610_08250 | WND82332.1 | 0,88 | 3,56E-01 | | | RIP metalloprotease RseP |
| RJ610_08255 | WND82333.1 | 1,32 | 7,59E-02 | | | 1-deoxy-D-xylulose-5-phosphate reductoisomerase |
| RJ610_08260 | WND82334.1 | 1,11 | 4,49E-01 | | | phosphatidate cytidylyltransferase |
| RJ610_08265 | WND82335.1 | 1,17 | 2,46E-01 | | | polyprenyl diphosphate synthase |
| RJ610_08270 | WND82336.1 | 1,08 | 6,21E-01 | | | ribosome recycling factor |
| RJ610_08275 | WND82337.1 | 0,64 | 1,97E-03 | | | cation diffusion facilitator family transporter |
| RJ610_08280 | WND82338.1 | 0,81 | 3,07E-01 | | | BlaI/MecI/CopY family transcriptional regulator |
| RJ610_08285 | WND82339.1 | 0,93 | 5,82E-01 | | | M56 family metallopeptidase |
| RJ610_08290 | WND82340.1 | 0,81 | 1,30E-01 | | | UMP kinase |
| RJ610_08295 | WND82341.1 | 0,32 | 1,50E-20 | | | AIM24 family protein |
| RJ610_08300 | WND82342.1 | 0,46 | 2,13E-07 | | | hypothetical protein |
| RJ610_08305 | WND82343.1 | 0,47 | 2,61E-03 | | | hypothetical protein |
| RJ610_08310 | WND82344.1 | 0,87 | 5,39E-01 | | | translation elongation factor Ts |
| RJ610_08315 | WND82345.1 | 1,05 | 8,10E-01 | | | 30S ribosomal protein S2 |
| RJ610_08320 | WND82346.1 | 0,62 | 6,25E-04 | | | MBL fold metallo-hydrolase |

| | | | | | | |
|---|---|---|---|---|---|---|
| RJ610_08325 | WND82347.1 | 0,97 | 8,19E-01 | | | type I methionyl aminopeptidase |
| RJ610_08330 | WND82348.1 | 1,03 | 8,19E-01 | | | [protein-PII] uridylyltransferase |
| RJ610_08335 | WND83206.1 | 0,85 | 1,63E-01 | | | 2,3,4,5-tetrahydropyridine-2,6-dicarboxylate N-succinyltransferase |
| RJ610_08340 | WND82349.1 | 0,86 | 2,13E-01 | | | hypothetical protein |
| RJ610_08345 | WND82350.1 | 0,95 | 7,81E-01 | | | hypothetical protein |
| RJ610_08350 | WND82351.1 | 1,03 | 8,60E-01 | | | hypothetical protein |
| RJ610_08355 | WND82352.1 | 1,09 | 6,13E-01 | | | Spx/MgsR family RNA polymerase-binding regulatory protein |
| RJ610_08365 | WND82354.1 | 0,83 | 9,01E-02 | | | penicillin acylase family protein |
| RJ610_08370 | WND82355.1 | 1,12 | 6,20E-01 | | | hypothetical protein |
| RJ610_08375 | WND82356.1 | 1,07 | 9,18E-01 | | | asparagine synthase B |
| RJ610_08380 | WND82357.1 | 1,02 | 9,80E-01 | | | hypothetical protein |
| RJ610_08385 | WND82358.1 | 1,15 | 7,75E-01 | | | hypothetical protein |
| RJ610_08390 | WND82359.1 | 0,58 | 6,40E-06 | | | polysaccharide deacetylase family protein |
| RJ610_08395 | WND82360.1 | 0,55 | 1,65E-05 | | | tetratricopeptide repeat protein |
| RJ610_08400 | WND82361.1 | 1,07 | 6,10E-01 | | | monovalent cation:proton antiporter-2 (CPA2) family protein |
| RJ610_08405 | WND82362.1 | 1,02 | 9,38E-01 | | | hypothetical protein |
| RJ610_08410 | WND82363.1 | 0,68 | 7,79E-03 | | | hypothetical protein |
| RJ610_08415 | WND82364.1 | 0,73 | 6,91E-02 | | | CGNR zinc finger domain-containing protein |
| RJ610_08420 | WND83207.1 | 0,55 | 2,23E-04 | | | MBL fold metallo-hydrolase |
| RJ610_08430 | WND82366.1 | 1,35 | 5,26E-04 | | | ribonuclease R |
| RJ610_08435 | WND82367.1 | 0,90 | 3,71E-01 | | | 23S rRNA (guanosine(2251)-2'-O)-methyltransferase RlmB |
| RJ610_08440 | WND82368.1 | 0,88 | 3,85E-01 | | | DUF3592 domain-containing protein |
| RJ610_08445 | WND82369.1 | 0,86 | 3,41E-01 | | | quaternary ammonium compound efflux SMR transporter SugE |
| RJ610_08450 | WND82370.1 | 0,95 | 8,01E-01 | | | acetyltransferase |
| RJ610_08455 | WND82371.1 | 1,06 | 8,85E-01 | | | glycoside hydrolase family 19 protein |
| RJ610_08460 | WND82372.1 | 0,62 | 5,00E-04 | | | hypothetical protein |
| RJ610_08465 | WND82373.1 | 0,74 | 2,46E-02 | | | ribonuclease T |
| RJ610_08470 | WND82374.1 | 0,87 | 4,00E-01 | | | putative DNA-binding domain-containing protein |
| RJ610_08475 | WND82375.1 | 0,91 | 4,82E-01 | | | DUF692 domain-containing protein |
| RJ610_08480 | WND82376.1 | 0,74 | 8,39E-03 | | | hypothetical protein |
| RJ610_08485 | WND82377.1 | 1,22 | 1,22E-01 | | | sigma-70 family RNA polymerase sigma factor |
| RJ610_08490 | WND82378.1 | 0,66 | 2,54E-02 | | | phosphate signaling complex protein PhoU |
| RJ610_08495 | WND82379.1 | 0,75 | 1,56E-01 | | | phosphate ABC transporter ATP-binding protein PstB |
| RJ610_08500 | WND82380.1 | 0,54 | 2,94E-02 | | | phosphate ABC transporter permease PstA |
| RJ610_08505 | WND82381.1 | 0,44 | 9,41E-02 | | | phosphate ABC transporter permease subunit PstC |
| RJ610_08510 | WND82382.1 | 0,78 | 2,20E-01 | | | hypothetical protein |
| RJ610_08520 | WND83208.1 | 0,80 | 2,98E-01 | | | phosphate ABC transporter substrate-binding protein PstS |
| RJ610_08525 | WND82384.1 | 0,80 | 2,82E-01 | | | OprO/OprP family phosphate-selective porin |
| RJ610_08530 | WND83209.1 | 2,67 | 3,11E-12 | | | endonuclease III |
| RJ610_08535 | WND82385.1 | 1,50 | 6,11E-03 | | | hypothetical protein |
| RJ610_08545 | WND82387.1 | 1,47 | 3,51E-04 | | | FKBP-type peptidyl-prolyl cis-trans isomerase N-terminal domain-containing protein |
| RJ610_08550 | | 1,69 | 1,90E-04 | | | DUF1289 domain-containing protein |

| | | | | | | |
|---|---|---|---|---|---|---|
| RJ610_08555 | WND82388.1 | 2,21 | 1,43E-07 | | | sulfurtransferase |
| RJ610_08560 | WND82389.1 | 1,60 | 1,79E-03 | | | substrate-binding domain-containing protein |
| RJ610_08565 | WND82390.1 | 0,92 | 5,86E-01 | | | enoyl-CoA hydratase/isomerase family protein |
| RJ610_08575 | WND82392.1 | 1,48 | 2,30E-03 | | | copper chaperone PCu(A)C |
| RJ610_08580 | WND82393.1 | 1,57 | 3,23E-03 | | | thioesterase family protein |
| RJ610_08585 | WND82394.1 | 1,15 | 3,29E-01 | | | excinuclease ABC subunit UvrA |
| RJ610_08590 | WND82395.1 | 4,44 | 2,47E-19 | | | OmpA family protein |
| RJ610_08600 | WND82397.1 | 3,60 | 1,19E-08 | | | hypothetical protein |
| RJ610_08605 | WND82398.1 | 0,87 | 2,60E-01 | | | 50S ribosomal protein L21 |
| RJ610_08610 | WND82399.1 | 0,87 | 2,09E-01 | | | 50S ribosomal protein L27 |
| RJ610_08615 | WND82400.1 | 0,75 | 1,62E-02 | | | GTPase ObgE |
| RJ610_08620 | WND82401.1 | 0,65 | 6,13E-03 | | | hypothetical protein |
| RJ610_08625 | WND82402.1 | 0,78 | 6,83E-02 | | | 30S ribosomal protein S20 |
| RJ610_08630 | WND82403.1 | 1,08 | 5,84E-01 | | | murein biosynthesis integral membrane protein MurJ |
| RJ610_08635 | | 1,03 | 8,39E-01 | | | bifunctional riboflavin kinase/FAD synthetase |
| RJ610_08640 | WND83210.1 | 0,89 | 3,08E-01 | | | isoleucine--tRNA ligase |
| RJ610_08645 | WND83211.1 | 0,80 | 6,89E-02 | | | signal peptidase II |
| RJ610_08650 | WND82404.1 | 0,81 | 4,10E-02 | | | 4-hydroxy-3-methylbut-2-enyl diphosphate reductase |
| RJ610_08655 | | 0,81 | 8,04E-02 | | | tRNA-Thr |
| RJ610_08660 | | 0,75 | 2,58E-02 | | | tRNA-Thr |
| RJ610_08665 | WND82405.1 | 0,65 | 6,25E-06 | | | hypothetical protein |
| RJ610_08670 | WND82406.1 | 0,82 | 6,45E-02 | | | DNA adenine methylase |
| RJ610_08675 | WND82407.1 | 0,80 | 3,69E-01 | | | helix-turn-helix domain-containing protein |
| RJ610_08680 | WND82408.1 | 0,80 | 4,35E-01 | | | hypothetical protein |
| RJ610_08685 | WND82409.1 | 0,79 | 5,46E-01 | | | hypothetical protein |
| RJ610_08690 | WND82410.1 | 0,55 | 2,88E-01 | | | hypothetical protein |
| RJ610_08695 | WND82411.1 | 1,13 | 7,14E-01 | | | hypothetical protein |
| RJ610_08700 | WND82412.1 | 0,95 | 7,79E-01 | | | hypothetical protein |
| RJ610_08705 | WND82413.1 | 1,03 | 8,27E-01 | | | hypothetical protein |
| RJ610_08710 | WND82414.1 | 0,85 | 3,92E-01 | | | tyrosine-type recombinase/integrase |
| RJ610_08715 | WND82415.1 | 0,56 | 1,30E-07 | | | hypothetical protein |
| RJ610_08720 | WND82416.1 | 0,74 | 6,58E-02 | | | hypothetical protein |
| RJ610_08725 | WND82417.1 | 0,76 | 5,21E-01 | | | RebB family R body protein |
| RJ610_08730 | WND82418.1 | 2,02 | 1,84E-02 | | | hypothetical protein |
| RJ610_08735 | WND82419.1 | 0,38 | 5,66E-15 | | | hypothetical protein |
| RJ610_08740 | WND83212.1 | 0,52 | 6,47E-04 | | | DNA repair protein RadA |
| RJ610_08745 | WND82420.1 | 0,73 | 1,91E-01 | | | hypothetical protein |
| RJ610_08750 | WND82421.1 | 3,28 | 3,16E-09 | | | glutaredoxin family protein |
| RJ610_08755 | WND82422.1 | 6,94 | 3,94E-30 | | | hypothetical protein |
| RJ610_08760 | WND82423.1 | 22,72 | 3,36E-41 | | | hypothetical protein |
| RJ610_08765 | WND82424.1 | 12,57 | 5,81E-36 | | | hypothetical protein |
| RJ610_08770 | WND82425.1 | 288,58 | 5,38E-218 | | | enoyl-CoA hydratase-related protein |
| RJ610_08775 | WND82426.1 | 64,61 | 2,20E-43 | | | hypothetical protein |

| | | | | | | |
|---|---|---|---|---|---|---|
| RJ610_08780 | WND82427.1 | 2,20 | 5,09E-08 | | | hypothetical protein |
| RJ610_08785 | WND82428.1 | 1,81 | 2,39E-06 | | | DHA2 family efflux MFS transporter permease subunit |
| RJ610_08790 | WND82429.1 | 1,65 | 3,21E-04 | | | efflux RND transporter periplasmic adaptor subunit |
| RJ610_08795 | WND82430.1 | 1,85 | 2,14E-07 | | | efflux transporter outer membrane subunit |
| RJ610_08800 | WND82431.1 | 1,53 | 1,16E-03 | | | MarR family transcriptional regulator |
| RJ610_08805 | WND82432.1 | 0,94 | 6,03E-01 | | | cytochrome c biogenesis protein CcsA |
| RJ610_08810 | WND82433.1 | 0,76 | 8,79E-03 | | | signal recognition particle protein |
| RJ610_08815 | WND82434.1 | 0,80 | 4,96E-02 | | | 30S ribosomal protein S16 |
| RJ610_08820 | WND82435.1 | 0,85 | 2,51E-01 | | | ribosome maturation factor RimM |
| RJ610_08825 | WND82436.1 | 1,01 | 9,60E-01 | | | hypothetical protein |
| RJ610_08830 | WND82437.1 | 0,90 | 4,99E-01 | | | tRNA (guanosine(37)-N1)-methyltransferase TrmD |
| RJ610_08840 | WND82439.1 | 0,68 | 1,61E-02 | | | hypothetical protein |
| RJ610_08845 | WND82440.1 | 0,86 | 3,04E-01 | | | hypothetical protein |
| RJ610_08850 | WND82441.1 | 0,87 | 4,38E-01 | | | MATE family efflux transporter |
| RJ610_08855 | WND82442.1 | 0,91 | 5,84E-01 | | | RNA-binding S4 domain-containing protein |
| RJ610_08860 | WND82443.1 | 0,65 | 1,40E-02 | | | DUF937 domain-containing protein |
| RJ610_08865 | WND82444.1 | 3,68 | 3,15E-32 | | | catalase/peroxidase HPI |
| RJ610_08870 | WND82445.1 | 1,78 | 1,44E-04 | | | hypothetical protein |
| RJ610_08875 | WND82446.1 | 3,30 | 3,72E-06 | | | hypothetical protein |
| RJ610_08880 | WND82447.1 | 1,75 | 2,01E-06 | | | AAA family ATPase |
| RJ610_08885 | WND83213.1 | 0,94 | 5,33E-01 | | | DNA mismatch repair protein MutS |
| RJ610_08890 | WND82448.1 | 0,95 | 7,61E-01 | | | hypothetical protein |
| RJ610_08895 | WND82449.1 | 1,18 | 2,82E-01 | | | hypothetical protein |
| RJ610_08900 | WND82450.1 | 1,40 | 1,60E-02 | | | hypothetical protein |
| RJ610_08905 | WND82451.1 | 9,05 | 1,59E-45 | | | hypothetical protein |
| RJ610_08910 | WND82452.1 | 6,85 | 9,58E-14 | | | hypothetical protein |
| RJ610_08915 | WND82453.1 | 3,02 | 2,29E-06 | | | apolipoprotein N-acyltransferase |
| RJ610_08920 | WND82454.1 | 1,69 | 1,47E-03 | | | serine hydrolase domain-containing protein |
| RJ610_08925 | WND82455.1 | 6,06 | 4,30E-16 | | | amino acid permease |
| RJ610_08930 | WND82456.1 | 8,50 | 4,01E-24 | | | hypothetical protein |
| RJ610_08935 | WND82457.1 | 82,01 | 7,13E-102 | | | MFS transporter |
| RJ610_08940 | WND82458.1 | 509,10 | 2,09E-219 | | | NADP-dependent oxidoreductase |
| RJ610_08945 | WND82459.1 | 560,48 | 3,09E-241 | | | NAD(P)/FAD-dependent oxidoreductase |
| RJ610_08950 | WND82460.1 | 637,07 | 3,98E-186 | | | NAD(P)/FAD-dependent oxidoreductase |
| RJ610_08955 | WND82461.1 | 822,76 | 4,28E-242 | | | NAD(P)/FAD-dependent oxidoreductase |
| RJ610_08960 | WND82462.1 | 1118,94 | 0,00E+00 | | | HSAF biosynthetic non-ribosomal peptide synthetase/polyketide synthase |
| RJ610_08965 | WND82463.1 | 478,90 | 6,57E-143 | | | sterol desaturase family protein |
| RJ610_08970 | WND82464.1 | 1533,43 | 2,30E-33 | | | hypothetical protein |
| RJ610_08975 | WND82465.1 | 606,63 | 4,56E-159 | | | ferredoxin--NADP reductase |
| RJ610_08980 | WND82466.1 | 186,71 | 6,00E-130 | | | arginase |
| RJ610_08985 | WND82467.1 | 1,26 | 1,86E-01 | | | TonB-dependent receptor |
| RJ610_08990 | WND83214.1 | 0,86 | 3,12E-01 | | | CinA family protein |

| | | | | | | |
|---|---|---|---|---|---|---|
| RJ610_08995 | WND82468.1 | 1,00 | 9,85E-01 | | | helix-turn-helix domain-containing GNAT family N-acetyltransferase |
| RJ610_09000 | WND82469.1 | 0,43 | 7,36E-13 | | | transcriptional repressor LexA |
| RJ610_09005 | WND82470.1 | 0,37 | 9,29E-14 | | | hypothetical protein |
| RJ610_09010 | WND82471.1 | 0,43 | 1,51E-06 | | | recombinase RecA |
| RJ610_09015 | | 0,53 | 8,19E-04 | | | recombination regulator RecX |
| RJ610_09020 | WND82472.1 | 0,76 | 7,26E-02 | | | alanine--tRNA ligase |
| RJ610_09025 | WND82473.1 | 2,42 | 2,66E-13 | | | carbon storage regulator CsrA |
| RJ610_09030 | | 2,04 | 1,67E-11 | | | tRNA-Ser |
| RJ610_09035 | | 1,74 | 3,93E-07 | | | tRNA-Arg |
| RJ610_09040 | WND82474.1 | 0,73 | 3,64E-01 | | | hypothetical protein |
| RJ610_09045 | WND82475.1 | 1,29 | 6,67E-02 | | | response regulator |
| RJ610_09050 | WND82476.1 | 1,37 | 4,29E-02 | | | sensor histidine kinase KdpD |
| RJ610_09055 | WND82477.1 | 1,03 | 9,15E-01 | | | potassium-transporting ATPase subunit KdpC |
| RJ610_09060 | WND83215.1 | 0,87 | 5,07E-01 | | | potassium-transporting ATPase subunit KdpB |
| RJ610_09065 | WND82478.1 | 0,71 | 1,66E-01 | | | potassium-transporting ATPase subunit KdpA |
| RJ610_09070 | WND82479.1 | 0,35 | 6,50E-03 | | | potassium-transporting ATPase subunit F |
| RJ610_09075 | WND82480.1 | 0,39 | 7,62E-04 | | | TorF family putative porin |
| RJ610_09080 | WND82481.1 | 7,38 | 1,91E-02 | | | hypothetical protein |
| RJ610_09085 | WND82482.1 | 0,46 | 6,02E-09 | | | alpha/beta fold hydrolase |
| RJ610_09090 | WND82483.1 | 0,63 | 2,86E-04 | | | bifunctional hydroxymethylpyrimidine kinase/phosphomethylpyrimidine kinase |
| RJ610_09095 | WND82484.1 | 0,60 | 9,51E-08 | | | DUF5625 family protein |
| RJ610_09100 | WND82485.1 | 0,58 | 2,72E-06 | | | PhoH family protein |
| RJ610_09105 | WND82486.1 | 0,78 | 7,42E-02 | | | peroxiredoxin |
| RJ610_09110 | WND82487.1 | 0,86 | 1,66E-01 | | | glycine cleavage system protein R |
| RJ610_09125 | WND82490.1 | 1,03 | 8,59E-01 | | | hypothetical protein |
| RJ610_09130 | WND82491.1 | 2,33 | 1,87E-10 | | | ferredoxin family protein |
| RJ610_09135 | | 1,38 | 2,22E-02 | | | tRNA-Glu |
| RJ610_09140 | WND82492.1 | 0,78 | 1,17E-02 | | | polynucleotide adenylyltransferase PcnB |
| RJ610_09145 | WND82493.1 | 0,71 | 3,65E-02 | | | 2-amino-4-hydroxy-6-hydroxymethyldihydropteridine diphosphokinase |
| RJ610_09150 | WND82494.1 | 0,75 | 1,98E-02 | | | 3-methyl-2-oxobutanoate hydroxymethyltransferase |
| RJ610_09155 | WND82495.1 | 0,80 | 8,65E-02 | | | pantoate--beta-alanine ligase |
| RJ610_09165 | WND82497.1 | 0,84 | 1,08E-01 | | | glucose-6-phosphate isomerase |
| RJ610_09170 | WND83216.1 | 0,49 | 1,54E-08 | | | tRNA epoxyqueuosine(34) reductase QueG |
| RJ610_09175 | WND82498.1 | 0,75 | 1,90E-02 | | | NAD(P)H-hydrate dehydratase |
| RJ610_09180 | WND82499.1 | 0,69 | 8,72E-03 | | | tRNA (adenosine(37)-N6)-threonylcarbamoyltransferase complex ATPase subunit type 1 TsaE |
| RJ610_09185 | WND82500.1 | 1,24 | 1,71E-02 | | | N-acetylmuramoyl-L-alanine amidase |
| RJ610_09190 | WND82501.1 | 1,14 | 3,23E-01 | | | hypothetical protein |
| RJ610_09195 | WND82502.1 | 0,81 | 5,84E-02 | | | DNA mismatch repair endonuclease MutL |
| RJ610_09200 | WND82503.1 | 1,00 | 9,84E-01 | | | DUF1684 domain-containing protein |

| | | | | | | |
|---|---|---|---|---|---|---|
| RJ610_09205 | WND82504.1 | 1,07 | 5,21E-01 | | | TraB/GumN family protein |
| RJ610_09210 | WND82505.1 | 1,31 | 5,13E-02 | | | Rnf electron transport complex subunit RnfB |
| RJ610_09215 | WND82506.1 | 0,71 | 9,21E-04 | | | methionine--tRNA ligase |
| RJ610_09220 | WND82507.1 | 0,90 | 4,20E-01 | | | DUF2147 domain-containing protein |
| RJ610_09225 | WND82508.1 | 0,08 | 1,26E-72 | | | TonB-dependent receptor |
| RJ610_09230 | WND82509.1 | 0,72 | 8,93E-03 | | | iron-sulfur cluster carrier protein ApbC |
| RJ610_09235 | WND82510.1 | 0,75 | 8,52E-03 | | | dCTP deaminase |
| RJ610_09240 | WND82511.1 | 0,32 | 1,31E-17 | | | RDD family protein |
| RJ610_09245 | WND82512.1 | 0,76 | 1,44E-01 | | | hypothetical protein |
| RJ610_09250 | WND82513.1 | 0,47 | 6,04E-06 | | | pyroglutamyl-peptidase I |
| RJ610_09255 | WND82514.1 | 0,52 | 2,28E-05 | | | DUF979 domain-containing protein |
| RJ610_09260 | WND82515.1 | 0,45 | 4,71E-07 | | | DUF969 domain-containing protein |
| RJ610_09265 | WND82516.1 | 0,59 | 3,17E-05 | | | 5-oxoprolinase subunit PxpA |
| RJ610_09270 | WND82517.1 | 0,63 | 2,35E-04 | | | DUF5668 domain-containing protein |
| RJ610_09275 | WND82518.1 | 0,64 | 1,07E-03 | | | DUF885 domain-containing protein |
| RJ610_09280 | WND82519.1 | 1,11 | 4,20E-01 | | | serine/threonine-protein kinase |
| RJ610_09285 | WND82520.1 | 1,17 | 1,57E-01 | | | serine/threonine-protein kinase |
| RJ610_09290 | WND82521.1 | 1,42 | 1,65E-03 | | | ECF-type sigma factor |
| RJ610_09295 | WND82522.1 | 1,31 | 8,10E-02 | | | biotin-dependent carboxyltransferase family protein |
| RJ610_09300 | WND82523.1 | 0,70 | 9,82E-03 | | | 5-oxoprolinase subunit PxpB |
| RJ610_09305 | WND82524.1 | 0,73 | 3,60E-02 | | | HIT family protein |
| RJ610_09310 | WND82525.1 | 0,67 | 1,36E-04 | | | dienelactone hydrolase family protein |
| RJ610_09315 | WND82526.1 | 0,67 | 8,56E-04 | | | 30S ribosomal protein S12 methylthiotransferase RimO |
| RJ610_09320 | WND82527.1 | 0,89 | 5,70E-01 | | | NAD(P)/FAD-dependent oxidoreductase |
| RJ610_09325 | WND82528.1 | 0,84 | 2,61E-01 | | | YaeQ family protein |
| RJ610_09330 | WND82529.1 | 0,78 | 9,83E-02 | | | pseudouridine synthase |
| RJ610_09335 | WND82530.1 | 0,68 | 6,12E-04 | | | gamma-glutamyl-gamma-aminobutyrate hydrolase family protein |
| RJ610_09340 | WND82531.1 | 1,19 | 1,16E-01 | | | DEAD/DEAH box helicase |
| RJ610_09345 | WND82532.1 | 1,28 | 4,90E-01 | | | DeoR/GlpR family DNA-binding transcription regulator |
| RJ610_09350 | WND82533.1 | 1,27 | 4,35E-01 | | | MFS transporter |
| RJ610_09355 | WND82534.1 | 1,59 | 8,40E-02 | | | YaiI/YqxD family protein |
| RJ610_09360 | WND82535.1 | 1,04 | 8,37E-01 | | | glutathione S-transferase |
| RJ610_09365 | WND82536.1 | 1,16 | 4,53E-01 | | | YdiU family protein |
| RJ610_09370 | | 0,90 | 5,94E-01 | | | GNAT family N-acetyltransferase |
| RJ610_09375 | WND82537.1 | 0,83 | 2,20E-01 | | | hypothetical protein |
| RJ610_09380 | WND82538.1 | 1,03 | 8,58E-01 | | | ATP-binding cassette domain-containing protein |
| RJ610_09385 | WND82539.1 | 0,34 | 8,41E-09 | | | SRPBCC domain-containing protein |
| RJ610_09390 | WND82540.1 | 0,33 | 2,24E-08 | | | helix-turn-helix domain-containing protein |
| RJ610_09395 | WND82541.1 | 1,11 | 5,93E-01 | | | hypothetical protein |
| RJ610_09400 | WND82542.1 | 0,64 | 1,02E-03 | | | RNA 2',3'-cyclic phosphodiesterase |
| RJ610_09405 | WND82543.1 | 0,86 | 3,46E-01 | | | YdeI/OmpD-associated family protein |
| RJ610_09410 | WND82544.1 | 0,93 | 6,69E-01 | | | esterase-like activity of phytase family protein |
| RJ610_09415 | WND82545.1 | 0,64 | 3,21E-04 | | | endonuclease/exonuclease/phosphatase family protein |

| | | | | | | |
|---|---|---|---|---|---|---|
| RJ610_09420 | WND82546.1 | 0,61 | 4,61E-03 | | | hypothetical protein |
| RJ610_09430 | WND82548.1 | 0,86 | 3,58E-01 | | | hypothetical protein |
| RJ610_09435 | WND82549.1 | 0,58 | 6,77E-07 | | | glutathione S-transferase family protein |
| RJ610_09440 | WND82550.1 | 0,69 | 3,68E-02 | | | hypothetical protein |
| RJ610_09445 | WND82551.1 | 1,19 | 2,31E-01 | | | oligopeptide:H+ symporter |
| RJ610_09450 | WND82552.1 | 1,32 | 1,39E-01 | | | rhomboid family intramembrane serine protease |
| RJ610_09455 | WND82553.1 | 0,17 | 2,81E-21 | | | hypothetical protein |
| RJ610_09460 | WND82554.1 | 2,67 | 2,80E-08 | | | hypothetical protein |
| RJ610_09465 | WND82555.1 | 2,58 | 4,61E-10 | | | GNAT family N-acetyltransferase |
| RJ610_09470 | WND82556.1 | 1,00 | 9,96E-01 | | | pseudouridine synthase |
| RJ610_09475 | WND82557.1 | 0,41 | 8,52E-17 | | | DUF3298 domain-containing protein |
| RJ610_09480 | WND82558.1 | 2,43 | 1,01E-08 | | | cyclopropane fatty acyl phospholipid synthase |
| RJ610_09485 | WND82559.1 | 0,81 | 2,85E-02 | | | amino acid permease |
| RJ610_09490 | WND82560.1 | 5,02 | 1,04E-35 | | | hypothetical protein |
| RJ610_09495 | WND82561.1 | 3,34 | 1,30E-07 | | | hypothetical protein |
| RJ610_09500 | WND82562.1 | 0,75 | 1,75E-02 | | | glycerophosphodiester phosphodiesterase family protein |
| RJ610_09505 | WND82563.1 | 1,34 | 2,24E-01 | | | NAD-dependent protein deacetylase |
| RJ610_09510 | WND82564.1 | 0,86 | 3,95E-01 | | | hypothetical protein |
| RJ610_09515 | WND82565.1 | 3,61 | 9,22E-11 | | | YadA family autotransporter adhesin |
| RJ610_09520 | WND82566.1 | 29,51 | 3,77E-78 | | | winged helix-turn-helix domain-containing protein |
| RJ610_09530 | WND82568.1 | 0,47 | 9,76E-05 | | | hypothetical protein |
| RJ610_09535 | WND82569.1 | 0,47 | 6,14E-05 | | | amidohydrolase family protein |
| RJ610_09545 | WND82571.1 | 5,81 | 3,67E-28 | | | glutathione peroxidase |
| RJ610_09550 | WND82572.1 | 0,82 | 5,39E-01 | | | acyltransferase family protein |
| RJ610_09555 | WND82573.1 | 0,63 | 2,28E-04 | | | LytTR family DNA-binding domain-containing protein |
| RJ610_09560 | WND82574.1 | 0,80 | 1,39E-01 | | | TlpA disulfide reductase family protein |
| RJ610_09570 | WND82576.1 | 0,59 | 6,94E-06 | | | Tex family protein |
| RJ610_09575 | WND82577.1 | 0,85 | 6,00E-01 | | | hypothetical protein |
| RJ610_09580 | WND82578.1 | 1,35 | 1,09E-02 | | | PQQ-dependent sugar dehydrogenase |
| RJ610_09585 | WND82579.1 | 1,03 | 8,96E-01 | | | hypothetical protein |
| RJ610_09590 | WND82580.1 | 0,76 | 1,06E-02 | | | ABC transporter ATP-binding protein |
| RJ610_09600 | WND82582.1 | 5,94 | 1,98E-14 | | | hypothetical protein |
| RJ610_09605 | WND82583.1 | 3,61 | 5,42E-10 | | | hypothetical protein |
| RJ610_09610 | WND82584.1 | 1,41 | 4,03E-02 | | | fumarate hydratase |
| RJ610_09615 | WND82585.1 | 17,48 | 9,35E-111 | | | patatin-like phospholipase family protein |
| RJ610_09620 | WND82586.1 | 2,33 | 8,65E-19 | | | phospholipase A |
| RJ610_09625 | WND83217.1 | 2,82 | 5,98E-23 | | | DUF456 domain-containing protein |
| RJ610_09630 | WND82587.1 | 1,46 | 2,68E-02 | | | cold-shock protein |
| RJ610_09640 | WND82589.1 | 0,39 | 1,40E-17 | | | glycine zipper 2TM domain-containing protein |
| RJ610_09645 | WND82590.1 | 1,07 | 5,80E-01 | | | hypothetical protein |
| RJ610_09650 | WND82591.1 | 0,97 | 8,40E-01 | | | tRNA threonylcarbamoyladenosine dehydratase |
| RJ610_09655 | WND82592.1 | 0,59 | 7,70E-06 | | | TatD family hydrolase |
| RJ610_09660 | WND82593.1 | 0,53 | 1,59E-06 | | | DUF6116 family protein |

| | | | | | | |
|---|---|---|---|---|---|---|
| RJ610_09665 | WND82594.1 | 2,22 | 2,65E-06 | | | glycine zipper 2TM domain-containing protein |
| RJ610_09670 | WND82595.1 | 0,94 | 6,56E-01 | | | acyl-CoA dehydrogenase family protein |
| RJ610_09675 | WND83218.1 | 1,76 | 1,98E-04 | | | GNAT family N-acetyltransferase |
| RJ610_09680 | WND83219.1 | 1,83 | 2,58E-05 | | | glutathione S-transferase family protein |
| RJ610_09685 | WND83220.1 | 1,48 | 6,10E-03 | | | thiamine pyrophosphate-dependent enzyme |
| RJ610_09690 | WND82596.1 | 1,00 | 9,89E-01 | | | folate-binding protein |
| RJ610_09695 | WND82597.1 | 1,29 | 7,44E-02 | | | DUF1674 domain-containing protein |
| RJ610_09700 | WND82598.1 | 0,90 | 3,59E-01 | | | succinate dehydrogenase, cytochrome b556 subunit |
| RJ610_09705 | WND82599.1 | 0,71 | 1,51E-02 | | | succinate dehydrogenase, hydrophobic membrane anchor protein |
| RJ610_09710 | WND83221.1 | 0,90 | 5,12E-01 | | | succinate dehydrogenase flavoprotein subunit |
| RJ610_09720 | WND82601.1 | 1,03 | 9,27E-01 | | | hypothetical protein |
| RJ610_09725 | WND82602.1 | 1,37 | 2,37E-01 | | | MAPEG family protein |
| RJ610_09730 | WND82603.1 | 0,80 | 3,94E-01 | | | hypothetical protein |
| RJ610_09735 | WND82604.1 | 0,92 | 7,53E-01 | | | succinate dehydrogenase assembly factor 2 |
| RJ610_09740 | WND82605.1 | 0,83 | 3,19E-01 | | | hypothetical protein |
| RJ610_09745 | WND82606.1 | 1,01 | 9,63E-01 | | | lipoprotein-releasing ABC transporter permease subunit |
| RJ610_09750 | WND83222.1 | 1,02 | 9,00E-01 | | | ATP-binding cassette domain-containing protein |
| RJ610_09755 | WND82607.1 | 1,10 | 5,97E-01 | | | DNA internalization-related competence protein ComEC/Rec2 |
| RJ610_09760 | WND82608.1 | 1,04 | 7,29E-01 | | | MotA/TolQ/ExbB proton channel family protein |
| RJ610_09765 | WND82609.1 | 1,23 | 1,45E-01 | | | biopolymer transporter ExbD |
| RJ610_09770 | WND82610.1 | 1,20 | 2,04E-01 | | | lipid A export permease/ATP-binding protein MsbA |
| RJ610_09775 | WND82611.1 | 1,54 | 7,09E-04 | | | tetraacyldisaccharide 4'-kinase |
| RJ610_09780 | WND82612.1 | 1,61 | 1,05E-04 | | | 3-deoxy-manno-octulosonate cytidylyltransferase |
| RJ610_09785 | WND82613.1 | 1,44 | 2,39E-02 | | | low molecular weight protein-tyrosine-phosphatase |
| RJ610_09790 | | 1,26 | 1,40E-01 | | | excinuclease ABC subunit UvrC |
| RJ610_09795 | WND82614.1 | 1,62 | 7,94E-07 | | | CDP-diacylglycerol--glycerol-3-phosphate 3-phosphatidyltransferase |
| RJ610_09800 | | 1,37 | 1,37E-02 | | | tRNA-Gly |
| RJ610_09805 | | 1,07 | 5,80E-01 | | | tRNA-Cys |
| RJ610_09810 | | 1,12 | 5,04E-01 | | | tRNA-Gly |
| RJ610_09815 | | 0,90 | 6,62E-01 | | | tRNA-Cys |
| RJ610_09820 | WND82615.1 | 0,67 | 5,90E-03 | | | DUF5713 family protein |
| RJ610_09825 | | 1,12 | 6,84E-01 | | | tRNA-Leu |
| RJ610_09830 | WND82616.1 | 0,71 | 1,21E-03 | | | tyrosine-type recombinase/integrase |
| RJ610_09835 | WND82617.1 | 1,11 | 3,65E-01 | | | hypothetical protein |
| RJ610_09840 | WND82618.1 | 0,89 | 3,57E-01 | | | hypothetical protein |
| RJ610_09845 | WND82619.1 | 0,87 | 2,81E-01 | | | hypothetical protein |
| RJ610_09850 | WND82620.1 | 0,90 | 3,65E-01 | | | hypothetical protein |
| RJ610_09855 | WND82621.1 | 0,88 | 3,59E-01 | | | hypothetical protein |
| RJ610_09860 | WND82622.1 | 1,03 | 9,12E-01 | | | hypothetical protein |
| RJ610_09865 | WND82623.1 | 0,99 | 9,74E-01 | | | hypothetical protein |
| RJ610_09870 | WND82624.1 | 1,18 | 2,27E-01 | | | HAMP domain-containing sensor histidine kinase |
| RJ610_09875 | WND82625.1 | 1,67 | 2,00E-03 | | | hypothetical protein |

| | | | | | | |
|---|---|---|---|---|---|---|
| RJ610_09880 | WND82626.1 | 1,19 | 3,58E-01 | | | hypothetical protein |
| RJ610_09885 | WND82627.1 | 0,78 | 1,07E-01 | | | ankyrin repeat domain-containing protein |
| RJ610_09890 | WND82628.1 | 1,00 | 9,91E-01 | | | hypothetical protein |
| RJ610_09895 | WND82629.1 | 1,17 | 2,88E-01 | | | hypothetical protein |
| RJ610_09900 | WND82630.1 | 0,69 | 4,98E-02 | | | hypothetical protein |
| RJ610_09905 | | 1,41 | 9,11E-02 | | | MetQ/NlpA family ABC transporter substrate-binding protein |
| RJ610_09910 | WND82631.1 | 1,05 | 7,34E-01 | | | VOC family protein |
| RJ610_09915 | WND82632.1 | 0,95 | 7,78E-01 | | | helix-turn-helix transcriptional regulator |
| RJ610_09920 | WND82633.1 | 1,70 | 2,25E-01 | | | hypothetical protein |
| RJ610_09925 | WND82634.1 | 2,09 | 4,54E-04 | | | hypothetical protein |
| RJ610_09930 | WND82635.1 | 2,49 | 1,59E-05 | | | hypothetical protein |
| RJ610_09935 | WND82636.1 | 6,22 | 2,98E-37 | | | hypothetical protein |
| RJ610_09940 | WND82637.1 | 6,00 | 5,76E-45 | | | hypothetical protein |
| RJ610_09945 | WND82638.1 | 5,77 | 5,42E-14 | | | multidrug efflux SMR transporter |
| RJ610_09950 | WND82639.1 | 37,14 | 3,02E-11 | | | hypothetical protein |
| RJ610_09955 | WND82640.1 | 1,05 | 7,85E-01 | | | cation:proton antiporter |
| RJ610_09960 | WND82641.1 | 0,93 | 6,85E-01 | | | hemerythrin domain-containing protein |
| RJ610_09965 | WND82642.1 | 0,42 | 2,58E-06 | | | pyridoxamine 5'-phosphate oxidase family protein |
| RJ610_09970 | WND83223.1 | 0,34 | 9,62E-10 | | | GlsB/YeaQ/YmgE family stress response membrane protein |
| RJ610_09975 | WND82643.1 | 0,85 | 3,10E-01 | | | GNAT family N-acetyltransferase |
| RJ610_09980 | WND82644.1 | 0,86 | 4,16E-01 | | | helix-turn-helix domain-containing protein |
| RJ610_09985 | WND82645.1 | 0,61 | 2,14E-05 | | | NAD(P)-dependent oxidoreductase |
| RJ610_09990 | WND82646.1 | 0,66 | 7,12E-04 | | | DUF6194 family protein |
| RJ610_09995 | WND82647.1 | 0,75 | 3,34E-02 | | | 2-amino-4-hydroxy-6-hydroxymethyldihydropteridine diphosphokinase |
| RJ610_10000 | WND82648.1 | 0,88 | 4,50E-01 | | | SDR family oxidoreductase |
| RJ610_10005 | WND82649.1 | 1,41 | 8,88E-03 | | | hypothetical protein |
| RJ610_10010 | WND82650.1 | 3,70 | 1,67E-11 | | | macro domain-containing protein |
| RJ610_10015 | WND82651.1 | 9,99 | 3,03E-40 | | | hypothetical protein |
| RJ610_10020 | WND82652.1 | 2,61 | 2,02E-08 | | | diguanylate cyclase |
| RJ610_10025 | WND82653.1 | 2,16 | 4,33E-05 | | | hypothetical protein |
| RJ610_10030 | WND82654.1 | 1,45 | 1,25E-04 | | | nucleotide sugar dehydrogenase |
| RJ610_10035 | WND82655.1 | 0,79 | 5,46E-02 | | | FMN-binding glutamate synthase family protein |
| RJ610_10040 | WND82656.1 | 1,05 | 7,69E-01 | | | arsenate reductase (glutaredoxin) |
| RJ610_10045 | | 0,84 | 5,74E-01 | | | tRNA-Val |
| RJ610_10050 | WND82657.1 | 0,52 | 3,08E-10 | | | hypothetical protein |
| RJ610_10055 | WND82658.1 | 0,68 | 5,99E-03 | | | hypothetical protein |
| RJ610_10060 | WND82659.1 | 1,27 | 1,65E-01 | | | hypothetical protein |
| RJ610_10065 | WND82660.1 | 2,80 | 3,81E-14 | | | hypothetical protein |
| RJ610_10070 | WND82661.1 | 0,91 | 3,54E-01 | | | TetR/AcrR family transcriptional regulator |
| RJ610_10075 | WND82662.1 | 0,79 | 3,66E-02 | | | acyl-CoA-binding protein |
| RJ610_10080 | WND82663.1 | 0,65 | 4,71E-05 | | | SDR family oxidoreductase |
| RJ610_10085 | WND82664.1 | 1,44 | 6,77E-04 | | | restriction endonuclease |

| | | | | | | |
|---|---|---|---|---|---|---|
| RJ610_10090 | WND82665.1 | 1,12 | 3,56E-01 | | | phasin family protein |
| RJ610_10095 | WND82666.1 | 0,41 | 4,48E-19 | | | patatin-like phospholipase family protein |
| RJ610_10100 | WND82667.1 | 0,72 | 1,04E-02 | | | hypothetical protein |
| RJ610_10105 | WND82668.1 | 0,73 | 1,81E-03 | | | polyhydroxyalkanoic acid system family protein |
| RJ610_10110 | WND82669.1 | 0,66 | 1,03E-03 | | | FHA domain-containing protein |
| RJ610_10115 | WND83224.1 | 2,78 | 2,08E-13 | | | protein-methionine-sulfoxide reductase heme-binding subunit MsrQ |
| RJ610_10120 | WND82670.1 | 3,35 | 6,58E-17 | | | protein-methionine-sulfoxide reductase catalytic subunit MsrP |
| RJ610_10130 | WND82672.1 | 0,67 | 5,53E-02 | | | prephenate dehydratase |
| RJ610_10135 | WND82673.1 | 0,60 | 2,34E-02 | | | histidinol-phosphate transaminase |
| RJ610_10145 | WND82675.1 | 1,16 | 4,14E-01 | | | hypothetical protein |
| RJ610_10150 | WND82676.1 | 2,94 | 9,85E-16 | | | energy transducer TonB |
| RJ610_10155 | WND82677.1 | 0,73 | 3,16E-03 | | | energy transducer TonB |
| RJ610_10160 | WND82678.1 | 0,98 | 8,90E-01 | | | serine--tRNA ligase |
| RJ610_10165 | WND82679.1 | 1,59 | 3,31E-03 | | | hypothetical protein |
| RJ610_10170 | WND82680.1 | 1,49 | 5,67E-04 | | | NAD(P)H-dependent oxidoreductase |
| RJ610_10175 | | 1,11 | 4,62E-01 | | | LysR family transcriptional regulator |
| RJ610_10180 | | 1,36 | 2,44E-02 | | | tRNA-Ser |
| RJ610_10185 | WND82681.1 | 3,09 | 1,98E-11 | | | hypothetical protein |
| RJ610_10190 | WND82682.1 | 1,22 | 2,78E-01 | | | hypothetical protein |
| RJ610_10195 | WND82683.1 | 0,86 | 1,68E-01 | | | trans-2-enoyl-CoA reductase family protein |
| RJ610_10200 | WND82684.1 | 0,70 | 7,75E-03 | | | rRNA pseudouridine synthase |
| RJ610_10205 | WND82685.1 | 0,67 | 1,15E-03 | | | PA4780 family RIO1-like protein kinase |
| RJ610_10210 | WND82686.1 | 0,69 | 3,56E-03 | | | RNA ligase (ATP) |
| RJ610_10215 | WND82687.1 | 0,62 | 1,00E-02 | | | LysE family transporter |
| RJ610_10220 | WND82688.1 | 1,86 | 5,43E-02 | | | LysR substrate-binding domain-containing protein |
| RJ610_10225 | WND82689.1 | 101,56 | 2,21E-49 | | | class III lanthipeptide |
| RJ610_10230 | WND82690.1 | 10,99 | 2,03E-28 | | | class III lanthionine synthetase LanKC |
| RJ610_10235 | WND82691.1 | 3,31 | 1,51E-04 | | | ABC transporter ATP-binding protein |
| RJ610_10240 | WND82692.1 | 3,27 | 1,30E-07 | | | HlyD family efflux transporter periplasmic adaptor subunit |
| RJ610_10245 | WND82693.1 | 2,19 | 5,36E-04 | | | ABC transporter ATP-binding protein |
| RJ610_10250 | WND83225.1 | 2,78 | 4,04E-10 | | | ADOP family duplicated permease |
| RJ610_10255 | WND82694.1 | 0,78 | 2,62E-01 | | | AraC family transcriptional regulator |
| RJ610_10260 | WND82695.1 | 1,85 | 2,78E-03 | | | SDR family oxidoreductase |
| RJ610_10265 | WND82696.1 | 2,32 | 1,18E-17 | | | TIGR04222 domain-containing membrane protein |
| RJ610_10275 | WND82698.1 | 10,15 | 2,00E-14 | | | hypothetical protein |
| RJ610_10280 | WND82699.1 | 2,74 | 6,47E-25 | | | response regulator |
| RJ610_10285 | WND82700.1 | 0,83 | 7,92E-02 | | | Rne/Rng family ribonuclease |
| RJ610_10290 | WND82701.1 | 0,95 | 6,58E-01 | | | RluA family pseudouridine synthase |
| RJ610_10295 | WND82702.1 | 0,87 | 2,09E-01 | | | energy transducer TonB |
| RJ610_10300 | WND82703.1 | 0,85 | 9,35E-02 | | | energy transducer TonB |
| RJ610_10305 | WND82704.1 | 0,89 | 3,22E-01 | | | 4a-hydroxytetrahydrobiopterin dehydratase |
| RJ610_10310 | WND82705.1 | 0,85 | 2,62E-01 | | | NfuA family Fe-S biogenesis protein |

| | | | | | | |
|---|---|---|---|---|---|---|
| RJ610_10315 | WND82706.1 | 0,85 | 2,52E-01 | | | hypothetical protein |
| RJ610_10320 | WND82707.1 | 0,85 | 1,91E-01 | | | c-type cytochrome |
| RJ610_10325 | WND83226.1 | 0,95 | 7,21E-01 | | | cytochrome c |
| RJ610_10330 | WND83227.1 | 1,04 | 8,52E-01 | | | efflux RND transporter periplasmic adaptor subunit |
| RJ610_10335 | | 0,71 | 6,05E-02 | | | efflux RND transporter permease subunit |
| RJ610_10340 | WND82708.1 | 0,72 | 7,23E-02 | | | efflux RND transporter permease subunit |
| RJ610_10345 | WND82709.1 | 0,38 | 3,04E-11 | | | hypothetical protein |
| RJ610_10350 | WND82710.1 | 0,11 | 1,53E-63 | | | hypothetical protein |
| RJ610_10355 | WND82711.1 | 0,12 | 7,60E-39 | | | hypothetical protein |
| RJ610_10360 | WND82712.1 | 0,75 | 3,50E-02 | | | hypothetical protein |
| RJ610_10365 | WND82713.1 | 0,81 | 6,38E-02 | | | TraB/GumN family protein |
| RJ610_10370 | WND82714.1 | 1,14 | 5,10E-01 | | | alpha/beta fold hydrolase |
| RJ610_10375 | WND82715.1 | 0,69 | 1,76E-03 | | | TetR/AcrR family transcriptional regulator |
| RJ610_10380 | WND82716.1 | 0,65 | 1,27E-04 | | | TetR family transcriptional regulator C-terminal domain-containing protein |
| RJ610_10385 | WND82717.1 | 1,15 | 4,50E-01 | | | NADP-dependent oxidoreductase |
| RJ610_10390 | WND82718.1 | 0,85 | 2,93E-01 | | | N-acetyltransferase family protein |
| RJ610_10395 | WND82719.1 | 0,69 | 2,43E-04 | | | carbon-nitrogen hydrolase |
| RJ610_10400 | WND82720.1 | 1,01 | 9,71E-01 | | | hypothetical protein |
| RJ610_10405 | WND82721.1 | 2,23 | 3,46E-17 | | | agmatine deiminase family protein |
| RJ610_10410 | WND82722.1 | 3,79 | 2,20E-45 | | | hypothetical protein |
| RJ610_10415 | WND82723.1 | 1,12 | 5,09E-01 | | | type B 50S ribosomal protein L36 |
| RJ610_10420 | WND83228.1 | 1,01 | 9,53E-01 | | | (d)CMP kinase |
| RJ610_10425 | WND82724.1 | 0,99 | 9,13E-01 | | | 30S ribosomal protein S1 |
| RJ610_10430 | WND82725.1 | 1,18 | 3,03E-01 | | | integration host factor subunit beta |
| RJ610_10435 | WND82726.1 | 0,84 | 1,70E-01 | | | lipopolysaccharide assembly protein LapA domain-containing protein |
| RJ610_10440 | WND82727.1 | 0,81 | 7,32E-02 | | | lipopolysaccharide assembly protein LapB |
| RJ610_10445 | WND82728.1 | 0,77 | 2,82E-02 | | | lipopolysaccharide biosynthesis protein |
| RJ610_10450 | WND82729.1 | 1,75 | 4,32E-08 | | | nucleoside-diphosphate sugar epimerase/dehydratase |
| RJ610_10455 | WND82730.1 | 1,56 | 1,59E-06 | | | UTP--glucose-1-phosphate uridylyltransferase GalU |
| RJ610_10460 | WND82731.1 | 1,19 | 1,30E-01 | | | hypothetical protein |
| RJ610_10465 | WND82732.1 | 0,77 | 3,98E-02 | | | FAD-binding oxidoreductase |
| RJ610_10470 | WND82733.1 | 1,02 | 8,70E-01 | | | tRNA-binding protein |
| RJ610_10475 | WND82734.1 | 1,29 | 8,01E-02 | | | hypothetical protein |
| RJ610_10480 | WND82735.1 | 1,77 | 1,54E-05 | | | DUF1295 domain-containing protein |
| RJ610_10485 | WND82736.1 | 0,97 | 8,93E-01 | | | acetyl-CoA C-acyltransferase |
| RJ610_10490 | WND82737.1 | 0,93 | 7,09E-01 | | | 3-hydroxyacyl-CoA dehydrogenase/enoyl-CoA hydratase family protein |
| RJ610_10495 | WND82738.1 | 1,41 | 2,03E-01 | | | TetR family transcriptional regulator |
| RJ610_10500 | WND82739.1 | 1,18 | 1,22E-01 | | | nucleoside-diphosphate kinase |
| RJ610_10505 | WND83229.1 | 1,39 | 5,90E-04 | | | 23S rRNA (adenine(2503)-C(2))-methyltransferase RlmN |
| RJ610_10510 | WND82740.1 | 1,49 | 8,96E-05 | | | type IV pilus biogenesis/stability protein PilW |

| | | | | | | |
|---|---|---|---|---|---|---|
| RJ610_10515 | WND82741.1 | 1,72 | 9,90E-09 | | | DUF4115 domain-containing protein |
| RJ610_10520 | WND82742.1 | 0,86 | 2,21E-01 | | | tetratricopeptide repeat protein |
| RJ610_10525 | WND82743.1 | 0,87 | 3,00E-01 | | | outer membrane protein assembly factor BamB |
| RJ610_10530 | WND82744.1 | 0,73 | 6,46E-04 | | | ribosome biogenesis GTPase Der |
| RJ610_10535 | WND82745.1 | 0,50 | 2,76E-05 | | | molybdenum cofactor guanylyltransferase |
| RJ610_10540 | WND82746.1 | 0,61 | 1,44E-05 | | | molybdopterin molybdotransferase MoeA |
| RJ610_10545 | WND82747.1 | 0,86 | 3,05E-01 | | | molybdopterin-synthase adenylyltransferase MoeB |
| RJ610_10550 | WND82748.1 | 1,00 | 1,00E+00 | | | RidA family protein |
| RJ610_10555 | WND82749.1 | 0,60 | 1,07E-03 | | | alpha/beta fold hydrolase |
| RJ610_10560 | WND82750.1 | 0,74 | 1,25E-01 | | | DUF2272 domain-containing protein |
| RJ610_10565 | WND82751.1 | 0,80 | 1,78E-01 | | | DUF1244 domain-containing protein |
| RJ610_10570 | WND82752.1 | 0,74 | 3,91E-03 | | | bifunctional methylenetetrahydrofolate dehydrogenase/methenyltetrahydrofolate cyclohydrolase FolD |
| RJ610_10575 | WND82753.1 | 0,84 | 1,39E-01 | | | IMP dehydrogenase |
| RJ610_10580 | WND82754.1 | 0,95 | 6,36E-01 | | | glutamine-hydrolyzing GMP synthase |
| RJ610_10585 | WND82755.1 | 1,17 | 1,60E-01 | | | hypothetical protein |
| RJ610_10590 | WND82756.1 | 6,97 | 3,63E-19 | | | hypothetical protein |
| RJ610_10595 | WND82757.1 | 19,52 | 6,51E-55 | | | hypothetical protein |
| RJ610_10600 | WND82758.1 | 1,51 | 1,65E-01 | | | hypothetical protein |
| RJ610_10605 | WND82759.1 | 0,75 | 9,92E-02 | | | TetR/AcrR family transcriptional regulator |
| RJ610_10610 | WND82760.1 | 1,50 | 1,49E-02 | | | DUF1993 domain-containing protein |
| RJ610_10615 | WND82761.1 | 0,74 | 4,14E-02 | | | hypothetical protein |
| RJ610_10620 | WND82762.1 | 0,71 | 6,40E-02 | | | tRNA adenosine(34) deaminase TadA |
| RJ610_10625 | WND82763.1 | 1,60 | 1,72E-02 | | | hypothetical protein |
| RJ610_10630 | WND82764.1 | 1,87 | 1,04E-05 | | | Cache 3/Cache 2 fusion domain-containing protein |
| RJ610_10635 | WND82765.1 | 1,76 | 2,35E-05 | | | DUF2721 domain-containing protein |
| RJ610_10640 | WND82766.1 | 1,28 | 6,89E-02 | | | DUF2721 domain-containing protein |
| RJ610_10645 | WND82767.1 | 1,19 | 1,36E-01 | | | oligoribonuclease |
| RJ610_10650 | WND82768.1 | 1,99 | 3,17E-11 | | | mechanosensitive ion channel |
| RJ610_10655 | WND82769.1 | 1,81 | 3,78E-11 | | | phosphoenolpyruvate synthase |
| RJ610_10660 | WND82770.1 | 0,93 | 5,88E-01 | | | pyruvate, water dikinase regulatory protein |
| RJ610_10665 | WND82771.1 | 0,73 | 2,75E-03 | | | DUF1249 domain-containing protein |
| RJ610_10670 | | 1,14 | 1,77E-01 | | | tRNA-Ala |
| RJ610_10675 | | 1,20 | 8,29E-02 | | | tRNA-Glu |
| RJ610_10680 | WND82772.1 | 0,90 | 5,71E-01 | | | alpha/beta hydrolase-fold protein |
| RJ610_10685 | WND82773.1 | 0,76 | 3,82E-02 | | | hypothetical protein |
| RJ610_10690 | WND82774.1 | 0,57 | 2,49E-03 | | | STM4011 family radical SAM protein |
| RJ610_10695 | WND82775.1 | 0,51 | 5,17E-04 | | | STM4012 family radical SAM protein |
| RJ610_10700 | WND82776.1 | 0,61 | 7,87E-03 | | | STM4013/SEN3800 family hydrolase |
| RJ610_10705 | WND83230.1 | 0,54 | 2,91E-03 | | | STM4014 family protein |
| RJ610_10715 | WND82778.1 | 1,06 | 8,58E-01 | | | GntP family permease |
| RJ610_10720 | WND82779.1 | 1,85 | 2,19E-02 | | | TonB-dependent receptor |
| RJ610_10725 | WND82780.1 | 0,73 | 3,26E-01 | | | hypothetical protein |

| | | | | | | |
|---|---|---|---|---|---|---|
| RJ610_10730 | WND82781.1 | 0,84 | 2,37E-01 | | | PAS-domain containing protein |
| RJ610_10735 | WND82782.1 | 0,84 | 3,59E-01 | | | response regulator transcription factor |
| RJ610_10740 | WND82783.1 | 1,18 | 4,86E-01 | | | 3-hydroxybutyrate oligomer hydrolase family protein |
| RJ610_10745 | WND82784.1 | 0,74 | 1,46E-03 | | | CDP-diacylglycerol--serine O-phosphatidyltransferase |
| RJ610_10750 | WND82785.1 | 0,72 | 1,37E-02 | | | class III poly(R)-hydroxyalkanoic acid synthase subunit PhaE |
| RJ610_10755 | WND82786.1 | 0,81 | 8,97E-02 | | | class III poly(R)-hydroxyalkanoic acid synthase subunit PhaC |
| RJ610_10765 | WND82788.1 | 0,61 | 7,75E-03 | | | DUF5329 domain-containing protein |
| RJ610_10770 | WND82789.1 | 1,06 | 7,09E-01 | | | PspC domain-containing protein |
| RJ610_10775 | WND82790.1 | 1,26 | 1,53E-01 | | | hypothetical protein |
| RJ610_10780 | WND82791.1 | 0,72 | 2,95E-02 | | | NRDE family protein |
| RJ610_10785 | WND82792.1 | 0,45 | 3,90E-07 | | | hypothetical protein |
| RJ610_10790 | WND82793.1 | 0,44 | 5,30E-06 | | | hypothetical protein |
| RJ610_10795 | | 0,44 | 1,06E-04 | | | tRNA-Leu |
| RJ610_10800 | WND82794.1 | 1,28 | 6,33E-01 | | | hypothetical protein |
| RJ610_10805 | WND82795.1 | 1,05 | 7,82E-01 | | | SAM-dependent methyltransferase |
| RJ610_10810 | WND82796.1 | 0,53 | 5,70E-06 | | | hypothetical protein |
| RJ610_10815 | WND82797.1 | 0,58 | 5,28E-05 | | | hypothetical protein |
| RJ610_10820 | WND82798.1 | 0,67 | 6,79E-02 | | | DUF2214 family protein |
| RJ610_10825 | WND82799.1 | 0,73 | 2,66E-03 | | | cysteine dioxygenase family protein |
| RJ610_10830 | WND82800.1 | 1,71 | 9,14E-06 | | | homoserine O-acetyltransferase |
| RJ610_10835 | WND83231.1 | 1,06 | 6,98E-01 | | | cytochrome C biogenesis protein |
| RJ610_10840 | WND82801.1 | 1,32 | 7,30E-02 | | | cytochrome c-type biogenesis protein |
| RJ610_10845 | WND82802.1 | 1,13 | 4,54E-01 | | | DsbE family thiol:disulfide interchange protein |
| RJ610_10850 | | 1,05 | 7,71E-01 | | | heme lyase CcmF/NrfE family subunit |
| RJ610_10855 | WND82803.1 | 0,95 | 7,53E-01 | | | cytochrome c maturation protein CcmE |
| RJ610_10860 | WND82804.1 | 0,84 | 3,43E-01 | | | heme exporter protein CcmD |
| RJ610_10865 | WND82805.1 | 0,99 | 9,25E-01 | | | heme ABC transporter permease CcmC |
| RJ610_10870 | WND82806.1 | 1,12 | 4,22E-01 | | | heme exporter protein CcmB |
| RJ610_10875 | WND82807.1 | 0,76 | 1,13E-01 | | | heme ABC exporter ATP-binding protein CcmA |
| RJ610_10880 | WND82808.1 | 0,66 | 2,66E-03 | | | DUF3293 domain-containing protein |
| RJ610_10885 | WND82809.1 | 1,87 | 2,78E-12 | | | pyridoxal phosphate-dependent aminotransferase |
| RJ610_10890 | | 1,17 | 3,34E-01 | | | hypothetical protein |
| RJ610_10895 | WND82810.1 | 0,60 | 4,91E-04 | | | type IV secretion system protein |
| RJ610_10905 | WND82812.1 | 0,36 | 6,97E-14 | | | TrbI/VirB10 family protein |
| RJ610_10910 | WND82813.1 | 0,63 | 3,26E-04 | | | P-type DNA transfer ATPase VirB11 |
| RJ610_10915 | WND83232.1 | 1,21 | 1,52E-01 | | | lytic transglycosylase domain-containing protein |
| RJ610_10920 | WND82814.1 | 0,53 | 2,53E-11 | | | TrbC/VirB2 family protein |
| RJ610_10925 | WND82815.1 | 0,48 | 1,36E-13 | | | VirB3 family type IV secretion system protein |
| RJ610_10930 | WND82816.1 | 0,90 | 4,15E-01 | | | VirB4 family type IV secretion/conjugal transfer ATPase |
| RJ610_10935 | WND82817.1 | 0,60 | 5,81E-06 | | | hypothetical protein |
| RJ610_10940 | WND82818.1 | 0,54 | 2,17E-08 | | | hypothetical protein |
| RJ610_10945 | WND82819.1 | 0,42 | 2,46E-13 | | | type IV secretion system protein |
| RJ610_10950 | WND82820.1 | 0,71 | 6,26E-03 | | | DUF4189 domain-containing protein |

| | | | | | | |
|---|---|---|---|---|---|---|
| RJ610_10955 | WND82821.1 | 0,68 | 2,28E-03 | | | DUF4189 domain-containing protein |
| RJ610_10960 | WND82822.1 | 0,72 | 2,47E-02 | | | DUF4189 domain-containing protein |
| RJ610_10965 | WND82823.1 | 0,72 | 2,79E-03 | | | DUF4189 domain-containing protein |
| RJ610_10970 | WND82824.1 | 0,72 | 4,69E-03 | | | DUF4189 domain-containing protein |
| RJ610_10975 | WND82825.1 | 0,50 | 9,72E-04 | | | error-prone DNA polymerase |
| RJ610_10980 | WND82826.1 | 0,48 | 4,51E-04 | | | DNA polymerase Y family protein |
| RJ610_10985 | WND82827.1 | 0,52 | 8,95E-04 | | | translesion DNA synthesis-associated protein ImuA |
| RJ610_10990 | WND82828.1 | 0,64 | 8,02E-03 | | | transcriptional repressor LexA |
| RJ610_10995 | WND82829.1 | 1,02 | 8,85E-01 | | | GbsR/MarR family transcriptional regulator |
| RJ610_11000 | WND82830.1 | 1,76 | 1,05E-01 | | | DUF393 domain-containing protein |
| RJ610_11005 | WND82831.1 | 2,92 | 5,05E-06 | | | TIGR01777 family oxidoreductase |
| RJ610_11010 | WND82832.1 | 2,54 | 8,12E-04 | | | DUF4166 domain-containing protein |
| RJ610_11015 | WND82833.1 | 1,08 | 6,64E-01 | | | RES family NAD+ phosphorylase |
| RJ610_11020 | WND82834.1 | 0,68 | 1,22E-02 | | | DUF2384 domain-containing protein |
| RJ610_11025 | WND82835.1 | 0,51 | 1,98E-08 | | | hypothetical protein |
| RJ610_11030 | WND82836.1 | 0,50 | 1,62E-02 | | | hypothetical protein |
| RJ610_11040 | WND82837.1 | 1,37 | 7,23E-02 | | | acyl-CoA dehydrogenase family protein |
| RJ610_11045 | WND82838.1 | 1,43 | 2,05E-02 | | | enoyl-CoA hydratase |
| RJ610_11050 | WND82839.1 | 1,33 | 3,85E-02 | | | enoyl-CoA hydratase/isomerase family protein |
| RJ610_11055 | WND82840.1 | 1,39 | 1,36E-02 | | | 3-hydroxyisobutyrate dehydrogenase |
| RJ610_11060 | WND82841.1 | 1,33 | 8,74E-03 | | | epoxyqueuosine reductase QueH |
| RJ610_11065 | WND82842.1 | 0,16 | 9,25E-52 | | | cytochrome c peroxidase |
| RJ610_11070 | WND82843.1 | 0,07 | 6,59E-69 | | | hypothetical protein |
| RJ610_11075 | WND82844.1 | 2,31 | 9,10E-10 | | | hypothetical protein |
| RJ610_11080 | WND82845.1 | 0,98 | 8,86E-01 | | | S8 family peptidase |
| RJ610_11090 | WND82847.1 | 1,08 | 5,29E-01 | | | hypothetical protein |
| RJ610_11095 | | 1,22 | 4,13E-02 | | | transfer-messenger RNA |
| RJ610_11100 | WND82848.1 | 1,15 | 2,65E-01 | | | DNA-binding protein |
| RJ610_11105 | WND82849.1 | 1,84 | 2,13E-06 | | | hypothetical protein |
| RJ610_11110 | WND82850.1 | 1,46 | 1,79E-01 | | | hypothetical protein |
| RJ610_11115 | WND82851.1 | 0,65 | 1,22E-03 | | | CHASE domain-containing protein |
| RJ610_11120 | WND82852.1 | 0,85 | 2,00E-01 | | | SsrA-binding protein SmpB |
| RJ610_11125 | WND82853.1 | 0,86 | 4,67E-01 | | | type II toxin-antitoxin system RatA family toxin |
| RJ610_11130 | WND82854.1 | 0,82 | 3,53E-01 | | | RnfH family protein |
| RJ610_11135 | WND82855.1 | 0,80 | 6,27E-02 | | | outer membrane protein assembly factor BamE |
| RJ610_11140 | WND82856.1 | 1,59 | 1,75E-05 | | | ferric iron uptake transcriptional regulator |
| RJ610_11145 | WND82857.1 | 0,48 | 2,06E-06 | | | DNA repair protein RecN |
| RJ610_11150 | WND82858.1 | 1,04 | 8,62E-01 | | | heat-inducible transcriptional repressor HrcA |
| RJ610_11155 | WND82859.1 | 0,98 | 9,43E-01 | | | nucleotide exchange factor GrpE |
| RJ610_11165 | WND82861.1 | 1,25 | 4,51E-01 | | | molecular chaperone DnaJ |
| RJ610_11170 | WND82862.1 | 0,31 | 4,75E-22 | | | DUF3857 domain-containing protein |
| RJ610_11175 | WND82863.1 | 0,30 | 1,41E-18 | | | hypothetical protein |
| RJ610_11180 | WND82864.1 | 0,20 | 7,94E-11 | | | DUF3828 domain-containing protein |

| | | | | | | |
|---|---|---|---|---|---|---|
| RJ610_11185 | WND82865.1 | 0,85 | 1,43E-01 | | | 4-hydroxy-tetrahydrodipicolinate reductase |
| RJ610_11190 | WND82866.1 | 0,93 | 5,67E-01 | | | glutamine-hydrolyzing carbamoyl-phosphate synthase small subunit |
| RJ610_11195 | WND82867.1 | 1,09 | 5,75E-01 | | | hypothetical protein |
| RJ610_11200 | WND82868.1 | 1,47 | 1,50E-03 | | | hypothetical protein |
| RJ610_11205 | WND82869.1 | 1,23 | 5,48E-02 | | | carbamoyl-phosphate synthase large subunit |
| RJ610_11210 | WND82870.1 | 0,91 | 5,21E-01 | | | transcription elongation factor GreA |
| RJ610_11215 | WND82871.1 | 0,85 | 2,59E-01 | | | phosphoglycerate mutase |
| RJ610_11220 | WND82872.1 | 0,98 | 9,17E-01 | | | single-stranded-DNA-specific exonuclease RecJ |
| RJ610_11225 | WND82873.1 | 12,80 | 1,39E-23 | | | hypothetical protein |
| RJ610_11230 | WND82874.1 | 1,61 | 1,23E-04 | | | N(4)-(beta-N-acetylglucosaminyl)-L-asparaginase |
| RJ610_11235 | WND83234.1 | 1,37 | 1,35E-01 | | | copper homeostasis protein CutC |
| RJ610_11240 | WND82875.1 | 1,35 | 2,13E-01 | | | hypothetical protein |
| RJ610_11245 | WND82876.1 | 3,17 | 1,61E-20 | | | TonB-dependent receptor |
| RJ610_11255 | WND82877.1 | 3,27 | 8,03E-30 | | | family 20 glycosylhydrolase |
| RJ610_11265 | WND82879.1 | 1,59 | 3,06E-03 | | | glycoside hydrolase family 2 protein |
| RJ610_11270 | WND82880.1 | 0,59 | 1,75E-04 | | | VIT domain-containing protein |
| RJ610_11275 | WND82881.1 | 0,85 | 1,07E-01 | | | peptide chain release factor 2 |
| RJ610_11280 | WND82882.1 | 0,85 | 1,27E-01 | | | lysine--tRNA ligase |
| RJ610_11285 | WND83236.1 | 1,03 | 8,37E-01 | | | two-component system response regulator |
| RJ610_11290 | WND82883.1 | 0,76 | 2,03E-02 | | | long-chain fatty acid--CoA ligase |
| RJ610_11295 | WND82884.1 | 0,55 | 1,80E-04 | | | nuclear transport factor 2 family protein |
| RJ610_11300 | WND82885.1 | 0,85 | 2,18E-01 | | | aconitate hydratase AcnA |
| RJ610_11305 | WND82886.1 | 1,00 | 9,98E-01 | | | LysR family transcriptional regulator |
| RJ610_11310 | WND82887.1 | 0,74 | 2,34E-01 | | | 4-oxalomesaconate tautomerase |
| RJ610_11315 | WND82888.1 | 0,48 | 6,35E-03 | | | ABC transporter substrate-binding protein |
| RJ610_11320 | WND82889.1 | 0,58 | 4,37E-02 | | | ABC transporter ATP-binding protein |
| RJ610_11325 | WND82890.1 | 0,74 | 1,29E-01 | | | ABC transporter permease |
| RJ610_11330 | WND82891.1 | 0,68 | 3,08E-01 | | | porin |
| RJ610_11335 | WND82892.1 | 1,01 | 9,48E-01 | | | AbrB/MazE/SpoVT family DNA-binding domain-containing protein |
| RJ610_11340 | WND82893.1 | 0,92 | 5,82E-01 | | | type II toxin-antitoxin system VapC family toxin |
| RJ610_11345 | WND82894.1 | 1,19 | 1,22E-01 | | | bifunctional aconitate hydratase 2/2-methylisocitrate dehydratase |
| RJ610_11350 | WND82895.1 | 0,93 | 7,22E-01 | | | hypothetical protein |
| RJ610_11355 | WND82896.1 | 0,99 | 9,25E-01 | | | high frequency lysogenization protein HflD |
| RJ610_11360 | WND82897.1 | 0,90 | 4,62E-01 | | | tRNA 2-thiouridine(34) synthase MnmA |
| RJ610_11365 | WND82898.1 | 1,01 | 9,45E-01 | | | NUDIX hydrolase |
| RJ610_11370 | WND82899.1 | 1,18 | 3,60E-01 | | | YbjN domain-containing protein |
| RJ610_11375 | WND82900.1 | 0,97 | 8,11E-01 | | | ATP-dependent Clp protease adapter ClpS |
| RJ610_11380 | WND82901.1 | 0,98 | 9,22E-01 | | | ATP-dependent Clp protease ATP-binding subunit ClpA |
| RJ610_11385 | WND82902.1 | 0,32 | 3,24E-14 | | | hypothetical protein |
| RJ610_11390 | WND82903.1 | 0,38 | 2,39E-12 | | | hypothetical protein |
| RJ610_11400 | WND82905.1 | 3,80 | 4,28E-06 | | | hypothetical protein |
| RJ610_11405 | WND82906.1 | 4,92 | 3,46E-20 | | | hypothetical protein |

| | | | | | | |
|---|---|---|---|---|---|---|
| RJ610_11410 | WND82907.1 | 3,10 | 3,46E-13 | | | hypothetical protein |
| RJ610_11415 | WND82908.1 | 1,58 | 6,26E-03 | | | hypothetical protein |
| RJ610_11420 | WND82909.1 | 1,09 | 5,25E-01 | | | translation initiation factor IF-1 |
| RJ610_11425 | WND82910.1 | 0,79 | 2,07E-01 | | | leucyl/phenylalanyl-tRNA--protein transferase |
| RJ610_11430 | WND83237.1 | 1,30 | 1,36E-01 | | | GNAT family N-acetyltransferase |
| RJ610_11435 | WND82911.1 | 1,25 | 2,19E-01 | | | GNAT family N-acetyltransferase |
| RJ610_11445 | WND82913.1 | 0,72 | 5,89E-03 | | | alanine dehydrogenase |
| RJ610_11450 | WND83238.1 | 0,96 | 7,75E-01 | | | DNA translocase FtsK 4TM domain-containing protein |
| RJ610_11455 | WND82914.1 | 1,04 | 7,57E-01 | | | outer membrane lipoprotein chaperone LolA |
| RJ610_11460 | WND82915.1 | 0,96 | 8,71E-01 | | | GNAT family N-acetyltransferase |
| RJ610_11465 | WND82916.1 | 1,10 | 7,26E-01 | | | hypothetical protein |
| RJ610_11475 | WND82918.1 | 0,56 | 1,08E-04 | | | hypothetical protein |
| RJ610_11480 | WND82919.1 | 0,43 | 3,87E-07 | | | hypothetical protein |
| RJ610_11485 | WND82920.1 | 0,88 | 5,03E-01 | | | hypothetical protein |
| RJ610_11490 | WND82921.1 | 0,59 | 9,61E-03 | | | LysR family transcriptional regulator |
| RJ610_11495 | WND82922.1 | 1,42 | 1,71E-01 | | | alpha/beta fold hydrolase |
| RJ610_11500 | | 1,52 | 5,34E-02 | | | glycosyl hydrolase 108 family protein |
| RJ610_11505 | WND82923.1 | 1,14 | 3,29E-01 | | | replication-associated recombination protein A |
| RJ610_11510 | WND82924.1 | 0,31 | 1,12E-16 | | | CrcB family protein |
| RJ610_11515 | WND82925.1 | 0,26 | 7,25E-31 | | | hypothetical protein |
| RJ610_11520 | WND82926.1 | 2,52 | 4,11E-13 | | | SapC family protein |
| RJ610_11525 | WND82927.1 | 20,48 | 1,86E-42 | | | hypothetical protein |
| RJ610_11530 | WND82928.1 | 11,89 | 1,65E-11 | | | ShlB/FhaC/HecB family hemolysin secretion/activation protein |
| RJ610_11535 | WND82929.1 | 1,79 | 3,95E-02 | | | DUF2341 domain-containing protein |
| RJ610_11540 | WND82930.1 | 2,17 | 2,82E-02 | | | biopolymer transporter ExbD |
| RJ610_11545 | WND82931.1 | 1,78 | 1,00E-01 | | | TonB family protein |
| RJ610_11550 | WND82932.1 | 0,99 | 9,82E-01 | | | putative porin |
| RJ610_11555 | WND82933.1 | 0,98 | 9,82E-01 | | | hypothetical protein |
| RJ610_11560 | WND82934.1 | 1,16 | 8,66E-01 | | | hypothetical protein |
| RJ610_11565 | WND82935.1 | 1,94 | 4,28E-01 | | | hypothetical protein |
| RJ610_11570 | WND82936.1 | 1,36 | 3,18E-02 | | | filamentous hemagglutinin family protein |
| RJ610_11580 | WND82938.1 | 2,44 | 3,23E-02 | | | hypothetical protein |
| RJ610_11585 | WND82939.1 | 1,33 | 5,84E-02 | | | ABC transporter permease subunit |
| RJ610_11590 | WND83239.1 | 1,48 | 1,77E-03 | | | ABC transporter permease subunit |
| RJ610_11595 | WND82940.1 | 1,24 | 7,11E-02 | | | ABC transporter ATP-binding protein |
| RJ610_11600 | WND83240.1 | 1,20 | 6,76E-02 | | | polyamine ABC transporter substrate-binding protein |
| RJ610_11605 | WND82941.1 | 1,55 | 1,35E-03 | | | TorF family putative porin |
| RJ610_11610 | WND82942.1 | 1,53 | 4,79E-02 | | | LysR family transcriptional regulator |
| RJ610_11615 | WND82943.1 | 1,88 | 3,53E-06 | | | acyl-CoA dehydrogenase family protein |
| RJ610_11620 | WND82944.1 | 1,63 | 2,45E-03 | | | SDR family oxidoreductase |
| RJ610_11625 | WND82945.1 | 1,26 | 1,21E-01 | | | phosphotransferase family protein |
| RJ610_11630 | WND82946.1 | 1,14 | 4,49E-01 | | | histidine phosphatase family protein |
| RJ610_11635 | WND82947.1 | 1,38 | 5,35E-02 | | | SDR family oxidoreductase |

| | | | | | | |
|---|---|---|---|---|---|---|
| RJ610_11640 | WND82948.1 | 0,94 | 7,40E-01 | | | Cu(I)-responsive transcriptional regulator |
| RJ610_11645 | WND82949.1 | 0,68 | 1,92E-03 | | | heavy metal translocating P-type ATPase |
| RJ610_11650 | WND82950.1 | 0,57 | 2,49E-03 | | | DEAD/DEAH box helicase |
| RJ610_11655 | WND82951.1 | 0,71 | 9,33E-02 | | | hypothetical protein |
| RJ610_11660 | WND82952.1 | 0,99 | 9,71E-01 | | | low affinity iron permease family protein |
| RJ610_11665 | WND82953.1 | 1,30 | 1,63E-01 | | | Ku protein |
| RJ610_11670 | WND82954.1 | 1,73 | 5,57E-05 | | | DNA ligase D |
| RJ610_11675 | WND82955.1 | 1,74 | 6,91E-02 | | | MgtC/SapB family protein |
| RJ610_11680 | | 2,20 | 6,69E-03 | | | HupB |
| RJ610_11685 | WND82956.1 | 2,39 | 4,71E-06 | | | BON domain-containing protein |
| RJ610_11690 | WND82957.1 | 1,68 | 1,04E-02 | | | DUF3247 family protein |
| RJ610_11695 | WND82958.1 | 1,09 | 6,89E-01 | | | GIY-YIG nuclease family protein |
| RJ610_11700 | WND82959.1 | 1,62 | 2,38E-03 | | | hypothetical protein |
| RJ610_11705 | WND82960.1 | 1,85 | 1,38E-03 | | | alpha-ketoglutarate-dependent dioxygenase AlkB |
| RJ610_11710 | WND83241.1 | 2,83 | 2,19E-10 | | | MFS transporter |
| RJ610_11715 | WND82961.1 | 0,92 | 6,44E-01 | | | helix-turn-helix transcriptional regulator |
| RJ610_11720 | WND82962.1 | 0,91 | 6,78E-01 | | | alpha/beta hydrolase |
| RJ610_11725 | WND82963.1 | 1,53 | 5,64E-02 | | | isoamylase |
| RJ610_11730 | WND82964.1 | 1,36 | 5,67E-01 | | | class I SAM-dependent methyltransferase |
| RJ610_11735 | WND82965.1 | 1,12 | 7,36E-01 | | | glycosyltransferase |
| RJ610_11740 | WND82966.1 | 1,13 | 5,22E-01 | | | hypothetical protein |
| RJ610_11745 | WND82967.1 | 1,32 | 3,17E-01 | | | ATP-binding protein |
| RJ610_11750 | WND82968.1 | 0,90 | 7,92E-01 | | | hypothetical protein |
| RJ610_11755 | WND82969.1 | 1,55 | 5,20E-03 | | | vWA domain-containing protein |
| RJ610_11760 | WND82970.1 | 1,06 | 8,99E-01 | | | hypothetical protein |
| RJ610_11765 | WND82971.1 | 1,25 | 5,42E-01 | | | hypothetical protein |
| RJ610_11770 | WND82972.1 | 1,09 | 7,70E-01 | | | hypothetical protein |
| RJ610_11775 | WND82973.1 | 1,65 | 1,14E-01 | | | phage tail protein |
| RJ610_11780 | WND82974.1 | 1,34 | 5,06E-01 | | | baseplate J/gp47 family protein |
| RJ610_11785 | WND82975.1 | 1,86 | 3,74E-01 | | | GPW/gp25 family protein |
| RJ610_11790 | WND82976.1 | 1,25 | 5,21E-01 | | | hypothetical protein |
| RJ610_11795 | WND82977.1 | 3,58 | 9,61E-05 | | | phage baseplate assembly protein V |
| RJ610_11800 | WND82978.1 | 1,17 | 7,50E-01 | | | contractile injection system protein, VgrG/Pvc8 family |
| RJ610_11805 | WND82979.1 | 2,33 | 3,70E-01 | | | hypothetical protein |
| RJ610_11810 | WND82980.1 | 1,92 | 8,74E-02 | | | LysM peptidoglycan-binding domain-containing protein |
| RJ610_11815 | WND82981.1 | 1,02 | 9,90E-01 | | | phage tail protein |
| RJ610_11820 | WND82982.1 | 0,96 | 9,17E-01 | | | hypothetical protein |
| RJ610_11825 | WND82983.1 | 1,77 | 2,68E-01 | | | phage tail protein |
| RJ610_11830 | WND82984.1 | 1,72 | 3,78E-02 | | | phage tail sheath subtilisin-like domain-containing protein |
| RJ610_11835 | WND82985.1 | 3,66 | 6,36E-02 | | | hypothetical protein |
| RJ610_11840 | WND82986.1 | 0,88 | 8,81E-01 | | | DUF4255 domain-containing protein |
| RJ610_11845 | WND82987.1 | 3,61 | 2,99E-04 | | | hypothetical protein |
| RJ610_11850 | WND82988.1 | 2,10 | 3,12E-02 | | | sigma-54 dependent transcriptional regulator |

| | | | | | | |
|---|---|---|---|---|---|---|
| RJ610_11855 | WND82989.1 | 2,17 | 1,07E-08 | | | hypothetical protein |
| RJ610_11860 | WND82990.1 | 1,48 | 3,40E-03 | | | hypothetical protein |
| RJ610_11865 | WND82991.1 | 1,67 | 6,14E-06 | | | peptidoglycan-binding domain-containing protein |
| RJ610_11870 | WND82992.1 | 1,42 | 3,37E-02 | | | hypothetical protein |
| RJ610_11875 | WND82993.1 | 2,22 | 1,62E-04 | | | hypothetical protein |
| RJ610_11880 | WND83242.1 | 1,73 | 9,81E-04 | | | DUF4189 domain-containing protein |
| RJ610_11885 | WND82994.1 | 1,62 | 3,15E-04 | | | peptidoglycan-binding domain-containing protein |
| RJ610_11890 | WND82995.1 | 2,50 | 6,63E-05 | | | hypothetical protein |
| RJ610_11895 | WND82996.1 | 3,00 | 5,18E-07 | | | hypothetical protein |
| RJ610_11900 | WND82997.1 | 7,01 | 1,06E-54 | | | type IV secretion system protein |
| RJ610_11905 | WND82998.1 | 10,13 | 9,01E-67 | | | type IV secretion system protein |
| RJ610_11910 | WND82999.1 | 5,12 | 1,27E-21 | | | hypothetical protein |
| RJ610_11915 | WND83000.1 | 3,46 | 6,98E-10 | | | response regulator transcription factor |
| RJ610_11920 | WND83001.1 | 8,06 | 1,66E-06 | | | response regulator transcription factor |
| RJ610_11925 | WND83002.1 | 8,18 | 9,60E-06 | | | ATP-binding protein |
| RJ610_11930 | WND83003.1 | 12,27 | 1,74E-06 | | | SURF1 family protein |
| RJ610_11935 | WND83004.1 | 8,79 | 2,15E-05 | | | cytochrome o ubiquinol oxidase subunit IV |
| RJ610_11940 | WND83005.1 | 8,36 | 7,57E-05 | | | cytochrome o ubiquinol oxidase subunit III |
| RJ610_11945 | WND83006.1 | 5,01 | 1,83E-03 | | | cytochrome o ubiquinol oxidase subunit I |
| RJ610_11950 | WND83007.1 | 1,51 | 9,69E-02 | | | ubiquinol oxidase subunit II |
| RJ610_11955 | WND83008.1 | 1,35 | 1,44E-01 | | | MFS transporter |
| RJ610_11960 | WND83009.1 | 7,81 | 8,82E-74 | | | MbtH family protein |
| RJ610_11965 | WND83010.1 | 18,70 | 7,73E-109 | | | aminotransferase class V-fold PLP-dependent enzyme |
| RJ610_11970 | WND83011.1 | 214,23 | 5,67E-45 | | | hypothetical protein |
| RJ610_11975 | WND83012.1 | 6,97 | 1,85E-55 | | | hypothetical protein |
| RJ610_11980 | WND83013.1 | 2,57 | 6,23E-12 | | | efflux RND transporter periplasmic adaptor subunit |
| RJ610_11985 | WND83014.1 | 2,31 | 4,23E-09 | | | efflux RND transporter permease subunit |
| RJ610_11990 | WND83015.1 | 3,12 | 6,77E-21 | | | efflux RND transporter permease subunit |
| RJ610_11995 | WND83016.1 | 2,55 | 5,85E-09 | | | efflux transporter outer membrane subunit |
| RJ610_12000 | WND83017.1 | 2,01 | 3,12E-08 | | | hypothetical protein |
| RJ610_12005 | WND83018.1 | 0,87 | 4,18E-01 | | | M23 family metallopeptidase |
| RJ610_12010 | WND83019.1 | 1,18 | 1,52E-01 | | | transporter |
| RJ610_12015 | WND83020.1 | 3,53 | 2,00E-17 | | | benzoate/H(+) symporter BenE family transporter |
| RJ610_12020 | WND83021.1 | 113,97 | 3,32E-79 | | | YdcF family protein |
| RJ610_12025 | WND83022.1 | 205,65 | 1,02E-196 | | | glycosyltransferase family 4 protein |
| RJ610_12030 | WND83023.1 | 271,72 | 2,58E-196 | | | alginate lyase family protein |
| RJ610_12035 | WND83024.1 | 408,38 | 1,73E-287 | | | bi-domain-containing oxidoreductase |
| RJ610_12040 | WND83025.1 | 480,62 | 9,30E-168 | | | hypothetical protein |
| RJ610_12045 | WND83026.1 | 763,41 | 3,33E-196 | | | hypothetical protein |
| RJ610_12050 | WND83027.1 | 250,74 | 2,93E-149 | | | O-antigen ligase family protein |
| RJ610_12055 | WND83028.1 | 761,87 | 4,97E-169 | | | acyltransferase |
| RJ610_12060 | WND83029.1 | 1136,69 | 1,91E-183 | | | oligosaccharide flippase family protein |
| RJ610_12065 | WND83030.1 | 894,14 | 2,14E-272 | | | UDP-N-acetyl-D-mannosamine dehydrogenase |

| | | | | | | |
|---|---|---|---|---|---|---|
| RJ610_12070 | WND83031.1 | 755,88 | 0,00E+00 | | | UDP-N-acetylglucosamine 2-epimerase (non-hydrolyzing) |
| RJ610_12075 | WND83032.1 | 349,91 | 0,00E+00 | | | polysaccharide biosynthesis tyrosine autokinase |
| RJ610_12080 | WND83033.1 | 363,79 | 6,06E-290 | | | low molecular weight protein-tyrosine-phosphatase |
| RJ610_12085 | WND83034.1 | 94,15 | 3,52E-210 | | | polysaccharide biosynthesis/export family protein |
| RJ610_12090 | WND83035.1 | 8,09 | 2,64E-66 | | | CocE/NonD family hydrolase |
| RJ610_12095 | WND83036.1 | 2,26 | 9,78E-14 | | | methyl-accepting chemotaxis protein |
| RJ610_12100 | WND83037.1 | 2,18 | 2,30E-10 | | | chemotaxis protein CheW |
| RJ610_12105 | WND83038.1 | 1,95 | 1,81E-07 | | | CheR family methyltransferase |
| RJ610_12110 | WND83039.1 | 1,75 | 2,31E-06 | | | chemotaxis protein CheW |
| RJ610_12115 | WND83040.1 | 1,55 | 8,69E-05 | | | hybrid sensor histidine kinase/response regulator |
| RJ610_12120 | WND83041.1 | 1,73 | 1,18E-05 | | | chemotaxis response regulator protein-glutamate methylesterase |
| RJ610_12125 | WND83042.1 | 1,78 | 5,95E-07 | | | diguanylate cyclase |
| RJ610_12130 | WND83043.1 | 7,79 | 1,31E-37 | | | GH-E family nuclease |
| RJ610_12135 | WND83044.1 | 2,83 | 9,19E-05 | | | hypothetical protein |
| RJ610_12140 | WND83045.1 | 1,45 | 2,41E-02 | | | hypothetical protein |
| RJ610_12145 | WND83046.1 | 1,34 | 1,16E-01 | | | hypothetical protein |
| RJ610_12150 | WND83047.1 | 2,70 | 6,37E-10 | | | M15 family metallopeptidase |
| RJ610_12155 | WND83048.1 | 1,24 | 1,10E-01 | | | PAAR domain-containing protein |
| RJ610_12160 | WND83049.1 | 0,95 | 8,14E-01 | | | hypothetical protein |
| RJ610_12165 | WND83050.1 | 0,66 | 1,80E-03 | | | type VI secretion system membrane subunit TssM |
| RJ610_12170 | WND83051.1 | 0,33 | 2,87E-04 | | | type VI secretion system-associated protein TagF |
| RJ610_12175 | WND83052.1 | 0,31 | 7,36E-06 | | | OmpA family protein |
| RJ610_12180 | WND83053.1 | 2,23 | 2,93E-05 | | | type VI secretion system tip protein TssI/VgrG |
| RJ610_12185 | WND83054.1 | 1,15 | 6,06E-01 | | | hypothetical protein |
| RJ610_12190 | WND83055.1 | 2,10 | 7,27E-13 | | | RHS repeat-associated core domain-containing protein |
| RJ610_12195 | WND83056.1 | 2,07 | 3,88E-10 | | | hypothetical protein |
| RJ610_12200 | WND83057.1 | 1,42 | 2,87E-03 | | | type VI secretion system protein TssA |
| RJ610_12205 | WND83243.1 | 1,74 | 5,40E-06 | | | type VI secretion system Vgr family protein |
| RJ610_12210 | WND83058.1 | 2,23 | 9,69E-09 | | | DUF4123 domain-containing protein |
| RJ610_12215 | WND83059.1 | 0,92 | 7,46E-01 | | | hypothetical protein |
| RJ610_12220 | WND83060.1 | 1,26 | 3,07E-02 | | | DUF3304 domain-containing protein |
| RJ610_12225 | WND83061.1 | 1,29 | 4,65E-02 | | | DUF2235 domain-containing protein |
| RJ610_12230 | WND83062.1 | 2,35 | 5,19E-14 | | | hypothetical protein |
| RJ610_12235 | WND83063.1 | 1,79 | 5,45E-08 | | | hypothetical protein |
| RJ610_12240 | WND83064.1 | 0,99 | 9,09E-01 | | | type VI secretion system Vgr family protein |
| RJ610_12245 | WND83065.1 | 0,80 | 7,44E-02 | | | type IVB secretion system protein IcmH/DotU |
| RJ610_12250 | WND83066.1 | 1,43 | 1,14E-03 | | | type VI secretion system baseplate subunit TssK |
| RJ610_12255 | WND83067.1 | 3,76 | 1,81E-26 | | | type VI secretion system lipoprotein TssJ |
| RJ610_12260 | WND83068.1 | 0,97 | 7,84E-01 | | | hypothetical protein |
| RJ610_12265 | WND83069.1 | 0,49 | 1,30E-09 | | | type VI secretion system contractile sheath small subunit |
| RJ610_12270 | WND83070.1 | 0,34 | 7,47E-27 | | | type VI secretion system contractile sheath large subunit |
| RJ610_12275 | WND83071.1 | 0,13 | 2,07E-48 | | | type VI secretion system tube protein Hcp |
| RJ610_12280 | WND83072.1 | 0,55 | 1,60E-02 | | | type VI secretion system baseplate subunit TssE |

| | | | | | | |
|---|---|---|---|---|---|---|
| RJ610_12285 | WND83073.1 | 0,76 | 1,48E-01 | | | type VI secretion system baseplate subunit TssF |
| RJ610_12290 | WND83074.1 | 0,89 | 6,40E-01 | | | type VI secretion system baseplate subunit TssG |
| RJ610_12295 | WND83075.1 | 0,78 | 1,24E-01 | | | type VI secretion system ATPase TssH |
| RJ610_12300 | WND83076.1 | 0,91 | 5,17E-01 | | | type VI secretion system tip protein TssI/VgrG |
| RJ610_12305 | WND83077.1 | 3,17 | 6,09E-13 | | | DUF4123 domain-containing protein |
| RJ610_12310 | WND83078.1 | 1,93 | 7,31E-07 | | | hypothetical protein |
| RJ610_12315 | WND83079.1 | 3,46 | 3,13E-26 | | | hypothetical protein |
| RJ610_12320 | WND83080.1 | 3,19 | 4,33E-27 | | | hypothetical protein |
| RJ610_12325 | WND83081.1 | 2,79 | 2,04E-13 | | | malate dehydrogenase (quinone) |
| RJ610_12330 | WND83082.1 | 13,37 | 2,37E-38 | | | hypothetical protein |
| RJ610_12340 | WND83084.1 | 162,61 | 0,00E+00 | | | DUF6289 family protein |
| RJ610_12345 | WND83085.1 | 43,78 | 5,44E-217 | | | GFA family protein |
| RJ610_12350 | WND83086.1 | 4,75 | 2,51E-38 | | | efflux RND transporter permease subunit |
| RJ610_12355 | WND83087.1 | 1,04 | 8,22E-01 | | | efflux RND transporter periplasmic adaptor subunit |
| RJ610_12360 | WND83088.1 | 0,52 | 1,16E-08 | | | cupin domain-containing protein |
| RJ610_12365 | WND83089.1 | 0,52 | 4,49E-04 | | | hypothetical protein |
| RJ610_12370 | WND83090.1 | 0,56 | 1,76E-03 | | | hypothetical protein |
| RJ610_12375 | WND83091.1 | 0,94 | 6,83E-01 | | | glycosyltransferase family 4 protein |
| RJ610_12380 | WND83092.1 | 1,19 | 1,55E-01 | | | amino acid adenylation domain-containing protein |
| RJ610_12385 | WND83093.1 | 1,88 | 5,52E-08 | | | hypothetical protein |
| RJ610_12390 | WND83244.1 | 0,97 | 7,88E-01 | | | non-ribosomal peptide synthase/polyketide synthase |
| RJ610_12395 | WND83094.1 | 0,83 | 3,40E-01 | | | M28 family peptidase |
| RJ610_12400 | WND83095.1 | 0,88 | 5,64E-01 | | | cyclic peptide export ABC transporter |
| RJ610_12405 | WND83096.1 | 0,97 | 8,22E-01 | | | amino acid adenylation domain-containing protein |
| RJ610_12410 | WND83097.1 | 0,67 | 6,35E-02 | | | thiol:disulfide interchange protein DsbA/DsbL |
| RJ610_12415 | WND83098.1 | 0,77 | 2,05E-01 | | | cysteine synthase A |
| RJ610_12420 | WND83099.1 | 0,85 | 2,49E-01 | | | amino acid adenylation domain-containing protein |
| RJ610_12425 | WND83100.1 | 0,75 | 1,84E-02 | | | amino acid adenylation domain-containing protein |
| RJ610_12430 | WND83101.1 | 1,11 | 5,17E-01 | | | thioesterase domain-containing protein |
| RJ610_12435 | WND83102.1 | 1,23 | 8,40E-02 | | | cupin domain-containing protein |
| RJ610_12440 | WND83103.1 | 2,53 | 1,29E-13 | | | DUF4238 domain-containing protein |
| RJ610_12445 | WND83104.1 | 1,63 | 8,74E-06 | | | TonB-dependent receptor |
| RJ610_12450 | WND83105.1 | 0,63 | 2,81E-02 | | | MFS transporter |
| RJ610_12455 | WND83106.1 | 0,94 | 7,26E-01 | | | carbohydrate kinase family protein |
| RJ610_12460 | WND83107.1 | 1,09 | 4,55E-01 | | | ROK family transcriptional regulator |
| RJ610_12470 | WND83109.1 | 1,39 | 6,38E-04 | | | DUF4238 domain-containing protein |
| RJ610_12475 | WND83110.1 | 1,27 | 5,21E-01 | | | hypothetical protein |
| RJ610_12480 | WND83111.1 | 1,07 | 7,74E-01 | | | adhesin |
| RJ610_12485 | WND83112.1 | 0,55 | 2,63E-04 | | | single-stranded DNA-binding protein |
| RJ610_12490 | WND83113.1 | 0,88 | 6,09E-01 | | | capsid protein |
| RJ610_12495 | WND83114.1 | 1,19 | 4,32E-01 | | | hypothetical protein |
| RJ610_12500 | WND83115.1 | 0,80 | 5,18E-01 | | | phage coat protein |
| RJ610_12505 | WND83116.1 | 1,80 | 5,18E-05 | | | zonular occludens toxin domain-containing protein |

| | | | | | | |
|---|---|---|---|---|---|---|
| RJ610_12510 | WND83117.1 | 6,55 | 1,82E-36 | | | hypothetical protein |
| RJ610_12515 | WND83118.1 | 6,43 | 2,70E-14 | | | hypothetical protein |
| RJ610_12520 | WND83119.1 | 161,26 | 1,21E-299 | | | ABC transporter ATP-binding protein |
| RJ610_12525 | WND83120.1 | 153,57 | 4,47E-239 | | | TauD/TfdA family dioxygenase |
| RJ610_12530 | WND83121.1 | 83,96 | 1,04E-262 | | | non-ribosomal peptide synthase/polyketide synthase |
| RJ610_12535 | WND83122.1 | 45,57 | 1,18E-115 | | | non-ribosomal peptide synthase/polyketide synthase |
| RJ610_12540 | WND83123.1 | 46,88 | 3,39E-99 | | | MbtH family protein |
| RJ610_12545 | WND83124.1 | 4,00 | 6,27E-16 | | | hypothetical protein |
| RJ610_12550 | WND83125.1 | 2,87 | 1,01E-05 | | | DUF2272 domain-containing protein |
| RJ610_12555 | WND83126.1 | 3,60 | 6,58E-18 | | | alpha/beta hydrolase |
| RJ610_12560 | WND83127.1 | 2,24 | 1,65E-14 | | | polymorphic toxin type 46 domain-containing protein |
| RJ610_12570 | WND83129.1 | 5,20 | 7,74E-38 | | | hypothetical protein |
| RJ610_12575 | WND83130.1 | 3,08 | 1,03E-37 | | | 2,3-dihydro-2,3-dihydroxybenzoate dehydrogenase |
| RJ610_12580 | WND83131.1 | 2,91 | 2,05E-34 | | | siderophore-interacting protein |
| RJ610_12585 | WND83132.1 | 2,93 | 3,78E-40 | | | condensation domain-containing protein |
| RJ610_12590 | WND83133.1 | 2,71 | 2,00E-31 | | | isochorismatase family protein |
| RJ610_12595 | WND83134.1 | 2,91 | 9,75E-32 | | | (2,3-dihydroxybenzoyl)adenylate synthase |
| RJ610_12600 | WND83135.1 | 3,59 | 2,48E-31 | | | isochorismate synthase |
| RJ610_12610 | WND83137.1 | 2,63 | 1,15E-10 | | | MBL fold metallo-hydrolase |
| RJ610_12615 | WND83138.1 | 1,88 | 9,30E-07 | | | CopL family metal-binding regulatory protein |
| RJ610_12620 | WND83139.1 | 1,82 | 2,33E-08 | | | copper resistance system multicopper oxidase |
| RJ610_12625 | WND83140.1 | 1,69 | 1,13E-03 | | | copper resistance protein B |
| RJ610_12630 | WND83141.1 | 1,98 | 3,29E-13 | | | hypothetical protein |
| RJ610_12635 | WND83142.1 | 1,37 | 1,56E-02 | | | FAD-dependent oxidoreductase |
| RJ610_12640 | WND83143.1 | 1,35 | 6,89E-02 | | | transporter substrate-binding domain-containing protein |
| RJ610_12645 | WND83144.1 | 1,44 | 2,11E-01 | | | response regulator |
| RJ610_12650 | WND83145.1 | 0,49 | 1,64E-07 | | | OmpA family protein |
| RJ610_12655 | WND83146.1 | 1,11 | 3,93E-01 | | | wax ester/triacylglycerol synthase family O-acyltransferase |
| RJ610_12660 | WND83147.1 | 1,37 | 4,93E-03 | | | deoxyribodipyrimidine photo-lyase |
| RJ610_12670 | WND83149.1 | 0,86 | 3,31E-01 | | | alanine racemase |
| RJ610_12675 | WND83245.1 | 1,21 | 1,94E-01 | | | glycine zipper 2TM domain-containing protein |
| RJ610_12680 | WND83150.1 | 52,25 | 1,25E-180 | | | FAD-dependent oxidoreductase |
| RJ610_12685 | WND83151.1 | 486,81 | 0,00E+00 | | | methyltransferase domain-containing protein |
| RJ610_12690 | WND83152.1 | 827,42 | 0,00E+00 | | | hypothetical protein |
| RJ610_12695 | WND83153.1 | 482,77 | 6,48E-196 | | | hypothetical protein |
| RJ610_12700 | WND83154.1 | 586,59 | 0,00E+00 | | | HEXXH motif-containing putative peptide modification protein |
| RJ610_12705 | WND78177.1 | 321,53 | 0,00E+00 | | | PqqD family protein |
| RJ610_12710 | WND78178.1 | 5,03 | 2,63E-35 | | | hypothetical protein |
| RJ610_12715 | WND83246.1 | 0,80 | 2,25E-01 | | | rhodanese-like domain-containing protein |
| RJ610_12720 | WND78179.1 | 0,78 | 1,09E-01 | | | replicative DNA helicase |
| RJ610_12725 | WND78180.1 | 0,78 | 4,23E-02 | | | 50S ribosomal protein L9 |
| RJ610_12730 | WND78181.1 | 0,72 | 1,87E-03 | | | 30S ribosomal protein S18 |
| RJ610_12735 | WND78182.1 | 0,73 | 2,61E-03 | | | 30S ribosomal protein S6 |

| | | | | | | |
|---|---|---|---|---|---|---|
| RJ610_12740 | WND78183.1 | 0,55 | 1,09E-05 | | | DUF885 domain-containing protein |
| RJ610_12745 | WND78184.1 | 1,66 | 1,34E-02 | | | iron-sulfur cluster assembly accessory protein |
| RJ610_12750 | WND78185.1 | 0,66 | 2,75E-03 | | | DUF2891 domain-containing protein |
| RJ610_12755 | WND78186.1 | 0,80 | 1,84E-02 | | | asparagine--tRNA ligase |
| RJ610_12760 | WND78187.1 | 0,90 | 5,12E-01 | | | hypothetical protein |
| RJ610_12765 | WND78188.1 | 0,96 | 7,91E-01 | | | hypothetical protein |
| RJ610_12770 | WND78189.1 | 1,29 | 1,06E-01 | | | FMN-binding negative transcriptional regulator |
| RJ610_12775 | WND78190.1 | 1,57 | 6,63E-04 | | | SDR family oxidoreductase |
| RJ610_12780 | WND78191.1 | 0,99 | 9,63E-01 | | | carboxypeptidase regulatory-like domain-containing protein |
| RJ610_12785 | WND78192.1 | 0,87 | 4,34E-01 | | | hypothetical protein |
| RJ610_12790 | WND78193.1 | 0,57 | 1,20E-02 | | | BatA domain-containing protein |
| RJ610_12795 | WND78194.1 | 0,78 | 1,92E-01 | | | DUF58 domain-containing protein |
| RJ610_12800 | WND78195.1 | 0,76 | 8,20E-02 | | | MoxR family ATPase |
| RJ610_12805 | WND78196.1 | 1,05 | 8,19E-01 | | | hypothetical protein |
| RJ610_12810 | WND78197.1 | 0,84 | 2,71E-01 | | | DUF4159 domain-containing protein |
| RJ610_12815 | WND78198.1 | 0,60 | 5,66E-04 | | | TldD/PmbA family protein |
| RJ610_12830 | WND78201.1 | 1,64 | 1,44E-04 | | | hypothetical protein |
| RJ610_12835 | WND78202.1 | 1,17 | 1,71E-01 | | | aldehyde dehydrogenase |
| RJ610_12845 | WND78204.1 | 1,10 | 5,12E-01 | | | RidA family protein |
| RJ610_12850 | WND78205.1 | 1,06 | 6,72E-01 | | | 3-hydroxyanthranilate 3,4-dioxygenase |
| RJ610_12855 | WND78206.1 | 1,24 | 9,14E-02 | | | amidohydrolase family protein |
| RJ610_12860 | WND78207.1 | 1,26 | 1,44E-01 | | | hypothetical protein |
| RJ610_12865 | WND78208.1 | 1,01 | 9,22E-01 | | | kynureninase |
| RJ610_12870 | WND78209.1 | 1,15 | 2,60E-01 | | | GIY-YIG nuclease family protein |
| RJ610_12880 | WND78211.1 | 0,99 | 9,42E-01 | | | exodeoxyribonuclease I |
| RJ610_12885 | WND78212.1 | 1,50 | 2,75E-02 | | | DUF2461 domain-containing protein |
| RJ610_12890 | WND78213.1 | 1,36 | 1,33E-01 | | | MFS transporter |
| RJ610_12895 | WND83247.1 | 2,35 | 3,50E-04 | | | hypothetical protein |
| RJ610_12900 | WND78214.1 | 2,20 | 7,84E-05 | | | DUF4785 family protein |
| RJ610_12905 | WND78215.1 | 0,68 | 2,74E-02 | | | DUF2939 domain-containing protein |
| RJ610_12910 | WND78216.1 | 0,23 | 7,42E-23 | | | lysozyme inhibitor LprI family protein |
| RJ610_12915 | WND78217.1 | 0,90 | 3,74E-01 | | | 5'-nucleotidase |
| RJ610_12920 | WND78218.1 | 1,32 | 3,22E-02 | | | amino acid permease |
| RJ610_12925 | WND78219.1 | 1,26 | 1,44E-01 | | | hypothetical protein |
| RJ610_12930 | WND78220.1 | 1,42 | 3,26E-04 | | | NAD kinase |
| RJ610_12935 | WND78221.1 | 1,14 | 4,11E-01 | | | hypothetical protein |
| RJ610_12940 | WND78222.1 | 0,81 | 1,47E-01 | | | NAD-glutamate dehydrogenase |
| RJ610_12945 | WND78223.1 | 0,85 | 1,37E-01 | | | acyl-CoA dehydrogenase family protein |
| RJ610_12950 | WND78224.1 | 2,41 | 9,32E-06 | | | metalloregulator ArsR/SmtB family transcription factor |
| RJ610_12955 | WND78225.1 | 3,05 | 1,35E-05 | | | homocysteine S-methyltransferase family protein |
| RJ610_12960 | WND78226.1 | 3,54 | 2,50E-06 | | | methionine synthase |
| RJ610_12965 | WND78227.1 | 0,95 | 8,11E-01 | | | hypothetical protein |
| RJ610_12975 | WND78229.1 | 0,37 | 3,91E-12 | | | hypothetical protein |

| | | | | | | |
|---|---|---|---|---|---|---|
| RJ610_12985 | WND78231.1 | 0,75 | 7,11E-02 | | | helix-turn-helix domain-containing protein |
| RJ610_12990 | WND78232.1 | 0,62 | 3,02E-03 | | | SDR family oxidoreductase |
| RJ610_12995 | WND78233.1 | 1,18 | 3,22E-01 | | | alpha/beta hydrolase |
| RJ610_13000 | WND78234.1 | 1,48 | 2,14E-03 | | | DUF2058 family protein |
| RJ610_13005 | WND78235.1 | 1,62 | 1,88E-04 | | | SlyX family protein |
| RJ610_13010 | WND78236.1 | 2,37 | 2,32E-12 | | | UDP-glucose/GDP-mannose dehydrogenase family protein |
| RJ610_13015 | WND78237.1 | 2,57 | 1,17E-18 | | | FKBP-type peptidyl-prolyl cis-trans isomerase |
| RJ610_13020 | WND78238.1 | 2,42 | 4,86E-12 | | | FKBP-type peptidyl-prolyl cis-trans isomerase |
| RJ610_13025 | WND78239.1 | 0,84 | 9,92E-02 | | | glutathione peroxidase |
| RJ610_13030 | WND78240.1 | 0,02 | 1,72E-82 | | | hypothetical protein |
| RJ610_13035 | WND78241.1 | 0,04 | 7,45E-71 | | | GntR family transcriptional regulator |
| RJ610_13040 | WND78242.1 | 0,06 | 3,81E-49 | | | ABC transporter ATP-binding protein |
| RJ610_13045 | WND78243.1 | 0,11 | 1,54E-34 | | | ABC transporter permease |
| RJ610_13050 | WND78244.1 | 0,18 | 1,49E-25 | | | DUF4097 family beta strand repeat-containing protein |
| RJ610_13055 | WND78245.1 | 0,20 | 1,05E-18 | | | hypothetical protein |
| RJ610_13060 | WND78246.1 | 0,24 | 6,89E-18 | | | hypothetical protein |
| RJ610_13065 | WND78247.1 | 0,60 | 2,17E-04 | | | inner membrane protein YiaA |
| RJ610_13070 | WND78248.1 | 0,70 | 8,89E-03 | | | FadR/GntR family transcriptional regulator |
| RJ610_13075 | WND78249.1 | 1,72 | 8,34E-04 | | | alpha-galactosidase |
| RJ610_13080 | WND78250.1 | 2,31 | 7,23E-03 | | | aldose 1-epimerase |
| RJ610_13085 | WND78251.1 | 1,45 | 3,95E-01 | | | hypothetical protein |
| RJ610_13090 | WND78252.1 | 1,94 | 3,68E-07 | | | sodium/solute symporter |
| RJ610_13095 | WND78253.1 | 1,18 | 5,87E-01 | | | 2-dehydro-3-deoxygalactonokinase |
| RJ610_13100 | WND78254.1 | 1,43 | 4,80E-02 | | | SMP-30/gluconolactonase/LRE family protein |
| RJ610_13105 | WND78255.1 | 1,68 | 1,48E-03 | | | galactonate dehydratase |
| RJ610_13110 | WND78256.1 | 1,41 | 1,05E-01 | | | 2-dehydro-3-deoxy-6-phosphogalactonate aldolase |
| RJ610_13115 | WND78257.1 | 1,80 | 3,92E-03 | | | SDR family oxidoreductase |
| RJ610_13120 | WND78258.1 | 2,21 | 7,88E-09 | | | glycoside hydrolase family 2 TIM barrel-domain containing protein |
| RJ610_13125 | WND78259.1 | 4,24 | 2,38E-18 | | | TonB-dependent receptor |
| RJ610_13130 | WND78260.1 | 5,14 | 7,93E-43 | | | hypothetical protein |
| RJ610_13135 | WND78261.1 | 1,61 | 9,92E-03 | | | class II fumarate hydratase |
| RJ610_13140 | WND78262.1 | 0,96 | 7,10E-01 | | | adenylosuccinate lyase |
| RJ610_13145 | WND78263.1 | 0,89 | 5,97E-01 | | | subclass B3 metallo-beta-lactamase |
| RJ610_13150 | WND78264.1 | 0,76 | 1,33E-01 | | | VOC family protein |
| RJ610_13155 | WND78265.1 | 0,77 | 1,55E-01 | | | YoaK family protein |
| RJ610_13160 | WND78266.1 | 2,36 | 1,12E-12 | | | cupin domain-containing protein |
| RJ610_13165 | WND78267.1 | 1,71 | 3,56E-04 | | | GNAT family N-acetyltransferase |
| RJ610_13175 | WND78269.1 | 1,26 | 4,00E-02 | | | dihydrolipoyllysine-residue succinyltransferase |
| RJ610_13185 | WND78271.1 | 1,18 | 2,46E-01 | | | TIGR00730 family Rossman fold protein |
| RJ610_13190 | WND78272.1 | 0,91 | 4,70E-01 | | | hypothetical protein |
| RJ610_13195 | WND78273.1 | 2,27 | 1,69E-11 | | | YhdH/YhfP family quinone oxidoreductase |
| RJ610_13200 | WND78274.1 | 1,24 | 8,89E-02 | | | response regulator |
| RJ610_13205 | WND78275.1 | 1,03 | 8,96E-01 | | | DnaJ C-terminal domain-containing protein |

| | | | | | | |
|---|---|---|---|---|---|---|
| RJ610_13210 | WND78276.1 | 0,64 | 1,39E-04 | | | peroxiredoxin |
| RJ610_13215 | WND78277.1 | 0,75 | 3,18E-02 | | | adenosine deaminase |
| RJ610_13220 | WND78278.1 | 0,90 | 5,94E-01 | | | hypothetical protein |
| RJ610_13225 | WND83248.1 | 0,38 | 5,35E-12 | | | penicillin-binding protein 1C |
| RJ610_13230 | WND78279.1 | 0,56 | 8,27E-08 | | | hypothetical protein |
| RJ610_13235 | WND78280.1 | 0,44 | 6,99E-09 | | | hypothetical protein |
| RJ610_13240 | WND83249.1 | 0,45 | 3,62E-18 | | | alpha-2-macroglobulin |
| RJ610_13260 | WND78284.1 | 1,91 | 5,53E-05 | | | hypothetical protein |
| RJ610_13265 | WND78285.1 | 0,84 | 4,34E-01 | | | DUF1428 domain-containing protein |
| RJ610_13270 | WND78286.1 | 1,19 | 2,87E-01 | | | MFS transporter |
| RJ610_13285 | WND78288.1 | 1,33 | 3,02E-01 | | | carbohydrate porin |
| RJ610_13290 | WND78289.1 | 1,93 | 2,47E-02 | | | CoA-acylating methylmalonate-semialdehyde dehydrogenase |
| RJ610_13295 | WND78290.1 | 2,00 | 1,84E-02 | | | 5-deoxy-glucuronate isomerase |
| RJ610_13300 | WND78291.1 | 2,58 | 2,12E-01 | | | myo-inosose-2 dehydratase |
| RJ610_13305 | WND78292.1 | 1,18 | 7,88E-01 | | | 3D-(3,5/4)-trihydroxycyclohexane-1,2-dione acylhydrolase (decyclizing) |
| RJ610_13310 | WND78293.1 | 2,41 | 1,38E-03 | | | 5-dehydro-2-deoxygluconokinase |
| RJ610_13315 | WND78294.1 | 1,70 | 5,93E-02 | | | ATP-binding cassette domain-containing protein |
| RJ610_13320 | WND78295.1 | 1,67 | 2,24E-01 | | | ABC transporter permease |
| RJ610_13325 | WND78296.1 | 1,72 | 2,74E-02 | | | sugar ABC transporter substrate-binding protein |
| RJ610_13330 | WND78297.1 | 0,85 | 3,83E-01 | | | MurR/RpiR family transcriptional regulator |
| RJ610_13335 | WND78298.1 | 1,27 | 2,39E-01 | | | inositol 2-dehydrogenase |
| RJ610_13340 | WND78299.1 | 0,69 | 2,32E-03 | | | Gfo/Idh/MocA family oxidoreductase |
| RJ610_13345 | WND78300.1 | 1,14 | 2,74E-01 | | | DNA gyrase subunit A |
| RJ610_13350 | WND78301.1 | 0,83 | 1,24E-01 | | | S-methyl-5-thioribose-1-phosphate isomerase |
| RJ610_13355 | WND78302.1 | 0,84 | 1,39E-01 | | | DUF3011 domain-containing protein |
| RJ610_13360 | WND78303.1 | 0,47 | 4,23E-07 | | | DUF3011 domain-containing protein |
| RJ610_13365 | WND78304.1 | 0,78 | 7,81E-02 | | | EF-P lysine aminoacylase EpmA |
| RJ610_13370 | WND78305.1 | 0,59 | 1,82E-05 | | | NAD-dependent DNA ligase LigA |
| RJ610_13375 | WND78306.1 | 1,28 | 6,99E-02 | | | cell division protein ZipA |
| RJ610_13380 | WND78307.1 | 1,29 | 1,49E-02 | | | chromosome segregation protein SMC |
| RJ610_13385 | WND78308.1 | 1,05 | 6,69E-01 | | | BlaI/MecI/CopY family transcriptional regulator |
| RJ610_13390 | WND78309.1 | 0,84 | 1,38E-01 | | | M56 family metallopeptidase |
| RJ610_13400 | WND78311.1 | 0,92 | 6,10E-01 | | | LacI family DNA-binding transcriptional regulator |
| RJ610_13415 | WND78314.1 | 2,23 | 2,65E-10 | | | MFS transporter |
| RJ610_13420 | WND78315.1 | 0,79 | 1,69E-01 | | | TerD family protein |
| RJ610_13425 | WND78316.1 | 0,92 | 8,63E-01 | | | hypothetical protein |
| RJ610_13430 | WND78317.1 | 0,50 | 7,23E-05 | | | aldehyde reductase |
| RJ610_13435 | WND78318.1 | 0,57 | 4,79E-04 | | | hypothetical protein |
| RJ610_13440 | WND78319.1 | 0,91 | 5,72E-01 | | | DUF2235 domain-containing protein |
| RJ610_13445 | WND78320.1 | 1,06 | 6,76E-01 | | | hypothetical protein |
| RJ610_13450 | WND78321.1 | 0,88 | 3,65E-01 | | | sn-glycerol-3-phosphate ABC transporter ATP-binding protein UgpC |

| | | | | | | |
|---|---|---|---|---|---|---|
| RJ610_13460 | WND78323.1 | 1,24 | 8,64E-02 | | | glucose-6-phosphate dehydrogenase |
| RJ610_13470 | WND78325.1 | 1,61 | 1,91E-04 | | | 6-phosphogluconolactonase |
| RJ610_13475 | WND83251.1 | 1,91 | 7,08E-08 | | | discoidin domain-containing protein |
| RJ610_13480 | WND78326.1 | 1,52 | 1,72E-03 | | | carbohydrate ABC transporter permease |
| RJ610_13485 | WND78327.1 | 1,46 | 1,23E-02 | | | sugar ABC transporter permease |
| RJ610_13490 | WND83252.1 | 1,32 | 6,40E-02 | | | sugar ABC transporter substrate-binding protein |
| RJ610_13505 | WND78330.1 | 1,28 | 6,14E-02 | | | LacI family DNA-binding transcriptional regulator |
| RJ610_13510 | WND83253.1 | 1,23 | 2,43E-01 | | | BolA family protein |
| RJ610_13515 | WND78331.1 | 0,94 | 7,26E-01 | | | YciI family protein |
| RJ610_13520 | WND78332.1 | 0,45 | 6,57E-13 | | | peptidoglycan-binding domain-containing protein |
| RJ610_13525 | WND78333.1 | 0,83 | 1,11E-01 | | | hypothetical protein |
| RJ610_13530 | WND78334.1 | 1,30 | 2,25E-02 | | | ScpA family protein |
| RJ610_13535 | WND78335.1 | 1,25 | 6,89E-02 | | | SMC-Scp complex subunit ScpB |
| RJ610_13540 | WND78336.1 | 1,21 | 9,56E-02 | | | pseudouridine synthase |
| RJ610_13550 | WND78338.1 | 1,14 | 7,03E-01 | | | nuclear transport factor 2 family protein |
| RJ610_13555 | WND78339.1 | 0,79 | 2,43E-01 | | | TetR/AcrR family transcriptional regulator |
| RJ610_13560 | WND78340.1 | 1,35 | 2,52E-01 | | | NAD(P)/FAD-dependent oxidoreductase |
| RJ610_13565 | WND78341.1 | 0,72 | 7,98E-04 | | | hypothetical protein |
| RJ610_13575 | WND78343.1 | 0,54 | 6,99E-08 | | | type IV secretory system conjugative DNA transfer family protein |
| RJ610_13580 | WND78344.1 | 1,49 | 1,86E-03 | | | hypothetical protein |
| RJ610_13585 | WND78345.1 | 1,82 | 2,23E-05 | | | hypothetical protein |
| RJ610_13600 | WND78348.1 | 3,81 | 4,72E-23 | | | hypothetical protein |
| RJ610_13610 | WND78350.1 | 1,48 | 1,23E-04 | | | hypothetical protein |
| RJ610_13615 | WND78351.1 | 1,91 | 2,80E-07 | | | hypothetical protein |
| RJ610_13625 | WND78353.1 | 2,14 | 3,56E-19 | | | hypothetical protein |
| RJ610_13630 | WND78354.1 | 2,63 | 1,16E-06 | | | response regulator |
| RJ610_13635 | WND78355.1 | 3,11 | 1,53E-14 | | | ATP-binding protein |
| RJ610_13640 | WND78356.1 | 3,06 | 1,18E-17 | | | hypothetical protein |
| RJ610_13650 | WND78358.1 | 41,17 | 3,77E-149 | | | TonB-dependent receptor |
| RJ610_13655 | WND78359.1 | 1227,19 | 1,08E-260 | | | DUF1800 domain-containing protein |
| RJ610_13665 | WND78361.1 | 9,92 | 3,83E-31 | | | LysR family transcriptional regulator |
| RJ610_13670 | WND78362.1 | 0,62 | 4,02E-03 | | | SDR family oxidoreductase |
| RJ610_13675 | WND78363.1 | 2,09 | 5,04E-02 | | | SDR family NAD(P)-dependent oxidoreductase |
| RJ610_13680 | WND78364.1 | 1,00 | 9,92E-01 | | | hypothetical protein |
| RJ610_13685 | WND78365.1 | 2,06 | 1,71E-03 | | | hypothetical protein |
| RJ610_13690 | WND78366.1 | 2,12 | 7,31E-03 | | | DUF5671 domain-containing protein |
| RJ610_13695 | WND78367.1 | 0,49 | 6,64E-08 | | | hypothetical protein |
| RJ610_13700 | WND78368.1 | 0,69 | 4,38E-03 | | | hypothetical protein |
| RJ610_13705 | WND78369.1 | 0,80 | 3,36E-01 | | | hypothetical protein |
| RJ610_13710 | WND78370.1 | 1,18 | 3,17E-01 | | | hypothetical protein |
| RJ610_13715 | WND78371.1 | 1,52 | 2,59E-04 | | | hypothetical protein |
| RJ610_13720 | WND78372.1 | 1,13 | 3,30E-01 | | | CPBP family glutamic-type intramembrane protease |
| RJ610_13725 | WND78373.1 | 1,29 | 8,72E-02 | | | hypothetical protein |

| | | | | | | |
|---|---|---|---|---|---|---|
| RJ610_13735 | WND78375.1 | 0,59 | 2,97E-07 | | | 30S ribosomal protein S15 |
| RJ610_13740 | WND78376.1 | 1,16 | 1,55E-01 | | | tRNA pseudouridine(55) synthase TruB |
| RJ610_13750 | WND78378.1 | 1,30 | 8,01E-03 | | | 30S ribosome-binding factor RbfA |
| RJ610_13755 | WND78379.1 | 1,03 | 8,29E-01 | | | translation initiation factor IF-2 |
| RJ610_13760 | WND78380.1 | 0,70 | 1,38E-04 | | | transcription termination factor NusA |
| RJ610_13765 | WND78381.1 | 0,51 | 3,36E-11 | | | ribosome maturation factor RimP |
| RJ610_13770 | | 0,91 | 4,66E-01 | | | tRNA-Met |
| RJ610_13775 | WND78382.1 | 1,40 | 1,84E-02 | | | NADH-quinone oxidoreductase subunit NuoN |
| RJ610_13780 | WND78383.1 | 1,38 | 2,44E-02 | | | NADH-quinone oxidoreductase subunit M |
| RJ610_13785 | WND78384.1 | 1,30 | 3,22E-02 | | | NADH-quinone oxidoreductase subunit L |
| RJ610_13790 | WND78385.1 | 1,79 | 3,85E-05 | | | NADH-quinone oxidoreductase subunit NuoK |
| RJ610_13795 | WND78386.1 | 1,09 | 6,47E-01 | | | NADH-quinone oxidoreductase subunit J |
| RJ610_13800 | WND78387.1 | 1,61 | 6,50E-05 | | | NADH-quinone oxidoreductase subunit NuoI |
| RJ610_13805 | WND83254.1 | 1,50 | 8,36E-04 | | | NADH-quinone oxidoreductase subunit NuoH |
| RJ610_13810 | WND78388.1 | 1,53 | 9,33E-04 | | | NADH-quinone oxidoreductase subunit NuoG |
| RJ610_13815 | WND78389.1 | 1,55 | 1,58E-04 | | | NADH-quinone oxidoreductase subunit NuoF |
| RJ610_13820 | WND78390.1 | 1,32 | 7,90E-02 | | | NADH-quinone oxidoreductase subunit NuoE |
| RJ610_13825 | WND83255.1 | 1,58 | 2,89E-05 | | | NADH-quinone oxidoreductase subunit D |
| RJ610_13830 | WND78391.1 | 1,55 | 1,12E-03 | | | NADH-quinone oxidoreductase subunit C |
| RJ610_13835 | WND78392.1 | 1,53 | 1,07E-04 | | | NADH-quinone oxidoreductase subunit B family protein |
| RJ610_13840 | WND78393.1 | 1,86 | 1,01E-12 | | | NADH-quinone oxidoreductase subunit A |
| RJ610_13845 | | 1,99 | 9,11E-12 | | | tRNA-Leu |
| RJ610_13850 | WND78394.1 | 0,88 | 2,21E-01 | | | preprotein translocase subunit SecG |
| RJ610_13860 | WND78396.1 | 2,01 | 9,91E-04 | | | DNA-deoxyinosine glycosylase |
| RJ610_13865 | WND78397.1 | 0,81 | 4,48E-01 | | | hypothetical protein |
| RJ610_13870 | WND78398.1 | 2,10 | 1,00E-09 | | | DUF6445 family protein |
| RJ610_13875 | WND78399.1 | 0,50 | 7,55E-12 | | | 2-oxoglutarate and iron-dependent oxygenase domain-containing protein |
| RJ610_13880 | WND78400.1 | 0,81 | 1,33E-01 | | | sulfatase-like hydrolase/transferase |
| RJ610_13885 | WND78401.1 | 0,93 | 5,21E-01 | | | phosphoglucosamine mutase |
| RJ610_13890 | WND78402.1 | 1,03 | 8,21E-01 | | | acetyl-CoA carboxylase, carboxyltransferase subunit beta |
| RJ610_13895 | WND78403.1 | 1,09 | 5,35E-01 | | | tryptophan synthase subunit alpha |
| RJ610_13900 | WND78404.1 | 1,05 | 7,22E-01 | | | tryptophan synthase subunit beta |
| RJ610_13905 | WND78405.1 | 0,73 | 1,33E-02 | | | phosphoribosylanthranilate isomerase |
| RJ610_13910 | WND78406.1 | 0,76 | 1,18E-02 | | | tRNA pseudouridine(38-40) synthase TruA |
| RJ610_13915 | WND78407.1 | 0,81 | 5,43E-02 | | | FimV/HubP family polar landmark protein |
| RJ610_13920 | WND78408.1 | 0,97 | 8,58E-01 | | | aspartate-semialdehyde dehydrogenase |
| RJ610_13925 | WND78409.1 | 0,92 | 5,71E-01 | | | D-glycerate dehydrogenase |
| RJ610_13930 | WND78410.1 | 1,00 | 9,74E-01 | | | chorismate synthase |
| RJ610_13935 | WND78411.1 | 0,63 | 1,66E-04 | | | 50S ribosomal protein L3 N(5)-glutamine methyltransferase |
| RJ610_13940 | WND78412.1 | 0,51 | 1,16E-08 | | | SCO family protein |
| RJ610_13945 | WND78413.1 | 0,70 | 1,62E-03 | | | archaetidylserine decarboxylase |
| RJ610_13950 | WND78414.1 | 0,94 | 7,46E-01 | | | hypothetical protein |

| | | | | | | |
|---|---|---|---|---|---|---|
| RJ610_13955 | | 1,18 | 7,17E-01 | | | arsenical resistance protein ArsH |
| RJ610_13960 | WND83256.1 | 1,16 | 3,39E-01 | | | EAL domain-containing protein |
| RJ610_13965 | WND78415.1 | 2,01 | 2,61E-06 | | | lysoplasmalogenase |
| RJ610_13970 | WND78416.1 | 1,41 | 9,80E-02 | | | hypothetical protein |
| RJ610_13975 | WND78417.1 | 0,62 | 1,50E-06 | | | transglycosylase SLT domain-containing protein |
| RJ610_13980 | WND78418.1 | 0,97 | 8,30E-01 | | | DUF853 family protein |
| RJ610_13985 | WND78419.1 | 0,82 | 1,48E-01 | | | copper resistance protein NlpE |
| RJ610_13990 | WND78420.1 | 1,12 | 3,56E-01 | | | transcription elongation factor GreB |
| RJ610_13995 | WND78421.1 | 2,64 | 7,66E-16 | | | cytochrome P450 |
| RJ610_14000 | WND78422.1 | 2,46 | 5,23E-10 | | | hypothetical protein |
| RJ610_14005 | WND78423.1 | 1,27 | 1,07E-01 | | | helix-turn-helix domain-containing protein |
| RJ610_14010 | | 0,97 | 8,72E-01 | | | FKBP-type peptidyl-prolyl cis-trans isomerase |
| RJ610_14015 | WND78424.1 | 0,02 | 2,06E-160 | | | roadblock/LC7 domain-containing protein |
| RJ610_14020 | WND78425.1 | 0,02 | 4,03E-153 | | | roadblock/LC7 domain-containing protein |
| RJ610_14025 | WND78426.1 | 0,02 | 4,06E-172 | | | hypothetical protein |
| RJ610_14030 | WND83257.1 | 0,27 | 4,69E-27 | | | ATP/GTP-binding protein |
| RJ610_14035 | WND78427.1 | 1,08 | 7,58E-01 | | | LLM class flavin-dependent oxidoreductase |
| RJ610_14040 | WND78428.1 | 1,37 | 3,04E-03 | | | hypothetical protein |
| RJ610_14045 | WND78429.1 | 1,42 | 1,05E-02 | | | hypothetical protein |
| RJ610_14050 | WND78430.1 | 0,42 | 3,72E-16 | | | alpha-2-macroglobulin |
| RJ610_14055 | WND78431.1 | 0,57 | 1,24E-02 | | | penicillin-binding protein 1C |
| RJ610_14060 | WND78432.1 | 1,04 | 8,52E-01 | | | hypothetical protein |
| RJ610_14065 | WND78433.1 | 1,19 | 2,00E-01 | | | hypothetical protein |
| RJ610_14070 | WND78434.1 | 2,89 | 9,97E-11 | | | sorbosone dehydrogenase family protein |
| RJ610_14075 | WND78435.1 | 1,82 | 5,38E-03 | | | LysR family transcriptional regulator |
| RJ610_14080 | WND78436.1 | 0,87 | 2,95E-01 | | | serine hydrolase |
| RJ610_14085 | WND78437.1 | 0,95 | 7,27E-01 | | | TIGR03862 family flavoprotein |
| RJ610_14090 | WND78438.1 | 2,34 | 4,41E-10 | | | hypothetical protein |
| RJ610_14095 | WND78439.1 | 1,13 | 3,05E-01 | | | sulfurtransferase |
| RJ610_14100 | WND78440.1 | 1,98 | 1,24E-03 | | | HPF/RaiA family ribosome-associated protein |
| RJ610_14105 | WND78441.1 | 2,37 | 1,06E-11 | | | hypothetical protein |
| RJ610_14110 | WND78442.1 | 1,17 | 4,19E-01 | | | hypothetical protein |
| RJ610_14115 | WND78443.1 | 1,64 | 1,21E-01 | | | hypothetical protein |
| RJ610_14120 | WND78444.1 | 1,23 | 1,96E-01 | | | putative Ig domain-containing protein |
| RJ610_14125 | WND78445.1 | 3,86 | 3,07E-10 | | | tail fiber protein |
| RJ610_14130 | WND78446.1 | 2,87 | 5,20E-07 | | | tail fiber protein |
| RJ610_14135 | WND78447.1 | 2,98 | 1,47E-08 | | | tail fiber protein |
| RJ610_14140 | WND78448.1 | 1,56 | 4,30E-02 | | | GNAT family N-acetyltransferase |
| RJ610_14145 | WND78449.1 | 0,69 | 1,33E-01 | | | hypothetical protein |
| RJ610_14150 | WND78450.1 | 0,73 | 2,30E-02 | | | hypothetical protein |
| RJ610_14155 | WND78451.1 | 0,65 | 2,12E-02 | | | Ig-like domain-containing protein |
| RJ610_14165 | WND78453.1 | 0,78 | 7,87E-02 | | | redox-sensitive transcriptional activator SoxR |
| RJ610_14170 | WND78454.1 | 0,70 | 3,32E-01 | | | NADP-dependent oxidoreductase |

| | | | | | | |
|---|---|---|---|---|---|---|
| RJ610_14175 | WND83258.1 | 1,31 | 2,15E-01 | | | MFS transporter |
| RJ610_14180 | WND78455.1 | 0,88 | 7,29E-01 | | | glycosyltransferase |
| RJ610_14185 | WND78456.1 | 1,11 | 4,66E-01 | | | phospholipase D-like domain-containing protein |
| RJ610_14190 | WND78457.1 | 1,03 | 8,75E-01 | | | hypothetical protein |
| RJ610_14195 | WND78458.1 | 0,77 | 2,53E-02 | | | hypothetical protein |
| RJ610_14200 | WND78459.1 | 0,89 | 7,49E-01 | | | hypothetical protein |
| RJ610_14205 | WND78460.1 | 1,45 | 4,51E-02 | | | hypothetical protein |
| RJ610_14215 | WND78462.1 | 0,85 | 3,26E-01 | | | M48 family metallopeptidase |
| RJ610_14220 | WND78463.1 | 0,82 | 6,27E-02 | | | 2OG-Fe(II) oxygenase |
| RJ610_14225 | WND78464.1 | 1,23 | 1,01E-01 | | | MFS transporter |
| RJ610_14230 | WND78465.1 | 0,90 | 5,07E-01 | | | oxidoreductase |
| RJ610_14235 | WND78466.1 | 0,95 | 7,88E-01 | | | carboxylate-amine ligase |
| RJ610_14240 | WND78467.1 | 1,50 | 1,68E-03 | | | type 1 glutamine amidotransferase |
| RJ610_14245 | WND78468.1 | 1369,92 | 2,05E-201 | | | S8 family serine peptidase |
| RJ610_14250 | WND78469.1 | 23,81 | 1,48E-43 | | | DUF4189 domain-containing protein |
| RJ610_14260 | WND78471.1 | 3,59 | 6,98E-36 | | | hypothetical protein |
| RJ610_14270 | WND78473.1 | 52,51 | 2,36E-09 | | | hypothetical protein |
| RJ610_14275 | WND78474.1 | 5,33 | 6,73E-25 | | | linear amide C-N hydrolase |
| RJ610_14280 | WND78475.1 | 2,67 | 3,26E-09 | | | S41 family peptidase |
| RJ610_14285 | WND78476.1 | 2,54 | 1,93E-08 | | | HAD family hydrolase |
| RJ610_14290 | WND78477.1 | 28,82 | 2,98E-06 | | | PAAR domain-containing protein |
| RJ610_14295 | WND78478.1 | 14,29 | 9,52E-19 | | | hypothetical protein |
| RJ610_14300 | WND78479.1 | 1,65 | 1,73E-02 | | | serine hydrolase domain-containing protein |
| RJ610_14305 | WND78480.1 | 0,96 | 8,89E-01 | | | hypothetical protein |
| RJ610_14310 | WND78481.1 | 2,61 | 3,25E-12 | | | DUF1684 domain-containing protein |
| RJ610_14315 | WND78482.1 | 1,19 | 2,27E-01 | | | hypothetical protein |
| RJ610_14320 | WND78483.1 | 1,00 | 9,90E-01 | | | VOC family protein |
| RJ610_14325 | WND78484.1 | 1,24 | 2,27E-01 | | | helix-turn-helix domain-containing protein |
| RJ610_14335 | WND78486.1 | 1,19 | 2,38E-01 | | | LysR family transcriptional regulator |
| RJ610_14340 | WND78487.1 | 0,56 | 5,55E-05 | | | hypothetical protein |
| RJ610_14345 | WND78488.1 | 0,85 | 6,70E-01 | | | hypothetical protein |
| RJ610_14350 | WND78489.1 | 1,33 | 2,43E-02 | | | plasmid replication/partition related protein |
| RJ610_14355 | WND78490.1 | 0,72 | 6,59E-03 | | | DUF333 domain-containing protein |
| RJ610_14360 | WND78491.1 | 1,24 | 8,76E-02 | | | NAD(P)/FAD-dependent oxidoreductase |
| RJ610_14365 | WND78492.1 | 1,41 | 2,63E-02 | | | Rrf2 family transcriptional regulator |
| RJ610_14370 | WND78493.1 | 8,01 | 1,18E-24 | | | lipase family protein |
| RJ610_14385 | WND78496.1 | 0,96 | 8,54E-01 | | | alpha/beta hydrolase |
| RJ610_14390 | WND78497.1 | 0,74 | 4,17E-02 | | | hypothetical protein |
| RJ610_14395 | WND78498.1 | 0,98 | 9,00E-01 | | | hypothetical protein |
| RJ610_14400 | WND78499.1 | 1,16 | 2,47E-01 | | | hypothetical protein |
| RJ610_14405 | WND78500.1 | 16,18 | 1,11E-93 | | | hypothetical protein |
| RJ610_14410 | WND78501.1 | 3,49 | 1,74E-11 | | | hypothetical protein |
| RJ610_14415 | WND78502.1 | 3,05 | 1,63E-06 | | | hypothetical protein |

| | | | | | | |
|---|---|---|---|---|---|---|
| RJ610_14420 | WND78503.1 | 6,14 | 1,00E-28 | | | tyrosinase family protein |
| RJ610_14425 | WND78504.1 | 3,22 | 4,86E-07 | | | hypothetical protein |
| RJ610_14430 | WND78505.1 | 1,41 | 3,56E-02 | | | amidohydrolase family protein |
| RJ610_14440 | WND78507.1 | 0,77 | 8,21E-02 | | | caspase family protein |
| RJ610_14445 | WND78508.1 | 0,79 | 2,33E-01 | | | hypothetical protein |
| RJ610_14450 | WND78509.1 | 1,03 | 9,17E-01 | | | YqaA family protein |
| RJ610_14460 | WND78511.1 | 1,87 | 8,03E-08 | | | SAM-dependent methyltransferase |
| RJ610_14465 | WND78512.1 | 2,73 | 8,67E-17 | | | hypothetical protein |
| RJ610_14470 | WND78513.1 | 2,89 | 3,28E-08 | | | nuclease-related domain-containing protein |
| RJ610_14475 | WND78514.1 | 3,29 | 1,52E-15 | | | hypothetical protein |
| RJ610_14485 | WND78516.1 | 2,72 | 1,08E-06 | | | hypothetical protein |
| RJ610_14490 | WND78517.1 | 1,46 | 3,05E-02 | | | SMI1/KNR4 family protein |
| RJ610_14500 | WND78519.1 | 1,32 | 1,80E-01 | | | hypothetical protein |
| RJ610_14510 | WND78521.1 | 1,72 | 1,43E-06 | | | hypothetical protein |
| RJ610_14515 | WND78522.1 | 1,83 | 2,19E-07 | | | hypothetical protein |
| RJ610_14520 | WND78523.1 | 1,48 | 3,51E-03 | | | hypothetical protein |
| RJ610_14525 | WND78524.1 | 1,09 | 5,60E-01 | | | hypothetical protein |
| RJ610_14530 | WND78525.1 | 1,16 | 2,75E-01 | | | hypothetical protein |
| RJ610_14535 | WND78526.1 | 0,87 | 2,93E-01 | | | hypothetical protein |
| RJ610_14540 | WND78527.1 | 0,91 | 6,52E-01 | | | hypothetical protein |
| RJ610_14545 | WND78528.1 | 1,01 | 9,83E-01 | | | hypothetical protein |
| RJ610_14550 | WND78529.1 | 1,27 | 3,57E-01 | | | ParA family protein |
| RJ610_14555 | WND78530.1 | 1,87 | 1,28E-05 | | | hypothetical protein |
| RJ610_14560 | WND78531.1 | 3,72 | 2,77E-21 | | | hypothetical protein |
| RJ610_14565 | WND78532.1 | 3,39 | 3,14E-13 | | | DUF3228 family protein |
| RJ610_14570 | WND78533.1 | 0,61 | 1,18E-06 | | | methylmalonyl-CoA mutase family protein |
| RJ610_14575 | WND78534.1 | 4,80 | 9,62E-07 | | | YXWGXW repeat-containing protein |
| RJ610_14585 | WND78536.1 | 2,40 | 7,00E-09 | | | carbonic anhydrase family protein |
| RJ610_14590 | WND78537.1 | 1,72 | 1,57E-05 | | | isovaleryl-CoA dehydrogenase |
| RJ610_14600 | WND78539.1 | 1,13 | 2,42E-01 | | | thiolase family protein |
| RJ610_14605 | WND78540.1 | 0,47 | 1,59E-07 | | | hypothetical protein |
| RJ610_14610 | WND78541.1 | 0,69 | 1,85E-02 | | | hypothetical protein |
| RJ610_14615 | WND78542.1 | 0,71 | 4,10E-04 | | | carboxyl transferase domain-containing protein |
| RJ610_14620 | WND78543.1 | 0,68 | 8,07E-05 | | | molecular chaperone HscC |
| RJ610_14625 | WND78544.1 | 0,77 | 3,64E-02 | | | hypothetical protein |
| RJ610_14630 | WND78545.1 | 0,77 | 9,99E-02 | | | J domain-containing protein |
| RJ610_14635 | WND78546.1 | 1,05 | 7,34E-01 | | | hypothetical protein |
| RJ610_14640 | WND78547.1 | 15,88 | 2,08E-99 | | | polymer-forming cytoskeletal protein |
| RJ610_14645 | WND78548.1 | 16,19 | 3,48E-163 | | | hypothetical protein |
| RJ610_14650 | WND78549.1 | 10,35 | 1,34E-56 | | | hypothetical protein |
| RJ610_14655 | WND78550.1 | 0,97 | 8,60E-01 | | | hypothetical protein |
| RJ610_14660 | WND78551.1 | 0,73 | 1,97E-03 | | | ferrous iron transporter B |
| RJ610_14665 | WND78552.1 | 0,90 | 4,79E-01 | | | FeoA family protein |

| | | | | | | |
|---|---|---|---|---|---|---|
| RJ610_14670 | WND78553.1 | 0,80 | 1,46E-01 | | | enoyl-CoA hydratase-related protein |
| RJ610_14675 | WND78554.1 | 0,75 | 7,31E-02 | | | VOC family protein |
| RJ610_14680 | WND78555.1 | 1,99 | 3,61E-10 | | | acetyl/propionyl/methylcrotonyl-CoA carboxylase subunit alpha |
| RJ610_14685 | WND78556.1 | 1,24 | 2,65E-01 | | | hypothetical protein |
| RJ610_14690 | WND78557.1 | 1,27 | 2,92E-01 | | | hypothetical protein |
| RJ610_14695 | WND78558.1 | 1,11 | 4,97E-01 | | | hypothetical protein |
| RJ610_14700 | WND78559.1 | 0,91 | 4,83E-01 | | | hydroxymethylglutaryl-CoA lyase |
| RJ610_14705 | WND78560.1 | 0,97 | 8,46E-01 | | | EAL domain-containing protein |
| RJ610_14710 | WND78561.1 | 1,54 | 5,93E-01 | | | hypothetical protein |
| RJ610_14715 | WND78562.1 | 1,82 | 2,70E-02 | | | hypothetical protein |
| RJ610_14720 | WND78563.1 | 0,88 | 4,93E-01 | | | hypothetical protein |
| RJ610_14725 | WND78564.1 | 1,20 | 5,57E-01 | | | hypothetical protein |
| RJ610_14730 | WND78565.1 | 1,23 | 1,30E-01 | | | DUF1801 domain-containing protein |
| RJ610_14735 | WND78566.1 | 1,28 | 1,74E-01 | | | DUF1801 domain-containing protein |
| RJ610_14740 | WND78567.1 | 1,57 | 5,37E-02 | | | hypothetical protein |
| RJ610_14745 | WND78568.1 | 0,82 | 5,50E-01 | | | thioredoxin family protein |
| RJ610_14750 | WND78569.1 | 0,89 | 4,86E-01 | | | PQQ-binding-like beta-propeller repeat protein |
| RJ610_14755 | WND78570.1 | 1,32 | 1,12E-01 | | | helix-turn-helix domain-containing protein |
| RJ610_14760 | WND78571.1 | 1,19 | 1,74E-01 | | | hypothetical protein |
| RJ610_14765 | WND78572.1 | 1,81 | 3,31E-06 | | | hypothetical protein |
| RJ610_14770 | WND78573.1 | 2,24 | 1,97E-10 | | | hypothetical protein |
| RJ610_14775 | WND78574.1 | 2,41 | 4,14E-09 | | | hypothetical protein |
| RJ610_14780 | WND78575.1 | 1,82 | 2,68E-06 | | | hydroxylase |
| RJ610_14785 | WND78576.1 | 1,97 | 2,39E-06 | | | hypothetical protein |
| RJ610_14790 | WND78577.1 | 2,80 | 9,85E-12 | | | hypothetical protein |
| RJ610_14795 | WND78578.1 | 6,15 | 4,13E-16 | | | hypothetical protein |
| RJ610_14800 | WND78579.1 | 1,33 | 1,53E-01 | | | 5-aminolevulinate synthase |
| RJ610_14805 | WND78580.1 | 1,81 | 1,70E-03 | | | TauD/TfdA family dioxygenase |
| RJ610_14810 | WND78581.1 | 1,22 | 4,99E-01 | | | 3-oxoacyl-[acyl-carrier-protein] synthase III C-terminal domain-containing protein |
| RJ610_14815 | WND78582.1 | 1,70 | 7,38E-03 | | | ATP-grasp domain-containing protein |
| RJ610_14820 | WND78583.1 | 1,54 | 1,73E-02 | | | hypothetical protein |
| RJ610_14825 | WND78584.1 | 1,77 | 1,20E-01 | | | MFS transporter |
| RJ610_14830 | WND78585.1 | 1,68 | 8,26E-02 | | | NADP-dependent oxidoreductase |
| RJ610_14835 | WND78586.1 | 1,17 | 4,87E-01 | | | CocE/NonD family hydrolase |
| RJ610_14840 | WND78587.1 | 2,25 | 7,19E-04 | | | DUF5916 domain-containing protein |
| RJ610_14845 | WND78588.1 | 7,63 | 2,91E-26 | | | GNAT family N-acetyltransferase |
| RJ610_14850 | WND78589.1 | 0,81 | 5,77E-01 | | | hypothetical protein |
| RJ610_14855 | WND78590.1 | 0,72 | 2,10E-01 | | | hypothetical protein |
| RJ610_14860 | WND78591.1 | 1,28 | 8,58E-02 | | | SDR family NAD(P)-dependent oxidoreductase |
| RJ610_14865 | WND78592.1 | 1,07 | 6,00E-01 | | | elongation factor P-like protein YeiP |
| RJ610_14870 | WND78593.1 | 1,27 | 1,58E-01 | | | hypothetical protein |
| RJ610_14875 | WND78594.1 | 1,75 | 8,34E-03 | | | GNAT family N-acetyltransferase |

| | | | | | | |
|---|---|---|---|---|---|---|
| RJ610_14885 | WND78596.1 | 0,80 | 5,91E-02 | | | FAD-dependent oxidoreductase |
| RJ610_14890 | WND78597.1 | 1,26 | 9,32E-02 | | | hypothetical protein |
| RJ610_14895 | WND78598.1 | 0,93 | 6,51E-01 | | | hypothetical protein |
| RJ610_14900 | WND78599.1 | 1,07 | 6,99E-01 | | | NUDIX hydrolase |
| RJ610_14905 | WND78600.1 | 1,17 | 1,07E-01 | | | amino acid permease |
| RJ610_14910 | WND78601.1 | 1,39 | 1,10E-02 | | | amino acid permease |
| RJ610_14915 | WND78602.1 | 0,78 | 5,54E-03 | | | methylthioribulose 1-phosphate dehydratase |
| RJ610_14920 | WND78603.1 | 0,79 | 1,98E-02 | | | acireductone dioxygenase |
| RJ610_14925 | WND78604.1 | 0,98 | 9,26E-01 | | | hypothetical protein |
| RJ610_14930 | WND78605.1 | 0,70 | 5,44E-03 | | | acireductone synthase |
| RJ610_14935 | WND78606.1 | 0,67 | 1,80E-01 | | | hypothetical protein |
| RJ610_14940 | WND78607.1 | 0,73 | 2,34E-01 | | | type II 3-dehydroquinate dehydratase |
| RJ610_14945 | WND78608.1 | 1,11 | 6,15E-01 | | | YdeI/OmpD-associated family protein |
| RJ610_14950 | WND78609.1 | 0,82 | 3,02E-01 | | | calcineurin-like phosphoesterase family protein |
| RJ610_14955 | WND83259.1 | 0,68 | 6,68E-01 | | | bifunctional phosphoribosyl-AMP cyclohydrolase/phosphoribosyl-ATP diphosphatase HisIE |
| RJ610_14960 | WND78610.1 | 0,64 | 6,02E-01 | | | imidazole glycerol phosphate synthase subunit HisF |
| RJ610_14965 | WND78611.1 | 0,64 | 6,17E-01 | | | 1-(5-phosphoribosyl)-5-[(5-phosphoribosylamino)methylideneamino]imidazole-4-carboxamide isomerase |
| RJ610_14970 | WND78612.1 | 0,69 | 6,98E-01 | | | imidazole glycerol phosphate synthase subunit HisH |
| RJ610_14975 | WND78613.1 | 0,65 | 6,26E-01 | | | histidinol-phosphatase |
| RJ610_14980 | WND78614.1 | 0,55 | 4,65E-01 | | | histidinol-phosphate transaminase |
| RJ610_14985 | WND83260.1 | 0,58 | 5,78E-01 | | | histidinol dehydrogenase |
| RJ610_14990 | WND78615.1 | 0,44 | 3,58E-01 | | | ATP phosphoribosyltransferase |
| RJ610_14995 | WND78616.1 | 0,40 | 2,47E-01 | | | YerC/YecD family TrpR-related protein |
| RJ610_15000 | WND78617.1 | 0,78 | 3,92E-01 | | | 7TM diverse intracellular signaling domain-containing protein |
| RJ610_15005 | WND78618.1 | 0,74 | 1,00E-01 | | | 2OG-Fe(II) oxygenase |
| RJ610_15015 | WND78620.1 | 0,82 | 6,81E-02 | | | histidine--tRNA ligase |
| RJ610_15020 | | 0,86 | 8,06E-01 | | | tRNA-Pro |
| RJ610_15025 | WND78621.1 | 1,53 | 3,76E-03 | | | hypothetical protein |
| RJ610_15030 | WND78622.1 | 2,92 | 3,35E-11 | | | polysaccharide deacetylase family protein |
| RJ610_15035 | WND78623.1 | 33,92 | 2,26E-56 | | | glycosyltransferase family 2 protein |
| RJ610_15040 | WND78624.1 | 88,35 | 4,86E-84 | | | methyltransferase domain-containing protein |
| RJ610_15045 | WND78625.1 | 79,65 | 2,08E-65 | | | MFS transporter |
| RJ610_15050 | WND78626.1 | 19,25 | 1,28E-44 | | | hypothetical protein |
| RJ610_15055 | WND78627.1 | 1,69 | 1,23E-06 | | | GH92 family glycosyl hydrolase |
| RJ610_15060 | WND78628.1 | 0,03 | 8,09E-138 | | | poly-beta-1,6 N-acetyl-D-glucosamine export porin PgaA |
| RJ610_15065 | WND83261.1 | 0,01 | 4,58E-117 | | | poly-beta-1,6-N-acetyl-D-glucosamine N-deacetylase PgaB |
| RJ610_15070 | WND78629.1 | 0,01 | 2,11E-137 | | | poly-beta-1,6-N-acetyl-D-glucosamine synthase |
| RJ610_15075 | WND78630.1 | 0,05 | 2,68E-93 | | | poly-beta-1,6-N-acetyl-D-glucosamine biosynthesis protein PgaD |
| RJ610_15080 | WND78631.1 | 0,51 | 1,45E-04 | | | CocE/NonD family hydrolase |
| RJ610_15085 | WND78632.1 | 3,67 | 2,04E-11 | | | hypothetical protein |

| | | | | | | |
|---|---|---|---|---|---|---|
| RJ610_15090 | WND78633.1 | 2,78 | 1,22E-04 | | | hypothetical protein |
| RJ610_15095 | WND78634.1 | 3,73 | 2,21E-05 | | | hypothetical protein |
| RJ610_15100 | WND78635.1 | 2,53 | 4,76E-03 | | | N-acetylmuramidase family protein |
| RJ610_15105 | WND78636.1 | 2,26 | 5,08E-04 | | | hypothetical protein |
| RJ610_15110 | WND78637.1 | 2,96 | 4,46E-06 | | | hypothetical protein |
| RJ610_15115 | WND78638.1 | 4,38 | 3,78E-13 | | | glutamate-5-semialdehyde dehydrogenase |
| RJ610_15120 | WND78639.1 | 3,98 | 7,72E-11 | | | glutamate 5-kinase |
| RJ610_15125 | WND78640.1 | 3,94 | 1,70E-12 | | | argininosuccinate lyase |
| RJ610_15130 | WND78641.1 | 3,70 | 1,93E-13 | | | proline--tRNA ligase |
| RJ610_15135 | WND78642.1 | 3,77 | 3,36E-11 | | | N-acetyl-gamma-glutamyl-phosphate reductase |
| RJ610_15140 | WND78643.1 | 4,47 | 1,55E-13 | | | GNAT family N-acetyltransferase |
| RJ610_15145 | WND78644.1 | 4,42 | 2,18E-13 | | | acetylglutamate kinase |
| RJ610_15155 | WND78646.1 | 3,64 | 9,22E-10 | | | argininosuccinate synthase |
| RJ610_15165 | WND78648.1 | 1,08 | 6,10E-01 | | | hypothetical protein |
| RJ610_15170 | WND78649.1 | 1,06 | 5,60E-01 | | | cysteine--tRNA ligase |
| RJ610_15175 | WND78650.1 | 1,24 | 6,60E-02 | | | SufE family protein |
| RJ610_15180 | WND83262.1 | 0,83 | 2,25E-01 | | | MFS transporter |
| RJ610_15185 | WND78651.1 | 0,83 | 1,73E-01 | | | hypothetical protein |
| RJ610_15190 | WND78652.1 | 0,91 | 5,04E-01 | | | Imm30 family immunity protein |
| RJ610_15195 | WND78653.1 | 0,83 | 6,80E-02 | | | RNA polymerase-binding protein DksA |
| RJ610_15200 | WND78654.1 | 1,07 | 5,51E-01 | | | membrane protein insertion efficiency factor YidD |
| RJ610_15210 | WND78656.1 | 1,35 | 4,25E-03 | | | M23 family metallopeptidase |
| RJ610_15215 | WND78657.1 | 5,99 | 7,83E-34 | | | hypothetical protein |
| RJ610_15220 | WND78658.1 | 9,80 | 1,06E-109 | | | diguanylate cyclase |
| RJ610_15230 | WND78660.1 | 1,53 | 9,99E-04 | | | phytoene/squalene synthase family protein |
| RJ610_15235 | WND78661.1 | 1,38 | 1,90E-02 | | | phosphoglycolate phosphatase |
| RJ610_15240 | WND78662.1 | 1,29 | 1,63E-02 | | | bifunctional 2-polyprenyl-6-hydroxyphenol methylase/3-demethylubiquinol 3-O-methyltransferase UbiG |
| RJ610_15250 | WND78664.1 | 1,02 | 8,57E-01 | | | elongation factor P |
| RJ610_15255 | WND78665.1 | 0,98 | 8,67E-01 | | | EF-P beta-lysylation protein EpmB |
| RJ610_15260 | WND78666.1 | 1,99 | 1,37E-15 | | | EAL domain-containing protein |
| RJ610_15265 | WND78667.1 | 1,47 | 6,13E-02 | | | protease HtpX |
| RJ610_15270 | WND78668.1 | 0,95 | 7,47E-01 | | | tRNA glutamyl-Q(34) synthetase GluQRS |
| RJ610_15275 | WND78669.1 | 1,63 | 2,39E-06 | | | acetoacetyl-CoA reductase |
| RJ610_15280 | WND78670.1 | 0,76 | 1,37E-02 | | | polyhydroxyalkanoate synthesis repressor PhaR |
| RJ610_15285 | WND78671.1 | 0,55 | 1,04E-05 | | | acetoacetyl-CoA reductase |
| RJ610_15290 | WND78672.1 | 0,66 | 9,11E-04 | | | M48 family metallopeptidase |
| RJ610_15295 | WND78673.1 | 0,62 | 1,01E-04 | | | formylglycine-generating enzyme family protein |
| RJ610_15300 | WND78674.1 | 0,59 | 1,79E-06 | | | ribonuclease D |
| RJ610_15305 | | 1,07 | 8,57E-01 | | | tRNA-Ala |
| RJ610_15310 | WND78675.1 | 2,04 | 1,78E-04 | | | hypothetical protein |
| RJ610_15315 | WND78676.1 | 3,13 | 2,40E-17 | | | hypothetical protein |
| RJ610_15320 | WND78677.1 | 5,77 | 5,22E-37 | | | dihydroxyacetone kinase subunit DhaK |

| | | | | | | |
|---|---|---|---|---|---|---|
| RJ610_15325 | WND78678.1 | 1,62 | 2,42E-02 | | | hypothetical protein |
| RJ610_15330 | WND78679.1 | 2,27 | 9,72E-06 | | | hypothetical protein |
| RJ610_15335 | WND78680.1 | 2,85 | 8,30E-08 | | | DUF4189 domain-containing protein |
| RJ610_15340 | WND78681.1 | 2,79 | 3,72E-06 | | | DUF4189 domain-containing protein |
| RJ610_15345 | WND78682.1 | 2,01 | 3,64E-03 | | | hypothetical protein |
| RJ610_15350 | WND78683.1 | 1,22 | 5,20E-01 | | | ATP-binding protein |
| RJ610_15355 | WND78684.1 | 0,77 | 4,29E-01 | | | winged helix-turn-helix domain-containing protein |
| RJ610_15360 | | 1,54 | 1,11E-01 | | | tRNA-Leu |
| RJ610_15365 | WND78685.1 | 1,85 | 1,24E-04 | | | anti-sigma factor |
| RJ610_15370 | WND78686.1 | 2,15 | 1,20E-05 | | | sigma-70 family RNA polymerase sigma factor |
| RJ610_15375 | WND78687.1 | 0,72 | 8,01E-03 | | | nucleoside diphosphate kinase regulator |
| RJ610_15380 | WND78688.1 | 0,26 | 1,03E-14 | | | heavy metal sensor histidine kinase |
| RJ610_15385 | WND78689.1 | 0,20 | 8,96E-18 | | | heavy metal response regulator transcription factor |
| RJ610_15390 | WND78690.1 | 0,62 | 4,88E-04 | | | glutaminyl-peptide cyclotransferase |
| RJ610_15395 | WND78691.1 | 0,55 | 9,82E-09 | | | DNA topoisomerase IV subunit A |
| RJ610_15400 | WND78692.1 | 1,05 | 8,38E-01 | | | nucleotide pyrophosphohydrolase |
| RJ610_15405 | WND78693.1 | 0,91 | 4,84E-01 | | | TonB family protein |
| RJ610_15410 | WND78694.1 | 1,27 | 1,53E-01 | | | DJ-1/PfpI family protein |
| RJ610_15415 | WND78695.1 | 1,20 | 4,87E-01 | | | GlxA family transcriptional regulator |
| RJ610_15420 | WND78696.1 | 1,64 | 7,53E-02 | | | hypothetical protein |
| RJ610_15425 | WND78697.1 | 0,33 | 1,03E-22 | | | autotransporter domain-containing protein |
| RJ610_15430 | WND78698.1 | 0,59 | 4,69E-05 | | | hypothetical protein |
| RJ610_15435 | | 1,22 | 3,97E-01 | | | tRNA-Arg |
| RJ610_15445 | WND78700.1 | 1,27 | 5,29E-02 | | | hypothetical protein |
| RJ610_15450 | WND78701.1 | 1,07 | 7,03E-01 | | | hypothetical protein |
| RJ610_15455 | WND78702.1 | 1,45 | 1,46E-01 | | | hypothetical protein |
| RJ610_15460 | WND78703.1 | 11,09 | 4,36E-20 | | | hypothetical protein |
| RJ610_15465 | WND78704.1 | 5,40 | 6,04E-20 | | | hypothetical protein |
| RJ610_15470 | WND78705.1 | 1,04 | 8,46E-01 | | | DUF4190 domain-containing protein |
| RJ610_15475 | WND78706.1 | 1,52 | 6,75E-03 | | | hypothetical protein |
| RJ610_15480 | WND78707.1 | 2,41 | 3,75E-10 | | | hypothetical protein |
| RJ610_15485 | WND78708.1 | 17,27 | 1,48E-86 | | | hypothetical protein |
| RJ610_15490 | WND78709.1 | 0,79 | 7,52E-01 | | | hypothetical protein |
| RJ610_15495 | WND78710.1 | 1,62 | 1,24E-07 | | | quinone-dependent dihydroorotate dehydrogenase |
| RJ610_15500 | WND78711.1 | 1,34 | 2,71E-02 | | | UDP-N-acetylmuramate dehydrogenase |
| RJ610_15505 | WND83263.1 | 1,36 | 8,65E-02 | | | DMT family transporter |
| RJ610_15515 | WND78713.1 | 8,87 | 4,74E-31 | | | hypothetical protein |
| RJ610_15520 | WND78714.1 | 1,18 | 1,21E-01 | | | sensor domain-containing diguanylate cyclase |
| RJ610_15525 | WND78715.1 | 0,64 | 3,88E-02 | | | hypothetical protein |
| RJ610_15535 | WND78717.1 | 0,24 | 6,18E-30 | | | YadA-like family protein |
| RJ610_15545 | WND83264.1 | 0,94 | 7,28E-01 | | | phosphatase PAP2 family protein |
| RJ610_15550 | WND78719.1 | 0,78 | 2,16E-02 | | | flavodoxin-dependent (E)-4-hydroxy-3-methylbut-2-enyl-diphosphate synthase |

| | | | | | | |
|---|---|---|---|---|---|---|
| RJ610_15555 | WND78720.1 | 0,80 | 1,40E-01 | | | exodeoxyribonuclease VII large subunit |
| RJ610_15560 | WND78721.1 | 1,29 | 3,93E-02 | | | acetolactate synthase large subunit |
| RJ610_15565 | WND78722.1 | 1,56 | 7,27E-03 | | | aldehyde dehydrogenase family protein |
| RJ610_15570 | WND78723.1 | 1,01 | 9,58E-01 | | | VWA domain-containing protein |
| RJ610_15575 | WND78724.1 | 1,22 | 6,55E-02 | | | EAL domain-containing protein |
| RJ610_15580 | WND83265.1 | 1,42 | 1,82E-02 | | | RNA polymerase sigma factor |
| RJ610_15585 | WND78725.1 | 0,79 | 1,58E-01 | | | hypothetical protein |
| RJ610_15590 | WND78726.1 | 1,03 | 8,83E-01 | | | YigZ family protein |
| RJ610_15595 | WND78727.1 | 2,02 | 1,32E-03 | | | ABC transporter transmembrane domain-containing protein |
| RJ610_15600 | WND78728.1 | 0,80 | 1,87E-01 | | | hypothetical protein |
| RJ610_15615 | WND78731.1 | 1,61 | 3,97E-02 | | | DUF4279 domain-containing protein |
| RJ610_15620 | WND78732.1 | 2,10 | 2,36E-04 | | | DUF4279 domain-containing protein |
| RJ610_15625 | WND78733.1 | 1,59 | 1,14E-03 | | | hypothetical protein |
| RJ610_15630 | WND83266.1 | 0,44 | 3,87E-05 | | | hypothetical protein |
| RJ610_15635 | WND78734.1 | 0,22 | 2,54E-20 | | | YfbM family protein |
| RJ610_15640 | WND78735.1 | 0,18 | 1,54E-37 | | | hypothetical protein |
| RJ610_15645 | WND78736.1 | 0,72 | 7,07E-02 | | | L,D-transpeptidase family protein |
| RJ610_15650 | WND78737.1 | 0,92 | 5,05E-01 | | | acyl-CoA dehydrogenase family protein |
| RJ610_15655 | WND78738.1 | 1,14 | 5,17E-01 | | | cold-shock protein |
| RJ610_15660 | WND78739.1 | 0,92 | 4,85E-01 | | | S-methyl-5'-thioinosine phosphorylase |
| RJ610_15665 | WND78740.1 | 0,90 | 4,23E-01 | | | hypoxanthine-guanine phosphoribosyltransferase |
| RJ610_15670 | WND78741.1 | 0,87 | 2,74E-01 | | | beta-N-acetylhexosaminidase |
| RJ610_15675 | WND78742.1 | 1,32 | 5,93E-02 | | | CYTH domain-containing protein |
| RJ610_15680 | WND78743.1 | 2,68 | 6,05E-04 | | | hypothetical protein |
| RJ610_15685 | WND83267.1 | 0,90 | 5,77E-01 | | | 23S rRNA (uracil(1939)-C(5))-methyltransferase RlmD |
| RJ610_15690 | WND78744.1 | 0,61 | 5,80E-05 | | | DUF1318 domain-containing protein |
| RJ610_15695 | WND78745.1 | 0,51 | 3,84E-07 | | | hypothetical protein |
| RJ610_15700 | WND78746.1 | 0,62 | 3,54E-05 | | | response regulator |
| RJ610_15705 | WND78747.1 | 0,95 | 7,80E-01 | | | DNA repair protein RecO |
| RJ610_15710 | WND78748.1 | 1,30 | 6,53E-02 | | | hypothetical protein |
| RJ610_15715 | WND78749.1 | 1,05 | 7,14E-01 | | | GTPase Era |
| RJ610_15720 | WND78750.1 | 2,95 | 4,18E-18 | | | ribonuclease III |
| RJ610_15725 | WND78751.1 | 3,04 | 2,30E-19 | | | DUF4845 domain-containing protein |
| RJ610_15730 | WND78752.1 | 1,88 | 6,31E-10 | | | signal peptidase I |
| RJ610_15735 | WND78753.1 | 1,51 | 1,44E-04 | | | translation elongation factor 4 |
| RJ610_15745 | WND78755.1 | 3,20 | 1,99E-18 | | | sigma-E factor negative regulatory protein |
| RJ610_15750 | WND78756.1 | 3,05 | 1,00E-13 | | | RNA polymerase sigma factor RpoE |
| RJ610_15755 | WND78757.1 | 1,47 | 1,20E-03 | | | 3-hydroxyacyl-CoA dehydrogenase NAD-binding domain-containing protein |
| RJ610_15760 | WND78758.1 | 1,08 | 6,77E-01 | | | hypothetical protein |
| RJ610_15765 | WND78759.1 | 0,99 | 9,58E-01 | | | PilZ domain-containing protein |
| RJ610_15770 | WND78760.1 | 0,73 | 4,18E-03 | | | DNA polymerase III subunit delta' |
| RJ610_15775 | WND78761.1 | 0,73 | 3,68E-03 | | | dTMP kinase |

| | | | | | | |
|---|---|---|---|---|---|---|
| RJ610_15780 | WND78762.1 | 0,76 | 1,19E-02 | | | endolytic transglycosylase MltG |
| RJ610_15785 | WND78763.1 | 0,86 | 3,31E-01 | | | aminodeoxychorismate lyase |
| RJ610_15795 | WND78765.1 | 0,97 | 8,45E-01 | | | acyl carrier protein |
| RJ610_15800 | WND78766.1 | 1,20 | 2,08E-01 | | | 3-oxoacyl-ACP reductase FabG |
| RJ610_15805 | WND78767.1 | 8,56 | 2,48E-23 | | | SDR family oxidoreductase |
| RJ610_15810 | WND78768.1 | 1,24 | 1,58E-01 | | | ACP S-malonyltransferase |
| RJ610_15815 | WND78769.1 | 0,85 | 1,15E-01 | | | beta-ketoacyl-ACP synthase III |
| RJ610_15820 | WND78770.1 | 0,76 | 7,05E-02 | | | 50S ribosomal protein L32 |
| RJ610_15825 | WND78771.1 | 0,89 | 3,28E-01 | | | YceD family protein |
| RJ610_15830 | WND78772.1 | 0,70 | 4,75E-02 | | | hypothetical protein |
| RJ610_15835 | WND78773.1 | 1,15 | 3,21E-01 | | | Maf family nucleotide pyrophosphatase |
| RJ610_15840 | WND78774.1 | 0,91 | 4,59E-01 | | | glycosyltransferase family 39 protein |
| RJ610_15845 | WND83268.1 | 2,10 | 4,97E-13 | | | AAA family ATPase |
| RJ610_15850 | WND83269.1 | 1,18 | 2,09E-01 | | | DUF58 domain-containing protein |
| RJ610_15855 | WND78775.1 | 0,56 | 2,52E-06 | | | hypothetical protein |
| RJ610_15860 | WND78776.1 | 0,81 | 3,99E-02 | | | M91 family zinc metallopeptidase |
| RJ610_15865 | WND83270.1 | 1,69 | 6,58E-08 | | | DUF3488 and transglutaminase-like domain-containing protein |
| RJ610_15875 | WND78778.1 | 1,55 | 8,50E-04 | | | histidine triad nucleotide-binding protein |
| RJ610_15880 | WND78779.1 | 1,04 | 8,57E-01 | | | recombination mediator RecR |
| RJ610_15885 | WND78780.1 | 0,95 | 8,05E-01 | | | hypothetical protein |
| RJ610_15890 | WND78781.1 | 1,01 | 9,70E-01 | | | YbaB/EbfC family nucleoid-associated protein |
| RJ610_15895 | WND78782.1 | 0,99 | 9,27E-01 | | | DNA polymerase III subunit gamma/tau |
| RJ610_15900 | | 0,64 | 4,19E-01 | | | tRNA-Ser |
| RJ610_15905 | WND78783.1 | 3,90 | 3,29E-13 | | | hypothetical protein |
| RJ610_15910 | WND78784.1 | 4,15 | 1,39E-27 | | | RHS repeat-associated core domain-containing protein |
| RJ610_15915 | WND78785.1 | 3,57 | 8,34E-12 | | | hypothetical protein |
| RJ610_15920 | WND78786.1 | 5,58 | 3,16E-23 | | | hypothetical protein |
| RJ610_15925 | WND78787.1 | 0,86 | 3,17E-01 | | | energy transducer TonB |
| RJ610_15930 | WND78788.1 | 0,85 | 1,77E-01 | | | metallophosphoesterase family protein |
| RJ610_15935 | | 0,86 | 4,92E-01 | | | signal recognition particle sRNA small type |
| RJ610_15945 | WND78790.1 | 1,09 | 7,88E-01 | | | hypothetical protein |
| RJ610_15950 | WND78791.1 | 1,09 | 5,90E-01 | | | molybdenum cofactor biosynthesis protein MoaE |
| RJ610_15955 | WND78792.1 | 0,92 | 6,10E-01 | | | MoaD/ThiS family protein |
| RJ610_15960 | | 0,84 | 2,43E-01 | | | cyclic pyranopterin monophosphate synthase MoaC |
| RJ610_15965 | WND78793.1 | 1,08 | 5,72E-01 | | | phytanoyl-CoA dioxygenase family protein |
| RJ610_15970 | WND78794.1 | 1,11 | 3,96E-01 | | | GTP 3',8-cyclase MoaA |
| RJ610_15975 | WND78795.1 | 0,81 | 1,15E-01 | | | MBL fold metallo-hydrolase |
| RJ610_15980 | WND78796.1 | 0,73 | 2,23E-03 | | | 3-deoxy-D-manno-octulosonic acid kinase |
| RJ610_15985 | WND78797.1 | 0,58 | 1,31E-05 | | | glycosyltransferase family 9 protein |
| RJ610_15990 | WND78798.1 | 0,61 | 1,59E-03 | | | NADAR family protein |
| RJ610_15995 | WND78799.1 | 0,81 | 1,14E-01 | | | hypothetical protein |
| RJ610_16000 | WND78800.1 | 0,84 | 1,51E-01 | | | DUF6165 family protein |
| RJ610_16005 | | 1,14 | 5,81E-01 | | | tRNA-Ser |

| | | | | | | |
|---|---|---|---|---|---|---|
| RJ610_16010 | WND78801.1 | 0,52 | 9,38E-10 | | | protein phosphatase 2C domain-containing protein |
| RJ610_16015 | WND78802.1 | 1,17 | 1,45E-01 | | | DNA polymerase III subunit epsilon |
| RJ610_16020 | WND78803.1 | 0,78 | 2,43E-02 | | | ribonuclease HI |
| RJ610_16025 | WND78804.1 | 1,29 | 6,81E-02 | | | methyltransferase domain-containing protein |
| RJ610_16030 | WND78805.1 | 1,07 | 6,25E-01 | | | hydroxyacylglutathione hydrolase |
| RJ610_16035 | WND78806.1 | 1,02 | 9,04E-01 | | | transglycosylase SLT domain-containing protein |
| RJ610_16040 | WND78807.1 | 1,12 | 5,21E-01 | | | Na+/H+ antiporter NhaC family protein |
| RJ610_16045 | WND78808.1 | 1,30 | 2,71E-02 | | | isocitrate dehydrogenase |
| RJ610_16050 | WND83271.1 | 0,87 | 4,34E-01 | | | carboxymuconolactone decarboxylase family protein |
| RJ610_16055 | WND78809.1 | 0,98 | 9,17E-01 | | | glutaredoxin 3 |
| RJ610_16060 | WND78810.1 | 2,18 | 8,08E-09 | | | M48 family metalloprotease |
| RJ610_16065 | WND78811.1 | 0,65 | 8,75E-03 | | | phosphate regulon transcriptional regulator PhoB |
| RJ610_16070 | WND78812.1 | 0,97 | 8,01E-01 | | | phosphate regulon sensor histidine kinase PhoR |
| RJ610_16075 | WND78813.1 | 0,89 | 3,67E-01 | | | polyphosphate kinase 1 |
| RJ610_16080 | WND78814.1 | 0,94 | 6,32E-01 | | | exopolyphosphatase |
| RJ610_16085 | WND78815.1 | 1,56 | 4,49E-05 | | | AMP-binding protein |
| RJ610_16090 | WND78816.1 | 0,67 | 9,52E-03 | | | hypothetical protein |
| RJ610_16105 | WND78819.1 | 1,38 | 1,26E-03 | | | glucans biosynthesis glucosyltransferase MdoH |
| RJ610_16110 | WND78820.1 | 2,25 | 1,62E-11 | | | glucan biosynthesis protein G |
| RJ610_16115 | WND78821.1 | 2,25 | 8,08E-02 | | | glycosyltransferase family 1 protein |
| RJ610_16120 | WND78822.1 | 3,04 | 1,95E-03 | | | phosphatase PAP2 family protein |
| RJ610_16125 | WND83272.1 | 0,48 | 6,35E-04 | | | carbon-nitrogen hydrolase family protein |
| RJ610_16130 | WND78823.1 | 0,53 | 1,65E-02 | | | GNAT family N-acetyltransferase |
| RJ610_16135 | WND78824.1 | 0,66 | 2,05E-02 | | | cell envelope integrity protein CreD |
| RJ610_16140 | WND78825.1 | 0,52 | 2,29E-01 | | | hypothetical protein |
| RJ610_16145 | WND78826.1 | 0,63 | 9,72E-04 | | | two-component system sensor histidine kinase CreC |
| RJ610_16150 | WND78827.1 | 0,65 | 1,10E-03 | | | two-component system response regulator CreB |
| RJ610_16155 | WND78828.1 | 0,73 | 6,68E-02 | | | 23S rRNA (adenine(2030)-N(6))-methyltransferase RlmJ |
| RJ610_16160 | WND78829.1 | 1,08 | 7,75E-01 | | | hypothetical protein |
| RJ610_16165 | WND78830.1 | 0,82 | 5,22E-01 | | | hypothetical protein |
| RJ610_16170 | WND78831.1 | 0,80 | 1,15E-01 | | | GNAT family N-acetyltransferase |
| RJ610_16175 | WND78832.1 | 0,69 | 7,86E-04 | | | DUF1963 domain-containing protein |
| RJ610_16180 | WND78833.1 | 0,49 | 8,81E-14 | | | cation diffusion facilitator family transporter |
| RJ610_16185 | WND78834.1 | 0,33 | 2,63E-28 | | | GNAT family N-acetyltransferase |
| RJ610_16190 | WND78835.1 | 0,44 | 2,39E-12 | | | hypothetical protein |
| RJ610_16195 | WND78836.1 | 1,07 | 6,93E-01 | | | hypothetical protein |
| RJ610_16200 | WND78837.1 | 1,39 | 6,74E-02 | | | transcription-repair coupling factor |
| RJ610_16205 | WND78838.1 | 0,91 | 6,90E-01 | | | heme-binding protein |
| RJ610_16210 | WND78839.1 | 0,67 | 6,40E-03 | | | phosphoglycerate mutase family protein |
| RJ610_16220 | WND78841.1 | 1,34 | 2,44E-03 | | | pirin family protein |
| RJ610_16225 | WND78842.1 | 0,20 | 1,60E-46 | | | hypothetical protein |
| RJ610_16230 | WND78843.1 | 0,69 | 2,62E-03 | | | aquaporin Z |
| RJ610_16235 | WND78844.1 | 0,94 | 8,40E-01 | | | hypothetical protein |

| | | | | | | |
|---|---|---|---|---|---|---|
| RJ610_16240 | WND78845.1 | 1,03 | 8,90E-01 | | | pirin family protein |
| RJ610_16245 | WND78846.1 | 0,89 | 5,28E-01 | | | methyltransferase domain-containing protein |
| RJ610_16250 | WND78847.1 | 0,92 | 4,75E-01 | | | bifunctional 23S rRNA (guanine(2069)-N(7))-methyltransferase RlmK/23S rRNA (guanine(2445)-N(2))-methyltransferase RlmL |
| RJ610_16255 | WND83273.1 | 0,92 | 5,39E-01 | | | hypothetical protein |
| RJ610_16260 | WND78848.1 | 1,01 | 9,34E-01 | | | MOSC domain-containing protein |
| RJ610_16265 | WND78849.1 | 0,75 | 1,55E-02 | | | N-acetylmuramoyl-L-alanine amidase |
| RJ610_16270 | WND78850.1 | 1,04 | 8,04E-01 | | | hypothetical protein |
| RJ610_16275 | WND78851.1 | 0,74 | 4,16E-03 | | | acyl-CoA thioesterase II |
| RJ610_16280 | WND78852.1 | 0,82 | 1,05E-01 | | | hypothetical protein |
| RJ610_16285 | WND78853.1 | 1,33 | 3,33E-02 | | | hypothetical protein |
| RJ610_16290 | WND78854.1 | 0,71 | 3,98E-02 | | | sigma-70 family RNA polymerase sigma factor |
| RJ610_16295 | WND78855.1 | 0,75 | 2,94E-01 | | | hypothetical protein |
| RJ610_16300 | WND78856.1 | 0,44 | 1,37E-03 | | | EF-hand domain-containing protein |
| RJ610_16305 | WND78857.1 | 1,25 | 6,53E-02 | | | arginyltransferase |
| RJ610_16310 | WND78858.1 | 0,40 | 3,71E-09 | | | hypothetical protein |
| RJ610_16315 | WND78859.1 | 0,93 | 7,09E-01 | | | endonuclease/exonuclease/phosphatase family protein |
| RJ610_16320 | WND78860.1 | 4,40 | 5,01E-14 | | | hypothetical protein |
| RJ610_16325 | WND78861.1 | 1,63 | 1,45E-03 | | | ATP-grasp domain-containing protein |
| RJ610_16330 | WND78862.1 | 1,51 | 1,83E-02 | | | SMI1/KNR4 family protein |
| RJ610_16335 | WND78863.1 | 0,77 | 9,10E-02 | | | ADP-ribosylation/crystallin J1 |
| RJ610_16340 | WND78864.1 | 0,73 | 1,02E-02 | | | hypothetical protein |
| RJ610_16345 | WND78865.1 | 0,88 | 3,05E-01 | | | formate-dependent phosphoribosylglycinamide formyltransferase |
| RJ610_16350 | WND78866.1 | 0,71 | 1,24E-02 | | | hypothetical protein |
| RJ610_16355 | WND78867.1 | 0,56 | 5,28E-10 | | | SPFH domain-containing protein |
| RJ610_16360 | WND78868.1 | 0,52 | 9,26E-10 | | | hypothetical protein |
| RJ610_16365 | WND78869.1 | 0,65 | 3,75E-04 | | | Arc family DNA binding domain-containing protein |
| RJ610_16370 | WND78870.1 | 0,52 | 1,45E-06 | | | hypothetical protein |
| RJ610_16375 | WND78871.1 | 0,63 | 5,14E-06 | | | D-alanyl-D-alanine carboxypeptidase family protein |
| RJ610_16380 | WND78872.1 | 0,64 | 1,52E-03 | | | hypothetical protein |
| RJ610_16385 | WND78873.1 | 0,73 | 8,49E-03 | | | class I SAM-dependent methyltransferase |
| RJ610_16390 | WND78874.1 | 1,29 | 9,48E-02 | | | DNA-3-methyladenine glycosylase |
| RJ610_16395 | WND78875.1 | 1,15 | 3,99E-01 | | | DUF423 domain-containing protein |
| RJ610_16405 | WND78877.1 | 0,40 | 4,49E-14 | | | response regulator transcription factor |
| RJ610_16410 | WND78878.1 | 0,39 | 2,83E-09 | | | histidine kinase |
| RJ610_16415 | WND78879.1 | 0,65 | 1,01E-06 | | | GNAT family N-acetyltransferase |
| RJ610_16420 | WND78880.1 | 0,61 | 1,47E-06 | | | septum site-determining protein MinC |
| RJ610_16425 | WND78881.1 | 0,65 | 1,89E-05 | | | septum site-determining protein MinD |
| RJ610_16430 | WND78882.1 | 0,70 | 4,16E-03 | | | cell division topological specificity factor MinE |
| RJ610_16435 | WND78883.1 | 0,68 | 5,12E-03 | | | hypothetical protein |
| RJ610_16440 | WND83274.1 | 0,72 | 9,90E-03 | | | SDR family oxidoreductase |
| RJ610_16445 | WND78884.1 | 0,74 | 1,66E-01 | | | hypothetical protein |
| RJ610_16450 | WND78885.1 | 1,18 | 1,82E-01 | | | M2 family metallopeptidase |

| | | | | | | |
|---|---|---|---|---|---|---|
| RJ610_16455 | WND78886.1 | 2,83 | 9,76E-05 | | | DUF2235 domain-containing protein |
| RJ610_16460 | WND78887.1 | 1,09 | 6,83E-01 | | | amidohydrolase family protein |
| RJ610_16465 | WND78888.1 | 1,18 | 3,50E-01 | | | helix-turn-helix transcriptional regulator |
| RJ610_16470 | WND78889.1 | 1,05 | 8,45E-01 | | | DMT family transporter |
| RJ610_16475 | WND78890.1 | 1,57 | 5,72E-05 | | | winged helix-turn-helix domain-containing protein |
| RJ610_16480 | WND78891.1 | 0,80 | 7,00E-02 | | | hypothetical protein |
| RJ610_16485 | WND78892.1 | 1,41 | 9,79E-02 | | | hypothetical protein |
| RJ610_16490 | WND78893.1 | 1,03 | 8,89E-01 | | | hypothetical protein |
| RJ610_16495 | WND78894.1 | 0,47 | 1,95E-05 | | | hypothetical protein |
| RJ610_16500 | WND78895.1 | 0,45 | 8,69E-10 | | | hypothetical protein |
| RJ610_16505 | WND78896.1 | 0,46 | 4,11E-06 | | | hypothetical protein |
| RJ610_16510 | WND78897.1 | 0,43 | 6,58E-12 | | | PP2C family serine/threonine-protein phosphatase |
| RJ610_16515 | WND78898.1 | 0,63 | 2,73E-06 | | | VWA domain-containing protein |
| RJ610_16520 | WND78899.1 | 1,43 | 5,74E-02 | | | phospholipase C, phosphocholine-specific |
| RJ610_16525 | WND78900.1 | 1,22 | 6,43E-02 | | | multidrug effflux MFS transporter |
| RJ610_16530 | WND78901.1 | 1,43 | 1,10E-03 | | | VOC family protein |
| RJ610_16540 | WND78903.1 | 47622,94 | 1,90E-30 | | | hypothetical protein |
| RJ610_16545 | WND78904.1 | 25339,70 | 1,62E-34 | | | hypothetical protein |
| RJ610_16550 | WND78905.1 | 4257,49 | 1,12E-248 | | | hypothetical protein |
| RJ610_16555 | WND78906.1 | 5051,28 | 1,70E-17 | | | lysylphosphatidylglycerol synthase domain-containing protein |
| RJ610_16560 | WND78907.1 | 198,47 | 1,86E-28 | | | glycosyltransferase family 4 protein |
| RJ610_16565 | WND78908.1 | 257,79 | 2,17E-145 | | | glycosyltransferase family 2 protein |
| RJ610_16570 | WND78909.1 | 879,92 | 5,09E-57 | | | hypothetical protein |
| RJ610_16575 | WND78910.1 | 2,01 | 3,31E-05 | | | QsdR family transcriptional regulator |
| RJ610_16580 | WND78911.1 | 1,87 | 1,57E-06 | | | acyl-CoA dehydrogenase |
| RJ610_16585 | WND78912.1 | 13,45 | 6,89E-31 | | | sulfatase-like hydrolase/transferase |
| RJ610_16590 | WND78913.1 | 0,83 | 4,92E-01 | | | beta-eliminating lyase-related protein |
| RJ610_16595 | WND78914.1 | 0,97 | 8,96E-01 | | | histidine kinase |
| RJ610_16600 | WND78915.1 | 1,17 | 4,57E-01 | | | LytTR family DNA-binding domain-containing protein |
| RJ610_16605 | WND78916.1 | 2,10 | 9,00E-05 | | | hypothetical protein |
| RJ610_16610 | WND78917.1 | 1,37 | 2,00E-01 | | | hypothetical protein |
| RJ610_16615 | WND78918.1 | 1,06 | 6,95E-01 | | | hypothetical protein |
| RJ610_16620 | WND78919.1 | 0,81 | 5,50E-01 | | | hypothetical protein |
| RJ610_16625 | WND78920.1 | 0,78 | 1,16E-01 | | | hypothetical protein |
| RJ610_16630 | WND78921.1 | 0,38 | 6,90E-05 | | | dipeptide epimerase |
| RJ610_16635 | WND78922.1 | 0,53 | 1,23E-02 | | | membrane dipeptidase |
| RJ610_16640 | WND78923.1 | 0,50 | 1,98E-02 | | | TonB-dependent receptor |
| RJ610_16645 | WND78924.1 | 0,48 | 1,45E-02 | | | DUF1611 domain-containing protein |
| RJ610_16650 | WND78925.1 | 0,85 | 6,37E-01 | | | hypothetical protein |
| RJ610_16655 | WND78926.1 | 0,77 | 1,76E-01 | | | serine hydrolase domain-containing protein |
| RJ610_16660 | WND78927.1 | 0,59 | 7,58E-04 | | | hypothetical protein |
| RJ610_16665 | WND78928.1 | 1,06 | 7,18E-01 | | | DUF2252 family protein |
| RJ610_16670 | WND78929.1 | 0,81 | 2,21E-01 | | | siderophore-interacting protein |

| | | | | | | |
|---|---|---|---|---|---|---|
| RJ610_16680 | WND78931.1 | 1,22 | 2,50E-01 | | | LysR substrate-binding domain-containing protein |
| RJ610_16685 | WND78932.1 | 1,18 | 4,54E-01 | | | type 1 glutamine amidotransferase domain-containing protein |
| RJ610_16690 | WND78933.1 | 1,36 | 2,58E-01 | | | type 1 glutamine amidotransferase domain-containing protein |
| RJ610_16695 | WND78934.1 | 1,74 | 2,81E-02 | | | carboxymuconolactone decarboxylase family protein |
| RJ610_16700 | WND78935.1 | 1,47 | 7,40E-02 | | | hypothetical protein |
| RJ610_16705 | WND78936.1 | 4,41 | 6,93E-25 | | | phospholipase |
| RJ610_16710 | WND78937.1 | 5,97 | 1,30E-31 | | | ankyrin repeat domain-containing protein |
| RJ610_16715 | WND78938.1 | 16,22 | 2,39E-12 | | | hypothetical protein |
| RJ610_16720 | WND78939.1 | 31,66 | 2,26E-180 | | | hypothetical protein |
| RJ610_16725 | WND83275.1 | 15,83 | 3,58E-80 | | | type 2 lanthipeptide synthetase LanM family protein |
| RJ610_16730 | WND78940.1 | 1,11 | 7,14E-01 | | | hypothetical protein |
| RJ610_16735 | WND78941.1 | 0,64 | 1,34E-02 | | | hypothetical protein |
| RJ610_16740 | WND78942.1 | 0,38 | 5,22E-11 | | | hypothetical protein |
| RJ610_16745 | WND78943.1 | 0,75 | 2,32E-02 | | | adenylosuccinate synthase |
| RJ610_16755 | WND78945.1 | 1,29 | 1,52E-01 | | | DUF2065 family protein |
| RJ610_16760 | WND78946.1 | 1,22 | 2,54E-01 | | | protease modulator HflC |
| RJ610_16765 | WND78947.1 | 1,15 | 4,71E-01 | | | FtsH protease activity modulator HflK |
| RJ610_16770 | WND78948.1 | 1,05 | 8,13E-01 | | | ribosome rescue GTPase HflX |
| RJ610_16775 | WND78949.1 | 0,93 | 4,79E-01 | | | RNA chaperone Hfq |
| RJ610_16780 | WND78950.1 | 0,73 | 5,74E-02 | | | tRNA (adenosine(37)-N6)-dimethylallyltransferase MiaA |
| RJ610_16785 | WND78951.1 | 0,89 | 3,93E-01 | | | dihydropteroate synthase |
| RJ610_16790 | WND78952.1 | 1,23 | 2,93E-01 | | | ATP-dependent zinc metalloprotease FtsH |
| RJ610_16795 | WND78953.1 | 1,37 | 8,70E-02 | | | 23S rRNA (uridine(2552)-2'-O)-methyltransferase RlmE |
| RJ610_16800 | WND78954.1 | 0,90 | 3,53E-01 | | | ribosome assembly RNA-binding protein YhbY |
| RJ610_16805 | WND78955.1 | 0,83 | 1,01E-01 | | | Mth938-like domain-containing protein |
| RJ610_16810 | WND78956.1 | 1,05 | 7,97E-01 | | | hypothetical protein |
| RJ610_16815 | WND78957.1 | 0,47 | 3,11E-07 | | | hypothetical protein |
| RJ610_16820 | WND78958.1 | 0,64 | 2,90E-03 | | | hypothetical protein |
| RJ610_16825 | WND78959.1 | 1,29 | 7,23E-02 | | | hypothetical protein |
| RJ610_16830 | WND78960.1 | 0,99 | 9,70E-01 | | | peptidoglycan DD-metalloendopeptidase family protein |
| RJ610_16835 | WND78961.1 | 0,65 | 1,79E-04 | | | YqaA family protein |
| RJ610_16840 | WND78962.1 | 0,83 | 1,11E-01 | | | protein-L-isoaspartate(D-aspartate) O-methyltransferase |
| RJ610_16845 | WND78963.1 | 0,68 | 2,45E-03 | | | 5'/3'-nucleotidase SurE |
| RJ610_16850 | WND78964.1 | 0,71 | 2,92E-03 | | | LuxR C-terminal-related transcriptional regulator |
| RJ610_16855 | WND78965.1 | 0,56 | 8,46E-04 | | | Smr/MutS family protein |
| RJ610_16860 | WND78966.1 | 0,28 | 3,01E-22 | | | hypothetical protein |
| RJ610_16865 | WND78967.1 | 0,82 | 5,09E-01 | | | hypothetical protein |
| RJ610_16870 | WND83276.1 | 1,05 | 8,03E-01 | | | tRNA pseudouridine(13) synthase TruD |
| RJ610_16880 | WND78969.1 | 1,15 | 4,12E-01 | | | 2-C-methyl-D-erythritol 4-phosphate cytidylyltransferase |
| RJ610_16885 | WND78970.1 | 1,05 | 7,07E-01 | | | cell division protein FtsB |
| RJ610_16895 | WND78972.1 | 0,91 | 5,45E-01 | | | hypothetical protein |
| RJ610_16900 | WND78973.1 | 0,78 | 7,87E-02 | | | 3-deoxy-8-phosphooctulonate synthase |
| RJ610_16905 | WND78974.1 | 0,78 | 8,13E-02 | | | hypothetical protein |

| | | | | | | |
|---|---|---|---|---|---|---|
| RJ610_16910 | WND78975.1 | 0,66 | 1,93E-05 | | | CTP synthase |
| RJ610_16915 | WND78976.1 | 1,00 | 9,86E-01 | | | hypothetical protein |
| RJ610_16920 | WND78977.1 | 1,03 | 8,45E-01 | | | DUF488 family protein |
| RJ610_16925 | WND78978.1 | 1,26 | 5,54E-02 | | | hypothetical protein |
| RJ610_16930 | WND78979.1 | 1,39 | 1,01E-01 | | | hypothetical protein |
| RJ610_16935 | WND78980.1 | 9,21 | 2,07E-21 | | | globin |
| RJ610_16940 | WND78981.1 | 0,64 | 1,45E-02 | | | DNA topoisomerase IV subunit B |
| RJ610_16950 | WND78983.1 | 0,66 | 2,27E-03 | | | hypothetical protein |
| RJ610_16955 | WND78984.1 | 0,84 | 3,04E-01 | | | hypothetical protein |
| RJ610_16960 | WND78985.1 | 1,40 | 1,28E-02 | | | crosslink repair DNA glycosylase YcaQ family protein |
| RJ610_16970 | WND78987.1 | 1,51 | 1,17E-04 | | | prolyl oligopeptidase family serine peptidase |
| RJ610_16980 | WND78989.1 | 1,48 | 1,10E-03 | | | FAD-dependent oxidoreductase |
| RJ610_16985 | WND78990.1 | 1,31 | 1,01E-01 | | | M4 family metallopeptidase |
| RJ610_16990 | WND78991.1 | 11,86 | 2,14E-27 | | | M4 family metallopeptidase |
| RJ610_16995 | WND78992.1 | 1,04 | 8,12E-01 | | | DUF418 domain-containing protein |
| RJ610_17000 | WND78993.1 | 0,25 | 6,53E-27 | | | hypothetical protein |
| RJ610_17010 | WND78995.1 | 0,72 | 2,60E-03 | | | peptidylprolyl isomerase |
| RJ610_17015 | WND83277.1 | 1,60 | 1,38E-06 | | | C40 family peptidase |
| RJ610_17020 | WND78996.1 | 0,78 | 8,26E-02 | | | NlpC/P60 family protein |
| RJ610_17025 | WND78997.1 | 0,75 | 3,88E-02 | | | acyl-CoA thioesterase |
| RJ610_17030 | WND78998.1 | 0,67 | 7,10E-03 | | | NAD(P)H-dependent oxidoreductase |
| RJ610_17035 | WND78999.1 | 0,59 | 4,75E-06 | | | pitrilysin family protein |
| RJ610_17040 | WND79000.1 | 0,52 | 1,30E-07 | | | pitrilysin family protein |
| RJ610_17045 | WND79001.1 | 0,80 | 7,78E-02 | | | 8-oxo-dGTP diphosphatase |
| RJ610_17050 | WND79002.1 | 1,03 | 8,49E-01 | | | hypothetical protein |
| RJ610_17055 | WND79003.1 | 0,70 | 3,94E-03 | | | HlyD family secretion protein |
| RJ610_17060 | WND79004.1 | 0,52 | 2,81E-06 | | | PaaI family thioesterase |
| RJ610_17065 | WND79005.1 | 0,39 | 1,62E-04 | | | TetR/AcrR family transcriptional regulator |
| RJ610_17070 | WND79006.1 | 0,88 | 4,41E-01 | | | DHA2 family efflux MFS transporter permease subunit |
| RJ610_17075 | WND79007.1 | 2,00 | 2,83E-06 | | | hypothetical protein |
| RJ610_17080 | WND79008.1 | 1,41 | 1,79E-03 | | | dicarboxylate/amino acid:cation symporter |
| RJ610_17090 | WND79010.1 | 2,18 | 3,19E-04 | | | hypothetical protein |
| RJ610_17095 | WND79011.1 | 9,52 | 9,45E-30 | | | CopD family protein |
| RJ610_17100 | WND79012.1 | 4,49 | 2,31E-14 | | | class I SAM-dependent methyltransferase |
| RJ610_17105 | WND83278.1 | 0,27 | 1,06E-23 | | | polyhydroxyalkanoate depolymerase |
| RJ610_17110 | WND79013.1 | 29,55 | 5,25E-33 | | | hypothetical protein |
| RJ610_17115 | WND79014.1 | 2,98 | 2,95E-06 | | | hypothetical protein |
| RJ610_17120 | WND79015.1 | 1,31 | 3,97E-01 | | | hypothetical protein |
| RJ610_17125 | WND79016.1 | 0,61 | 4,41E-02 | | | hypothetical protein |
| RJ610_17130 | WND79017.1 | 0,05 | 2,05E-60 | | | sensor domain-containing protein |
| RJ610_17135 | WND79018.1 | 0,05 | 1,06E-71 | | | PadR family transcriptional regulator |
| RJ610_17140 | WND83279.1 | 0,38 | 2,58E-13 | | | peptidylprolyl isomerase |
| RJ610_17145 | WND79019.1 | 1,41 | 9,63E-03 | | | spermidine N1-acetyltransferase |

| | | | | | | |
|---|---|---|---|---|---|---|
| RJ610_17150 | WND79020.1 | 0,83 | 2,43E-01 | | | hypothetical protein |
| RJ610_17155 | WND79021.1 | 0,78 | 1,10E-01 | | | DUF4349 domain-containing protein |
| RJ610_17160 | WND79022.1 | 0,86 | 5,14E-01 | | | TonB-dependent receptor |
| RJ610_17165 | WND79023.1 | 0,64 | 9,72E-02 | | | hypothetical protein |
| RJ610_17175 | WND79025.1 | 0,90 | 6,19E-01 | | | substrate-binding domain-containing protein |
| RJ610_17180 | WND79026.1 | 0,89 | 6,13E-01 | | | hypothetical protein |
| RJ610_17185 | WND79027.1 | 1,29 | 1,97E-01 | | | OFA family MFS transporter |
| RJ610_17190 | WND83280.1 | 1,22 | 3,42E-01 | | | formate dehydrogenase accessory sulfurtransferase FdhD |
| RJ610_17195 | WND79028.1 | 1,21 | 3,60E-01 | | | FdhF/YdeP family oxidoreductase |
| RJ610_17200 | WND79029.1 | 1,37 | 7,67E-02 | | | hypothetical protein |
| RJ610_17205 | WND79030.1 | 1,53 | 5,67E-03 | | | GFA family protein |
| RJ610_17210 | WND79031.1 | 1,50 | 1,42E-02 | | | VOC family protein |
| RJ610_17215 | WND79032.1 | 0,68 | 2,21E-02 | | | DUF72 domain-containing protein |
| RJ610_17220 | WND79033.1 | 1,08 | 6,66E-01 | | | aldo/keto reductase |
| RJ610_17225 | WND79034.1 | 0,86 | 3,99E-01 | | | NAD(P)-dependent oxidoreductase |
| RJ610_17230 | WND79035.1 | 1,41 | 2,03E-02 | | | LysR family transcriptional regulator |
| RJ610_17235 | WND79036.1 | 2,01 | 1,08E-08 | | | cyclase family protein |
| RJ610_17240 | WND79037.1 | 1,86 | 6,91E-11 | | | DMT family transporter |
| RJ610_17245 | WND79038.1 | 0,98 | 9,04E-01 | | | LysR substrate-binding domain-containing protein |
| RJ610_17250 | WND79039.1 | 1,06 | 8,40E-01 | | | hypothetical protein |
| RJ610_17255 | WND79040.1 | 1,99 | 1,21E-05 | | | TfoX/Sxy family protein |
| RJ610_17265 | WND79042.1 | 1,50 | 1,82E-03 | | | M1 family metallopeptidase |
| RJ610_17275 | WND79044.1 | 2,53 | 2,57E-16 | | | ATP-dependent RNA helicase DbpA |
| RJ610_17280 | WND79045.1 | 3,66 | 6,39E-28 | | | hypothetical protein |
| RJ610_17285 | WND79046.1 | 1,99 | 1,05E-07 | | | GreA/GreB family elongation factor |
| RJ610_17290 | WND79047.1 | 0,79 | 2,23E-01 | | | Imm8 family immunity protein |
| RJ610_17295 | WND79048.1 | 0,68 | 2,97E-02 | | | acyl-CoA desaturase |
| RJ610_17300 | WND79049.1 | 0,88 | 4,29E-01 | | | hypothetical protein |
| RJ610_17305 | WND79050.1 | 0,70 | 5,71E-02 | | | sigma-70 family RNA polymerase sigma factor |
| RJ610_17310 | WND79051.1 | 1,01 | 9,39E-01 | | | hypothetical protein |
| RJ610_17315 | WND79052.1 | 1,06 | 7,30E-01 | | | metalloregulator ArsR/SmtB family transcription factor |
| RJ610_17320 | WND79053.1 | 1,30 | 1,20E-02 | | | fatty acid desaturase |
| RJ610_17325 | WND79054.1 | 1,33 | 9,04E-02 | | | GIY-YIG nuclease family protein |
| RJ610_17330 | WND79055.1 | 2,02 | 1,11E-07 | | | MAPEG family protein |
| RJ610_17335 | WND79056.1 | 4,31 | 5,87E-42 | | | zinc-dependent alcohol dehydrogenase family protein |
| RJ610_17340 | WND79057.1 | 0,66 | 3,88E-02 | | | RNA-binding S4 domain-containing protein |
| RJ610_17345 | WND79058.1 | 0,66 | 6,69E-02 | | | hypothetical protein |
| RJ610_17350 | WND79059.1 | 0,79 | 9,42E-02 | | | hypothetical protein |
| RJ610_17355 | WND79060.1 | 1,02 | 8,53E-01 | | | bifunctional nicotinamide-nucleotide adenylyltransferase/Nudix hydroxylase |
| RJ610_17360 | WND79061.1 | 1,22 | 1,51E-01 | | | nicotinate phosphoribosyltransferase |
| RJ610_17365 | WND83281.1 | 0,95 | 7,95E-01 | | | hypothetical protein |
| RJ610_17370 | WND79062.1 | 0,88 | 4,14E-01 | | | DNA methyltransferase |

| | | | | | | |
|---|---|---|---|---|---|---|
| RJ610_17375 | WND79063.1 | 0,70 | 5,31E-02 | | | glycoside hydrolase family 75 protein |
| RJ610_17380 | WND79064.1 | 2,40 | 3,31E-05 | | | GNAT family N-acetyltransferase |
| RJ610_17385 | WND79065.1 | 0,84 | 5,16E-01 | | | NAD(P)-dependent alcohol dehydrogenase |
| RJ610_17390 | WND79066.1 | 0,80 | 1,52E-01 | | | LysR family transcriptional regulator |
| RJ610_17395 | WND79067.1 | 1,39 | 1,90E-02 | | | hypothetical protein |
| RJ610_17400 | WND79068.1 | 1,15 | 3,41E-01 | | | AAA family ATPase |
| RJ610_17405 | WND79069.1 | 0,02 | 3,85E-117 | | | CsgG/HfaB family protein |
| RJ610_17420 | WND79072.1 | 1,25 | 1,19E-01 | | | hypothetical protein |
| RJ610_17425 | WND79073.1 | 0,85 | 1,36E-01 | | | hypothetical protein |
| RJ610_17435 | WND79075.1 | 1,11 | 5,22E-01 | | | TetR/AcrR family transcriptional regulator |
| RJ610_17440 | WND79076.1 | 0,70 | 1,10E-02 | | | hypothetical protein |
| RJ610_17445 | WND79077.1 | 0,30 | 2,21E-13 | | | hypothetical protein |
| RJ610_17450 | WND79078.1 | 0,33 | 1,47E-09 | | | DUF4440 domain-containing protein |
| RJ610_17455 | WND79079.1 | 0,76 | 8,66E-02 | | | ectonucleotide pyrophosphatase/phosphodiesterase |
| RJ610_17465 | WND79081.1 | 0,69 | 3,85E-03 | | | AlkA N-terminal domain-containing protein |
| RJ610_17470 | WND79082.1 | 1,15 | 4,46E-01 | | | methylated-DNA--[protein]-cysteine S-methyltransferase |
| RJ610_17475 | WND79083.1 | 0,72 | 2,03E-02 | | | formimidoylglutamate deiminase |
| RJ610_17480 | WND79084.1 | 0,53 | 1,01E-07 | | | imidazolonepropionase |
| RJ610_17485 | WND79085.1 | 0,81 | 7,16E-02 | | | dipeptidase |
| RJ610_17490 | WND79086.1 | 0,82 | 9,74E-02 | | | DUF6348 family protein |
| RJ610_17500 | WND79088.1 | 0,89 | 3,56E-01 | | | 30S ribosomal protein THX |
| RJ610_17505 | WND79089.1 | 1,47 | 1,05E-02 | | | MerC domain-containing protein |
| RJ610_17515 | WND79091.1 | 0,96 | 7,60E-01 | | | MarR family winged helix-turn-helix transcriptional regulator |
| RJ610_17520 | WND79092.1 | 0,81 | 1,67E-01 | | | MFS transporter |
| RJ610_17525 | WND79093.1 | 1,39 | 1,14E-01 | | | transcriptional repressor |
| RJ610_17530 | WND79094.1 | 0,91 | 3,83E-01 | | | glutamate--tRNA ligase |
| RJ610_17535 | WND79095.1 | 0,68 | 5,55E-03 | | | UDP-2,3-diacylglucosamine diphosphatase |
| RJ610_17540 | WND79096.1 | 0,53 | 7,17E-06 | | | hypothetical protein |
| RJ610_17545 | WND79097.1 | 2,01 | 1,60E-03 | | | TonB-dependent receptor |
| RJ610_17550 | WND79098.1 | 1,47 | 4,67E-02 | | | DPP IV N-terminal domain-containing protein |
| RJ610_17555 | WND79099.1 | 1,08 | 6,49E-01 | | | hypothetical protein |
| RJ610_17560 | WND79100.1 | 1,05 | 7,50E-01 | | | hypothetical protein |
| RJ610_17565 | WND79101.1 | 0,90 | 5,93E-01 | | | DPP IV N-terminal domain-containing protein |
| RJ610_17570 | WND79102.1 | 0,85 | 3,52E-01 | | | ferritin-like domain-containing protein |
| RJ610_17575 | WND79103.1 | 0,94 | 6,68E-01 | | | amidophosphoribosyltransferase |
| RJ610_17580 | WND79104.1 | 1,08 | 5,86E-01 | | | CvpA family protein |
| RJ610_17585 | WND79105.1 | 0,79 | 1,90E-02 | | | SPOR domain-containing protein |
| RJ610_17590 | WND79106.1 | 0,90 | 5,99E-01 | | | bifunctional tetrahydrofolate synthase/dihydrofolate synthase |
| RJ610_17595 | WND79107.1 | 2,17 | 5,35E-03 | | | energy transducer TonB |
| RJ610_17600 | WND79108.1 | 4,67 | 6,25E-04 | | | hypothetical protein |
| RJ610_17605 | WND79109.1 | 6,41 | 3,09E-04 | | | histidine phosphatase family protein |
| RJ610_17610 | WND79110.1 | 3,48 | 5,59E-03 | | | phosphoglycerate dehydrogenase |
| RJ610_17615 | WND79111.1 | 15,04 | 2,07E-55 | | | DUF6289 family protein |

| | | | | | | |
|---|---|---|---|---|---|---|
| RJ610_17620 | WND79112.1 | 26,90 | 5,02E-109 | | | DUF6289 family protein |
| RJ610_17625 | WND79113.1 | 1,41 | 3,72E-04 | | | S46 family peptidase |
| RJ610_17640 | WND79116.1 | 0,68 | 5,78E-04 | | | S46 family peptidase |
| RJ610_17655 | WND79119.1 | 1,08 | 7,54E-01 | | | HNH endonuclease |
| RJ610_17660 | WND79120.1 | 1,25 | 8,73E-02 | | | pseudouridine synthase |
| RJ610_17665 | WND79121.1 | 0,78 | 7,33E-03 | | | hypothetical protein |
| RJ610_17685 | WND79125.1 | 1,02 | 9,20E-01 | | | class III extradiol ring-cleavage dioxygenase |
| RJ610_17690 | WND79126.1 | 1,40 | 4,30E-02 | | | LysR family transcriptional regulator |
| RJ610_17695 | WND79127.1 | 1,45 | 5,47E-02 | | | MFS transporter |
| RJ610_17700 | WND79128.1 | 0,68 | 4,83E-02 | | | LysR family transcriptional regulator |
| RJ610_17705 | WND79129.1 | 1,27 | 5,05E-01 | | | SGNH/GDSL hydrolase family protein |
| RJ610_17710 | WND79130.1 | 0,78 | 2,11E-01 | | | zinc-binding alcohol dehydrogenase family protein |
| RJ610_17715 | WND79131.1 | 0,30 | 5,29E-12 | | | prolyl oligopeptidase family serine peptidase |
| RJ610_17725 | WND79133.1 | 0,33 | 3,14E-18 | | | elongation factor G |
| RJ610_17730 | WND79134.1 | 0,65 | 7,05E-05 | | | AAA family ATPase |
| RJ610_17735 | WND79135.1 | 0,52 | 7,25E-05 | | | hypothetical protein |
| RJ610_17740 | WND79136.1 | 0,50 | 1,42E-07 | | | hypothetical protein |
| RJ610_17745 | WND79137.1 | 1,26 | 8,93E-02 | | | NADPH-dependent 2,4-dienoyl-CoA reductase |
| RJ610_17750 | WND79138.1 | 1,41 | 3,63E-04 | | | cell wall hydrolase |
| RJ610_17755 | WND79139.1 | 1,19 | 1,59E-01 | | | glutathione binding-like protein |
| RJ610_17760 | WND79140.1 | 32,96 | 2,99E-161 | | | hypothetical protein |
| RJ610_17765 | WND79141.1 | 2,10 | 2,36E-11 | | | 2-methylcitrate synthase |
| RJ610_17770 | WND79142.1 | 2,17 | 1,87E-14 | | | methylisocitrate lyase |
| RJ610_17775 | WND79143.1 | 0,45 | 2,81E-12 | | | suppressor of fused domain protein |
| RJ610_17780 | WND79144.1 | 1,79 | 7,73E-08 | | | propionate--CoA ligase |
| RJ610_17785 | WND79145.1 | 3,35 | 3,33E-05 | | | TonB-dependent receptor |
| RJ610_17790 | WND79146.1 | 2,92 | 1,41E-06 | | | MFS transporter |
| RJ610_17795 | | 2,97 | 7,43E-07 | | | dicarboxylate/amino acid:cation symporter |
| RJ610_17800 | WND83282.1 | 1,49 | 3,22E-01 | | | porin |
| RJ610_17805 | WND79147.1 | 1,00 | 9,93E-01 | | | PAS-domain containing protein |
| RJ610_17810 | WND79148.1 | 0,71 | 1,60E-02 | | | response regulator transcription factor |
| RJ610_17820 | WND79150.1 | 1,25 | 4,04E-02 | | | glutaredoxin domain-containing protein |
| RJ610_17825 | WND79151.1 | 1,03 | 8,07E-01 | | | pyridoxal phosphate-dependent aminotransferase |
| RJ610_17830 | WND79152.1 | 0,11 | 8,25E-38 | | | hypothetical protein |
| RJ610_17835 | WND79153.1 | 0,23 | 2,95E-22 | | | M48 family metallopeptidase |
| RJ610_17840 | WND79154.1 | 0,32 | 3,49E-12 | | | hypothetical protein |
| RJ610_17845 | WND79155.1 | 0,99 | 9,63E-01 | | | peptidylprolyl isomerase |
| RJ610_17850 | WND79156.1 | 0,94 | 5,87E-01 | | | translational GTPase TypA |
| RJ610_17855 | WND79157.1 | 1,05 | 8,19E-01 | | | hypothetical protein |
| RJ610_17860 | WND79158.1 | 1,60 | 1,29E-02 | | | DUF2127 domain-containing protein |
| RJ610_17865 | WND79159.1 | 5,44 | 3,05E-29 | | | pyrroloquinoline quinone biosynthesis protein PqqB |
| RJ610_17870 | WND83283.1 | 4,51 | 1,22E-17 | | | pyrroloquinoline-quinone synthase PqqC |
| RJ610_17875 | WND79160.1 | 5,44 | 8,83E-28 | | | pyrroloquinoline quinone biosynthesis peptide chaperone PqqD |

| | | | | | | |
|---|---|---|---|---|---|---|
| RJ610_17880 | WND79161.1 | 3,50 | 1,07E-20 | | | pyrroloquinoline quinone biosynthesis protein PqqE |
| RJ610_17885 | WND79162.1 | 3,16 | 2,77E-12 | | | hypothetical protein |
| RJ610_17890 | WND83284.1 | 0,96 | 8,03E-01 | | | amidase |
| RJ610_17895 | WND79163.1 | 2,01 | 3,71E-04 | | | hypothetical protein |
| RJ610_17900 | WND79164.1 | 2,18 | 1,53E-07 | | | 3-deoxy-7-phosphoheptulonate synthase class II |
| RJ610_17905 | WND79165.1 | 2,56 | 4,02E-20 | | | disulfide bond formation protein B |
| RJ610_17910 | WND79166.1 | 1,23 | 1,24E-01 | | | 50S ribosomal protein L17 |
| RJ610_17915 | WND79167.1 | 1,12 | 4,54E-01 | | | DNA-directed RNA polymerase subunit alpha |
| RJ610_17920 | WND79168.1 | 0,93 | 6,30E-01 | | | 30S ribosomal protein S4 |
| RJ610_17925 | WND79169.1 | 0,81 | 8,95E-02 | | | 30S ribosomal protein S11 |
| RJ610_17930 | WND79170.1 | 0,85 | 1,73E-01 | | | 30S ribosomal protein S13 |
| RJ610_17935 | WND79171.1 | 1,34 | 4,39E-02 | | | preprotein translocase subunit SecY |
| RJ610_17940 | WND79172.1 | 1,12 | 4,99E-01 | | | 50S ribosomal protein L15 |
| RJ610_17945 | WND79173.1 | 1,18 | 3,17E-01 | | | 50S ribosomal protein L30 |
| RJ610_17950 | WND79174.1 | 1,08 | 6,30E-01 | | | 30S ribosomal protein S5 |
| RJ610_17955 | WND79175.1 | 0,97 | 8,03E-01 | | | 50S ribosomal protein L18 |
| RJ610_17960 | WND79176.1 | 0,85 | 2,26E-01 | | | 50S ribosomal protein L6 |
| RJ610_17970 | WND79178.1 | 1,41 | 1,70E-02 | | | 30S ribosomal protein S14 |
| RJ610_17990 | WND79182.1 | 1,29 | 5,56E-02 | | | 30S ribosomal protein S17 |
| RJ610_17995 | WND79183.1 | 1,44 | 4,40E-03 | | | 50S ribosomal protein L29 |
| RJ610_18005 | WND79185.1 | 1,32 | 2,66E-02 | | | 30S ribosomal protein S3 |
| RJ610_18015 | WND79187.1 | 1,29 | 1,13E-01 | | | 30S ribosomal protein S19 |
| RJ610_18025 | WND79189.1 | 1,08 | 6,66E-01 | | | 50S ribosomal protein L23 |
| RJ610_18040 | WND79192.1 | 0,85 | 2,87E-01 | | | 30S ribosomal protein S10 |
| RJ610_18050 | WND79194.1 | 0,96 | 7,38E-01 | | | elongation factor G |
| RJ610_18055 | WND79195.1 | 0,89 | 3,41E-01 | | | 30S ribosomal protein S7 |
| RJ610_18060 | WND79196.1 | 0,88 | 2,70E-01 | | | 30S ribosomal protein S12 |
| RJ610_18065 | WND79197.1 | 1,19 | 2,20E-01 | | | DNA-directed RNA polymerase subunit beta' |
| RJ610_18070 | WND83285.1 | 1,19 | 2,38E-01 | | | DNA-directed RNA polymerase subunit beta |
| RJ610_18075 | WND79198.1 | 0,98 | 9,29E-01 | | | 50S ribosomal protein L7/L12 |
| RJ610_18080 | WND79199.1 | 0,96 | 8,29E-01 | | | 50S ribosomal protein L10 |
| RJ610_18085 | WND79200.1 | 0,91 | 5,25E-01 | | | 50S ribosomal protein L1 |
| RJ610_18090 | WND79201.1 | 0,80 | 7,85E-02 | | | 50S ribosomal protein L11 |
| RJ610_18095 | WND79202.1 | 1,01 | 9,40E-01 | | | transcription termination/antitermination protein NusG |
| RJ610_18100 | WND79203.1 | 0,83 | 9,05E-02 | | | preprotein translocase subunit SecE |
| RJ610_18105 | | 0,99 | 9,17E-01 | | | tRNA-Trp |
| RJ610_18115 | | 1,02 | 9,17E-01 | | | tRNA-Thr |
| RJ610_18120 | | 1,01 | 9,27E-01 | | | tRNA-Gly |
| RJ610_18125 | | 0,79 | 8,16E-02 | | | tRNA-Tyr |
| RJ610_18130 | WND79205.1 | 1,14 | 3,28E-01 | | | redox-regulated ATPase YchF |
| RJ610_18135 | WND79206.1 | 1,02 | 9,14E-01 | | | aminoacyl-tRNA hydrolase |
| RJ610_18145 | WND83286.1 | 0,95 | 7,05E-01 | | | ribose-phosphate diphosphokinase |
| RJ610_18150 | | 0,90 | 5,07E-01 | | | tRNA-Gln |

| | | | | | | |
|---|---|---|---|---|---|---|
| RJ610_18155 | WND79208.1 | 0,88 | 3,22E-01 | | | 4-(cytidine 5'-diphospho)-2-C-methyl-D-erythritol kinase |
| RJ610_18160 | WND79209.1 | 0,82 | 1,63E-01 | | | lipoprotein insertase outer membrane protein LolB |
| RJ610_18165 | WND79210.1 | 0,87 | 2,77E-01 | | | tetratricopeptide repeat protein |
| RJ610_18170 | WND79211.1 | 0,86 | 2,85E-01 | | | glutamyl-tRNA reductase |
| RJ610_18175 | WND79212.1 | 0,68 | 3,74E-03 | | | peptide chain release factor 1 |
| RJ610_18180 | WND79213.1 | 1,21 | 3,85E-01 | | | GNAT family N-acetyltransferase |
| RJ610_18185 | WND79214.1 | 1,01 | 9,64E-01 | | | tetratricopeptide repeat protein |
| RJ610_18195 | WND79216.1 | 0,61 | 1,30E-04 | | | helix-turn-helix transcriptional regulator |
| RJ610_18200 | WND79217.1 | 0,66 | 1,37E-04 | | | polyphosphate kinase 2 |
| RJ610_18205 | WND79218.1 | 0,69 | 6,40E-03 | | | helix-turn-helix transcriptional regulator |
| RJ610_18210 | WND79219.1 | 1,87 | 7,44E-05 | | | hypothetical protein |
| RJ610_18215 | WND79220.1 | 1,79 | 4,63E-05 | | | DUF2145 domain-containing protein |
| RJ610_18220 | WND79221.1 | 0,93 | 6,95E-01 | | | hypothetical protein |
| RJ610_18225 | WND79222.1 | 0,76 | 1,03E-01 | | | acylphosphatase |
| RJ610_18230 | | 0,60 | 3,79E-05 | | | TlpA disulfide reductase family protein |
| RJ610_18235 | WND79223.1 | 0,42 | 4,74E-14 | | | YihY family inner membrane protein |
| RJ610_18240 | WND79224.1 | 0,85 | 3,04E-01 | | | NAD(P)H:quinone oxidoreductase |
| RJ610_18245 | WND79225.1 | 0,91 | 6,67E-01 | | | DUF2069 domain-containing protein |
| RJ610_18250 | WND79226.1 | 1,16 | 3,38E-01 | | | hypothetical protein |
| RJ610_18255 | WND79227.1 | 0,91 | 3,80E-01 | | | asparaginase domain-containing protein |
| RJ610_18260 | WND79228.1 | 1,27 | 3,33E-02 | | | hypothetical protein |
| RJ610_18300 | WND79236.1 | 1,08 | 4,91E-01 | | | hypothetical protein |
| RJ610_18305 | WND79237.1 | 2,54 | 2,99E-08 | | | S8 family serine peptidase |
| RJ610_18310 | WND79238.1 | 2,87 | 2,38E-15 | | | S8 family serine peptidase |
| RJ610_18315 | WND79239.1 | 1,86 | 4,48E-04 | | | epimerase |
| RJ610_18320 | WND79240.1 | 0,94 | 7,64E-01 | | | oxidoreductase |
| RJ610_18325 | WND79241.1 | 0,56 | 6,26E-03 | | | SDR family NAD(P)-dependent oxidoreductase |
| RJ610_18330 | WND79242.1 | 0,70 | 3,42E-03 | | | TetR/AcrR family transcriptional regulator |
| RJ610_18335 | WND79243.1 | 0,80 | 1,35E-01 | | | S8 family serine peptidase |
| RJ610_18340 | WND79244.1 | 1,22 | 1,32E-01 | | | S8 family serine peptidase |
| RJ610_18345 | WND79245.1 | 2,50 | 1,12E-11 | | | S8 family serine peptidase |
| RJ610_18350 | WND79246.1 | 0,94 | 8,45E-01 | | | S8 family serine peptidase |
| RJ610_18355 | WND79247.1 | 6,17 | 1,62E-12 | | | S8 family serine peptidase |
| RJ610_18360 | WND79248.1 | 0,71 | 9,12E-02 | | | proprotein convertase P-domain-containing protein |
| RJ610_18365 | WND79249.1 | 0,92 | 6,44E-01 | | | putative Fe-S cluster assembly protein SufT |
| RJ610_18370 | WND79250.1 | 0,86 | 2,19E-01 | | | hypothetical protein |
| RJ610_18375 | WND79251.1 | 0,68 | 9,32E-03 | | | PLP-dependent aminotransferase family protein |
| RJ610_18380 | WND79252.1 | 0,72 | 3,82E-02 | | | AraC family transcriptional regulator |
| RJ610_18385 | WND79253.1 | 0,63 | 3,43E-02 | | | hypothetical protein |
| RJ610_18390 | WND79254.1 | 1,33 | 1,91E-01 | | | cytochrome P460 family protein |
| RJ610_18395 | WND79255.1 | 0,75 | 2,92E-02 | | | DUF1456 family protein |
| RJ610_18400 | WND79256.1 | 0,73 | 8,28E-03 | | | hypothetical protein |
| RJ610_18405 | WND79257.1 | 1,54 | 6,73E-05 | | | hypothetical protein |

| | | | | | | |
|---|---|---|---|---|---|---|
| RJ610_18410 | WND83287.1 | 1,49 | 4,65E-04 | | | NAD(P)(+) transhydrogenase (Re/Si-specific) subunit beta |
| RJ610_18415 | WND79258.1 | 1,35 | 1,11E-02 | | | NAD(P) transhydrogenase subunit alpha |
| RJ610_18420 | WND83288.1 | 0,65 | 1,86E-03 | | | RNA polymerase sigma factor |
| RJ610_18425 | WND79259.1 | 0,64 | 1,39E-03 | | | hypothetical protein |
| RJ610_18430 | WND79260.1 | 0,79 | 1,30E-01 | | | DUF3106 domain-containing protein |
| RJ610_18435 | WND79261.1 | 1,35 | 9,33E-03 | | | hypothetical protein |
| RJ610_18440 | WND79262.1 | 1,42 | 3,71E-03 | | | NAD(P) transhydrogenase subunit alpha |
| RJ610_18445 | WND79263.1 | 6,66 | 2,18E-95 | | | DUF1631 family protein |
| RJ610_18450 | WND79264.1 | 1,58 | 1,44E-04 | | | nitroreductase |
| RJ610_18455 | WND79265.1 | 1,18 | 1,22E-01 | | | 5'-3' exonuclease H3TH domain-containing protein |
| RJ610_18460 | WND79266.1 | 1,23 | 3,26E-02 | | | NUDIX hydrolase |
| RJ610_18465 | WND79267.1 | 1,76 | 1,22E-06 | | | UDP-glucose 4-epimerase GalE |
| RJ610_18470 | WND79268.1 | 1,43 | 1,07E-02 | | | hypothetical protein |
| RJ610_18475 | WND79269.1 | 0,91 | 4,53E-01 | | | glycosyltransferase 61 family protein |
| RJ610_18480 | WND79270.1 | 0,88 | 3,38E-01 | | | hypothetical protein |
| RJ610_18490 | WND79272.1 | 1,05 | 7,51E-01 | | | peptide chain release factor N(5)-glutamine methyltransferase |
| RJ610_18510 | WND79276.1 | 0,82 | 3,00E-01 | | | LysR substrate-binding domain-containing protein |
| RJ610_18515 | WND79277.1 | 1,53 | 2,45E-02 | | | DJ-1/PfpI family protein |
| RJ610_18520 | WND83289.1 | 1,18 | 4,81E-01 | | | peptide-methionine (S)-S-oxide reductase MsrA |
| RJ610_18530 | WND79279.1 | 1,36 | 5,61E-02 | | | hypothetical protein |
| RJ610_18535 | WND79280.1 | 0,90 | 4,01E-01 | | | DMT family transporter |
| RJ610_18540 | WND79281.1 | 1,32 | 7,70E-02 | | | DUF2007 domain-containing protein |
| RJ610_18545 | WND79282.1 | 0,68 | 1,36E-04 | | | glutamine--tRNA ligase/YqeY domain fusion protein |
| RJ610_18550 | WND83290.1 | 0,76 | 2,53E-02 | | | nucleoside deaminase |
| RJ610_18555 | WND79283.1 | 0,97 | 8,76E-01 | | | 23S rRNA (cytidine(2498)-2'-O)-methyltransferase RlmM |
| RJ610_18560 | WND79284.1 | 1,45 | 9,11E-02 | | | hypothetical protein |
| RJ610_18565 | WND79285.1 | 0,77 | 1,02E-01 | | | MFS transporter |
| RJ610_18570 | WND79286.1 | 1,66 | 7,78E-02 | | | hypothetical protein |
| RJ610_18575 | WND79287.1 | 1,31 | 1,93E-01 | | | hypothetical protein |
| RJ610_18580 | WND79288.1 | 1,24 | 1,26E-01 | | | FAD-dependent oxidoreductase |
| RJ610_18585 | WND83291.1 | 1,26 | 4,52E-02 | | | FAD-dependent monooxygenase |
| RJ610_18590 | WND79289.1 | 0,73 | 6,81E-04 | | | hypothetical protein |
| RJ610_18595 | WND79290.1 | 0,82 | 4,89E-02 | | | cob(I)yrinic acid a,c-diamide adenosyltransferase |
| RJ610_18600 | WND79291.1 | 0,82 | 1,44E-01 | | | histone deacetylase family protein |
| RJ610_18605 | WND79292.1 | 0,81 | 1,53E-01 | | | hypothetical protein |
| RJ610_18610 | WND79293.1 | 1,75 | 8,56E-02 | | | hypothetical protein |
| RJ610_18615 | WND79294.1 | 1,23 | 3,41E-01 | | | peptide-methionine (R)-S-oxide reductase MsrB |
| RJ610_18625 | WND79296.1 | 0,85 | 8,36E-02 | | | peptidylprolyl isomerase |
| RJ610_18630 | WND79297.1 | 0,92 | 5,23E-01 | | | 4-hydroxythreonine-4-phosphate dehydrogenase PdxA |
| RJ610_18635 | WND79298.1 | 0,88 | 2,63E-01 | | | 16S rRNA (adenine(1518)-N(6)/adenine(1519)-N(6))-dimethyltransferase RsmA |
| RJ610_18640 | WND79299.1 | 0,57 | 5,32E-09 | | | Co2+/Mg2+ efflux protein ApaG |
| RJ610_18645 | WND79300.1 | 0,55 | 1,26E-10 | | | symmetrical bis(5'-nucleosyl)-tetraphosphatase |

| | | | | | | |
|---|---|---|---|---|---|---|
| RJ610_18650 | WND79301.1 | 0,54 | 2,85E-04 | | | DUF1287 domain-containing protein |
| RJ610_18655 | WND79302.1 | 0,93 | 7,28E-01 | | | hypothetical protein |
| RJ610_18660 | WND79303.1 | 1,00 | 9,85E-01 | | | dihydrofolate reductase |
| RJ610_18665 | WND79304.1 | 0,83 | 1,90E-01 | | | DUF3828 domain-containing protein |
| RJ610_18670 | WND79305.1 | 0,72 | 2,14E-03 | | | thymidylate synthase |
| RJ610_18675 | WND79306.1 | 0,67 | 1,65E-04 | | | prolipoprotein diacylglyceryl transferase |
| RJ610_18680 | WND79307.1 | 1,02 | 9,10E-01 | | | TPM domain-containing protein |
| RJ610_18685 | WND83292.1 | 0,86 | 4,99E-01 | | | TPM domain-containing protein |
| RJ610_18690 | WND79308.1 | 0,82 | 3,31E-01 | | | TPM domain-containing protein |
| RJ610_18695 | WND79309.1 | 0,85 | 3,56E-01 | | | TPM domain-containing protein |
| RJ610_18700 | WND79310.1 | 0,54 | 2,30E-07 | | | LemA family protein |
| RJ610_18705 | WND79311.1 | 0,68 | 7,86E-04 | | | TerC family protein |
| RJ610_18710 | WND79312.1 | 0,83 | 1,23E-01 | | | diacylglycerol kinase |
| RJ610_18715 | WND79313.1 | 0,88 | 4,16E-01 | | | ABC transporter permease |
| RJ610_18720 | WND79314.1 | 0,73 | 8,00E-02 | | | ABC transporter ATP-binding protein |
| RJ610_18725 | WND79315.1 | 0,74 | 1,81E-02 | | | efflux RND transporter periplasmic adaptor subunit |
| RJ610_18730 | WND79316.1 | 0,63 | 4,19E-05 | | | hypothetical protein |
| RJ610_18735 | WND79317.1 | 0,67 | 5,69E-03 | | | HAMP domain-containing sensor histidine kinase |
| RJ610_18740 | WND79318.1 | 0,50 | 3,26E-06 | | | response regulator transcription factor |
| RJ610_18745 | WND79319.1 | 0,60 | 8,10E-04 | | | DMT family protein |
| RJ610_18750 | WND79320.1 | 0,70 | 3,13E-03 | | | hypothetical protein |
| RJ610_18755 | WND79321.1 | 0,62 | 1,47E-02 | | | hypothetical protein |
| RJ610_18760 | WND79322.1 | 0,77 | 3,33E-02 | | | hypothetical protein |
| RJ610_18765 | WND79323.1 | 0,73 | 2,73E-02 | | | DUF962 domain-containing protein |
| RJ610_18770 | WND79324.1 | 0,68 | 1,16E-02 | | | hypothetical protein |
| RJ610_18775 | WND79325.1 | 0,65 | 1,03E-04 | | | hypothetical protein |
| RJ610_18780 | WND79326.1 | 1,11 | 6,42E-01 | | | YnfA family protein |
| RJ610_18785 | WND79327.1 | 0,95 | 7,35E-01 | | | nucleoside triphosphate pyrophosphohydrolase |
| RJ610_18790 | WND79328.1 | 0,75 | 6,07E-03 | | | 3'(2'),5'-bisphosphate nucleotidase CysQ |
| RJ610_18795 | | 0,75 | 4,94E-03 | | | ADP compounds hydrolase NudE |
| RJ610_18805 | WND79329.1 | 1,13 | 4,74E-01 | | | 16S rRNA (uracil(1498)-N(3))-methyltransferase |
| RJ610_18810 | WND79330.1 | 1,18 | 6,25E-01 | | | chemotaxis protein CheX |
| RJ610_18815 | WND79331.1 | 1,43 | 2,80E-01 | | | response regulator |
| RJ610_18820 | WND79332.1 | 1,42 | 7,85E-02 | | | ATP-binding protein |
| RJ610_18825 | | 0,69 | 1,36E-01 | | | porin |
| RJ610_18830 | WND79333.1 | 1,03 | 9,58E-01 | | | hypothetical protein |
| RJ610_18835 | WND79334.1 | 2,46 | 1,26E-11 | | | heparan-alpha-glucosaminide N-acetyltransferase domain-containing protein |
| RJ610_18840 | WND79335.1 | 2,01 | 7,68E-11 | | | chemotaxis protein CheW |
| RJ610_18845 | WND79336.1 | 1,90 | 4,42E-12 | | | chemotaxis protein CheB |
| RJ610_18850 | WND79337.1 | 2,16 | 1,36E-17 | | | Hpt domain-containing protein |
| RJ610_18855 | WND79338.1 | 1,62 | 1,26E-06 | | | methyl-accepting chemotaxis protein |
| RJ610_18860 | WND79339.1 | 1,56 | 6,64E-05 | | | chemotaxis protein CheW |

| | | | | | | |
|---|---|---|---|---|---|---|
| RJ610_18865 | WND79340.1 | 2,10 | 5,56E-13 | | | response regulator |
| RJ610_18870 | WND79341.1 | 2,68 | 1,20E-20 | | | twitching motility response regulator PilG |
| RJ610_18875 | WND79342.1 | 1,86 | 1,08E-09 | | | glutathione synthase |
| RJ610_18880 | WND79343.1 | 1,66 | 5,34E-06 | | | energy transducer TonB |
| RJ610_18885 | WND79344.1 | 1,31 | 9,31E-02 | | | hypothetical protein |
| RJ610_18890 | WND79345.1 | 0,83 | 2,79E-01 | | | hypothetical protein |
| RJ610_18895 | WND79346.1 | 0,75 | 2,88E-02 | | | tRNA (adenosine(37)-N6)-threonylcarbamoyltransferase complex dimerization subunit type 1 TsaB |
| RJ610_18900 | WND79347.1 | 0,74 | 1,98E-02 | | | VOC family protein |
| RJ610_18905 | WND79348.1 | 0,78 | 2,43E-02 | | | ATP-dependent DNA helicase |
| RJ610_18910 | WND79349.1 | 0,67 | 1,56E-03 | | | hypothetical protein |
| RJ610_18915 | WND79350.1 | 0,46 | 4,67E-15 | | | penicillin-binding protein 1B |
| RJ610_18920 | WND79351.1 | 0,82 | 2,02E-01 | | | glycosyltransferase family 2 protein |
| RJ610_18925 | WND79352.1 | 0,74 | 1,85E-02 | | | glycosyltransferase family 2 protein |
| RJ610_18930 | WND79353.1 | 0,58 | 4,00E-07 | | | hypothetical protein |
| RJ610_18935 | WND79354.1 | 0,85 | 2,47E-01 | | | hypothetical protein |
| RJ610_18940 | WND79355.1 | 0,55 | 3,71E-07 | | | putative Ig domain-containing protein |
| RJ610_18945 | WND79356.1 | 0,97 | 7,88E-01 | | | bifunctional (p)ppGpp synthetase/guanosine-3',5'-bis(diphosphate) 3'-pyrophosphohydrolase |
| RJ610_18950 | WND79357.1 | 0,65 | 4,65E-04 | | | hypothetical protein |
| RJ610_18955 | WND79358.1 | 0,70 | 2,40E-03 | | | hypothetical protein |
| RJ610_18960 | WND79359.1 | 0,83 | 3,88E-01 | | | DUF3418 domain-containing protein |
| RJ610_18965 | WND79360.1 | 0,70 | 1,62E-02 | | | Dps family protein |
| RJ610_18970 | WND79361.1 | 0,99 | 9,22E-01 | | | DNA helicase RecQ |
| RJ610_18975 | WND79362.1 | 9,94 | 1,93E-52 | | | ShlB/FhaC/HecB family hemolysin secretion/activation protein |
| RJ610_18980 | WND79363.1 | 4,36 | 6,01E-30 | | | collagen-like triple helix repeat-containing protein |
| RJ610_18985 | WND79364.1 | 1,58 | 4,64E-04 | | | PhzF family phenazine biosynthesis protein |
| RJ610_18990 | WND79365.1 | 31,34 | 1,33E-44 | | | hypothetical protein |
| RJ610_19000 | WND79367.1 | 29,73 | 3,96E-70 | | | response regulator transcription factor |
| RJ610_19005 | WND79368.1 | 11,54 | 1,40E-51 | | | sensor histidine kinase |
| RJ610_19010 | WND79369.1 | 7,25 | 5,98E-33 | | | ABC transporter substrate-binding protein |
| RJ610_19015 | WND79370.1 | 1,99 | 2,22E-05 | | | hypothetical protein |
| RJ610_19020 | WND79371.1 | 1,10 | 5,78E-01 | | | hypothetical protein |
| RJ610_19025 | WND79372.1 | 0,92 | 5,94E-01 | | | hypothetical protein |
| RJ610_19030 | WND79373.1 | 0,59 | 5,21E-04 | | | hypothetical protein |
| RJ610_19035 | WND79374.1 | 0,75 | 6,40E-01 | | | hypothetical protein |
| RJ610_19040 | WND79375.1 | 0,89 | 3,56E-01 | | | hypothetical protein |
| RJ610_19045 | WND79376.1 | 0,86 | 2,44E-01 | | | hypothetical protein |
| RJ610_19050 | WND79377.1 | 0,22 | 3,49E-24 | | | hypothetical protein |
| RJ610_19055 | WND79378.1 | 0,14 | 1,09E-16 | | | hypothetical protein |
| RJ610_19060 | WND79379.1 | 0,05 | 1,06E-32 | | | hypothetical protein |
| RJ610_19065 | WND79380.1 | 0,48 | 1,71E-09 | | | hypothetical protein |
| RJ610_19070 | WND79381.1 | 0,65 | 1,93E-05 | | | hypothetical protein |

| | | | | | | |
|---|---|---|---|---|---|---|
| RJ610_19075 | WND79382.1 | 0,07 | 1,36E-70 | | | D-Ala-D-Ala carboxypeptidase family metallohydrolase |
| RJ610_19080 | WND79383.1 | 0,28 | 3,23E-30 | | | hypothetical protein |
| RJ610_19085 | WND79384.1 | 0,02 | 1,43E-60 | | | hypothetical protein |
| RJ610_19090 | WND79385.1 | 0,01 | 2,47E-42 | | | aspartyl/asparaginyl beta-hydroxylase domain-containing protein |
| RJ610_19095 | WND79386.1 | 0,02 | 4,58E-145 | | | packaged DNA stabilization protein |
| RJ610_19100 | WND79387.1 | 0,05 | 1,91E-70 | | | packaged DNA stabilization gp4 family protein |
| RJ610_19105 | WND79388.1 | 0,03 | 1,76E-50 | | | hypothetical protein |
| RJ610_19110 | WND79389.1 | 0,01 | 1,73E-116 | | | P22 phage major capsid protein family protein |
| RJ610_19115 | WND79390.1 | 0,01 | 7,35E-17 | | | hypothetical protein |
| RJ610_19120 | WND79391.1 | 0,05 | 2,93E-68 | | | portal protein |
| RJ610_19125 | WND79392.1 | 0,06 | 1,37E-91 | | | hypothetical protein |
| RJ610_19130 | WND79393.1 | 0,07 | 1,48E-94 | | | hypothetical protein |
| RJ610_19135 | WND79394.1 | 0,02 | 1,40E-57 | | | hypothetical protein |
| RJ610_19140 | WND79395.1 | 0,01 | 3,26E-32 | | | hypothetical protein |
| RJ610_19145 | WND79396.1 | 0,02 | 3,85E-60 | | | hypothetical protein |
| RJ610_19150 | WND79397.1 | 0,01 | 5,69E-32 | | | hypothetical protein |
| RJ610_19155 | WND79398.1 | 0,01 | 5,32E-45 | | | hypothetical protein |
| RJ610_19160 | WND79399.1 | 0,01 | 3,82E-78 | | | M15 family metallopeptidase |
| RJ610_19165 | WND79400.1 | 0,01 | 2,07E-71 | | | hypothetical protein |
| RJ610_19170 | WND79401.1 | 0,06 | 7,60E-44 | | | hypothetical protein |
| RJ610_19175 | WND79402.1 | 0,05 | 1,87E-90 | | | hypothetical protein |
| RJ610_19180 | WND79403.1 | 0,07 | 6,34E-44 | | | Ref family recombination enhancement nuclease |
| RJ610_19185 | WND79404.1 | 0,03 | 7,72E-86 | | | hypothetical protein |
| RJ610_19190 | WND79405.1 | 0,01 | 3,58E-111 | | | hypothetical protein |
| RJ610_19195 | WND79406.1 | 0,02 | 1,81E-80 | | | hypothetical protein |
| RJ610_19200 | WND79407.1 | 0,01 | 1,96E-189 | | | DnaB-like helicase C-terminal domain-containing protein |
| RJ610_19205 | WND79408.1 | 0,03 | 6,58E-142 | | | hypothetical protein |
| RJ610_19210 | WND79409.1 | 0,01 | 1,26E-72 | | | hypothetical protein |
| RJ610_19215 | WND79410.1 | 0,01 | 2,45E-89 | | | hypothetical protein |
| RJ610_19220 | WND79411.1 | 0,01 | 6,94E-177 | | | RNA methyltransferase |
| RJ610_19225 | WND79412.1 | 0,01 | 8,41E-34 | | | hypothetical protein |
| RJ610_19230 | WND79413.1 | 0,00 | 3,26E-25 | | | hypothetical protein |
| RJ610_19235 | WND79414.1 | 0,01 | 2,73E-164 | | | helix-turn-helix transcriptional regulator |
| RJ610_19240 | WND79415.1 | 0,37 | 3,82E-23 | | | hypothetical protein |
| RJ610_19245 | WND79416.1 | 0,34 | 1,38E-27 | | | hypothetical protein |
| RJ610_19250 | WND79417.1 | 0,12 | 1,76E-67 | | | helix-turn-helix transcriptional regulator |
| RJ610_19255 | WND79418.1 | 0,09 | 1,41E-87 | | | hypothetical protein |
| RJ610_19260 | WND79419.1 | 0,12 | 6,80E-81 | | | hypothetical protein |
| RJ610_19265 | WND79420.1 | 0,01 | 1,82E-194 | | | hypothetical protein |
| RJ610_19270 | WND79421.1 | 0,01 | 9,06E-247 | | | hypothetical protein |
| RJ610_19275 | WND79422.1 | 0,02 | 1,12E-162 | | | hypothetical protein |
| RJ610_19280 | WND79423.1 | 0,02 | 1,81E-133 | | | hypothetical protein |
| RJ610_19285 | WND79424.1 | 0,04 | 1,13E-126 | | | hypothetical protein |

| | | | | | | |
|---|---|---|---|---|---|---|
| RJ610_19290 | WND79425.1 | 0,06 | 2,66E-128 | | | hypothetical protein |
| RJ610_19295 | WND79426.1 | 0,01 | 1,80E-142 | | | hypothetical protein |
| RJ610_19300 | WND79427.1 | 0,02 | 7,70E-94 | | | recombination protein NinB |
| RJ610_19305 | WND79428.1 | 0,01 | 8,76E-187 | | | hypothetical protein |
| RJ610_19310 | WND79429.1 | 0,02 | 1,25E-168 | | | YqaJ viral recombinase family protein |
| RJ610_19315 | WND79430.1 | 0,02 | 1,55E-97 | | | hypothetical protein |
| RJ610_19320 | WND79431.1 | 0,03 | 1,42E-111 | | | hypothetical protein |
| RJ610_19325 | WND79432.1 | 0,04 | 2,29E-159 | | | hypothetical protein |
| RJ610_19330 | WND79433.1 | 0,08 | 3,46E-107 | | | hypothetical protein |
| RJ610_19335 | WND79434.1 | 0,08 | 1,10E-93 | | | hypothetical protein |
| RJ610_19340 | WND79435.1 | 0,08 | 4,33E-84 | | | hypothetical protein |
| RJ610_19345 | WND79436.1 | 0,18 | 1,97E-46 | | | hypothetical protein |
| RJ610_19350 | WND79437.1 | 0,14 | 1,56E-36 | | | hypothetical protein |
| RJ610_19355 | WND79438.1 | 0,16 | 2,16E-55 | | | DUF4224 domain-containing protein |
| RJ610_19360 | WND83294.1 | 0,57 | 6,35E-08 | | | tyrosine-type recombinase/integrase |
| RJ610_19365 | | 1,45 | 4,74E-03 | | | tRNA-Lys |
| RJ610_19370 | WND79439.1 | 0,42 | 2,56E-19 | | | 7-cyano-7-deazaguanine synthase QueC |
| RJ610_19375 | WND79440.1 | 0,60 | 5,84E-06 | | | 7-carboxy-7-deazaguanine synthase QueE |
| RJ610_19395 | WND79444.1 | 0,56 | 3,13E-07 | | | hypothetical protein |
| RJ610_19400 | WND79445.1 | 0,57 | 1,39E-07 | | | hypothetical protein |
| RJ610_19405 | WND79446.1 | 0,63 | 5,55E-05 | | | cell envelope integrity protein TolA |
| RJ610_19410 | WND79447.1 | 0,53 | 1,01E-08 | | | protein TolR |
| RJ610_19415 | WND79448.1 | 0,57 | 5,78E-08 | | | protein TolQ |
| RJ610_19420 | WND79449.1 | 0,64 | 1,78E-05 | | | tol-pal system-associated acyl-CoA thioesterase |
| RJ610_19425 | WND79450.1 | 0,79 | 2,27E-02 | | | Holliday junction branch migration DNA helicase RuvB |
| RJ610_19430 | WND79451.1 | 1,05 | 8,22E-01 | | | SMI1/KNR4 family protein |
| RJ610_19435 | WND83295.1 | 1,25 | 5,28E-02 | | | potassium transporter Kup |
| RJ610_19440 | WND83296.1 | 0,78 | 2,01E-02 | | | potassium transporter Kup |
| RJ610_19445 | WND79452.1 | 0,93 | 6,15E-01 | | | Holliday junction branch migration protein RuvA |
| RJ610_19450 | WND79453.1 | 0,85 | 1,92E-01 | | | crossover junction endodeoxyribonuclease RuvC |
| RJ610_19455 | WND79454.1 | 0,69 | 2,14E-04 | | | YebC/PmpR family DNA-binding transcriptional regulator |
| RJ610_19460 | WND79455.1 | 1,42 | 5,83E-02 | | | alpha/beta hydrolase |
| RJ610_19465 | WND79456.1 | 78,06 | 2,76E-82 | | | DUF4832 domain-containing protein |
| RJ610_19475 | WND79458.1 | 74,59 | 8,91E-99 | | | hypothetical protein |
| RJ610_19480 | WND79459.1 | 1,07 | 7,52E-01 | | | hypothetical protein |
| RJ610_19485 | WND79460.1 | 1,06 | 6,59E-01 | | | GNAT family N-acetyltransferase |
| RJ610_19490 | WND79461.1 | 0,84 | 8,38E-02 | | | aspartate--tRNA ligase |
| RJ610_19495 | WND79462.1 | 0,75 | 9,65E-02 | | | zinc ribbon domain-containing protein |
| RJ610_19500 | WND79463.1 | 0,55 | 5,56E-05 | | | Slp family lipoprotein |
| RJ610_19505 | WND79464.1 | 0,68 | 7,62E-05 | | | ATP-binding cassette domain-containing protein |
| RJ610_19515 | WND79466.1 | 0,79 | 8,66E-02 | | | alpha/beta hydrolase |
| RJ610_19520 | WND79467.1 | 1,65 | 1,33E-04 | | | YbaL family putative K(+) efflux transporter |
| RJ610_19525 | WND79468.1 | 1,07 | 5,89E-01 | | | YceH family protein |

| | | | | | | |
|---|---|---|---|---|---|---|
| RJ610_19530 | WND79469.1 | 0,76 | 2,35E-02 | | | AraC family transcriptional regulator |
| RJ610_19535 | WND83297.1 | 0,45 | 1,37E-03 | | | adenosylcobinamide-GDP ribazoletransferase |
| RJ610_19540 | WND79470.1 | 0,44 | 2,14E-05 | | | histidine phosphatase family protein |
| RJ610_19545 | WND83298.1 | 0,35 | 9,76E-12 | | | nicotinate-nucleotide--dimethylbenzimidazole phosphoribosyltransferase |
| RJ610_19550 | WND79471.1 | 0,44 | 2,81E-05 | | | bifunctional adenosylcobinamide kinase/adenosylcobinamide-phosphate guanylyltransferase |
| RJ610_19555 | WND79472.1 | 0,43 | 4,01E-11 | | | cobyric acid synthase |
| RJ610_19560 | WND79473.1 | 0,41 | 4,62E-09 | | | threonine-phosphate decarboxylase CobD |
| RJ610_19565 | WND79474.1 | 0,38 | 1,77E-06 | | | adenosylcobinamide-phosphate synthase CbiB |
| RJ610_19570 | WND83299.1 | 0,43 | 1,83E-09 | | | cobyrinate a,c-diamide synthase |
| RJ610_19575 | WND79475.1 | 0,39 | 2,20E-10 | | | cob(I)yrinic acid a,c-diamide adenosyltransferase |
| RJ610_19580 | WND83300.1 | 0,38 | 2,34E-09 | | | hypothetical protein |
| RJ610_19585 | WND79476.1 | 0,45 | 1,04E-09 | | | hypothetical protein |
| RJ610_19595 | WND79477.1 | 1,62 | 1,02E-02 | | | SDR family oxidoreductase |
| RJ610_19600 | WND79478.1 | 1,12 | 6,70E-01 | | | hypothetical protein |
| RJ610_19610 | WND79480.1 | 2,10 | 1,32E-05 | | | amidohydrolase family protein |
| RJ610_19615 | WND79481.1 | 1,08 | 7,18E-01 | | | ATP-dependent chaperone ClpB |
| RJ610_19620 | WND79482.1 | 1,45 | 1,49E-02 | | | OmpA family protein |
| RJ610_19625 | WND79483.1 | 1,06 | 8,17E-01 | | | lactoylglutathione lyase |
| RJ610_19630 | WND79484.1 | 0,68 | 2,79E-04 | | | hypothetical protein |
| RJ610_19635 | WND79485.1 | 0,78 | 9,66E-02 | | | hypothetical protein |
| RJ610_19640 | WND79486.1 | 0,69 | 2,74E-02 | | | DUF4166 domain-containing protein |
| RJ610_19645 | WND79487.1 | 0,71 | 1,37E-03 | | | hypothetical protein |
| RJ610_19650 | WND79488.1 | 0,83 | 1,82E-01 | | | peptidoglycan editing factor PgeF |
| RJ610_19655 | WND79489.1 | 0,72 | 4,02E-03 | | | 23S rRNA pseudouridine(1911/1915/1917) synthase RluD |
| RJ610_19660 | WND79490.1 | 0,58 | 3,21E-11 | | | outer membrane protein assembly factor BamD |
| RJ610_19665 | WND79491.1 | 0,55 | 6,03E-05 | | | NAD+ synthase |
| RJ610_19670 | WND79492.1 | 0,88 | 6,56E-01 | | | endonuclease domain-containing protein |
| RJ610_19675 | WND79493.1 | 0,75 | 8,49E-03 | | | succinate--CoA ligase subunit alpha |
| RJ610_19680 | WND79494.1 | 0,69 | 5,89E-05 | | | ADP-forming succinate--CoA ligase subunit beta |
| RJ610_19685 | WND79495.1 | 1,17 | 1,65E-01 | | | ATP-binding protein |
| RJ610_19690 | WND79496.1 | 1,53 | 4,69E-06 | | | sigma-54 dependent transcriptional regulator |
| RJ610_19695 | WND79497.1 | 2,95 | 3,79E-19 | | | prepilin-type N-terminal cleavage/methylation domain-containing protein |
| RJ610_19700 | WND79498.1 | 6,43 | 4,48E-57 | | | pilin |
| RJ610_19705 | WND79499.1 | 3,69 | 1,85E-46 | | | hypothetical protein |
| RJ610_19710 | WND79500.1 | 3,97 | 4,03E-38 | | | hypothetical protein |
| RJ610_19715 | WND79501.1 | 2,31 | 4,66E-10 | | | glycosyltransferase |
| RJ610_19720 | WND79502.1 | 2,25 | 2,46E-09 | | | hypothetical protein |
| RJ610_19725 | WND79503.1 | 2,20 | 2,69E-11 | | | class I SAM-dependent methyltransferase |
| RJ610_19730 | WND79504.1 | 2,26 | 6,01E-10 | | | glycosyltransferase family 2 protein |
| RJ610_19735 | WND79505.1 | 3,71 | 2,64E-29 | | | type IV-A pilus assembly ATPase PilB |

| | | | | | | |
|---|---|---|---|---|---|---|
| RJ610_19740 | WND79506.1 | 2,73 | 2,81E-23 | | | type II secretion system F family protein |
| RJ610_19745 | WND79507.1 | 1,81 | 9,42E-12 | | | A24 family peptidase |
| RJ610_19750 | WND79508.1 | 1,56 | 1,45E-05 | | | dephospho-CoA kinase |
| RJ610_19755 | WND83302.1 | 1,51 | 5,31E-04 | | | Nudix family hydrolase |
| RJ610_19760 | WND79509.1 | 1,38 | 3,38E-02 | | | preprotein translocase subunit SecA |
| RJ610_19765 | WND83303.1 | 2,67 | 8,70E-17 | | | M23 family metallopeptidase |
| RJ610_19770 | WND79510.1 | 2,12 | 4,30E-11 | | | DUF721 domain-containing protein |
| RJ610_19775 | WND79511.1 | 0,94 | 6,16E-01 | | | UDP-3-O-acyl-N-acetylglucosamine deacetylase |
| RJ610_19780 | WND79512.1 | 0,85 | 1,71E-01 | | | cell division protein FtsZ |
| RJ610_19785 | WND79513.1 | 0,72 | 5,23E-03 | | | cell division protein FtsA |
| RJ610_19790 | | 0,64 | 9,41E-04 | | | cell division protein FtsQ/DivIB |
| RJ610_19795 | WND79514.1 | 0,66 | 6,75E-03 | | | D-alanine--D-alanine ligase |
| RJ610_19800 | WND79515.1 | 0,64 | 9,71E-05 | | | UDP-N-acetylmuramate--L-alanine ligase |
| RJ610_19805 | WND79516.1 | 0,77 | 8,23E-02 | | | undecaprenyldiphospho-muramoylpentapeptide beta-N-acetylglucosaminyltransferase |
| RJ610_19810 | WND79517.1 | 0,59 | 6,23E-05 | | | putative lipid II flippase FtsW |
| RJ610_19815 | WND79518.1 | 0,54 | 5,18E-06 | | | phospho-N-acetylmuramoyl-pentapeptide-transferase |
| RJ610_19820 | WND79519.1 | 0,63 | 8,02E-05 | | | UDP-N-acetylmuramoyl-tripeptide--D-alanyl-D-alanine ligase |
| RJ610_19825 | WND83304.1 | 0,57 | 1,14E-03 | | | UDP-N-acetylmuramoyl-L-alanyl-D-glutamate--2,6-diaminopimelate ligase |
| RJ610_19830 | WND79520.1 | 0,56 | 1,52E-04 | | | penicillin-binding transpeptidase domain-containing protein |
| RJ610_19835 | WND79521.1 | 0,48 | 2,39E-05 | | | cell division protein FtsL |
| RJ610_19840 | WND79522.1 | 0,57 | 2,01E-05 | | | 16S rRNA (cytosine(1402)-N(4))-methyltransferase RsmH |
| RJ610_19845 | WND79523.1 | 0,58 | 2,14E-06 | | | division/cell wall cluster transcriptional repressor MraZ |
| RJ610_19850 | WND79524.1 | 0,40 | 2,97E-13 | | | ester cyclase |
| RJ610_19855 | | 1,32 | 1,65E-02 | | | RNase P RNA component class A |
| RJ610_19860 | WND79525.1 | 0,79 | 1,14E-01 | | | hypothetical protein |
| RJ610_19865 | WND79526.1 | 0,67 | 1,09E-04 | | | 16S rRNA (cytidine(1402)-2'-O)-methyltransferase |
| RJ610_19870 | WND79527.1 | 0,60 | 7,50E-05 | | | penicillin-binding protein activator |
| RJ610_19875 | WND79528.1 | 0,77 | 8,22E-02 | | | YraN family protein |
| RJ610_19880 | WND79529.1 | 0,84 | 1,92E-01 | | | metal-dependent hydrolase |
| RJ610_19885 | WND79530.1 | 0,70 | 5,10E-03 | | | FAD-linked oxidase C-terminal domain-containing protein |
| RJ610_19890 | WND79531.1 | 0,30 | 1,60E-12 | | | hypothetical protein |
| RJ610_19895 | WND79532.1 | 1,12 | 4,73E-01 | | | thiamine-phosphate kinase |
| RJ610_19900 | WND79533.1 | 0,97 | 8,03E-01 | | | transcription antitermination factor NusB |
| RJ610_19910 | WND79535.1 | 1,15 | 2,42E-01 | | | phytanoyl-CoA dioxygenase family protein |
| RJ610_19915 | WND79536.1 | 1,15 | 3,90E-01 | | | 3,4-dihydroxy-2-butanone-4-phosphate synthase |
| RJ610_19925 | WND79538.1 | 0,74 | 5,42E-02 | | | class I SAM-dependent methyltransferase |
| RJ610_19930 | WND79539.1 | 0,80 | 1,45E-01 | | | bifunctional diaminohydroxyphosphoribosylaminopyrimidine deaminase/5-amino-6-(5-phosphoribosylamino)uracil reductase RibD |
| RJ610_19935 | WND79540.1 | 0,72 | 4,53E-03 | | | hypothetical protein |
| RJ610_19940 | WND79541.1 | 0,94 | 6,68E-01 | | | hypothetical protein |

| | | | | | | |
|---|---|---|---|---|---|---|
| RJ610_19945 | WND79542.1 | 1,16 | 2,32E-01 | | | transcriptional regulator NrdR |
| RJ610_19950 | WND79543.1 | 1,27 | 4,13E-02 | | | energy transducer TonB |
| RJ610_19955 | WND79544.1 | 1,01 | 9,24E-01 | | | TonB family protein |
| RJ610_19965 | WND79546.1 | 1,09 | 5,08E-01 | | | lipid A hydroxylase LpxO |
| RJ610_19970 | WND79547.1 | 0,83 | 1,54E-01 | | | energy-dependent translational throttle protein EttA |
| RJ610_19975 | WND79548.1 | 1,01 | 9,64E-01 | | | hypothetical protein |
| RJ610_19980 | WND79549.1 | 3,22 | 3,48E-14 | | | type I methionyl aminopeptidase |
| RJ610_19990 | WND79551.1 | 2,38 | 4,79E-06 | | | hypothetical protein |
| RJ610_19995 | WND79552.1 | 1,36 | 1,28E-01 | | | hypothetical protein |
| RJ610_20000 | WND79553.1 | 0,71 | 3,00E-02 | | | MBL fold metallo-hydrolase |
| RJ610_20005 | WND79554.1 | 0,51 | 1,08E-06 | | | glycosyltransferase family 2 protein |
| RJ610_20010 | | 0,52 | 4,96E-10 | | | glycosyltransferase |
| RJ610_20020 | WND79556.1 | 1,45 | 2,60E-03 | | | hypothetical protein |
| RJ610_20025 | WND83305.1 | 1,31 | 3,22E-02 | | | type II secretion system protein GspM |
| RJ610_20030 | WND79557.1 | 1,41 | 3,89E-04 | | | PilN domain-containing protein |
| RJ610_20035 | WND79558.1 | 1,48 | 4,60E-05 | | | type II secretion system protein GspK |
| RJ610_20040 | WND79559.1 | 1,42 | 8,32E-03 | | | prepilin-type N-terminal cleavage/methylation domain-containing protein |
| RJ610_20045 | WND79560.1 | 1,27 | 1,43E-01 | | | prepilin-type N-terminal cleavage/methylation domain-containing protein |
| RJ610_20050 | WND79561.1 | 1,31 | 2,32E-02 | | | GspH/FimT family pseudopilin |
| RJ610_20055 | WND79562.1 | 1,16 | 2,55E-01 | | | type II secretion system major pseudopilin GspG |
| RJ610_20060 | WND79563.1 | 1,48 | 4,49E-05 | | | type II secretion system F family protein |
| RJ610_20065 | WND83306.1 | 1,47 | 2,14E-05 | | | type II secretion system ATPase GspE |
| RJ610_20075 | WND79565.1 | 0,83 | 4,22E-02 | | | phosphoribosylformylglycinamidine synthase |
| RJ610_20080 | WND79566.1 | 2,61 | 7,59E-09 | | | hypothetical protein |
| RJ610_20085 | WND79567.1 | 7,11 | 1,39E-64 | | | DsbC family protein |
| RJ610_20090 | WND79568.1 | 0,83 | 2,29E-01 | | | site-specific tyrosine recombinase XerD |
| RJ610_20095 | WND79569.1 | 0,79 | 9,61E-02 | | | RDD family protein |
| RJ610_20100 | WND79570.1 | 0,56 | 5,41E-06 | | | LPS export ABC transporter permease LptG |
| RJ610_20105 | WND79571.1 | 0,64 | 7,49E-05 | | | LPS export ABC transporter permease LptF |
| RJ610_20115 | WND79573.1 | 1,14 | 3,68E-01 | | | DNA polymerase III subunit chi |
| RJ610_20120 | WND79574.1 | 1,08 | 5,33E-01 | | | valine--tRNA ligase |
| RJ610_20125 | WND79575.1 | 1,23 | 1,89E-01 | | | hypothetical protein |
| RJ610_20130 | WND79576.1 | 3,12 | 1,20E-10 | | | ankyrin repeat domain-containing protein |
| RJ610_20140 | WND79577.1 | 1,06 | 6,71E-01 | | | ribosomal protein S18-alanine N-acetyltransferase |
| RJ610_20145 | WND79578.1 | 0,84 | 1,78E-01 | | | alanine acetyltransferase |
| RJ610_20150 | WND79579.1 | 0,77 | 3,15E-02 | | | CDP-diacylglycerol--serine O-phosphatidyltransferase |
| RJ610_20155 | WND83308.1 | 2,51 | 1,89E-12 | | | DUF4124 domain-containing protein |
| RJ610_20160 | WND79580.1 | 0,90 | 3,33E-01 | | | proline--tRNA ligase |
| RJ610_20165 | WND79581.1 | 0,77 | 6,63E-02 | | | H-NS histone family protein |
| RJ610_20170 | WND79582.1 | 0,72 | 3,74E-02 | | | threonine/serine exporter family protein |
| RJ610_20175 | WND79583.1 | 0,55 | 7,75E-08 | | | MlaE family lipid ABC transporter permease subunit |

| | | | | | | |
|---|---|---|---|---|---|---|
| RJ610_20180 | WND79584.1 | 0,74 | 4,29E-02 | | | ABC transporter ATP-binding protein |
| RJ610_20185 | WND79585.1 | 0,80 | 1,65E-01 | | | CPBP family intramembrane glutamic endopeptidase |
| RJ610_20190 | WND79586.1 | 0,64 | 1,39E-04 | | | MlaD family protein |
| RJ610_20195 | WND79587.1 | 0,88 | 4,47E-01 | | | ABC-type transport auxiliary lipoprotein family protein |
| RJ610_20205 | WND79589.1 | 4,92 | 2,49E-12 | | | alpha-ketoglutarate-dependent dioxygenase AlkB |
| RJ610_20210 | WND79590.1 | 1,42 | 1,15E-04 | | | electron transfer flavoprotein-ubiquinone oxidoreductase |
| RJ610_20225 | WND79593.1 | 0,94 | 7,02E-01 | | | CoA transferase subunit A |
| RJ610_20230 | WND79594.1 | 0,99 | 9,46E-01 | | | hypothetical protein |
| RJ610_20235 | WND79595.1 | 0,91 | 4,32E-01 | | | mannose-1-phosphate guanylyltransferase/mannose-6-phosphate isomerase |
| RJ610_20240 | WND79596.1 | 3,33 | 3,09E-31 | | | D-hexose-6-phosphate mutarotase |
| RJ610_20245 | WND79597.1 | 4,94 | 2,85E-47 | | | hypothetical protein |
| RJ610_20250 | WND79598.1 | 1,04 | 8,12E-01 | | | electron transfer flavoprotein subunit beta/FixA family protein |
| RJ610_20255 | WND79599.1 | 1,21 | 1,39E-01 | | | electron transfer flavoprotein subunit alpha/FixB family protein |
| RJ610_20260 | WND79600.1 | 0,68 | 9,44E-05 | | | NAD-dependent epimerase/dehydratase family protein |
| RJ610_20265 | WND79601.1 | 0,59 | 1,10E-06 | | | glycosyltransferase |
| RJ610_20270 | WND79602.1 | 0,81 | 7,75E-02 | | | GtrA family protein |
| RJ610_20275 | WND79603.1 | 0,68 | 4,70E-04 | | | NAD(P)/FAD-dependent oxidoreductase |
| RJ610_20280 | WND79604.1 | 0,70 | 5,29E-04 | | | hypothetical protein |
| RJ610_20285 | WND79605.1 | 1,67 | 1,53E-05 | | | class I SAM-dependent methyltransferase |
| RJ610_20290 | WND79606.1 | 2,02 | 4,09E-12 | | | glycosyltransferase |
| RJ610_20295 | WND79607.1 | 2,36 | 4,73E-12 | | | GDP-mannose 4,6-dehydratase |
| RJ610_20300 | WND79608.1 | 3,71 | 2,01E-26 | | | GDP-mannose 4,6-dehydratase |
| RJ610_20305 | WND79609.1 | 1,63 | 1,25E-05 | | | methyltransferase domain-containing protein |
| RJ610_20310 | WND79610.1 | 1,11 | 3,51E-01 | | | ABC transporter ATP-binding protein |
| RJ610_20315 | WND79611.1 | 1,05 | 7,08E-01 | | | ABC transporter permease |
| RJ610_20320 | WND79612.1 | 0,92 | 4,66E-01 | | | glycosyltransferase family 4 protein |
| RJ610_20335 | WND79615.1 | 0,89 | 3,40E-01 | | | YdcH family protein |
| RJ610_20340 | WND79616.1 | 0,82 | 1,35E-01 | | | hypothetical protein |
| RJ610_20345 | WND79617.1 | 0,73 | 2,81E-03 | | | DUF4398 domain-containing protein |
| RJ610_20350 | WND79618.1 | 4,62 | 1,14E-50 | | | PilT/PilU family type 4a pilus ATPase |
| RJ610_20355 | WND79619.1 | 1,07 | 7,37E-01 | | | maleylacetoacetate isomerase |
| RJ610_20360 | WND79620.1 | 1,31 | 1,96E-01 | | | fumarylacetoacetate hydrolase family protein |
| RJ610_20370 | WND79622.1 | 1,66 | 8,50E-03 | | | tetratricopeptide repeat protein |
| RJ610_20375 | WND79623.1 | 2,91 | 2,43E-11 | | | ECF-type sigma factor |
| RJ610_20380 | WND79624.1 | 1,08 | 5,88E-01 | | | GNAT family N-acetyltransferase |
| RJ610_20385 | | 0,75 | 2,34E-02 | | | tRNA-Gln |
| RJ610_20390 | WND79625.1 | 0,68 | 1,43E-03 | | | 30S ribosomal protein S9 |
| RJ610_20400 | WND79627.1 | 1,00 | 9,77E-01 | | | (Fe-S)-binding protein |
| RJ610_20405 | WND79628.1 | 1,23 | 3,56E-01 | | | 2-polyprenyl-3-methyl-6-methoxy-1,4-benzoquinone monooxygenase |
| RJ610_20410 | WND79629.1 | 0,91 | 3,94E-01 | | | adenosylmethionine decarboxylase |
| RJ610_20415 | WND79630.1 | 3,86 | 2,77E-27 | | | cAMP-activated global transcriptional regulator CRP |

| | | | | | | |
|---|---|---|---|---|---|---|
| RJ610_20420 | WND79631.1 | 5,67 | 9,32E-56 | | | global regulator CLP-associated N-acetyltransferase |
| RJ610_20425 | WND79632.1 | 1,56 | 1,71E-03 | | | haloacid dehalogenase-like hydrolase |
| RJ610_20430 | WND79633.1 | 0,95 | 6,95E-01 | | | indole-3-glycerol phosphate synthase TrpC |
| RJ610_20435 | WND79634.1 | 0,68 | 2,99E-04 | | | Mur ligase family protein |
| RJ610_20440 | WND79635.1 | 0,69 | 8,49E-04 | | | cyanophycin synthetase |
| RJ610_20445 | WND79636.1 | 0,60 | 1,07E-07 | | | cyanophycinase |
| RJ610_20455 | WND79638.1 | 0,84 | 3,67E-01 | | | antibiotic biosynthesis monooxygenase |
| RJ610_20460 | WND79639.1 | 0,66 | 3,26E-04 | | | anthranilate phosphoribosyltransferase |
| RJ610_20465 | WND79640.1 | 0,58 | 9,63E-07 | | | aminodeoxychorismate/anthranilate synthase component II |
| RJ610_20470 | WND79641.1 | 1,11 | 5,99E-01 | | | HAMP domain-containing sensor histidine kinase |
| RJ610_20475 | WND79642.1 | 0,97 | 8,54E-01 | | | response regulator transcription factor |
| RJ610_20480 | WND79643.1 | 0,64 | 8,93E-02 | | | hypothetical protein |
| RJ610_20485 | WND79644.1 | 0,65 | 1,16E-03 | | | FG-GAP-like repeat-containing protein |
| RJ610_20490 | WND79645.1 | 0,86 | 1,30E-01 | | | anthranilate synthase component I |
| RJ610_20495 | WND79646.1 | 1,10 | 5,35E-01 | | | lipid kinase YegS |
| RJ610_20500 | WND79647.1 | 0,95 | 6,79E-01 | | | ribonuclease E activity regulator RraA |
| RJ610_20510 | WND79649.1 | 0,75 | 7,69E-02 | | | DnaJ domain-containing protein |
| RJ610_20515 | WND79650.1 | 0,84 | 1,22E-01 | | | phosphoribosylaminoimidazolesuccinocarboxamide synthase |
| RJ610_20520 | WND79651.1 | 0,42 | 1,55E-08 | | | multidrug efflux SMR transporter |
| RJ610_20525 | WND79652.1 | 0,45 | 6,46E-09 | | | DUF962 domain-containing protein |
| RJ610_20530 | WND79653.1 | 0,53 | 1,49E-04 | | | hypothetical protein |
| RJ610_20535 | WND79654.1 | 0,60 | 1,82E-03 | | | EamA family transporter |
| RJ610_20540 | WND79655.1 | 0,90 | 3,70E-01 | | | nucleoside-diphosphate sugar epimerase |
| RJ610_20545 | WND79656.1 | 1,02 | 8,44E-01 | | | Na+/H+ antiporter NhaA |
| RJ610_20550 | WND79657.1 | 0,57 | 3,20E-05 | | | sodium:calcium antiporter |
| RJ610_20555 | WND79658.1 | 0,43 | 1,59E-06 | | | lectin |
| RJ610_20565 | WND79659.1 | 1,40 | 5,48E-01 | | | hypothetical protein |
| RJ610_20580 | WND79662.1 | 1,02 | 8,70E-01 | | | oligopeptide:H+ symporter |
| RJ610_20585 | WND79663.1 | 0,93 | 5,17E-01 | | | tryptophan 2,3-dioxygenase family protein |
| RJ610_20590 | WND79664.1 | 0,65 | 8,85E-04 | | | lamin tail domain-containing protein |
| RJ610_20600 | WND79666.1 | 1,33 | 1,33E-02 | | | pyruvate dehydrogenase (acetyl-transferring) E1 component subunit alpha |
| RJ610_20605 | WND79667.1 | 1,45 | 3,84E-03 | | | alpha-ketoacid dehydrogenase subunit beta |
| RJ610_20610 | WND83310.1 | 1,45 | 2,62E-03 | | | SH3 domain-containing protein |
| RJ610_20615 | WND79668.1 | 1,42 | 3,13E-03 | | | dihydrolipoamide acetyltransferase family protein |
| RJ610_20620 | WND79669.1 | 6,31 | 1,11E-22 | | | peptidoglycan DD-metalloendopeptidase family protein |
| RJ610_20630 | WND79671.1 | 1,96 | 1,61E-05 | | | HEAT repeat domain-containing protein |
| RJ610_20635 | WND79672.1 | 4,47 | 1,84E-31 | | | VOC family protein |
| RJ610_20640 | WND79673.1 | 3,02 | 9,38E-19 | | | sensor domain-containing diguanylate cyclase |
| RJ610_20645 | WND79674.1 | 0,90 | 5,70E-01 | | | MBL fold metallo-hydrolase |
| RJ610_20650 | WND79675.1 | 0,96 | 8,61E-01 | | | hypothetical protein |
| RJ610_20660 | WND79677.1 | 0,88 | 4,84E-01 | | | hypothetical protein |
| RJ610_20665 | WND79678.1 | 3,86 | 6,69E-19 | | | CsbD family protein |

| | | | | | | |
|---|---|---|---|---|---|---|
| RJ610_20670 | WND79679.1 | 0,20 | 2,90E-19 | | | entericidin A/B family lipoprotein |
| RJ610_20680 | | 1,38 | 5,56E-02 | | | tRNA-Thr |
| RJ610_20685 | WND79681.1 | 0,95 | 7,50E-01 | | | SPOR domain-containing protein |
| RJ610_20690 | WND79682.1 | 0,65 | 9,66E-03 | | | type III pantothenate kinase |
| RJ610_20695 | WND79683.1 | 0,51 | 8,72E-06 | | | bifunctional biotin--[acetyl-CoA-carboxylase] ligase/biotin operon repressor BirA |
| RJ610_20700 | WND79684.1 | 0,54 | 3,62E-06 | | | glycerol-3-phosphate 1-O-acyltransferase PlsY |
| RJ610_20705 | WND79685.1 | 1,23 | 2,62E-01 | | | DegV family protein |
| RJ610_20710 | WND79686.1 | 1,10 | 4,40E-01 | | | hypothetical protein |
| RJ610_20715 | WND79687.1 | 0,82 | 1,15E-01 | | | zinc-dependent peptidase |
| RJ610_20720 | WND79688.1 | 0,76 | 3,23E-03 | | | ATP-binding protein |
| RJ610_20725 | WND79689.1 | 0,70 | 5,67E-03 | | | response regulator transcription factor |
| RJ610_20730 | WND79690.1 | 1,34 | 2,33E-02 | | | hypothetical protein |
| RJ610_20735 | WND79691.1 | 0,98 | 8,52E-01 | | | arginine deiminase-related protein |
| RJ610_20740 | WND79692.1 | 1,37 | 6,65E-04 | | | tRNA dihydrouridine(20/20a) synthase DusA |
| RJ610_20745 | WND79693.1 | 1,84 | 4,45E-06 | | | hypothetical protein |
| RJ610_20750 | WND79694.1 | 1,40 | 3,54E-02 | | | CatB-related O-acetyltransferase |
| RJ610_20755 | WND79695.1 | 0,85 | 3,82E-01 | | | RNA 3'-terminal phosphate cyclase |
| RJ610_20760 | WND79696.1 | 1,07 | 6,03E-01 | | | RtcB family protein |
| RJ610_20765 | WND79697.1 | 0,41 | 7,24E-06 | | | TROVE domain-containing protein |
| RJ610_20770 | WND79698.1 | 1,22 | 2,43E-01 | | | RNA repair transcriptional activator RtcR |
| RJ610_20775 | WND79699.1 | 7,81 | 3,68E-34 | | | YcaO-like family protein |
| RJ610_20780 | WND79700.1 | 19,57 | 1,81E-55 | | | hypothetical protein |
| RJ610_20785 | WND79701.1 | 0,94 | 6,04E-01 | | | penicillin-binding transpeptidase domain-containing protein |
| RJ610_20790 | WND79702.1 | 0,66 | 8,88E-03 | | | ABC transporter ATP-binding protein/permease |
| RJ610_20795 | WND79703.1 | 2,34 | 2,08E-06 | | | GNAT family N-acetyltransferase |
| RJ610_20800 | WND79704.1 | 1,07 | 7,88E-01 | | | hypothetical protein |
| RJ610_20805 | WND83311.1 | 0,68 | 1,69E-02 | | | hypothetical protein |
| RJ610_20810 | WND79705.1 | 0,85 | 4,03E-01 | | | hypothetical protein |
| RJ610_20815 | WND79706.1 | 1,34 | 2,03E-02 | | | HAD family hydrolase |
| RJ610_20820 | WND79707.1 | 2,52 | 3,79E-06 | | | EF-hand domain-containing protein |
| RJ610_20825 | WND79708.1 | 3,77 | 7,42E-23 | | | winged helix DNA-binding domain-containing protein |
| RJ610_20830 | WND79709.1 | 3,50 | 3,50E-13 | | | CPXCG motif-containing cysteine-rich protein |
| RJ610_20835 | WND79710.1 | 4,09 | 5,76E-10 | | | hypothetical protein |
| RJ610_20840 | WND79711.1 | 712,55 | 1,65E-254 | | | PQQ-dependent sugar dehydrogenase |
| RJ610_20845 | WND83312.1 | 2,95 | 1,71E-08 | | | lytic transglycosylase domain-containing protein |
| RJ610_20850 | WND79712.1 | 2,99 | 7,69E-03 | | | hypothetical protein |
| RJ610_20855 | WND83313.1 | 0,87 | 4,34E-01 | | | APC family permease |
| RJ610_20860 | WND79713.1 | 1,32 | 3,45E-01 | | | NAD(P)H-dependent oxidoreductase |
| RJ610_20865 | WND79714.1 | 2,08 | 2,60E-03 | | | RidA family protein |
| RJ610_20870 | WND79715.1 | 1,14 | 3,57E-01 | | | N-acetylmuramoyl-L-alanine amidase |
| RJ610_20875 | WND79716.1 | 2,93 | 3,42E-12 | | | hypothetical protein |
| RJ610_20880 | WND79717.1 | 0,58 | 1,34E-06 | | | hypothetical protein |

| | | | | | | |
|---|---|---|---|---|---|---|
| RJ610_20885 | WND79718.1 | 0,77 | 1,33E-01 | | | hypothetical protein |
| RJ610_20890 | WND79719.1 | 0,89 | 5,17E-01 | | | hypothetical protein |
| RJ610_20900 | WND79721.1 | 1,56 | 8,54E-04 | | | heparan-alpha-glucosaminide N-acetyltransferase domain-containing protein |
| RJ610_20910 | WND79723.1 | 1,09 | 6,33E-01 | | | hypothetical protein |
| RJ610_20915 | WND79724.1 | 0,66 | 6,86E-03 | | | alpha/beta hydrolase |
| RJ610_20920 | WND79725.1 | 1,05 | 8,38E-01 | | | YciI family protein |
| RJ610_20925 | WND83314.1 | 0,76 | 2,25E-02 | | | DUF1615 domain-containing protein |
| RJ610_20935 | WND79727.1 | 0,73 | 2,56E-01 | | | hypothetical protein |
| RJ610_20940 | WND83315.1 | 0,81 | 8,56E-02 | | | aldo/keto reductase |
| RJ610_20945 | WND79728.1 | 1,02 | 9,12E-01 | | | HD domain-containing protein |
| RJ610_20950 | WND79729.1 | 1,43 | 2,47E-01 | | | RidA family protein |
| RJ610_20955 | WND79730.1 | 1,95 | 6,48E-02 | | | rhodanese-like domain-containing protein |
| RJ610_20960 | WND79731.1 | 1,16 | 5,04E-01 | | | transcriptional regulator FtrA |
| RJ610_20965 | WND79732.1 | 4,66 | 1,96E-23 | | | HAD-IA family hydrolase |
| RJ610_20970 | WND79733.1 | 4,32 | 7,34E-24 | | | GNAT family N-acetyltransferase |
| RJ610_20975 | WND79734.1 | 36,04 | 9,63E-56 | | | alpha/beta hydrolase |
| RJ610_20980 | WND79735.1 | 28,09 | 4,79E-31 | | | hypothetical protein |
| RJ610_20985 | WND79736.1 | 3,13 | 5,21E-07 | | | pentapeptide repeat-containing protein |
| RJ610_20990 | WND79737.1 | 0,67 | 1,01E-02 | | | hypothetical protein |
| RJ610_20995 | WND79738.1 | 0,89 | 4,70E-01 | | | amidohydrolase family protein |
| RJ610_21000 | WND79739.1 | 1,24 | 1,69E-01 | | | hypothetical protein |
| RJ610_21005 | WND79740.1 | 1,33 | 6,29E-02 | | | Sir2 family NAD-dependent protein deacetylase |
| RJ610_21010 | WND79741.1 | 0,96 | 7,51E-01 | | | CshA/CshB family fibrillar adhesin-related protein |
| RJ610_21015 | WND79742.1 | 0,73 | 3,72E-02 | | | hypothetical protein |
| RJ610_21020 | WND79743.1 | 1,06 | 7,78E-01 | | | SRPBCC family protein |
| RJ610_21025 | WND79744.1 | 1,11 | 4,55E-01 | | | sterol desaturase family protein |
| RJ610_21030 | WND79745.1 | 1,18 | 3,35E-01 | | | glutathione S-transferase |
| RJ610_21035 | WND79746.1 | 0,94 | 8,19E-01 | | | M20/M25/M40 family metallo-hydrolase |
| RJ610_21040 | WND79747.1 | 0,70 | 3,61E-02 | | | Lrp/AsnC family transcriptional regulator |
| RJ610_21045 | WND79748.1 | 0,64 | 5,50E-03 | | | pyridoxal-phosphate dependent enzyme |
| RJ610_21050 | WND79749.1 | 1,23 | 1,17E-01 | | | LysR substrate-binding domain-containing protein |
| RJ610_21055 | WND79750.1 | 1,32 | 2,01E-02 | | | glutathione S-transferase family protein |
| RJ610_21060 | WND79751.1 | 0,95 | 7,23E-01 | | | acyl-CoA desaturase |
| RJ610_21065 | WND79752.1 | 0,91 | 5,14E-01 | | | ferredoxin reductase |
| RJ610_21070 | WND83316.1 | 0,70 | 2,87E-02 | | | HTH-type transcriptional repressor FabR |
| RJ610_21075 | WND79753.1 | 0,82 | 7,06E-02 | | | hypothetical protein |
| RJ610_21080 | WND79754.1 | 0,90 | 3,90E-01 | | | STM3941 family protein |
| RJ610_21085 | WND79755.1 | 1,56 | 3,59E-04 | | | peptidoglycan-binding protein |
| RJ610_21090 | WND79756.1 | 1,31 | 2,25E-02 | | | hypothetical protein |
| RJ610_21095 | WND79757.1 | 1,47 | 2,50E-02 | | | alkaline phosphatase |
| RJ610_21100 | WND79758.1 | 1,22 | 4,62E-01 | | | hypothetical protein |
| RJ610_21105 | WND79759.1 | 0,94 | 8,09E-01 | | | hypothetical protein |

| | | | | | | |
|---|---|---|---|---|---|---|
| RJ610_21110 | WND79760.1 | 0,76 | 8,36E-02 | | | YcaO-like family protein |
| RJ610_21115 | WND79761.1 | 0,46 | 2,98E-06 | | | hypothetical protein |
| RJ610_21120 | WND79762.1 | 0,18 | 1,45E-67 | | | hypothetical protein |
| RJ610_21125 | WND79763.1 | 0,09 | 2,26E-56 | | | hypothetical protein |
| RJ610_21130 | WND79764.1 | 0,08 | 1,11E-39 | | | energy transducer TonB |
| RJ610_21135 | WND79765.1 | 0,58 | 2,18E-03 | | | hypothetical protein |
| RJ610_21140 | WND79766.1 | 0,87 | 3,45E-01 | | | hypothetical protein |
| RJ610_21145 | WND79767.1 | 1,12 | 4,26E-01 | | | hypothetical protein |
| RJ610_21150 | WND79768.1 | 0,93 | 6,00E-01 | | | VOC family protein |
| RJ610_21155 | WND79769.1 | 1,01 | 9,38E-01 | | | hypothetical protein |
| RJ610_21160 | WND79770.1 | 1,00 | 9,89E-01 | | | hypothetical protein |
| RJ610_21170 | WND79772.1 | 1,02 | 9,22E-01 | | | LysR family transcriptional regulator |
| RJ610_21175 | WND79773.1 | 1,39 | 1,99E-01 | | | MFS transporter |
| RJ610_21180 | WND79774.1 | 0,89 | 7,57E-01 | | | hypothetical protein |
| RJ610_21185 | WND79775.1 | 1,87 | 1,66E-07 | | | class A beta-lactamase |
| RJ610_21190 | WND79776.1 | 0,65 | 4,40E-03 | | | NHL repeat-containing protein |
| RJ610_21195 | WND79777.1 | 0,48 | 5,14E-05 | | | PA2169 family four-helix-bundle protein |
| RJ610_21200 | WND79778.1 | 0,73 | 5,62E-02 | | | ligase-associated DNA damage response exonuclease |
| RJ610_21205 | WND79779.1 | 0,94 | 7,43E-01 | | | ATP-dependent DNA ligase |
| RJ610_21210 | WND79780.1 | 0,74 | 3,56E-03 | | | excinuclease ABC subunit UvrA |
| RJ610_21215 | WND79781.1 | 0,65 | 3,23E-02 | | | response regulator |
| RJ610_21220 | WND79782.1 | 0,62 | 8,40E-04 | | | response regulator |
| RJ610_21225 | WND79783.1 | 0,61 | 7,37E-04 | | | ATP-binding protein |
| RJ610_21230 | WND83317.1 | 0,63 | 1,45E-01 | | | DNA-formamidopyrimidine glycosylase family protein |
| RJ610_21235 | WND79784.1 | 0,88 | 6,36E-01 | | | DUF72 domain-containing protein |
| RJ610_21240 | WND79785.1 | 0,69 | 6,24E-02 | | | NAD(P)/FAD-dependent oxidoreductase |
| RJ610_21245 | WND79786.1 | 0,97 | 8,96E-01 | | | hypothetical protein |
| RJ610_21250 | WND79787.1 | 0,85 | 4,73E-01 | | | serine hydrolase domain-containing protein |
| RJ610_21255 | WND79788.1 | 0,81 | 3,78E-01 | | | sigma-70 family RNA polymerase sigma factor |
| RJ610_21260 | WND79789.1 | 0,63 | 7,61E-04 | | | hypothetical protein |
| RJ610_21270 | WND79791.1 | 3,57 | 3,57E-38 | | | aldehyde oxidoreductase molybdenum-binding subunit PaoC |
| RJ610_21275 | WND79792.1 | 3,87 | 1,93E-36 | | | xanthine dehydrogenase family protein subunit M |
| RJ610_21280 | WND79793.1 | 6,93 | 7,01E-58 | | | aldehyde dehydrogenase iron-sulfur subunit PaoA |
| RJ610_21285 | WND79794.1 | 1,66 | 2,46E-03 | | | hypothetical protein |
| RJ610_21290 | WND79795.1 | 2,55 | 6,31E-15 | | | GFA family protein |
| RJ610_21295 | WND79796.1 | 0,82 | 6,91E-02 | | | L,D-transpeptidase |
| RJ610_21300 | WND79797.1 | 0,80 | 7,79E-02 | | | M23 family metallopeptidase |
| RJ610_21305 | WND79798.1 | 0,80 | 1,11E-01 | | | PAS domain S-box protein |
| RJ610_21310 | WND79799.1 | 0,50 | 3,14E-08 | | | NrdJb |
| RJ610_21315 | WND79800.1 | 0,45 | 8,85E-09 | | | hypothetical protein |
| RJ610_21320 | WND79801.1 | 0,58 | 9,98E-06 | | | adenosylcobalamin-dependent ribonucleoside-diphosphate reductase |
| RJ610_21330 | WND79803.1 | 0,63 | 5,16E-03 | | | hypothetical protein |

| | | | | | | |
|---|---|---|---|---|---|---|
| RJ610_21335 | WND83318.1 | 0,73 | 6,62E-02 | | | phage holin family protein |
| RJ610_21340 | WND79804.1 | 0,97 | 8,69E-01 | | | hypothetical protein |
| RJ610_21345 | WND83319.1 | 0,39 | 3,29E-13 | | | AI-2E family transporter |
| RJ610_21350 | WND79805.1 | 0,55 | 4,63E-06 | | | endonuclease V |
| RJ610_21355 | WND79806.1 | 1,19 | 2,19E-01 | | | HAD-IA family hydrolase |
| RJ610_21365 | WND79808.1 | 1,21 | 8,26E-02 | | | nucleoside 2-deoxyribosyltransferase domain-containing protein |
| RJ610_21370 | WND79809.1 | 0,01 | 6,37E-268 | | | hypothetical protein |
| RJ610_21375 | WND79810.1 | 11,52 | 3,04E-76 | | | GEVED domain-containing protein |
| RJ610_21380 | WND79811.1 | 0,39 | 5,98E-23 | | | efflux RND transporter periplasmic adaptor subunit |
| RJ610_21385 | WND79812.1 | 0,64 | 2,04E-04 | | | ABC transporter ATP-binding protein |
| RJ610_21390 | WND79813.1 | 0,74 | 2,48E-02 | | | ABC transporter permease |
| RJ610_21395 | WND79814.1 | 0,89 | 3,93E-01 | | | FtsX-like permease family protein |
| RJ610_21400 | WND79815.1 | 1,39 | 6,44E-03 | | | hypothetical protein |
| RJ610_21405 | WND79816.1 | 1,34 | 1,03E-01 | | | EamA family transporter RarD |
| RJ610_21410 | WND79817.1 | 1,35 | 1,10E-01 | | | drug/metabolite exporter YedA |
| RJ610_21415 | WND79818.1 | 1,28 | 1,05E-01 | | | GNAT family N-acetyltransferase |
| RJ610_21420 | WND79819.1 | 1,28 | 2,12E-02 | | | Lrp/AsnC family transcriptional regulator |
| RJ610_21425 | WND79820.1 | 1,26 | 5,64E-02 | | | hypothetical protein |
| RJ610_21430 | WND79821.1 | 0,86 | 2,92E-01 | | | methylated-DNA--[protein]-cysteine S-methyltransferase |
| RJ610_21435 | WND79822.1 | 1,03 | 7,70E-01 | | | carboxy terminal-processing peptidase |
| RJ610_21440 | WND79823.1 | 0,85 | 3,47E-01 | | | lipoyl synthase |
| RJ610_21445 | WND83320.1 | 0,83 | 4,48E-01 | | | lipoyl(octanoyl) transferase LipB |
| RJ610_21450 | WND79824.1 | 0,84 | 4,91E-01 | | | DUF493 family protein |
| RJ610_21455 | WND79825.1 | 0,76 | 1,13E-01 | | | HAD family hydrolase |
| RJ610_21460 | WND79826.1 | 0,44 | 1,62E-16 | | | hypothetical protein |
| RJ610_21465 | WND79827.1 | 0,36 | 4,48E-05 | | | hypothetical protein |
| RJ610_21470 | WND79828.1 | 0,76 | 4,78E-02 | | | hypothetical protein |
| RJ610_21475 | WND79829.1 | 1,14 | 2,41E-01 | | | D-alanyl-D-alanine carboxypeptidase family protein |
| RJ610_21480 | WND79830.1 | 1,09 | 4,87E-01 | | | septal ring lytic transglycosylase RlpA family protein |
| RJ610_21485 | | 0,86 | 1,31E-01 | | | lytic murein transglycosylase B |
| RJ610_21490 | WND83321.1 | 0,64 | 2,77E-04 | | | type II toxin-antitoxin system PemK/MazF family toxin |
| RJ610_21495 | | 0,73 | 9,35E-02 | | | DUF4287 domain-containing protein |
| RJ610_21500 | WND79831.1 | 1,09 | 6,34E-01 | | | hypothetical protein |
| RJ610_21505 | WND79832.1 | 0,90 | 6,36E-01 | | | hypothetical protein |
| RJ610_21510 | WND79833.1 | 0,95 | 8,08E-01 | | | hypothetical protein |
| RJ610_21515 | WND79834.1 | 1,00 | 9,82E-01 | | | CPCC family cysteine-rich protein |
| RJ610_21520 | WND79835.1 | 1,08 | 5,86E-01 | | | hypothetical protein |
| RJ610_21525 | WND79836.1 | 1,29 | 5,05E-01 | | | isochorismatase family protein |
| RJ610_21530 | WND79837.1 | 0,95 | 7,79E-01 | | | LysR family transcriptional regulator |
| RJ610_21535 | WND79838.1 | 0,90 | 6,04E-01 | | | hypothetical protein |
| RJ610_21540 | WND79839.1 | 1,05 | 7,72E-01 | | | alpha/beta hydrolase |
| RJ610_21545 | WND79840.1 | 1,57 | 6,17E-05 | | | ankyrin repeat domain-containing protein |
| RJ610_21550 | WND79841.1 | 1,74 | 1,09E-04 | | | VOC family protein |

| | | | | | | |
|---|---|---|---|---|---|---|
| RJ610_21555 | WND83322.1 | 1,66 | 2,04E-06 | | | VOC family protein |
| RJ610_21560 | WND79842.1 | 2,05 | 5,18E-09 | | | hypothetical protein |
| RJ610_21565 | WND79843.1 | 1,58 | 4,28E-04 | | | hypothetical protein |
| RJ610_21570 | WND79844.1 | 1,42 | 2,05E-02 | | | ribonuclease E inhibitor RraB |
| RJ610_21575 | WND79845.1 | 1,82 | 2,03E-07 | | | hypothetical protein |
| RJ610_21580 | WND79846.1 | 1,19 | 1,70E-01 | | | hypothetical protein |
| RJ610_21585 | WND79847.1 | 1,04 | 7,57E-01 | | | hypothetical protein |
| RJ610_21595 | WND79849.1 | 0,81 | 1,90E-01 | | | hypothetical protein |
| RJ610_21600 | WND79850.1 | 0,71 | 2,54E-02 | | | DUF4304 domain-containing protein |
| RJ610_21605 | WND79851.1 | 0,75 | 4,51E-02 | | | hypothetical protein |
| RJ610_21610 | WND79852.1 | 0,74 | 3,72E-02 | | | hypothetical protein |
| RJ610_21615 | WND79853.1 | 1,02 | 8,75E-01 | | | hypothetical protein |
| RJ610_21620 | WND79854.1 | 0,86 | 3,50E-01 | | | hypothetical protein |
| RJ610_21625 | WND79855.1 | 0,78 | 5,28E-02 | | | rod shape-determining protein RodA |
| RJ610_21630 | WND79856.1 | 0,63 | 2,89E-06 | | | penicillin-binding protein 2 |
| RJ610_21635 | WND79857.1 | 0,49 | 1,81E-06 | | | rod shape-determining protein MreD |
| RJ610_21640 | WND79858.1 | 0,56 | 1,26E-08 | | | rod shape-determining protein MreC |
| RJ610_21645 | WND79859.1 | 0,55 | 2,29E-09 | | | rod shape-determining protein |
| RJ610_21650 | WND79860.1 | 0,82 | 8,45E-02 | | | carbohydrate kinase family protein |
| RJ610_21655 | WND79861.1 | 0,98 | 8,89E-01 | | | hypothetical protein |
| RJ610_21660 | WND79862.1 | 1,42 | 2,06E-02 | | | GH25 family lysozyme |
| RJ610_21665 | WND79863.1 | 1,35 | 4,02E-02 | | | hypothetical protein |
| RJ610_21670 | WND79864.1 | 1,31 | 1,00E-01 | | | alpha/beta hydrolase |
| RJ610_21675 | WND79865.1 | 1,22 | 1,56E-01 | | | M1 family metallopeptidase |
| RJ610_21685 | WND79867.1 | 2,92 | 5,61E-13 | | | bifunctional demethylmenaquinone methyltransferase/2-methoxy-6-polyprenyl-1,4-benzoquinol methylase UbiE |
| RJ610_21690 | WND79868.1 | 1,10 | 5,70E-01 | | | GNAT family N-acetyltransferase |
| RJ610_21700 | WND79870.1 | 0,65 | 7,95E-04 | | | N-acetyltransferase |
| RJ610_21705 | WND79871.1 | 0,52 | 7,24E-05 | | | DUF2269 domain-containing protein |
| RJ610_21710 | WND79872.1 | 0,67 | 1,43E-02 | | | SDR family oxidoreductase |
| RJ610_21715 | WND83323.1 | 1,08 | 7,75E-01 | | | ATP-dependent protease ATPase subunit HslU |
| RJ610_21725 | WND79874.1 | 0,70 | 1,80E-02 | | | tyrosine recombinase XerC |
| RJ610_21730 | WND79875.1 | 0,92 | 6,04E-01 | | | hypothetical protein |
| RJ610_21735 | WND79876.1 | 0,72 | 3,80E-03 | | | DUF484 family protein |
| RJ610_21740 | WND79877.1 | 0,63 | 1,56E-04 | | | diaminopimelate epimerase |
| RJ610_21745 | WND79878.1 | 0,79 | 7,37E-02 | | | lipoprotein |
| RJ610_21750 | WND79879.1 | 0,61 | 7,92E-02 | | | tautomerase family protein |
| RJ610_21755 | WND83324.1 | 0,67 | 7,33E-02 | | | YbaN family protein |
| RJ610_21770 | WND79882.1 | 0,40 | 4,99E-08 | | | hypothetical protein |
| RJ610_21775 | WND79883.1 | 1,11 | 3,26E-01 | | | patatin-like phospholipase family protein |
| RJ610_21780 | WND79884.1 | 0,99 | 9,59E-01 | | | YafY family protein |
| RJ610_21785 | WND79885.1 | 1,03 | 8,37E-01 | | | hypothetical protein |
| RJ610_21790 | WND79886.1 | 3,77 | 1,24E-24 | | | hypothetical protein |

| | | | | | | |
|---|---|---|---|---|---|---|
| RJ610_21795 | WND79887.1 | 0,96 | 7,76E-01 | | | molybdopterin oxidoreductase family protein |
| RJ610_21800 | WND79888.1 | 0,70 | 4,07E-01 | | | hypothetical protein |
| RJ610_21805 | WND79889.1 | 0,82 | 3,21E-01 | | | 4Fe-4S dicluster domain-containing protein |
| RJ610_21810 | WND79890.1 | 1,16 | 4,20E-01 | | | DmsC/YnfH family molybdoenzyme membrane anchor subunit |
| RJ610_21815 | WND79891.1 | 0,89 | 4,20E-01 | | | DUF481 domain-containing protein |
| RJ610_21820 | WND79892.1 | 0,77 | 1,70E-02 | | | hydroxymethylbilane synthase |
| RJ610_21825 | WND79893.1 | 0,75 | 6,18E-03 | | | LytTR family DNA-binding domain-containing protein |
| RJ610_21830 | WND83325.1 | 0,87 | 2,41E-01 | | | histidine kinase |
| RJ610_21835 | WND79894.1 | 0,68 | 2,48E-04 | | | carboxylesterase |
| RJ610_21840 | WND79895.1 | 1,35 | 9,24E-02 | | | TonB family protein |
| RJ610_21845 | WND79896.1 | 0,41 | 1,25E-08 | | | hypothetical protein |
| RJ610_21850 | WND79897.1 | 0,37 | 7,72E-06 | | | TolC family protein |
| RJ610_21855 | WND79898.1 | 0,34 | 2,50E-05 | | | efflux RND transporter periplasmic adaptor subunit |
| RJ610_21860 | WND83326.1 | 0,33 | 8,75E-09 | | | CusA/CzcA family heavy metal efflux RND transporter |
| RJ610_21865 | WND79899.1 | 0,66 | 1,24E-02 | | | cation diffusion facilitator family transporter |
| RJ610_21870 | WND79900.1 | 4,64 | 3,36E-24 | | | hypothetical protein |
| RJ610_21875 | WND79901.1 | 4,11 | 5,70E-26 | | | xanthine dehydrogenase family protein molybdopterin-binding subunit |
| RJ610_21880 | WND79902.1 | 3,19 | 1,67E-11 | | | hypothetical protein |
| RJ610_21885 | WND79903.1 | 2,79 | 2,64E-22 | | | (2Fe-2S)-binding protein |
| RJ610_21890 | WND79904.1 | 1,95 | 7,96E-14 | | | XdhC family protein |
| RJ610_21895 | WND79905.1 | 1,96 | 1,54E-08 | | | nucleotidyltransferase family protein |
| RJ610_21900 | WND79906.1 | 10,50 | 1,87E-110 | | | hypothetical protein |
| RJ610_21905 | WND79907.1 | 1,38 | 1,52E-02 | | | LysR family transcriptional regulator |
| RJ610_21910 | WND79908.1 | 1,14 | 6,82E-01 | | | carboxymuconolactone decarboxylase family protein |
| RJ610_21915 | WND79909.1 | 0,62 | 3,21E-03 | | | class I SAM-dependent methyltransferase |
| RJ610_21920 | WND79910.1 | 1,68 | 2,55E-05 | | | VOC family protein |
| RJ610_21925 | WND79911.1 | 1,08 | 6,09E-01 | | | DUF4031 domain-containing protein |
| RJ610_21930 | WND79912.1 | 1,62 | 7,59E-05 | | | LysE family translocator |
| RJ610_21940 | WND79914.1 | 0,64 | 1,09E-04 | | | response regulator transcription factor |
| RJ610_21945 | WND79915.1 | 1,00 | 9,74E-01 | | | HAMP domain-containing protein |
| RJ610_21950 | WND79916.1 | 1,30 | 1,59E-01 | | | DUF418 domain-containing protein |
| RJ610_21955 | WND79917.1 | 17,13 | 6,58E-51 | | | HlyD family efflux transporter periplasmic adaptor subunit |
| RJ610_21960 | WND79918.1 | 12,84 | 1,30E-95 | | | peptidase domain-containing ABC transporter |
| RJ610_21965 | WND79919.1 | 43,36 | 6,29E-250 | | | hypothetical protein |
| RJ610_21970 | WND79920.1 | 24,95 | 2,08E-130 | | | hypothetical protein |
| RJ610_21980 | WND79922.1 | 1,69 | 6,37E-04 | | | DUF3224 domain-containing protein |
| RJ610_21985 | WND79923.1 | 4,90 | 5,95E-17 | | | TonB-dependent siderophore receptor |
| RJ610_21990 | WND79924.1 | 5,71 | 2,45E-15 | | | PepSY domain-containing protein |
| RJ610_21995 | WND79925.1 | 38,40 | 2,34E-85 | | | autotransporter-associated beta strand repeat-containing protein |
| RJ610_22005 | WND79927.1 | 0,73 | 1,46E-01 | | | MarR family transcriptional regulator |
| RJ610_22010 | WND83327.1 | 3,02 | 2,09E-10 | | | serine hydrolase domain-containing protein |
| RJ610_22015 | WND79928.1 | 2,86 | 4,60E-16 | | | M15 family metallopeptidase |

| | | | | | | |
|---|---|---|---|---|---|---|
| RJ610_22020 | WND79929.1 | 1,83 | 1,13E-03 | | | serine hydrolase |
| RJ610_22025 | WND79930.1 | 1,07 | 7,45E-01 | | | TonB-dependent receptor |
| RJ610_22030 | WND83328.1 | 1,00 | 9,99E-01 | | | transglutaminase domain-containing protein |
| RJ610_22035 | WND79931.1 | 0,82 | 7,73E-02 | | | SH3 domain-containing protein |
| RJ610_22040 | WND79932.1 | 0,93 | 6,09E-01 | | | dipeptide epimerase |
| RJ610_22045 | WND79933.1 | 0,95 | 7,18E-01 | | | MurR/RpiR family transcriptional regulator |
| RJ610_22050 | WND83329.1 | 1,00 | 9,88E-01 | | | hypothetical protein |
| RJ610_22055 | WND83330.1 | 0,83 | 2,97E-01 | | | DUF885 family protein |
| RJ610_22060 | WND79934.1 | 2,37 | 6,31E-06 | | | hypothetical protein |
| RJ610_22065 | WND79935.1 | 5,46 | 1,20E-61 | | | C2 family cysteine protease |
| RJ610_22070 | WND79936.1 | 2,29 | 9,68E-10 | | | hypothetical protein |
| RJ610_22075 | WND79937.1 | 1,53 | 1,37E-03 | | | Xaa-Pro peptidase family protein |
| RJ610_22080 | WND79938.1 | 1,09 | 7,18E-01 | | | aldehyde dehydrogenase family protein |
| RJ610_22085 | WND79939.1 | 0,98 | 9,33E-01 | | | dihydrodipicolinate synthase family protein |
| RJ610_22090 | WND79940.1 | 1,21 | 6,91E-01 | | | FAD/NAD(P)-binding oxidoreductase |
| RJ610_22095 | WND79941.1 | 1,12 | 8,75E-01 | | | 2Fe-2S iron-sulfur cluster-binding protein |
| RJ610_22100 | WND79942.1 | 0,71 | 3,53E-01 | | | FAD-dependent oxidoreductase |
| RJ610_22105 | WND79943.1 | 0,72 | 1,62E-01 | | | 4-hydroxyproline epimerase |
| RJ610_22110 | WND83331.1 | 0,54 | 6,58E-03 | | | AraC family transcriptional regulator |
| RJ610_22115 | WND79944.1 | 0,68 | 3,32E-02 | | | hypothetical protein |
| RJ610_22120 | WND79945.1 | 0,91 | 6,89E-01 | | | arginase family protein |
| RJ610_22125 | WND79946.1 | 1,23 | 8,19E-01 | | | hypothetical protein |
| RJ610_22130 | WND79947.1 | 0,92 | 6,02E-01 | | | M23 family metallopeptidase |
| RJ610_22140 | WND79949.1 | 1,53 | 1,53E-04 | | | amino acid deaminase |
| RJ610_22145 | WND79950.1 | 0,79 | 1,05E-01 | | | nuclear transport factor 2 family protein |
| RJ610_22150 | WND79951.1 | 0,56 | 1,93E-06 | | | hypothetical protein |
| RJ610_22155 | WND79952.1 | 0,36 | 1,03E-05 | | | hypothetical protein |
| RJ610_22160 | WND79953.1 | 1,51 | 2,73E-03 | | | PLP-dependent aminotransferase family protein |
| RJ610_22165 | WND79954.1 | 4,03 | 1,60E-22 | | | diguanylate cyclase |
| RJ610_22170 | WND83332.1 | 1,25 | 1,77E-01 | | | D-amino acid dehydrogenase |
| RJ610_22175 | WND79955.1 | 0,48 | 1,80E-03 | | | CPBP family intramembrane glutamic endopeptidase |
| RJ610_22180 | WND79956.1 | 1,74 | 6,44E-04 | | | VOC family protein |
| RJ610_22185 | WND79957.1 | 2,08 | 1,20E-01 | | | hypothetical protein |
| RJ610_22190 | WND79958.1 | 3,63 | 3,32E-03 | | | TlpA disulfide reductase family protein |
| RJ610_22200 | WND79960.1 | 76,71 | 7,95E-76 | | | acyltransferase |
| RJ610_22205 | WND79961.1 | 3,89 | 3,98E-11 | | | ECF-type sigma factor |
| RJ610_22210 | WND79962.1 | 1,32 | 2,27E-02 | | | serine/threonine-protein kinase |
| RJ610_22215 | WND79963.1 | 0,70 | 9,06E-03 | | | hypothetical protein |
| RJ610_22220 | WND79964.1 | 1,51 | 5,90E-03 | | | serine hydrolase |
| RJ610_22225 | WND79965.1 | 1,10 | 5,39E-01 | | | winged helix-turn-helix domain-containing protein |
| RJ610_22230 | WND79966.1 | 0,46 | 9,39E-02 | | | hypothetical protein |
| RJ610_22235 | WND79967.1 | 1,32 | 1,57E-01 | | | MFS transporter |
| RJ610_22240 | WND79968.1 | 0,74 | 2,43E-02 | | | LysR substrate-binding domain-containing protein |

| | | | | | | |
|---|---|---|---|---|---|---|
| RJ610_22245 | WND79969.1 | 0,77 | 4,51E-02 | | | DUF2058 domain-containing protein |
| RJ610_22250 | WND79970.1 | 0,84 | 1,76E-01 | | | hypothetical protein |
| RJ610_22255 | WND79971.1 | 0,99 | 9,24E-01 | | | relaxation protein |
| RJ610_22260 | WND79972.1 | 0,91 | 4,24E-01 | | | hypothetical protein |
| RJ610_22265 | WND79973.1 | 0,59 | 1,99E-06 | | | MFS transporter |
| RJ610_22270 | WND79974.1 | 1,24 | 6,72E-02 | | | hypothetical protein |
| RJ610_22275 | WND79975.1 | 1,45 | 6,87E-03 | | | hypothetical protein |
| RJ610_22280 | WND79976.1 | 1,37 | 2,21E-02 | | | hypothetical protein |
| RJ610_22285 | WND79977.1 | 2,12 | 2,40E-07 | | | hypothetical protein |
| RJ610_22290 | WND79978.1 | 1,12 | 3,58E-01 | | | metallophosphoesterase |
| RJ610_22295 | WND79979.1 | 1,14 | 5,76E-01 | | | sigma-70 family RNA polymerase sigma factor |
| RJ610_22300 | WND79980.1 | 1,30 | 1,53E-01 | | | hypothetical protein |
| RJ610_22305 | WND79981.1 | 1,44 | 5,78E-02 | | | RidA family protein |
| RJ610_22310 | WND79982.1 | 1,13 | 5,17E-01 | | | LysR family transcriptional regulator |
| RJ610_22315 | WND79983.1 | 1,91 | 7,53E-08 | | | hypothetical protein |
| RJ610_22325 | WND79985.1 | 2,19 | 1,59E-14 | | | ZIP family metal transporter |
| RJ610_22330 | WND79986.1 | 1,70 | 5,13E-07 | | | WG repeat-containing protein |
| RJ610_22335 | WND79987.1 | 1,30 | 1,71E-01 | | | hypothetical protein |
| RJ610_22345 | WND79989.1 | 7,78 | 5,19E-17 | | | hypothetical protein |
| RJ610_22350 | WND79990.1 | 5,19 | 4,91E-12 | | | DUF4019 domain-containing protein |
| RJ610_22355 | WND79991.1 | 3,72 | 1,51E-12 | | | OmpA family protein |
| RJ610_22360 | WND79992.1 | 1,52 | 8,29E-04 | | | ESPR-type extended signal peptide-containing protein |
| RJ610_22365 | WND79993.1 | 2,75 | 4,26E-02 | | | hypothetical protein |
| RJ610_22370 | WND79994.1 | 19,57 | 1,66E-131 | | | ATP-binding protein |
| RJ610_22375 | WND79995.1 | 6,08 | 1,77E-47 | | | two-component regulator propeller domain-containing protein |
| RJ610_22380 | WND79996.1 | 6,85 | 1,18E-43 | | | response regulator transcription factor |
| RJ610_22385 | WND79997.1 | 3,58 | 2,69E-06 | | | hypothetical protein |
| RJ610_22390 | WND79998.1 | 2,11 | 4,62E-05 | | | LysR family transcriptional regulator |
| RJ610_22395 | WND79999.1 | 2,19 | 6,00E-10 | | | MFS transporter |
| RJ610_22400 | WND80000.1 | 2,40 | 3,82E-08 | | | alpha/beta fold hydrolase |
| RJ610_22405 | WND80001.1 | 1,61 | 8,98E-05 | | | gamma-glutamylcyclotransferase family protein |
| RJ610_22410 | WND80002.1 | 1,71 | 1,90E-05 | | | hypothetical protein |
| RJ610_22420 | WND80004.1 | 1,37 | 1,07E-03 | | | Dyp-type peroxidase |
| RJ610_22425 | WND80005.1 | 1,06 | 5,86E-01 | | | DnaJ C-terminal domain-containing protein |
| RJ610_22430 | WND80006.1 | 1,43 | 2,72E-03 | | | chaperone modulator CbpM |
| RJ610_22435 | WND80007.1 | 1,02 | 9,15E-01 | | | hypothetical protein |
| RJ610_22440 | WND80008.1 | 1,23 | 1,93E-01 | | | cupin domain-containing protein |
| RJ610_22445 | WND83333.1 | 1,77 | 1,73E-05 | | | class I SAM-dependent methyltransferase |
| RJ610_22450 | WND80009.1 | 4,16 | 7,28E-25 | | | hypothetical protein |
| RJ610_22455 | WND80010.1 | 3,76 | 7,86E-28 | | | type 2 lanthipeptide synthetase LanM family protein |
| RJ610_22460 | WND80011.1 | 1,20 | 1,62E-01 | | | DUF6229 family protein |
| RJ610_22465 | WND80012.1 | 10,63 | 1,40E-57 | | | MFS transporter |
| RJ610_22470 | WND80013.1 | 16,97 | 4,91E-33 | | | DUF4442 domain-containing protein |

| | | | | | | |
|---|---|---|---|---|---|---|
| RJ610_22475 | WND80014.1 | 34,56 | 5,43E-112 | | | phosphotransferase family protein |
| RJ610_22485 | WND80016.1 | 7,81 | 2,32E-78 | | | SapC family protein |
| RJ610_22490 | WND80017.1 | 7,15 | 3,53E-67 | | | cupin-like domain-containing protein |
| RJ610_22495 | WND80018.1 | 6,73 | 8,98E-107 | | | tryptophan 7-halogenase |
| RJ610_22505 | WND80020.1 | 5,47 | 6,32E-58 | | | glucokinase |
| RJ610_22510 | WND80021.1 | 2,15 | 6,39E-10 | | | AraC family transcriptional regulator |
| RJ610_22515 | WND80022.1 | 1,89 | 2,05E-02 | | | SDR family oxidoreductase |
| RJ610_22520 | WND80023.1 | 3,47 | 4,59E-09 | | | hypothetical protein |
| RJ610_22525 | WND80024.1 | 11,55 | 1,02E-25 | | | hypothetical protein |
| RJ610_22530 | WND80025.1 | 14,35 | 8,03E-102 | | | hypothetical protein |
| RJ610_22535 | WND80026.1 | 25,28 | 3,76E-223 | | | hypothetical protein |
| RJ610_22545 | WND80028.1 | 687,77 | 0,00E+00 | | | hypothetical protein |
| RJ610_22550 | WND80029.1 | 7,09 | 5,27E-19 | | | hypothetical protein |
| RJ610_22555 | WND80030.1 | 8,40 | 7,45E-19 | | | M15 family metallopeptidase |
| RJ610_22560 | WND80031.1 | 3,93 | 2,44E-13 | | | hypothetical protein |
| RJ610_22565 | WND80032.1 | 1,07 | 6,46E-01 | | | hypothetical protein |
| RJ610_22570 | WND80033.1 | 5,30 | 1,31E-53 | | | iron-containing redox enzyme family protein |
| RJ610_22575 | WND80034.1 | 1,24 | 1,41E-01 | | | aspartate aminotransferase family protein |
| RJ610_22580 | WND83334.1 | 1,17 | 3,93E-01 | | | acyltransferase |
| RJ610_22585 | WND80035.1 | 1,23 | 2,95E-01 | | | hypothetical protein |
| RJ610_22590 | WND80036.1 | 1,06 | 7,85E-01 | | | histidine kinase |
| RJ610_22595 | WND80037.1 | 0,90 | 7,19E-01 | | | LytTR family DNA-binding domain-containing protein |
| RJ610_22600 | WND80038.1 | 2,05 | 1,38E-03 | | | hypothetical protein |
| RJ610_22605 | WND80039.1 | 0,52 | 5,74E-06 | | | hypothetical protein |
| RJ610_22610 | WND80040.1 | 0,90 | 6,60E-01 | | | cupin domain-containing protein |
| RJ610_22615 | WND80041.1 | 1,02 | 9,25E-01 | | | hypothetical protein |
| RJ610_22620 | WND80042.1 | 1,19 | 1,30E-01 | | | DUF262 domain-containing protein |
| RJ610_22625 | WND80043.1 | 1,27 | 2,23E-02 | | | LacI family DNA-binding transcriptional regulator |
| RJ610_22635 | WND80044.1 | 1,54 | 4,66E-03 | | | nucleoside permease |
| RJ610_22640 | WND80045.1 | 1,91 | 1,55E-04 | | | Gfo/Idh/MocA family oxidoreductase |
| RJ610_22645 | WND83336.1 | 1,85 | 6,21E-04 | | | sugar phosphate isomerase/epimerase |
| RJ610_22650 | WND80046.1 | 1,93 | 1,06E-04 | | | cytochrome c |
| RJ610_22655 | WND80047.1 | 1,60 | 1,21E-02 | | | DUF1080 domain-containing protein |
| RJ610_22660 | WND80048.1 | 1,20 | 3,57E-01 | | | gluconate 2-dehydrogenase subunit 3 family protein |
| RJ610_22665 | WND80049.1 | 1,44 | 3,98E-02 | | | GMC family oxidoreductase |
| RJ610_22675 | WND80050.1 | 2,32 | 7,64E-09 | | | exo 1,3/1,4-beta-D-glucan glucohydrolase |
| RJ610_22685 | WND80052.1 | 1,43 | 8,52E-02 | | | hypothetical protein |
| RJ610_22690 | WND80053.1 | 1,09 | 6,05E-01 | | | LacI family DNA-binding transcriptional regulator |
| RJ610_22695 | WND80054.1 | 2,37 | 7,81E-08 | | | MFS transporter |
| RJ610_22700 | WND80055.1 | 4,55 | 2,79E-11 | | | hypothetical protein |
| RJ610_22705 | WND80056.1 | 12,45 | 4,83E-26 | | | hypothetical protein |
| RJ610_22710 | WND80057.1 | 52,66 | 9,81E-54 | | | YegP family protein |
| RJ610_22715 | WND80058.1 | 128,67 | 8,69E-199 | | | hypothetical protein |

| | | | | | | |
|---|---|---|---|---|---|---|
| RJ610_22720 | WND80059.1 | 1,45 | 8,59E-04 | | | hypothetical protein |
| RJ610_22725 | WND80060.1 | 1,59 | 3,46E-05 | | | DUF3800 domain-containing protein |
| RJ610_22730 | WND80061.1 | 1,15 | 2,73E-01 | | | hypothetical protein |
| RJ610_22735 | WND80062.1 | 1,32 | 1,88E-02 | | | hypothetical protein |
| RJ610_22740 | | 1,46 | 1,01E-01 | | | tRNA-Ile |
| RJ610_22745 | WND80063.1 | 1,36 | 1,84E-02 | | | GH92 family glycosyl hydrolase |
| RJ610_22750 | WND80064.1 | 1,03 | 8,50E-01 | | | LacI family DNA-binding transcriptional regulator |
| RJ610_22755 | WND80065.1 | 1,04 | 7,81E-01 | | | sugar MFS transporter |
| RJ610_22760 | WND80066.1 | 0,98 | 9,07E-01 | | | carbohydrate kinase |
| RJ610_22770 | WND80068.1 | 0,81 | 1,66E-01 | | | serine hydrolase domain-containing protein |
| RJ610_22775 | WND80069.1 | 0,60 | 1,19E-03 | | | nuclear transport factor 2 family protein |
| RJ610_22780 | WND80070.1 | 0,64 | 4,53E-04 | | | hypothetical protein |
| RJ610_22785 | WND80071.1 | 1,02 | 9,03E-01 | | | hypothetical protein |
| RJ610_22790 | WND80072.1 | 1,10 | 5,14E-01 | | | RNA polymerase sigma factor RpoD |
| RJ610_22795 | WND80073.1 | 1,91 | 3,15E-04 | | | D-aminoacyl-tRNA deacylase |
| RJ610_22800 | WND80074.1 | 0,80 | 7,66E-02 | | | lauroyl acyltransferase |
| RJ610_22805 | WND80075.1 | 0,80 | 1,44E-01 | | | PH domain-containing protein |
| RJ610_22810 | WND83338.1 | 0,83 | 9,66E-02 | | | PH domain-containing protein |
| RJ610_22815 | WND80076.1 | 0,62 | 8,78E-08 | | | UDP-N-acetylglucosamine 2-epimerase |
| RJ610_22820 | WND80077.1 | 0,65 | 8,43E-06 | | | glycosyltransferase family 2 protein |
| RJ610_22825 | WND80078.1 | 0,57 | 2,28E-04 | | | O-antigen ligase family protein |
| RJ610_22830 | WND80079.1 | 0,64 | 1,42E-05 | | | glycosyltransferase family 39 protein |
| RJ610_22835 | WND80080.1 | 1,53 | 4,79E-05 | | | 16S rRNA (cytosine(967)-C(5))-methyltransferase RsmB |
| RJ610_22840 | WND80081.1 | 1,55 | 5,13E-06 | | | methionyl-tRNA formyltransferase |
| RJ610_22855 | WND80084.1 | 1,16 | 2,90E-01 | | | DNA-processing protein DprA |
| RJ610_22860 | WND80085.1 | 1,11 | 3,24E-01 | | | DUF494 family protein |
| RJ610_22865 | WND80086.1 | 0,65 | 8,45E-05 | | | pilin |
| RJ610_22870 | WND80087.1 | 0,68 | 2,88E-05 | | | RDD family protein |
| RJ610_22875 | WND80088.1 | 1,24 | 5,64E-02 | | | DNA topoisomerase I |
| RJ610_22885 | WND83339.1 | 3,17 | 4,50E-15 | | | lytic polysaccharide monooxygenase |
| RJ610_22890 | WND80090.1 | 0,63 | 3,43E-03 | | | Sua5/YciO/YrdC/YwlC family protein |
| RJ610_22895 | WND80091.1 | 0,60 | 2,66E-03 | | | EamA family transporter |
| RJ610_22900 | WND80092.1 | 0,83 | 4,63E-02 | | | DUF4124 domain-containing protein |
| RJ610_22905 | WND80093.1 | 0,76 | 5,25E-03 | | | DUF4124 domain-containing protein |
| RJ610_22910 | WND80094.1 | 0,92 | 5,17E-01 | | | hypothetical protein |
| RJ610_22915 | WND80095.1 | 0,82 | 1,90E-01 | | | hypothetical protein |
| RJ610_22920 | WND80096.1 | 0,74 | 2,44E-02 | | | SDR family oxidoreductase |
| RJ610_22925 | WND80097.1 | 2,48 | 1,65E-14 | | | hypothetical protein |
| RJ610_22935 | WND80099.1 | 0,86 | 2,23E-01 | | | MATE family efflux transporter |
| RJ610_22940 | WND80100.1 | 0,52 | 4,99E-09 | | | DUF3667 domain-containing protein |
| RJ610_22945 | WND80101.1 | 0,62 | 2,98E-04 | | | DUF4286 family protein |
| RJ610_22955 | WND80103.1 | 0,78 | 9,04E-02 | | | DUF3106 domain-containing protein |
| RJ610_22960 | WND80104.1 | 0,85 | 1,94E-01 | | | hypothetical protein |

| | | | | | | |
|---|---|---|---|---|---|---|
| RJ610_22965 | WND80105.1 | 0,84 | 9,87E-02 | | | primosomal protein N' |
| RJ610_22970 | WND80106.1 | 0,68 | 8,79E-04 | | | g-type lysozyme inhibitor |
| RJ610_22975 | | 0,73 | 1,40E-02 | | | NYN domain-containing protein |
| RJ610_22985 | WND80108.1 | 1,05 | 8,08E-01 | | | NAD(P)/FAD-dependent oxidoreductase |
| RJ610_22990 | WND80109.1 | 7,07 | 3,35E-27 | | | hypothetical protein |
| RJ610_22995 | WND80110.1 | 1,22 | 2,33E-01 | | | DUF1203 domain-containing protein |
| RJ610_23000 | WND80111.1 | 1,54 | 1,43E-04 | | | RNA polymerase sigma factor RpoH |
| RJ610_23005 | WND80112.1 | 2,40 | 1,29E-06 | | | N-acetylmannosamine-6-phosphate 2-epimerase |
| RJ610_23010 | WND80113.1 | 1,94 | 2,33E-01 | | | hypothetical protein |
| RJ610_23015 | WND80114.1 | 2,19 | 2,95E-03 | | | peptidogalycan biosysnthesis protein |
| RJ610_23020 | WND80115.1 | 3,78 | 2,17E-07 | | | class I SAM-dependent methyltransferase |
| RJ610_23030 | WND80117.1 | 0,40 | 1,50E-06 | | | glycosyltransferase family 87 protein |
| RJ610_23035 | WND80118.1 | 0,19 | 1,14E-18 | | | HAD family hydrolase |
| RJ610_23040 | WND80119.1 | 0,34 | 3,75E-11 | | | glycosyltransferase 87 family protein |
| RJ610_23045 | WND80120.1 | 0,24 | 3,16E-14 | | | phosphatidylinositol-specific phospholipase C/glycerophosphodiester phosphodiesterase family protein |
| RJ610_23050 | WND80121.1 | 0,38 | 6,17E-07 | | | glycosyltransferase |
| RJ610_23055 | WND80122.1 | 0,26 | 3,25E-21 | | | NTP transferase domain-containing protein |
| RJ610_23060 | WND80123.1 | 0,86 | 3,02E-01 | | | class II aldolase/adducin family protein |
| RJ610_23065 | WND80124.1 | 0,15 | 6,36E-50 | | | amino acid adenylation domain-containing protein |
| RJ610_23070 | WND80125.1 | 0,16 | 5,97E-40 | | | amino acid adenylation domain-containing protein |
| RJ610_23075 | WND80126.1 | 0,14 | 9,67E-45 | | | amino acid adenylation domain-containing protein |
| RJ610_23080 | WND80127.1 | 1,28 | 2,46E-01 | | | MFS transporter |
| RJ610_23085 | WND80128.1 | 1,79 | 7,59E-04 | | | hypothetical protein |
| RJ610_23090 | WND80129.1 | 0,09 | 3,61E-36 | | | NAD(P)/FAD-dependent oxidoreductase |
| RJ610_23095 | WND80130.1 | 0,25 | 1,44E-14 | | | hypothetical protein |
| RJ610_23100 | WND80131.1 | 0,85 | 2,74E-01 | | | hypothetical protein |
| RJ610_23105 | WND80132.1 | 1,15 | 3,59E-01 | | | PLP-dependent aminotransferase family protein |
| RJ610_23110 | WND80133.1 | 1,27 | 2,27E-01 | | | glutathione binding-like protein |
| RJ610_23115 | WND80134.1 | 1,23 | 1,91E-01 | | | MBL fold metallo-hydrolase |
| RJ610_23120 | WND80135.1 | 1,21 | 1,63E-01 | | | cupin domain-containing protein |
| RJ610_23125 | WND80136.1 | 1,29 | 1,04E-01 | | | hypothetical protein |
| RJ610_23130 | WND80137.1 | 1,19 | 2,87E-01 | | | metalloregulator ArsR/SmtB family transcription factor |
| RJ610_23135 | WND80138.1 | 1,17 | 1,82E-01 | | | SRPBCC family protein |
| RJ610_23140 | WND80139.1 | 1,71 | 2,20E-05 | | | RHS repeat-associated core domain-containing protein |
| RJ610_23145 | WND80140.1 | 3,17 | 9,99E-25 | | | IPT/TIG domain-containing protein |
| RJ610_23150 | WND80141.1 | 2,55 | 3,03E-12 | | | cytochrome c peroxidase |
| RJ610_23155 | WND80142.1 | 1,12 | 6,01E-01 | | | hypothetical protein |
| RJ610_23160 | WND80143.1 | 1,63 | 5,95E-04 | | | hypothetical protein |
| RJ610_23165 | WND80144.1 | 1,63 | 9,93E-05 | | | hypothetical protein |
| RJ610_23170 | WND80145.1 | 1,95 | 7,88E-06 | | | response regulator |
| RJ610_23175 | WND80146.1 | 1,89 | 1,42E-04 | | | ATP-binding protein |
| RJ610_23180 | WND80147.1 | 2,26 | 1,23E-08 | | | hypothetical protein |

| | | | | | | |
|---|---|---|---|---|---|---|
| RJ610_23185 | WND80148.1 | 1,59 | 6,26E-03 | | | TetR/AcrR family transcriptional regulator |
| RJ610_23190 | WND80149.1 | 4030,81 | 0,00E+00 | | | amino acid adenylation domain-containing protein |
| RJ610_23195 | WND80150.1 | 1330,21 | 0,00E+00 | | | glycosyltransferase |
| RJ610_23200 | WND80151.1 | 8,22 | 1,75E-47 | | | patatin-like phospholipase family protein |
| RJ610_23205 | WND80152.1 | 3,84 | 1,09E-18 | | | phosphoethanolamine--lipid A transferase |
| RJ610_23210 | WND80153.1 | 2,30 | 3,63E-02 | | | phosphatase PAP2 family protein |
| RJ610_23215 | WND80154.1 | 1,08 | 6,33E-01 | | | response regulator transcription factor |
| RJ610_23220 | WND80155.1 | 1,02 | 9,07E-01 | | | HAMP domain-containing sensor histidine kinase |
| RJ610_23225 | WND80156.1 | 1,36 | 8,93E-02 | | | EcsC family protein |
| RJ610_23230 | WND80157.1 | 0,78 | 1,19E-01 | | | DUF3037 domain-containing protein |
| RJ610_23235 | WND83340.1 | 0,77 | 1,33E-02 | | | aminotransferase class I and II |
| RJ610_23240 | WND80158.1 | 1,07 | 7,72E-01 | | | NAD(P)H-dependent oxidoreductase |
| RJ610_23245 | WND80159.1 | 0,63 | 3,68E-02 | | | transcriptional regulator GcvA |
| RJ610_23250 | WND80160.1 | 0,79 | 4,20E-01 | | | 4-oxalocrotonate tautomerase family protein |
| RJ610_23255 | WND80161.1 | 1,77 | 2,05E-05 | | | LysR family transcriptional regulator |
| RJ610_23260 | WND80162.1 | 1,02 | 8,82E-01 | | | hypothetical protein |
| RJ610_23265 | WND80163.1 | 0,98 | 9,15E-01 | | | hypothetical protein |
| RJ610_23270 | WND80164.1 | 0,98 | 8,44E-01 | | | XRE family transcriptional regulator |
| RJ610_23275 | WND80165.1 | 1,46 | 2,49E-04 | | | uracil-DNA glycosylase |
| RJ610_23280 | WND83341.1 | 1,37 | 3,29E-02 | | | response regulator |
| RJ610_23285 | WND80166.1 | 0,61 | 1,38E-06 | | | permease-like cell division protein FtsX |
| RJ610_23290 | WND80167.1 | 0,54 | 3,59E-09 | | | cell division ATP-binding protein FtsE |
| RJ610_23295 | WND80168.1 | 5,05 | 2,13E-18 | | | hypothetical protein |
| RJ610_23300 | WND80169.1 | 0,95 | 6,27E-01 | | | ATP-dependent RNA helicase RhlB |
| RJ610_23310 | WND80171.1 | 1,02 | 8,46E-01 | | | transcription termination factor Rho |
| RJ610_23315 | WND80172.1 | 0,40 | 8,17E-15 | | | hypothetical protein |
| RJ610_23320 | WND80173.1 | 0,52 | 2,85E-06 | | | hypothetical protein |
| RJ610_23325 | WND80174.1 | 0,51 | 2,00E-08 | | | energy transducer TonB |
| RJ610_23330 | WND80175.1 | 0,89 | 4,51E-01 | | | monovalent cation:proton antiporter-2 (CPA2) family protein |
| RJ610_23335 | WND80176.1 | 0,97 | 9,04E-01 | | | ankyrin repeat domain-containing protein |
| RJ610_23340 | WND80177.1 | 1,71 | 3,72E-02 | | | YcgL domain-containing protein |
| RJ610_23345 | WND80178.1 | 8,55 | 2,48E-04 | | | cytochrome d ubiquinol oxidase subunit II |
| RJ610_23350 | WND80179.1 | 11,20 | 1,48E-06 | | | cytochrome ubiquinol oxidase subunit I |
| RJ610_23355 | WND80180.1 | 0,53 | 6,03E-04 | | | prolyl oligopeptidase family serine peptidase |
| RJ610_23360 | WND80181.1 | 8,47 | 4,29E-02 | | | hypothetical protein |
| RJ610_23370 | WND80183.1 | 0,85 | 5,05E-01 | | | DUF421 domain-containing protein |
| RJ610_23380 | WND80185.1 | 1,15 | 1,95E-01 | | | DNA polymerase I |
| RJ610_23385 | WND83342.1 | 2,43 | 1,24E-09 | | | DUF2782 domain-containing protein |
| RJ610_23390 | WND80186.1 | 2,08 | 9,37E-08 | | | GNAT family N-acetyltransferase |
| RJ610_23395 | WND80187.1 | 1,47 | 8,13E-02 | | | M15 family metallopeptidase |
| RJ610_23400 | WND80188.1 | 0,41 | 3,28E-09 | | | S9 family peptidase |
| RJ610_23405 | WND80189.1 | 0,53 | 4,79E-04 | | | hypothetical protein |
| RJ610_23410 | WND80190.1 | 0,85 | 6,80E-01 | | | phytase |

| | | | | | | |
|---|---|---|---|---|---|---|
| RJ610_23415 | WND80191.1 | 1,19 | 3,62E-01 | | | TonB-dependent receptor |
| RJ610_23420 | WND80192.1 | 1,02 | 8,94E-01 | | | DNA helicase II |
| RJ610_23425 | WND80193.1 | 1,83 | 1,44E-03 | | | hypothetical protein |
| RJ610_23430 | WND80194.1 | 0,55 | 5,16E-08 | | | TIGR00645 family protein |
| RJ610_23435 | WND80195.1 | 0,87 | 4,70E-01 | | | glutathione binding-like protein |
| RJ610_23440 | WND83343.1 | 0,76 | 4,72E-02 | | | S9 family peptidase |
| RJ610_23450 | WND80197.1 | 0,31 | 3,00E-13 | | | hypothetical protein |
| RJ610_23455 | WND80198.1 | 0,63 | 2,51E-01 | | | hypothetical protein |
| RJ610_23460 | WND80199.1 | 1,32 | 2,45E-01 | | | serine hydrolase domain-containing protein |
| RJ610_23465 | WND80200.1 | 1,09 | 8,45E-01 | | | hypothetical protein |
| RJ610_23470 | WND80201.1 | 2,48 | 1,77E-02 | | | hypothetical protein |
| RJ610_23475 | WND80202.1 | 4,04 | 1,00E-24 | | | XRE family transcriptional regulator |
| RJ610_23480 | WND80203.1 | 8,01 | 9,88E-25 | | | cupin domain-containing protein |
| RJ610_23485 | WND80204.1 | 7,08 | 6,73E-12 | | | hypothetical protein |
| RJ610_23490 | WND80205.1 | 14,62 | 9,72E-61 | | | DUF4135 domain-containing protein |
| RJ610_23495 | WND80206.1 | 17,88 | 5,73E-120 | | | hypothetical protein |
| RJ610_23500 | WND80207.1 | 18,07 | 4,44E-130 | | | hypothetical protein |
| RJ610_23505 | WND80208.1 | 0,97 | 8,85E-01 | | | YdcF family protein |
| RJ610_23510 | WND80209.1 | 0,59 | 2,89E-07 | | | beta-ketoacyl-[acyl-carrier-protein] synthase family protein |
| RJ610_23515 | WND83344.1 | 0,55 | 9,77E-06 | | | beta-ketoacyl synthase chain length factor |
| RJ610_23520 | WND80210.1 | 0,62 | 3,80E-05 | | | glycosyltransferase family 2 protein |
| RJ610_23525 | WND80211.1 | 0,92 | 5,84E-01 | | | IS1595 family transposase |
| RJ610_23530 | WND80212.1 | 0,80 | 3,30E-02 | | | hypothetical protein |
| RJ610_23535 | WND80213.1 | 0,60 | 1,63E-01 | | | hypothetical protein |
| RJ610_23540 | WND80214.1 | 0,77 | 1,98E-01 | | | 3-oxoacyl-ACP reductase FabG |
| RJ610_23545 | WND80215.1 | 0,51 | 2,63E-04 | | | hypothetical protein |
| RJ610_23550 | WND80216.1 | 0,76 | 4,31E-02 | | | hypothetical protein |
| RJ610_23555 | WND83345.1 | 0,59 | 5,13E-05 | | | phosphotransferase |
| RJ610_23565 | WND80218.1 | 0,75 | 9,92E-03 | | | MMPL family transporter |
| RJ610_23570 | WND80219.1 | 0,60 | 9,77E-04 | | | LolA-related protein |
| RJ610_23575 | WND80220.1 | 0,48 | 8,82E-12 | | | acyltransferase |
| RJ610_23580 | WND80221.1 | 0,48 | 2,50E-07 | | | hypothetical protein |
| RJ610_23585 | WND80222.1 | 0,53 | 6,24E-05 | | | ketosynthase |
| RJ610_23590 | WND80223.1 | 0,47 | 1,01E-11 | | | phosphopantetheine-binding protein |
| RJ610_23595 | WND80224.1 | 0,73 | 4,96E-04 | | | NAD(P)/FAD-dependent oxidoreductase |
| RJ610_23600 | WND83346.1 | 0,72 | 4,67E-03 | | | pteridine-dependent deoxygenase |
| RJ610_23605 | WND80225.1 | 0,20 | 7,62E-36 | | | hypothetical protein |
| RJ610_23610 | WND83347.1 | 0,53 | 5,10E-10 | | | hypothetical protein |
| RJ610_23620 | WND80227.1 | 1,29 | 2,51E-01 | | | hypothetical protein |
| RJ610_23625 | WND80228.1 | 0,98 | 9,33E-01 | | | ABC transporter ATP-binding protein |
| RJ610_23630 | WND80229.1 | 0,71 | 6,49E-02 | | | ABC transporter permease |
| RJ610_23635 | WND80230.1 | 2,95 | 2,03E-02 | | | Rrf2 family transcriptional regulator |
| RJ610_23640 | WND80231.1 | 3,78 | 2,46E-02 | | | hypothetical protein |

| | | | | | | |
|---|---|---|---|---|---|---|
| RJ610_23645 | WND80232.1 | 3,18 | 5,95E-02 | | | porin |
| RJ610_23655 | WND80234.1 | 1,86 | 4,67E-04 | | | hypothetical protein |
| RJ610_23660 | WND80235.1 | 2,04 | 6,06E-04 | | | sulfate ABC transporter permease subunit CysT |
| RJ610_23665 | WND80236.1 | 1,94 | 4,05E-04 | | | sulfate ABC transporter permease subunit CysW |
| RJ610_23670 | WND80237.1 | 2,22 | 1,81E-06 | | | sulfate/molybdate ABC transporter ATP-binding protein |
| RJ610_23680 | WND80239.1 | 12,54 | 7,45E-32 | | | hypothetical protein |
| RJ610_23685 | WND80240.1 | 7,34 | 1,46E-34 | | | hypothetical protein |
| RJ610_23690 | WND80241.1 | 2,16 | 1,05E-06 | | | TolB-like protein |
| RJ610_23695 | WND80242.1 | 1,38 | 6,00E-02 | | | shikimate dehydrogenase |
| RJ610_23700 | WND80243.1 | 1,31 | 2,22E-01 | | | hypothetical protein |
| RJ610_23705 | WND80244.1 | 1,38 | 9,28E-02 | | | DUF952 domain-containing protein |
| RJ610_23710 | WND80245.1 | 1,93 | 6,06E-04 | | | YkgJ family cysteine cluster protein |
| RJ610_23715 | WND80246.1 | 0,29 | 1,02E-13 | | | DUF58 domain-containing protein |
| RJ610_23720 | WND80247.1 | 0,14 | 1,34E-46 | | | MoxR family ATPase |
| RJ610_23725 | WND80248.1 | 0,26 | 4,06E-26 | | | DUF4350 domain-containing protein |
| RJ610_23730 | WND80249.1 | 0,20 | 1,60E-25 | | | DUF4129 domain-containing protein |
| RJ610_23735 | WND80250.1 | 0,18 | 8,29E-27 | | | stage II sporulation protein M |
| RJ610_23740 | WND80251.1 | 0,30 | 2,44E-25 | | | RDD family protein |
| RJ610_23745 | WND80252.1 | 0,39 | 3,04E-14 | | | RDD family protein |
| RJ610_23755 | WND80254.1 | 1,61 | 6,36E-07 | | | coniferyl aldehyde dehydrogenase |
| RJ610_23760 | WND80255.1 | 0,45 | 1,63E-06 | | | hypothetical protein |
| RJ610_23765 | WND80256.1 | 0,37 | 1,82E-06 | | | hypothetical protein |
| RJ610_23770 | WND80257.1 | 0,52 | 8,82E-14 | | | exodeoxyribonuclease III |
| RJ610_23775 | WND80258.1 | 0,43 | 8,59E-05 | | | GlsB/YeaQ/YmgE family stress response membrane protein |
| RJ610_23780 | WND80259.1 | 0,58 | 2,42E-07 | | | AMP-binding protein |
| RJ610_23785 | WND80260.1 | 0,56 | 9,26E-04 | | | 4'-phosphopantetheinyl transferase superfamily protein |
| RJ610_23790 | WND80261.1 | 0,72 | 1,25E-03 | | | 16S rRNA (guanine(527)-N(7))-methyltransferase RsmG |
| RJ610_23795 | WND80262.1 | 0,64 | 4,84E-06 | | | AAA family ATPase |
| RJ610_23800 | WND80263.1 | 0,72 | 5,17E-03 | | | ParB/RepB/Spo0J family partition protein |
| RJ610_23805 | WND80264.1 | 0,61 | 7,78E-05 | | | hypothetical protein |
| RJ610_23810 | WND80265.1 | 0,61 | 1,19E-06 | | | NAD-dependent epimerase/dehydratase family protein |
| RJ610_23815 | WND80266.1 | 0,72 | 4,01E-03 | | | glycosyltransferase family 2 protein |
| RJ610_23820 | WND80267.1 | 0,89 | 3,28E-01 | | | lipid-A-disaccharide synthase N-terminal domain-containing protein |
| RJ610_23825 | WND80268.1 | 0,95 | 6,86E-01 | | | GFA family protein |
| RJ610_23830 | WND80269.1 | 0,78 | 2,89E-02 | | | 50S ribosomal protein L28 |
| RJ610_23835 | WND80270.1 | 0,79 | 4,52E-02 | | | 50S ribosomal protein L33 |
| RJ610_23845 | WND80272.1 | 1,33 | 5,47E-02 | | | NahK/ErcS family hybrid sensor histidine kinase/response regulator |
| RJ610_23850 | WND80273.1 | 0,71 | 1,58E-01 | | | DUF2167 domain-containing protein |
| RJ610_23855 | WND80274.1 | 1,16 | 6,89E-01 | | | hypothetical protein |
| RJ610_23860 | WND80275.1 | 1,35 | 7,95E-02 | | | J domain-containing protein |
| RJ610_23865 | WND80276.1 | 1,98 | 5,32E-06 | | | response regulator transcription factor |

| | | | | | | |
|---|---|---|---|---|---|---|
| RJ610_23870 | WND80277.1 | 1,85 | 1,50E-02 | | | endonuclease domain-containing protein |
| RJ610_23880 | WND80279.1 | 3,46 | 2,52E-08 | | | DcaP family trimeric outer membrane transporter |
| RJ610_23885 | WND80280.1 | 2,88 | 5,35E-07 | | | MFS transporter |
| RJ610_23890 | WND80281.1 | 2,18 | 1,53E-08 | | | uracil-DNA glycosylase family protein |
| RJ610_23895 | WND80282.1 | 1,87 | 4,08E-03 | | | trimeric intracellular cation channel family protein |
| RJ610_23900 | WND80283.1 | 1,23 | 3,22E-01 | | | ABC transporter ATP-binding protein |
| RJ610_23905 | WND80284.1 | 2,36 | 2,40E-09 | | | hypothetical protein |
| RJ610_23910 | WND80285.1 | 2,39 | 2,81E-12 | | | hypothetical protein |
| RJ610_23915 | WND80286.1 | 4,04 | 2,12E-32 | | | kanamycin nucleotidyltransferase C-terminal domain-containing protein |
| RJ610_23920 | WND80287.1 | 1,62 | 8,25E-05 | | | hypothetical protein |
| RJ610_23925 | WND80288.1 | 2,28 | 2,51E-08 | | | hypothetical protein |
| RJ610_23930 | WND80289.1 | 0,71 | 2,96E-04 | | | fatty acid desaturase |
| RJ610_23935 | WND80290.1 | 1,04 | 8,29E-01 | | | CHAD domain-containing protein |
| RJ610_23940 | WND80291.1 | 0,81 | 1,75E-01 | | | bifunctional DNA-formamidopyrimidine glycosylase/DNA-(apurinic or apyrimidinic site) lyase |
| RJ610_23945 | WND80292.1 | 3,20 | 1,97E-24 | | | ECF-type sigma factor |
| RJ610_23950 | WND80293.1 | 2,46 | 6,36E-14 | | | serine/threonine-protein kinase |
| RJ610_23955 | WND80294.1 | 1,39 | 3,96E-02 | | | DinB family protein |
| RJ610_23960 | WND80295.1 | 0,85 | 2,08E-01 | | | translocation/assembly module TamB domain-containing protein |
| RJ610_23965 | WND80296.1 | 0,93 | 5,21E-01 | | | outer membrane protein assembly factor |
| RJ610_23975 | WND80298.1 | 0,91 | 8,03E-01 | | | hypothetical protein |
| RJ610_23980 | WND80299.1 | 0,93 | 5,62E-01 | | | glycine--tRNA ligase subunit beta |
| RJ610_23985 | WND80300.1 | 0,85 | 1,80E-01 | | | glycine--tRNA ligase subunit alpha |
| RJ610_23990 | WND80301.1 | 0,99 | 9,22E-01 | | | glutamine amidotransferase |
| RJ610_23995 | WND80302.1 | 0,95 | 6,72E-01 | | | hypothetical protein |
| RJ610_24000 | WND80303.1 | 1,07 | 6,67E-01 | | | RDD family protein |
| RJ610_24005 | WND80304.1 | 1,07 | 5,34E-01 | | | twin-arginine translocase subunit TatC |
| RJ610_24010 | WND80305.1 | 1,25 | 4,69E-02 | | | Sec-independent protein translocase protein TatB |
| RJ610_24015 | WND80306.1 | 1,07 | 5,65E-01 | | | Sec-independent protein translocase subunit TatA |
| RJ610_24020 | WND83348.1 | 0,50 | 4,16E-07 | | | lipid-binding SYLF domain-containing protein |
| RJ610_24025 | WND80307.1 | 0,40 | 8,05E-14 | | | ferrochelatase |
| RJ610_24030 | WND83349.1 | 0,55 | 2,27E-06 | | | alpha/beta hydrolase |
| RJ610_24035 | WND80308.1 | 0,50 | 1,75E-11 | | | SprT family zinc-dependent metalloprotease |
| RJ610_24040 | WND80309.1 | 0,46 | 5,15E-16 | | | recombination-associated protein RdgC |
| RJ610_24045 | WND80310.1 | 0,49 | 9,79E-11 | | | tRNA 2-thiocytidine(32) synthetase TtcA |
| RJ610_24050 | WND80311.1 | 0,69 | 8,44E-03 | | | YdcH family protein |
| RJ610_24055 | WND80312.1 | 0,70 | 9,51E-03 | | | hypothetical protein |
| RJ610_24060 | WND80313.1 | 0,62 | 2,91E-03 | | | hypothetical protein |
| RJ610_24065 | WND80314.1 | 0,70 | 3,82E-03 | | | hypothetical protein |
| RJ610_24070 | WND80315.1 | 0,55 | 2,02E-08 | | | hypothetical protein |
| RJ610_24075 | WND80316.1 | 0,33 | 7,46E-20 | | | hypothetical protein |
| RJ610_24080 | WND80317.1 | 0,80 | 2,44E-02 | | | glycerol-3-phosphate 1-O-acyltransferase PlsB |

| | | | | | | |
|---|---|---|---|---|---|---|
| RJ610_24085 | WND80318.1 | 0,91 | 4,65E-01 | | | hypothetical protein |
| RJ610_24090 | WND80319.1 | 63,51 | 1,54E-226 | | | spore coat U domain-containing protein |
| RJ610_24095 | WND80320.1 | 16,17 | 5,29E-126 | | | molecular chaperone |
| RJ610_24100 | WND80321.1 | 21,46 | 2,25E-88 | | | fimbria/pilus outer membrane usher protein |
| RJ610_24105 | WND80322.1 | 10,51 | 3,75E-50 | | | spore coat U domain-containing protein |
| RJ610_24110 | WND80323.1 | 0,95 | 8,04E-01 | | | alpha/beta hydrolase |
| RJ610_24115 | WND80324.1 | 0,71 | 2,73E-03 | | | AraC family transcriptional regulator |
| RJ610_24120 | WND80325.1 | 0,57 | 9,10E-09 | | | 5'-nucleotidase, lipoprotein e(P4) family |
| RJ610_24125 | WND80326.1 | 0,50 | 1,54E-05 | | | hypothetical protein |
| RJ610_24130 | WND80327.1 | 0,68 | 7,33E-03 | | | RidA family protein |
| RJ610_24135 | WND80328.1 | 0,73 | 1,02E-01 | | | GNAT family protein |
| RJ610_24140 | WND80329.1 | 1,14 | 3,88E-01 | | | GNAT family N-acetyltransferase |
| RJ610_24145 | WND80330.1 | 2,06 | 1,43E-14 | | | DUF4272 domain-containing protein |
| RJ610_24150 | WND80331.1 | 1,12 | 4,31E-01 | | | HNH endonuclease |
| RJ610_24155 | WND80332.1 | 1,00 | 9,86E-01 | | | UvrD-helicase domain-containing protein |
| RJ610_24160 | WND80333.1 | 0,71 | 2,50E-02 | | | hypothetical protein |
| RJ610_24165 | WND80334.1 | 0,81 | 1,23E-01 | | | thymidine kinase |
| RJ610_24170 | WND80335.1 | 0,57 | 3,20E-05 | | | nucleotidyltransferase domain-containing protein |
| RJ610_24175 | WND80336.1 | 0,43 | 4,05E-07 | | | GNAT family N-acetyltransferase |
| RJ610_24180 | WND80337.1 | 0,62 | 5,45E-04 | | | YiiX family permuted papain-like enzyme |
| RJ610_24185 | WND80338.1 | 0,62 | 6,56E-03 | | | DUF938 domain-containing protein |
| RJ610_24190 | WND80339.1 | 0,56 | 2,33E-04 | | | Hsp70 family protein |
| RJ610_24195 | WND80340.1 | 0,81 | 4,14E-01 | | | YcxB family protein |
| RJ610_24200 | WND80341.1 | 0,31 | 2,34E-07 | | | DEAD/DEAH box helicase |
| RJ610_24205 | WND80342.1 | 0,37 | 1,54E-05 | | | ribonuclease H-like domain-containing protein |
| RJ610_24210 | WND80343.1 | 0,51 | 2,03E-04 | | | I78 family peptidase inhibitor |
| RJ610_24215 | WND80344.1 | 3,86 | 1,32E-14 | | | I78 family peptidase inhibitor |
| RJ610_24220 | WND80345.1 | 1,10 | 5,03E-01 | | | ribonuclease domain-containing protein |
| RJ610_24225 | WND80346.1 | 0,93 | 7,50E-01 | | | barstar family protein |
| RJ610_24230 | WND80347.1 | 0,34 | 4,96E-11 | | | DNA topoisomerase IB |
| RJ610_24235 | WND80348.1 | 0,25 | 1,55E-13 | | | hypothetical protein |
| RJ610_24240 | WND80349.1 | 0,88 | 5,94E-01 | | | TCR/Tet family MFS transporter |
| RJ610_24245 | WND80350.1 | 0,37 | 2,00E-04 | | | glutamate synthase large subunit |
| RJ610_24250 | WND80351.1 | 0,30 | 6,13E-08 | | | FAD-dependent oxidoreductase |
| RJ610_24255 | WND80352.1 | 0,56 | 3,32E-06 | | | GTP cyclohydrolase I FolE |
| RJ610_24260 | WND80353.1 | 0,38 | 6,74E-11 | | | hypothetical protein |
| RJ610_24265 | WND80354.1 | 0,62 | 4,28E-05 | | | diguanylate cyclase |
| RJ610_24270 | WND80355.1 | 1,08 | 7,01E-01 | | | multidrug resistance efflux transporter family protein |
| RJ610_24275 | WND80356.1 | 1,25 | 1,67E-01 | | | malonyl-ACP O-methyltransferase BioC |
| RJ610_24280 | WND80357.1 | 1,40 | 4,11E-02 | | | pimeloyl-ACP methyl ester esterase BioH |
| RJ610_24285 | WND80358.1 | 1,79 | 3,24E-04 | | | ACT domain-containing protein |
| RJ610_24290 | WND80359.1 | 1,62 | 3,92E-04 | | | 8-amino-7-oxononanoate synthase |
| RJ610_24295 | | 1,34 | 8,88E-03 | | | biotin synthase BioB |

| | | | | | | |
|---|---|---|---|---|---|---|
| RJ610_24300 | | 0,87 | 8,32E-01 | | | double zinc ribbon domain-containing protein |
| RJ610_24305 | WND83350.1 | 4,72 | 4,19E-12 | | | ComF family protein |
| RJ610_24310 | WND80360.1 | 16,84 | 4,19E-50 | | | M64 family metallopeptidase |
| RJ610_24315 | WND80361.1 | 0,59 | 4,73E-09 | | | hypothetical protein |
| RJ610_24320 | WND80362.1 | 0,93 | 6,56E-01 | | | TIGR00366 family protein |
| RJ610_24325 | WND80363.1 | 1,37 | 6,18E-02 | | | Rid family hydrolase |
| RJ610_24330 | WND80364.1 | 0,52 | 9,05E-07 | | | CshA/CshB family fibrillar adhesin-related protein |
| RJ610_24335 | WND80365.1 | 0,72 | 5,76E-03 | | | 4-hydroxybenzoate octaprenyltransferase |
| RJ610_24340 | | 1,81 | 3,46E-04 | | | tRNA-Arg |
| RJ610_24345 | WND80366.1 | 1,09 | 5,21E-01 | | | hypothetical protein |
| RJ610_24350 | WND80367.1 | 0,95 | 7,76E-01 | | | hypothetical protein |
| RJ610_24355 | WND80368.1 | 0,87 | 2,96E-01 | | | TIR domain-containing protein |
| RJ610_24360 | WND80369.1 | 1,13 | 3,03E-01 | | | hypothetical protein |
| RJ610_24365 | WND80370.1 | 1,26 | 2,28E-02 | | | hypothetical protein |
| RJ610_24370 | WND80371.1 | 1,24 | 3,35E-02 | | | hypothetical protein |
| RJ610_24375 | WND80372.1 | 1,18 | 1,71E-01 | | | HEPN/Toprim-associated domain-containing protein |
| RJ610_24380 | WND80373.1 | 1,15 | 2,59E-01 | | | hypothetical protein |
| RJ610_24385 | WND80374.1 | 0,97 | 8,56E-01 | | | H-NS histone family protein |
| RJ610_24390 | WND80375.1 | 1,29 | 8,31E-02 | | | hypothetical protein |
| RJ610_24395 | WND80376.1 | 1,08 | 5,90E-01 | | | hypothetical protein |
| RJ610_24400 | WND80377.1 | 1,41 | 1,74E-02 | | | SMI1/KNR4 family protein |
| RJ610_24405 | WND80378.1 | 2,07 | 7,81E-08 | | | hypothetical protein |
| RJ610_24410 | WND80379.1 | 1,88 | 5,88E-07 | | | hypothetical protein |
| RJ610_24415 | | 2,92 | 2,45E-14 | | | YkgJ family cysteine cluster protein |
| RJ610_24420 | WND80380.1 | 2,09 | 7,84E-10 | | | hypothetical protein |
| RJ610_24425 | WND80381.1 | 1,92 | 1,25E-07 | | | hypothetical protein |
| RJ610_24430 | WND80382.1 | 2,21 | 1,91E-06 | | | hypothetical protein |
| RJ610_24435 | WND80383.1 | 2,67 | 1,02E-15 | | | hypothetical protein |
| RJ610_24440 | WND80384.1 | 1,72 | 9,67E-07 | | | helix-turn-helix transcriptional regulator |
| RJ610_24445 | WND80385.1 | 1,46 | 5,67E-03 | | | LacI family transcriptional regulator |
| RJ610_24450 | WND80386.1 | 1,42 | 7,77E-03 | | | recombinase family protein |
| RJ610_24455 | WND80387.1 | 1,22 | 3,00E-01 | | | hypothetical protein |
| RJ610_24460 | WND80388.1 | 1,19 | 3,48E-01 | | | recombinase family protein |
| RJ610_24465 | WND80389.1 | 1,12 | 5,30E-01 | | | DUF2924 domain-containing protein |
| RJ610_24470 | WND80390.1 | 1,23 | 1,85E-01 | | | hypothetical protein |
| RJ610_24475 | WND80391.1 | 0,95 | 8,59E-01 | | | helix-turn-helix domain-containing protein |
| RJ610_24480 | WND80392.1 | 0,84 | 2,27E-01 | | | hypothetical protein |
| RJ610_24485 | WND80393.1 | 1,05 | 7,91E-01 | | | ATP-binding protein |
| RJ610_24490 | WND80394.1 | 1,01 | 9,76E-01 | | | hypothetical protein |
| RJ610_24495 | WND80395.1 | 1,18 | 5,90E-01 | | | hypothetical protein |
| RJ610_24500 | WND80396.1 | 1,12 | 6,19E-01 | | | DUF6511 domain-containing protein |
| RJ610_24505 | WND80397.1 | 0,95 | 8,41E-01 | | | hypothetical protein |
| RJ610_24510 | WND80398.1 | 1,44 | 3,92E-02 | | | phage/plasmid primase, P4 family |

| | | | | | | |
|---|---|---|---|---|---|---|
| RJ610_24515 | WND80399.1 | 1,74 | 1,58E-04 | | | hypothetical protein |
| RJ610_24520 | WND80400.1 | 1,87 | 2,50E-04 | | | hypothetical protein |
| RJ610_24525 | WND80401.1 | 1,42 | 2,41E-03 | | | DUF6362 family protein |
| RJ610_24530 | WND80402.1 | 0,90 | 5,25E-01 | | | hypothetical protein |
| RJ610_24535 | WND80403.1 | 2,00 | 2,96E-08 | | | hypothetical protein |
| RJ610_24540 | WND80404.1 | 2,06 | 5,46E-09 | | | hypothetical protein |
| RJ610_24545 | WND80405.1 | 20,18 | 4,05E-87 | | | hypothetical protein |
| RJ610_24550 | WND80406.1 | 7,90 | 9,81E-49 | | | site-specific DNA-methyltransferase |
| RJ610_24555 | WND80407.1 | 18,72 | 1,50E-123 | | | hypothetical protein |
| RJ610_24560 | WND80408.1 | 3,70 | 2,05E-12 | | | hypothetical protein |
| RJ610_24565 | WND80409.1 | 2,53 | 4,89E-06 | | | hypothetical protein |
| RJ610_24570 | WND80410.1 | 1,47 | 1,73E-03 | | | hypothetical protein |
| RJ610_24575 | WND80411.1 | 1,68 | 5,78E-05 | | | DUF3489 domain-containing protein |
| RJ610_24580 | WND80412.1 | 2,48 | 2,13E-06 | | | hypothetical protein |
| RJ610_24585 | WND80413.1 | 2,42 | 1,41E-03 | | | hypothetical protein |
| RJ610_24590 | WND80414.1 | 1,28 | 3,18E-01 | | | hypothetical protein |
| RJ610_24595 | WND80415.1 | 1,50 | 4,03E-01 | | | hypothetical protein |
| RJ610_24600 | WND80416.1 | 1,33 | 5,17E-01 | | | hypothetical protein |
| RJ610_24605 | WND80417.1 | 0,94 | 9,21E-01 | | | elements of external origin |
| RJ610_24610 | WND80418.1 | 1,26 | 4,48E-01 | | | phage terminase large subunit family protein |
| RJ610_24615 | WND80419.1 | 0,99 | 9,89E-01 | | | hypothetical protein |
| RJ610_24620 | WND80420.1 | 1,65 | 1,66E-01 | | | phage portal protein |
| RJ610_24625 | WND80421.1 | 2,65 | 2,92E-05 | | | S49 family peptidase |
| RJ610_24630 | WND80422.1 | 1,30 | 4,82E-01 | | | head decoration protein |
| RJ610_24635 | WND80423.1 | 1,41 | 5,89E-02 | | | major capsid protein |
| RJ610_24640 | WND80424.1 | 2,49 | 3,57E-02 | | | hypothetical protein |
| RJ610_24645 | WND80425.1 | 2,36 | 1,07E-02 | | | hypothetical protein |
| RJ610_24650 | WND80426.1 | 3,57 | 6,58E-04 | | | hypothetical protein |
| RJ610_24655 | WND80427.1 | 1,85 | 6,75E-03 | | | hypothetical protein |
| RJ610_24660 | WND80428.1 | 3,52 | 4,57E-04 | | | hypothetical protein |
| RJ610_24665 | WND80429.1 | 2,50 | 1,07E-02 | | | hypothetical protein |
| RJ610_24670 | WND80430.1 | 1,91 | 1,22E-02 | | | glycoside hydrolase family 104 protein |
| RJ610_24675 | WND80431.1 | 24,54 | 4,05E-51 | | | diguanylate cyclase |
| RJ610_24680 | WND80432.1 | 2,97 | 1,18E-09 | | | tape measure protein |
| RJ610_24685 | WND80433.1 | 1,10 | 5,45E-01 | | | hypothetical protein |
| RJ610_24690 | WND80434.1 | 1,23 | 3,23E-01 | | | hypothetical protein |
| RJ610_24695 | WND80435.1 | 1,44 | 2,10E-01 | | | hypothetical protein |
| RJ610_24700 | WND80436.1 | 0,76 | 3,84E-01 | | | phage BR0599 family protein |
| RJ610_24705 | WND80437.1 | 2,44 | 1,59E-01 | | | hypothetical protein |
| RJ610_24710 | WND80438.1 | 3,45 | 5,46E-02 | | | hypothetical protein |
| RJ610_24715 | WND80439.1 | 1,20 | 2,97E-01 | | | phage tail protein |
| RJ610_24720 | WND80440.1 | 1,07 | 6,51E-01 | | | hypothetical protein |
| RJ610_24725 | WND80441.1 | 0,97 | 8,27E-01 | | | hypothetical protein |

| | | | | | | |
|---|---|---|---|---|---|---|
| RJ610_24730 | WND80442.1 | 1,15 | 3,85E-01 | | | hypothetical protein |
| RJ610_24735 | WND80443.1 | 0,96 | 7,50E-01 | | | RHS repeat-associated core domain-containing protein |
| RJ610_24740 | WND80444.1 | 0,69 | 7,25E-05 | | | hypothetical protein |
| RJ610_24745 | WND80445.1 | 3,25 | 1,60E-08 | | | response regulator transcription factor |
| RJ610_24750 | WND80446.1 | 3,69 | 7,84E-14 | | | hypothetical protein |
| RJ610_24755 | WND80447.1 | 2,95 | 1,33E-12 | | | DUF3024 domain-containing protein |
| RJ610_24760 | WND80448.1 | 1,34 | 1,51E-02 | | | H-NS histone family protein |
| RJ610_24765 | WND80449.1 | 0,68 | 2,46E-02 | | | hypothetical protein |
| RJ610_24770 | WND80450.1 | 3,09 | 1,64E-13 | | | S8 family serine peptidase |
| RJ610_24775 | WND80451.1 | 2,43 | 7,90E-11 | | | HAMP domain-containing sensor histidine kinase |
| RJ610_24780 | WND80452.1 | 2,28 | 3,30E-04 | | | hypothetical protein |
| RJ610_24785 | WND80453.1 | 7,91 | 1,14E-54 | | | ESPR-type extended signal peptide-containing protein |
| RJ610_24790 | WND80454.1 | 10,36 | 7,20E-37 | | | transposase |
| RJ610_24795 | WND80455.1 | 5,79 | 3,75E-75 | | | winged helix-turn-helix domain-containing protein |
| RJ610_24800 | WND80456.1 | 3,06 | 8,26E-19 | | | RES family NAD+ phosphorylase |
| RJ610_24805 | WND80457.1 | 2,38 | 4,97E-11 | | | CorA family divalent cation transporter |
| RJ610_24810 | WND80458.1 | 2,56 | 1,29E-13 | | | DUF1328 domain-containing protein |
| RJ610_24815 | WND80459.1 | 3,12 | 1,17E-16 | | | zinc-dependent alcohol dehydrogenase |
| RJ610_24820 | WND80460.1 | 3,19 | 7,02E-11 | | | DUF4142 domain-containing protein |
| RJ610_24825 | WND80461.1 | 3,61 | 7,87E-12 | | | hypothetical protein |
| RJ610_24830 | WND80462.1 | 1,50 | 2,62E-03 | | | DUF892 family protein |
| RJ610_24835 | WND80463.1 | 1,67 | 2,32E-03 | | | hypothetical protein |
| RJ610_24840 | WND80464.1 | 1,52 | 2,61E-03 | | | hypothetical protein |
| RJ610_24845 | | 1,96 | 2,44E-07 | | | histidine phosphatase family protein |
| RJ610_24850 | WND80465.1 | 1,94 | 1,75E-07 | | | NAD(P)H-hydrate dehydratase |
| RJ610_24855 | WND80466.1 | 3,68 | 4,17E-13 | | | SOS response-associated peptidase |
| RJ610_24860 | WND80467.1 | 3,63 | 1,86E-17 | | | GFA family protein |
| RJ610_24865 | WND80468.1 | 6,19 | 3,84E-26 | | | YbhB/YbcL family Raf kinase inhibitor-like protein |
| RJ610_24870 | WND80469.1 | 2,40 | 5,96E-08 | | | AarF/UbiB family protein |
| RJ610_24875 | WND80470.1 | 1,42 | 4,99E-02 | | | hypothetical protein |
| RJ610_24880 | WND80471.1 | 1,08 | 6,72E-01 | | | isochorismatase family cysteine hydrolase |
| RJ610_24885 | WND80472.1 | 2,42 | 1,54E-04 | | | hypothetical protein |
| RJ610_24890 | WND80473.1 | 3,38 | 6,73E-22 | | | cation:proton antiporter |
| RJ610_24895 | WND80474.1 | 2,54 | 5,29E-08 | | | hypothetical protein |
| RJ610_24900 | WND80475.1 | 2,32 | 4,51E-09 | | | SDR family oxidoreductase |
| RJ610_24905 | WND80476.1 | 3,37 | 7,64E-15 | | | hypothetical protein |
| RJ610_24910 | WND80477.1 | 1,31 | 2,08E-01 | | | hypothetical protein |
| RJ610_24915 | WND80478.1 | 1,52 | 4,92E-03 | | | HNH endonuclease |
| RJ610_24920 | WND80479.1 | 1,33 | 6,63E-02 | | | dsDNA nuclease domain-containing protein |
| RJ610_24925 | WND80480.1 | 1,29 | 4,37E-02 | | | hypothetical protein |
| RJ610_24930 | WND80481.1 | 1,44 | 7,87E-03 | | | DUF3732 domain-containing protein |
| RJ610_24935 | WND80482.1 | 1,26 | 6,89E-02 | | | hypothetical protein |
| RJ610_24940 | WND80483.1 | 1,21 | 6,91E-02 | | | hypothetical protein |

| | | | | | | |
|---|---|---|---|---|---|---|
| RJ610_24945 | WND80484.1 | 0,99 | 9,08E-01 | | | hypothetical protein |
| RJ610_24950 | WND80485.1 | 1,05 | 7,16E-01 | | | AAA family ATPase |
| RJ610_24955 | WND80486.1 | 0,98 | 9,17E-01 | | | response regulator transcription factor |
| RJ610_24960 | WND80487.1 | 1,21 | 5,39E-01 | | | hypothetical protein |
| RJ610_24965 | WND80488.1 | 0,45 | 4,02E-13 | | | RHS repeat-associated core domain-containing protein |
| RJ610_24970 | WND80489.1 | 0,47 | 3,60E-05 | | | hypothetical protein |
| RJ610_24975 | WND80490.1 | 0,87 | 3,06E-01 | | | hypothetical protein |
| RJ610_24980 | WND80491.1 | 0,81 | 8,46E-02 | | | hypothetical protein |
| RJ610_24985 | WND80492.1 | 1,12 | 5,22E-01 | | | hypothetical protein |
| RJ610_24990 | WND80493.1 | 1,76 | 4,67E-04 | | | SDR family oxidoreductase |
| RJ610_24995 | WND80494.1 | 1,11 | 4,00E-01 | | | AraC family transcriptional regulator |
| RJ610_25000 | WND80495.1 | 1,40 | 1,84E-02 | | | SRPBCC family protein |
| RJ610_25005 | WND80496.1 | 1,17 | 2,70E-01 | | | hypothetical protein |
| RJ610_25010 | WND80497.1 | 1,03 | 8,64E-01 | | | hypothetical protein |
| RJ610_25015 | WND80498.1 | 1,01 | 9,71E-01 | | | acyl carrier protein |
| RJ610_25020 | WND80499.1 | 1,36 | 2,21E-02 | | | ATP-binding protein |
| RJ610_25025 | WND80500.1 | 0,85 | 1,30E-01 | | | crotonase/enoyl-CoA hydratase family protein |
| RJ610_25030 | WND80501.1 | 1,01 | 9,38E-01 | | | beta-ketoacyl-[acyl-carrier-protein] synthase family protein |
| RJ610_25035 | WND80502.1 | 1,51 | 2,46E-02 | | | TonB family protein |
| RJ610_25040 | WND80503.1 | 1,04 | 7,65E-01 | | | hypothetical protein |
| RJ610_25045 | WND80504.1 | 1,08 | 5,93E-01 | | | LysR family transcriptional regulator |
| RJ610_25050 | WND80505.1 | 1,13 | 5,87E-01 | | | nuclear transport factor 2 family protein |
| RJ610_25055 | WND80506.1 | 1,01 | 9,82E-01 | | | SDR family oxidoreductase |
| RJ610_25060 | WND80507.1 | 1,06 | 7,25E-01 | | | antibiotic biosynthesis monooxygenase |
| RJ610_25065 | WND80508.1 | 0,33 | 1,86E-23 | | | hypothetical protein |
| RJ610_25070 | WND80509.1 | 0,95 | 7,95E-01 | | | hypothetical protein |
| RJ610_25075 | WND80510.1 | 1,90 | 1,68E-01 | | | hypothetical protein |
| RJ610_25080 | WND80511.1 | 1,14 | 3,05E-01 | | | helix-turn-helix domain-containing protein |
| RJ610_25085 | WND80512.1 | 1,10 | 6,25E-01 | | | nuclear transport factor 2 family protein |
| RJ610_25090 | WND80513.1 | 1,25 | 1,56E-01 | | | nuclear transport factor 2 family protein |
| RJ610_25095 | WND80514.1 | 1,57 | 1,29E-01 | | | hypothetical protein |
| RJ610_25100 | WND80515.1 | 1,03 | 8,52E-01 | | | GNAT family N-acetyltransferase |
| RJ610_25105 | WND80516.1 | 1,19 | 3,22E-01 | | | hypothetical protein |
| RJ610_25110 | WND80517.1 | 2,10 | 8,22E-03 | | | AraC family transcriptional regulator |
| RJ610_25115 | WND80518.1 | 1,34 | 4,02E-02 | | | SDR family oxidoreductase |
| RJ610_25120 | WND80519.1 | 1,14 | 2,76E-01 | | | pentapeptide repeat-containing protein |
| RJ610_25125 | WND80520.1 | 1,08 | 5,34E-01 | | | hypothetical protein |
| RJ610_25130 | WND80521.1 | 0,85 | 3,59E-01 | | | DUF4034 domain-containing protein |
| RJ610_25135 | WND80522.1 | 1,21 | 3,37E-01 | | | DUF1266 domain-containing protein |
| RJ610_25140 | | 0,83 | 2,00E-01 | | | TolC family protein |
| RJ610_25145 | WND80523.1 | 1,16 | 2,14E-01 | | | TetR/AcrR family transcriptional regulator |
| RJ610_25150 | WND80524.1 | 1,12 | 5,61E-01 | | | efflux RND transporter periplasmic adaptor subunit |
| RJ610_25155 | WND80525.1 | 1,38 | 9,62E-03 | | | efflux RND transporter permease subunit |

| | | | | | | |
|---|---|---|---|---|---|---|
| RJ610_25160 | WND80526.1 | 1,00 | 9,90E-01 | | | outer membrane protein transport protein |
| RJ610_25165 | WND80527.1 | 1,12 | 4,71E-01 | | | hypothetical protein |
| RJ610_25170 | WND80528.1 | 1,02 | 8,81E-01 | | | serine hydrolase domain-containing protein |
| RJ610_25175 | WND80529.1 | 1,41 | 5,73E-02 | | | hypothetical protein |
| RJ610_25180 | WND80530.1 | 1,05 | 8,58E-01 | | | hypothetical protein |
| RJ610_25185 | WND80531.1 | 0,70 | 4,81E-02 | | | DUF2306 domain-containing protein |
| RJ610_25190 | WND80532.1 | 0,75 | 9,57E-02 | | | hypothetical protein |
| RJ610_25195 | WND80533.1 | 0,88 | 6,98E-01 | | | hypothetical protein |
| RJ610_25200 | WND80534.1 | 1,19 | 2,19E-01 | | | hypothetical protein |
| RJ610_25205 | WND80535.1 | 1,94 | 3,00E-10 | | | SRPBCC domain-containing protein |
| RJ610_25210 | WND80536.1 | 1,11 | 4,03E-01 | | | DUF4166 domain-containing protein |
| RJ610_25215 | WND80537.1 | 2,24 | 5,17E-06 | | | hypothetical protein |
| RJ610_25220 | WND80538.1 | 1,44 | 2,64E-03 | | | hypothetical protein |
| RJ610_25225 | WND80539.1 | 21,84 | 2,19E-66 | | | hypothetical protein |
| RJ610_25230 | WND80540.1 | 2,62 | 1,22E-17 | | | hypothetical protein |
| RJ610_25235 | WND80541.1 | 2,56 | 2,51E-10 | | | IS1595 family transposase |
| RJ610_25240 | WND80542.1 | 1,63 | 2,63E-02 | | | hypothetical protein |
| RJ610_25245 | WND80543.1 | 0,91 | 3,91E-01 | | | hypothetical protein |
| RJ610_25250 | WND80544.1 | 0,74 | 3,01E-02 | | | DUF4844 domain-containing protein |
| RJ610_25255 | WND80545.1 | 0,71 | 8,40E-04 | | | H-NS histone family protein |
| RJ610_25260 | WND80546.1 | 0,73 | 6,70E-03 | | | DUF4189 domain-containing protein |
| RJ610_25265 | WND80547.1 | 0,57 | 9,24E-08 | | | hypothetical protein |
| RJ610_25270 | WND80548.1 | 0,94 | 7,15E-01 | | | LytTR family DNA-binding domain-containing protein |
| RJ610_25275 | WND80549.1 | 0,98 | 8,79E-01 | | | histidine kinase |
| RJ610_25280 | WND80550.1 | 1,16 | 5,11E-01 | | | nuclear transport factor 2 family protein |
| RJ610_25285 | WND80551.1 | 0,89 | 6,36E-01 | | | hypothetical protein |
| RJ610_25290 | WND80552.1 | 0,72 | 3,38E-02 | | | hypothetical protein |
| RJ610_25295 | WND80553.1 | 0,91 | 4,87E-01 | | | hypothetical protein |
| RJ610_25300 | WND80554.1 | 0,85 | 1,47E-01 | | | hypothetical protein |
| RJ610_25305 | WND80555.1 | 0,93 | 6,59E-01 | | | nucleotidyltransferase domain-containing protein |
| RJ610_25310 | WND80556.1 | 1,10 | 5,16E-01 | | | tyrosine-type recombinase/integrase |
| RJ610_25315 | WND80557.1 | 1,58 | 3,14E-02 | | | hypothetical protein |
| RJ610_25320 | WND80558.1 | 1,09 | 6,46E-01 | | | hypothetical protein |
| RJ610_25325 | WND80559.1 | 0,97 | 8,68E-01 | | | hypothetical protein |
| RJ610_25330 | WND80560.1 | 1,03 | 8,39E-01 | | | hypothetical protein |
| RJ610_25335 | WND80561.1 | 4,16 | 1,35E-26 | | | hypothetical protein |
| RJ610_25340 | WND80562.1 | 61,99 | 1,97E-200 | | | cupin domain-containing protein |
| RJ610_25345 | WND80563.1 | 1,86 | 6,71E-08 | | | arsenic resistance N-acetyltransferase ArsN2 |
| RJ610_25350 | WND80564.1 | 4,28 | 5,67E-22 | | | hypothetical protein |
| RJ610_25355 | WND80565.1 | 1,32 | 1,35E-01 | | | hypothetical protein |
| RJ610_25360 | WND80566.1 | 0,41 | 2,30E-07 | | | hypothetical protein |
| RJ610_25365 | WND80567.1 | 0,95 | 8,44E-01 | | | MarR family transcriptional regulator |
| RJ610_25370 | WND80568.1 | 0,73 | 1,31E-02 | | | serine hydrolase domain-containing protein |

| | | | | | | |
|---|---|---|---|---|---|---|
| RJ610_25375 | WND80569.1 | 0,86 | 2,48E-01 | | | MFS transporter |
| RJ610_25380 | WND80570.1 | 0,83 | 1,50E-01 | | | siderophore-interacting protein |
| RJ610_25385 | WND80571.1 | 0,79 | 8,78E-02 | | | PadR family transcriptional regulator |
| RJ610_25390 | WND80572.1 | 2,42 | 2,32E-09 | | | hypothetical protein |
| RJ610_25395 | WND80573.1 | 4,70 | 1,01E-19 | | | hypothetical protein |
| RJ610_25400 | WND80574.1 | 1,61 | 2,86E-03 | | | hypothetical protein |
| RJ610_25405 | WND80575.1 | 4,00 | 3,23E-15 | | | hypothetical protein |
| RJ610_25410 | WND80576.1 | 1,13 | 4,00E-01 | | | barstar family protein |
| RJ610_25415 | WND80577.1 | 1,02 | 8,57E-01 | | | TetR/AcrR family transcriptional regulator |
| RJ610_25420 | WND80578.1 | 1,42 | 6,00E-04 | | | MBL fold metallo-hydrolase |
| RJ610_25425 | WND80579.1 | 1,60 | 8,89E-03 | | | hypothetical protein |
| RJ610_25435 | WND80581.1 | 3,48 | 5,67E-12 | | | SgcJ/EcaC family oxidoreductase |
| RJ610_25440 | WND80582.1 | 1,29 | 1,86E-01 | | | hypothetical protein |
| RJ610_25445 | WND80583.1 | 1,66 | 1,42E-03 | | | hypothetical protein |
| RJ610_25450 | WND80584.1 | 1,43 | 9,04E-04 | | | hypothetical protein |
| RJ610_25455 | WND80585.1 | 0,81 | 9,69E-02 | | | glycoside hydrolase family 16 protein |
| RJ610_25460 | WND80586.1 | 3,62 | 2,47E-18 | | | M23 family metallopeptidase |
| RJ610_25465 | WND80587.1 | 1,42 | 1,46E-03 | | | methyltransferase domain-containing protein |
| RJ610_25470 | WND80588.1 | 1,01 | 9,37E-01 | | | helix-turn-helix domain-containing protein |
| RJ610_25475 | WND80589.1 | 0,87 | 4,72E-01 | | | NAD(P)H-dependent oxidoreductase |
| RJ610_25480 | WND80590.1 | 1,76 | 4,18E-02 | | | nuclear transport factor 2 family protein |
| RJ610_25485 | WND80591.1 | 1,61 | 4,44E-02 | | | MBL fold metallo-hydrolase |
| RJ610_25490 | WND80592.1 | 1,05 | 7,98E-01 | | | thioesterase family protein |
| RJ610_25495 | WND80593.1 | 0,82 | 1,33E-01 | | | hypothetical protein |
| RJ610_25500 | WND80594.1 | 0,91 | 6,00E-01 | | | hypothetical protein |
| RJ610_25505 | WND80595.1 | 0,83 | 1,68E-01 | | | hypothetical protein |
| RJ610_25510 | WND80596.1 | 0,74 | 1,65E-03 | | | helix-turn-helix transcriptional regulator |
| RJ610_25515 | WND80597.1 | 0,49 | 6,04E-07 | | | hypothetical protein |
| RJ610_25520 | WND80598.1 | 0,53 | 1,41E-07 | | | hypothetical protein |
| RJ610_25525 | WND80599.1 | 0,46 | 1,58E-08 | | | hypothetical protein |
| RJ610_25530 | WND80600.1 | 0,40 | 3,00E-09 | | | hypothetical protein |
| RJ610_25535 | WND80601.1 | 0,39 | 5,76E-09 | | | hypothetical protein |
| RJ610_25540 | WND80602.1 | 0,41 | 3,83E-07 | | | hypothetical protein |
| RJ610_25545 | WND80603.1 | 0,91 | 7,13E-01 | | | VOC family protein |
| RJ610_25550 | WND80604.1 | 0,51 | 1,89E-03 | | | response regulator |
| RJ610_25555 | WND80605.1 | 0,84 | 3,82E-01 | | | ATPase domain-containing protein |
| RJ610_25560 | WND80606.1 | 1,03 | 9,10E-01 | | | response regulator |
| RJ610_25565 | WND80607.1 | 1,44 | 2,25E-02 | | | beta-1,3-glucanase family protein |
| RJ610_25570 | WND80608.1 | 1,10 | 6,03E-01 | | | serine hydrolase domain-containing protein |
| RJ610_25575 | WND80609.1 | 0,71 | 4,26E-02 | | | LytTR family DNA-binding domain-containing protein |
| RJ610_25580 | WND80610.1 | 0,62 | 2,74E-03 | | | histidine kinase |
| RJ610_25585 | WND80611.1 | 0,49 | 5,59E-08 | | | Na+/H+ antiporter |
| RJ610_25590 | WND80612.1 | 0,55 | 3,77E-05 | | | hypothetical protein |

| | | | | | | |
|---|---|---|---|---|---|---|
| RJ610_25595 | WND80613.1 | 0,55 | 1,23E-04 | | | AAA family ATPase |
| RJ610_25600 | WND80614.1 | 0,61 | 5,05E-04 | | | glutathione S-transferase family protein |
| RJ610_25605 | WND80615.1 | 0,79 | 4,11E-02 | | | TetR/AcrR family transcriptional regulator |
| RJ610_25610 | WND80616.1 | 0,75 | 3,20E-02 | | | hypothetical protein |
| RJ610_25615 | WND80617.1 | 0,50 | 8,03E-09 | | | hypothetical protein |
| RJ610_25620 | WND80618.1 | 0,94 | 5,77E-01 | | | amidase |
| RJ610_25625 | WND80619.1 | 0,93 | 6,95E-01 | | | GNAT family N-acetyltransferase |
| RJ610_25630 | WND80620.1 | 2,29 | 2,20E-06 | | | hypothetical protein |
| RJ610_25635 | WND80621.1 | 0,79 | 1,38E-01 | | | PD40 domain-containing protein |
| RJ610_25640 | WND80622.1 | 0,41 | 6,77E-12 | | | hypothetical protein |
| RJ610_25645 | WND80623.1 | 0,58 | 1,05E-03 | | | LysR substrate-binding domain-containing protein |
| RJ610_25650 | WND80624.1 | 1,08 | 6,28E-01 | | | alpha/beta hydrolase |
| RJ610_25655 | WND80625.1 | 0,63 | 6,45E-03 | | | LysR family transcriptional regulator |
| RJ610_25665 | WND80627.1 | 1,27 | 6,14E-02 | | | hypothetical protein |
| RJ610_25670 | WND80628.1 | 1,73 | 1,00E-05 | | | hypothetical protein |
| RJ610_25675 | WND80629.1 | 0,71 | 2,10E-03 | | | DUF2268 domain-containing putative Zn-dependent protease |
| RJ610_25680 | WND80630.1 | 0,52 | 9,69E-09 | | | thioesterase family protein |
| RJ610_25685 | WND80631.1 | 1,06 | 7,16E-01 | | | transcriptional regulator GcvA |
| RJ610_25690 | WND80632.1 | 0,85 | 5,19E-01 | | | MBL fold metallo-hydrolase |
| RJ610_25695 | WND80633.1 | 0,51 | 3,37E-03 | | | MBL fold metallo-hydrolase |
| RJ610_25700 | WND80634.1 | 0,62 | 4,94E-05 | | | phosphoglucomutase (alpha-D-glucose-1,6-bisphosphate-dependent) |
| RJ610_25705 | WND83351.1 | 0,56 | 3,38E-02 | | | ACR3 family arsenite efflux transporter |
| RJ610_25710 | WND80635.1 | 0,41 | 1,68E-03 | | | helix-turn-helix transcriptional regulator |
| RJ610_25715 | WND80636.1 | 0,53 | 1,92E-02 | | | arsenical resistance protein ArsH |
| RJ610_25720 | WND80637.1 | 0,66 | 7,18E-02 | | | arsenate reductase (glutaredoxin) |
| RJ610_25725 | WND80638.1 | 0,88 | 4,91E-01 | | | bleomycin resistance family protein |
| RJ610_25730 | WND80639.1 | 1,04 | 8,15E-01 | | | winged helix DNA-binding protein |
| RJ610_25735 | WND80640.1 | 1,70 | 4,11E-05 | | | alpha/beta fold hydrolase |
| RJ610_25740 | WND80641.1 | 1,05 | 8,18E-01 | | | serine hydrolase domain-containing protein |
| RJ610_25745 | WND80642.1 | 0,67 | 6,23E-03 | | | hypothetical protein |
| RJ610_25750 | WND83352.1 | 0,64 | 1,71E-03 | | | nuclear transport factor 2 family protein |
| RJ610_25755 | WND83353.1 | 0,53 | 3,90E-05 | | | DUF411 domain-containing protein |
| RJ610_25760 | WND80643.1 | 0,73 | 6,90E-02 | | | cytochrome c |
| RJ610_25765 | WND80644.1 | 0,78 | 1,43E-01 | | | CopL family metal-binding regulatory protein |
| RJ610_25770 | WND80645.1 | 0,54 | 4,58E-07 | | | energy transducer TonB |
| RJ610_25775 | WND80646.1 | 0,35 | 5,66E-03 | | | hypothetical protein |
| RJ610_25780 | WND80647.1 | 0,66 | 8,43E-02 | | | hypothetical protein |
| RJ610_25785 | WND80648.1 | 0,71 | 1,38E-01 | | | toxic anion resistance protein |
| RJ610_25790 | WND80649.1 | 0,56 | 1,40E-03 | | | VWA domain-containing protein |
| RJ610_25795 | WND80650.1 | 0,94 | 7,91E-01 | | | hypothetical protein |
| RJ610_25800 | WND80651.1 | 0,80 | 1,22E-01 | | | hypothetical protein |
| RJ610_25805 | WND80652.1 | 1,27 | 3,78E-01 | | | hypothetical protein |

| | | | | | | |
|---|---|---|---|---|---|---|
| RJ610_25810 | WND80653.1 | 0,92 | 7,35E-01 | | | saccharopine dehydrogenase NADP-binding domain-containing protein |
| RJ610_25815 | WND80654.1 | 0,78 | 8,54E-02 | | | 2Fe-2S iron-sulfur cluster-binding protein |
| RJ610_25820 | WND80655.1 | 0,71 | 1,67E-02 | | | PepSY domain-containing protein |
| RJ610_25825 | WND80656.1 | 0,98 | 9,34E-01 | | | TonB-dependent receptor |
| RJ610_25830 | WND80657.1 | 2,20 | 2,05E-07 | | | CopL family metal-binding regulatory protein |
| RJ610_25835 | WND80658.1 | 1,28 | 7,64E-02 | | | histidine utilization repressor |
| RJ610_25840 | WND80659.1 | 4,15 | 1,59E-32 | | | VOC family protein |
| RJ610_25845 | WND80660.1 | 7,04 | 5,26E-55 | | | hypothetical protein |
| RJ610_25850 | WND80661.1 | 1,85 | 2,66E-04 | | | serine hydrolase domain-containing protein |
| RJ610_25855 | WND80662.1 | 1,43 | 2,63E-02 | | | DUF5694 domain-containing protein |
| RJ610_25860 | WND80663.1 | 1,65 | 7,00E-04 | | | TraB/GumN family protein |
| RJ610_25865 | WND80664.1 | 1,50 | 5,21E-03 | | | cation diffusion facilitator family transporter |
| RJ610_25870 | WND80665.1 | 2,16 | 3,45E-11 | | | hypothetical protein |
| RJ610_25875 | WND80666.1 | 2,42 | 1,38E-14 | | | hypothetical protein |
| RJ610_25880 | WND80667.1 | 1,51 | 1,35E-02 | | | catalase |
| RJ610_25885 | WND80668.1 | 0,92 | 6,88E-01 | | | hypothetical protein |
| RJ610_25890 | WND80669.1 | 0,76 | 1,55E-01 | | | hypothetical protein |
| RJ610_25895 | WND80670.1 | 0,67 | 4,39E-02 | | | hypothetical protein |
| RJ610_25900 | WND80671.1 | 0,79 | 2,85E-01 | | | hypothetical protein |
| RJ610_25905 | WND80672.1 | 0,81 | 8,89E-02 | | | peptidoglycan-binding protein |
| RJ610_25910 | WND80673.1 | 0,77 | 3,34E-01 | | | hypothetical protein |
| RJ610_25915 | WND80674.1 | 0,96 | 8,50E-01 | | | alpha/beta hydrolase |
| RJ610_25920 | WND80675.1 | 0,42 | 3,77E-05 | | | pirin family protein |
| RJ610_25925 | WND80676.1 | 0,93 | 6,54E-01 | | | LysR family transcriptional regulator |
| RJ610_25930 | WND80677.1 | 0,86 | 3,14E-01 | | | HAMP domain-containing sensor histidine kinase |
| RJ610_25935 | WND80678.1 | 0,79 | 2,39E-01 | | | response regulator |
| RJ610_25940 | WND80679.1 | 0,75 | 3,56E-01 | | | thioredoxin family protein |
| RJ610_25945 | WND80680.1 | 0,59 | 9,12E-02 | | | hypothetical protein |
| RJ610_25950 | WND80681.1 | 0,61 | 8,94E-02 | | | putative DNA-binding domain-containing protein |
| RJ610_25955 | WND80682.1 | 0,89 | 8,13E-01 | | | DUF692 domain-containing protein |
| RJ610_25960 | WND80683.1 | 0,98 | 9,69E-01 | | | EF-hand domain-containing protein |
| RJ610_25965 | WND80684.1 | 0,42 | 3,49E-01 | | | DoxX family protein |
| RJ610_25970 | WND80685.1 | 0,82 | 5,53E-01 | | | hypothetical protein |
| RJ610_25975 | WND80686.1 | 0,74 | 5,48E-02 | | | TonB-dependent receptor |
| RJ610_25980 | WND80687.1 | 0,51 | 2,90E-12 | | | hypothetical protein |
| RJ610_25985 | WND80688.1 | 0,39 | 6,48E-18 | | | lipase |
| RJ610_25990 | WND80689.1 | 0,47 | 3,37E-04 | | | NAD(P)H-dependent oxidoreductase |
| RJ610_25995 | WND80690.1 | 0,63 | 1,91E-02 | | | LysR family transcriptional regulator |
| RJ610_26000 | WND80691.1 | 0,72 | 1,34E-01 | | | LysM domain-containing protein |
| RJ610_26005 | WND80692.1 | 1,01 | 9,63E-01 | | | M20/M25/M40 family metallo-hydrolase |
| RJ610_26010 | WND83354.1 | 0,70 | 2,56E-02 | | | hypothetical protein |
| RJ610_26015 | WND80693.1 | 2,26 | 9,86E-04 | | | hypothetical protein |

| | | | | | | |
|---|---|---|---|---|---|---|
| RJ610_26020 | WND80694.1 | 24,27 | 1,07E-31 | | | hypothetical protein |
| RJ610_26025 | WND80695.1 | 20,44 | 9,35E-22 | | | hypothetical protein |
| RJ610_26030 | WND80696.1 | 60,81 | 4,00E-101 | | | S8 family serine peptidase |
| RJ610_26035 | WND80697.1 | 3,10 | 1,93E-10 | | | hypothetical protein |
| RJ610_26040 | WND80698.1 | 3,84 | 6,21E-04 | | | hypothetical protein |
| RJ610_26045 | WND80699.1 | 0,74 | 2,37E-02 | | | histidine kinase |
| RJ610_26050 | WND80700.1 | 0,68 | 2,47E-02 | | | LytTR family DNA-binding domain-containing protein |
| RJ610_26055 | WND80701.1 | 0,64 | 3,71E-03 | | | hypothetical protein |
| RJ610_26060 | WND80702.1 | 1,03 | 8,51E-01 | | | WG repeat-containing protein |
| RJ610_26065 | WND80703.1 | 2,20 | 4,95E-07 | | | YcaO-like family protein |
| RJ610_26070 | WND80704.1 | 3,17 | 6,43E-09 | | | hypothetical protein |
| RJ610_26075 | WND80705.1 | 0,73 | 3,15E-02 | | | aminodeoxychorismate synthase component I |
| RJ610_26080 | WND80706.1 | 1,12 | 4,81E-01 | | | 4'-phosphopantetheinyl transferase superfamily protein |
| RJ610_26085 | WND80707.1 | 20,00 | 4,50E-71 | | | M4 family metallopeptidase |
| RJ610_26090 | WND80708.1 | 1,36 | 1,67E-02 | | | hypothetical protein |
| RJ610_26095 | WND80709.1 | 2,05 | 1,62E-03 | | | M4 family metallopeptidase |
| RJ610_26100 | WND80710.1 | 0,57 | 1,92E-04 | | | glutathionylspermidine synthase family protein |
| RJ610_26105 | WND80711.1 | 0,53 | 9,40E-06 | | | DUF1190 domain-containing protein |
| RJ610_26110 | WND80712.1 | 0,48 | 2,05E-08 | | | DUF350 domain-containing protein |
| RJ610_26115 | WND80713.1 | 0,54 | 4,11E-08 | | | DUF2491 family protein |
| RJ610_26120 | WND80714.1 | 0,56 | 2,49E-04 | | | potassium channel family protein |
| RJ610_26125 | WND80715.1 | 0,59 | 1,06E-03 | | | PspA/IM30 family protein |
| RJ610_26135 | WND80717.1 | 1,36 | 2,62E-03 | | | c-type cytochrome |
| RJ610_26140 | WND80718.1 | 0,57 | 6,48E-05 | | | ExeM/NucH family extracellular endonuclease |
| RJ610_26145 | WND80719.1 | 0,47 | 4,97E-09 | | | alpha/beta fold hydrolase |
| RJ610_26150 | WND80720.1 | 0,53 | 3,66E-09 | | | lysozyme inhibitor LprI family protein |
| RJ610_26155 | WND83355.1 | 0,91 | 4,56E-01 | | | cell division protein ZapE |
| RJ610_26160 | WND80721.1 | 0,67 | 2,40E-03 | | | HU family DNA-binding protein |
| RJ610_26165 | WND80722.1 | 0,61 | 1,09E-06 | | | hypothetical protein |
| RJ610_26170 | WND80723.1 | 0,75 | 2,42E-01 | | | aminoglycoside phosphotransferase family protein |
| RJ610_26175 | WND80724.1 | 0,70 | 5,49E-02 | | | hypothetical protein |
| RJ610_26180 | WND80725.1 | 0,72 | 2,22E-02 | | | efflux transporter outer membrane subunit |
| RJ610_26185 | WND80726.1 | 0,61 | 2,71E-03 | | | efflux RND transporter periplasmic adaptor subunit |
| RJ610_26190 | WND80727.1 | 0,60 | 3,89E-05 | | | hypothetical protein |
| RJ610_26195 | WND80728.1 | 0,43 | 1,11E-06 | | | DUF2569 domain-containing protein |
| RJ610_26200 | WND80729.1 | 0,90 | 5,94E-01 | | | APC family permease |
| RJ610_26205 | WND83356.1 | 0,62 | 4,96E-04 | | | LysR family transcriptional regulator |
| RJ610_26210 | WND80730.1 | 0,83 | 3,45E-01 | | | aldo/keto reductase |
| RJ610_26215 | WND80731.1 | 3,85 | 6,10E-05 | | | hypothetical protein |
| RJ610_26220 | WND80732.1 | 4,41 | 8,17E-14 | | | hypothetical protein |
| RJ610_26225 | WND80733.1 | 11,70 | 1,12E-51 | | | hypothetical protein |
| RJ610_26230 | WND80734.1 | 16,85 | 5,64E-48 | | | hypothetical protein |
| RJ610_26240 | WND80736.1 | 1,91 | 3,86E-08 | | | hypothetical protein |

| | | | | | | |
|---|---|---|---|---|---|---|
| RJ610_26245 | WND80737.1 | 0,82 | 1,61E-01 | | | amidohydrolase |
| RJ610_26250 | WND80738.1 | 0,56 | 9,35E-09 | | | NADPH-dependent 7-cyano-7-deazaguanine reductase QueF |
| RJ610_26255 | WND80739.1 | 0,51 | 1,96E-04 | | | LysM peptidoglycan-binding domain-containing protein |
| RJ610_26260 | WND80740.1 | 0,76 | 9,32E-02 | | | hypothetical protein |
| RJ610_26265 | WND80741.1 | 1,60 | 1,23E-04 | | | YchJ family metal-binding protein |
| RJ610_26270 | WND80742.1 | 0,60 | 3,51E-04 | | | UPF0149 family protein |
| RJ610_26275 | WND80743.1 | 0,76 | 7,77E-02 | | | YkgJ family cysteine cluster protein |
| RJ610_26280 | WND80744.1 | 1,00 | 9,95E-01 | | | hypothetical protein |
| RJ610_26285 | WND80745.1 | 0,75 | 3,61E-02 | | | DUF4288 domain-containing protein |
| RJ610_26290 | WND80746.1 | 0,82 | 1,75E-01 | | | hypothetical protein |
| RJ610_26295 | WND80747.1 | 0,85 | 3,67E-01 | | | hypothetical protein |
| RJ610_26305 | WND80749.1 | 0,24 | 1,95E-37 | | | DUF4344 domain-containing metallopeptidase |
| RJ610_26310 | WND80750.1 | 1,32 | 2,63E-02 | | | hypothetical protein |
| RJ610_26315 | WND80751.1 | 1,27 | 1,19E-01 | | | hypothetical protein |
| RJ610_26320 | | 1,96 | 6,87E-04 | | | IS1595 family transposase |
| RJ610_26325 | WND80752.1 | 0,65 | 7,30E-03 | | | hypothetical protein |
| RJ610_26330 | WND80753.1 | 0,44 | 1,68E-05 | | | bifunctional isocitrate dehydrogenase kinase/phosphatase |
| RJ610_26335 | WND80754.1 | 0,85 | 3,15E-01 | | | hypothetical protein |
| RJ610_26340 | WND80755.1 | 1,08 | 7,05E-01 | | | hypothetical protein |
| RJ610_26345 | | 0,87 | 3,97E-01 | | | LysR substrate-binding domain-containing protein |
| RJ610_26350 | WND83357.1 | 0,51 | 1,47E-08 | | | methyltransferase |
| RJ610_26355 | WND80756.1 | 0,50 | 1,55E-08 | | | hypothetical protein |
| RJ610_26360 | WND80757.1 | 0,82 | 9,98E-02 | | | pseudouridine synthase |
| RJ610_26365 | WND80758.1 | 0,92 | 6,11E-01 | | | hypothetical protein |
| RJ610_26380 | WND80761.1 | 0,56 | 7,41E-06 | | | DMT family transporter |
| RJ610_26385 | WND80762.1 | 0,81 | 1,35E-01 | | | aldo/keto reductase |
| RJ610_26390 | WND80763.1 | 2,94 | 1,28E-24 | | | hybrid sensor histidine kinase/response regulator |
| RJ610_26395 | WND80764.1 | 0,26 | 6,14E-16 | | | hypothetical protein |
| RJ610_26400 | WND80765.1 | 0,54 | 1,68E-06 | | | ATP-dependent helicase HrpB |
| RJ610_26405 | WND80766.1 | 1,01 | 9,82E-01 | | | hypothetical protein |
| RJ610_26410 | WND80767.1 | 0,55 | 1,42E-06 | | | TatD family hydrolase |
| RJ610_26415 | WND80768.1 | 0,76 | 1,42E-01 | | | hypothetical protein |
| RJ610_26420 | WND80769.1 | 5,12 | 1,77E-20 | | | TonB-dependent receptor |
| RJ610_26425 | WND80770.1 | 1,26 | 2,83E-01 | | | hypothetical protein |
| RJ610_26430 | WND80771.1 | 0,56 | 1,45E-04 | | | hypothetical protein |
| RJ610_26435 | WND80772.1 | 0,67 | 5,66E-03 | | | ABC transporter permease |
| RJ610_26440 | WND80773.1 | 0,61 | 9,09E-05 | | | ABC transporter ATP-binding protein |
| RJ610_26445 | WND80774.1 | 0,90 | 5,08E-01 | | | ferredoxin--NADP reductase |
| RJ610_26450 | WND80775.1 | 0,59 | 7,58E-06 | | | alpha/beta hydrolase |
| RJ610_26455 | WND80776.1 | 0,53 | 1,41E-07 | | | ATP-binding cassette domain-containing protein |
| RJ610_26460 | WND80777.1 | 0,59 | 1,11E-05 | | | ABC transporter permease |
| RJ610_26465 | WND80778.1 | 0,81 | 2,31E-01 | | | SMI1/KNR4 family protein |
| RJ610_26470 | WND80779.1 | 0,91 | 7,90E-01 | | | SMI1/KNR4 family protein |

| | | | | | | |
|---|---|---|---|---|---|---|
| RJ610_26475 | WND80780.1 | 0,99 | 9,80E-01 | | | energy transducer TonB |
| RJ610_26480 | WND80781.1 | 0,59 | 8,98E-07 | | | SPFH domain-containing protein |
| RJ610_26485 | WND80782.1 | 0,76 | 1,37E-02 | | | hypothetical protein |
| RJ610_26490 | WND80783.1 | 0,98 | 9,20E-01 | | | hypothetical protein |
| RJ610_26495 | WND80784.1 | 1,05 | 8,03E-01 | | | MFS transporter |
| RJ610_26500 | WND80785.1 | 1,11 | 4,57E-01 | | | 2,5-didehydrogluconate reductase DkgB |
| RJ610_26505 | WND80786.1 | 0,97 | 8,46E-01 | | | alkene reductase |
| RJ610_26510 | WND80787.1 | 0,78 | 1,99E-02 | | | DEAD/DEAH box helicase family protein |
| RJ610_26515 | WND80788.1 | 0,75 | 4,53E-03 | | | site-specific DNA-methyltransferase |
| RJ610_26520 | WND80789.1 | 0,78 | 1,06E-01 | | | hypothetical protein |
| RJ610_26525 | WND80790.1 | 0,56 | 4,86E-06 | | | tRNA uridine-5-carboxymethylaminomethyl(34) synthesis GTPase MnmE |
| RJ610_26530 | WND80791.1 | 0,85 | 1,23E-01 | | | membrane protein insertase YidC |
| RJ610_26535 | WND80792.1 | 1,00 | 9,98E-01 | | | ribonuclease P protein component |
| RJ610_26540 | WND80793.1 | 1,07 | 7,18E-01 | | | 50S ribosomal protein L34 |

| | | | | | | |
|---|---|---|---|---|---|---|
| RJ610_26475 | WND80780.1 | 0,99 | 9,80E-01 | | | energy transducer TonB |
| RJ610_26480 | WND80781.1 | 0,59 | 8,98E-07 | | | SPFH domain-containing protein |
| RJ610_26485 | WND80782.1 | 0,76 | 1,37E-02 | | | hypothetical protein |
| RJ610_26490 | WND80783.1 | 0,98 | 9,20E-01 | | | hypothetical protein |
| RJ610_26495 | WND80784.1 | 1,05 | 8,03E-01 | | | MFS transporter |
| RJ610_26500 | WND80785.1 | 1,11 | 4,57E-01 | | | 2,5-didehydrogluconate reductase DkgB |
| RJ610_26505 | WND80786.1 | 0,97 | 8,46E-01 | | | alkene reductase |
| RJ610_26510 | WND80787.1 | 0,78 | 1,99E-02 | | | DEAD/DEAH box helicase family protein |
| RJ610_26515 | WND80788.1 | 0,75 | 4,53E-03 | | | site-specific DNA-methyltransferase |
| RJ610_26520 | WND80789.1 | 0,78 | 1,06E-01 | | | hypothetical protein |
| RJ610_26525 | WND80790.1 | 0,56 | 4,86E-06 | | | tRNA uridine-5-carboxymethylaminomethyl(34) synthesis GTPase MnmE |
| RJ610_26530 | WND80791.1 | 0,85 | 1,23E-01 | | | membrane protein insertase YidC |
| RJ610_26535 | WND80792.1 | 1,00 | 9,98E-01 | | | ribonuclease P protein component |
| RJ610_26540 | WND80793.1 | 1,07 | 7,18E-01 | | | 50S ribosomal protein L34 |

**Supplementary file Table S4**. All sequencing and alignment statistics of the proteomic and transcriptomic results.
